# Supplementary material for: Protocol for community-driven selection of strategies to implement evidence-based practices to reduce opioid overdoses in the HEALing Communities Study: a trial to evaluate a community-engaged intervention in Kentucky, Massachusetts, New York and Ohio
Source: BMJ Open. 2022 Sep 19;12(9):e059328. doi: 10.1136/bmjopen-2021-059328 (PMC9486330; doi:10.1136/bmjopen-2021-059328)
Supplement: Supplementary data [file bmjopen-2021-059328supp001.pdf]

Appendix 1. Kentucky Action Planning Tools

Community Goal Setting Tool

Purpose: To describe existing infrastructure, gaps, opportunities, needs, and goals related to opioid overdose prevention.

Description: The Kentucky Community Engagement and Continuum of Care teams partnered to facilitate communities' identification of their strengths and opportunities for expansion related to opioid overdose prevention followed by thoughtful consideration and selection of the ORCCA strategies. The process began with the Community Engagement team working with the coalition to describe existing infrastructure, gaps, opportunities, needs, and goals related to opioid overdose prevention in their communities using a Community Goal Setting Tool. The coalitions and/or menu-specific workgroups worked on completing the tool for each menu separately and presenting them to the full coalition.

Who completes the tool: Coalitions and/or coalition workgroups with facilitation by faculty and staff.

Community Goal Setting Tool — Menu 1

Menu 1 Goal: Increase Opioid Overdose Prevention Education and Naloxone Distribution (OEND)

Brainstorm! Think about your community: what you've experienced, what you learned from your community profile and data dashboard, and what you envision for the future. Answer the questions below and jot down your thoughts

What are we doing well? What OEND services already exist?

Ex. There is active Opioid Overdose Education and Naloxone Distribution (OEND) already occurring in the County Detention Center. The Detention Center is currently able to provide naloxone to all persons released from the facility (as part of partnership with the HEALing Communities Study) and is also partnering with other organizations to distribute naloxone to anyone who is interested and completes the requisite virtual training.

Who needs OEND in our community?

Ex. There is still a significant need for distribution among the peer community and their social networks, in homes with bystanders, and with medical and law enforcement professionals in contact with those at high-risk for overdose.

Where in our community should OEND services be provided?

Ex. There is significant opportunity for OEND expansion in Emergency Departments and the healthcare system generally, in pharmacies, with local Quick Response Teams (QRTs), in Substance Use Disorder (SUD) treatment facilities, and in other locations where the peer community can be found. The NKY Health Department also has capacity to expand OEND services.

Community OEND Goals:

Example:

1. Expand OEND in criminal justice settings, including Probation & Parole and Drug Court.
2. Expand OEND in healthcare settings, including Emergency Departments and Substance Use Disorder (SUD) treatment programs.
3. Expand OEND access among peer community and their support systems (via recovery support groups, social service agencies, and in "hot spots" frequented by those at high-risk, etc.).
4. Expand OEND with local QRTs.
5. Increase first responders carrying/administering naloxone.

Decision Aid Tool

Purpose: To facilitate discussion and prioritization of evidence-based practice strategies that align with community goals and the ORCCA

Description: HCS staff then populated menu-specific Decision Aid Tools with strategies listed in the ORCCA and TAG, integrating coalition's suggestions for specific strategies when available from Community Goal Setting Tool. In meetings facilitated by faculty and staff with expertise in community-engagement and the continuum of care, the coalitions and/or menu-specific workgroups reviewed each possible strategy one-by-one, offering a narrative description of factors that they believed would be relevant to deciding whether to implement the strategy (i.e., current relevant activities, barriers they anticipate encountering, agencies relevant to implementation). After offering a narrative description, the coalition scored on Likert scales the size of the current gap in the service/strategy (4-point scale; 0-3), feasibility of implementation within the next 12 months (4-point scale; 0-3), and its potential impact on overdose deaths (3-point scale; 1-3). Each dimension was scored through consensus or through in-meeting polling. Coalitions did not use specific criteria or thresholds in assigning the scores, rather they often scored them based on how they compared to others on the menu. Scores were summed to produce an overall "priority score" for each strategy.

Who completes the tool: Coalitions and/or coalition workgroups with facilitation by faculty and staff.

Menu 2: Strategies to Enhance Delivery of MOUD Maintenance Treatment, Including Agonist/Partial Agonist Medication					
**Strategy and Venue**	**Current Activity**	**Size of Gap** (Current Activity vs. Need)	**Feasibility in <12 Months**	**Impact on Overdose Deaths**	**Priority Score**
Working with the ORCCA menu, brainstorm possible strategies to meet the Community MOUD goals. Record all strategies in this column	Use this column to briefly summarize current activity in your community	0 None<br>1 Small<br>2 Medium<br>3 Large	0 Extremely Low<br>1 Small<br>2 Medium<br>3 Large	 1 Small<br>2 Medium<br>3 Large	Add previous 3 columns; if any column contains 0, priority score is 0
**a) Expand MOUD Treatment Availability (Capacity Building) – REQUIRED**					
**Add/expand MOUD in primary care, other general medical/BH settings, and specialty addiction/SUD & recovery programs**					
Ex. Expand Methadone in county OTPs 2.a.1.2	Ex. Have two OTP providers but one has long wait times; need to figure out what they need to be able to treat more patients				
**Add/expand MOUD treatment in Criminal Justice Settings (pre trial, jail, prisons, probation, parole)**					
Ex. Expand prescription of XR naltrexone and buprenorphine within jail	Ex. Interest in XR naltrexone at the jail and jail might be open but have a lot of concerns/barriers				
**Expand access to MOUD through telemedicine, interim buprenorphine or methadone, or medication units**					
Ex. Expand use of Telemedicine/telehealth/tele consults for buprenorphine treatment	Ex. Telehealth opportunities have grown due to COVID 19, but it is unknown whether this option will remain available.				
**b) Interventions to Link to MOUD – REQUIRED**					
**Linkage Programs (All Relevant settings)**					
Ex. Develop linkage program for incarcerated individuals about to be released (with peer support and/or other staff)	Ex. No current activity, the jail is somewhat interested but there are several barriers				
**Bridging MOUD medications as Linkage Adjunct (all relevant settings)**					
Ex. Expand bridge programs within county ED and inpatient settings	Ex. An existing bridge program is working well and can use as model for others. Difficulty with staffing & infrastructure is main barrier				
**c) MOUD Treatment Engagement and Retention – REQUIRED**					
**Enhancement of clinical delivery approaches that support engagement and retention**					
Ex. Expand recovery support services (peer support, telephone recovery support, recovery coaching, family support) in criminal justice settings (Voices of Hope)	Ex. Add peer supports within system; no current long term supports, or programs/mentorship programs engaged				
**Use virtual retention approaches (e.g. mobile, web, digital therapeutics)**					
Ex. Implement use of [illegible] app to improve retention, incentives, and education 2.c.3	Ex. Some clinics within county already using it; potential cost barriers and potential disinterest from buprenorphine providers				
**Utilize retention care coordinators**					
Ex. Utilize retention care navigators to increase/add care navigators in all MOUD treatment settings 2.c.1	Ex. Other care navigation efforts are already underway that could be leveraged, and there is a desire to increase support of and leverage local efforts where possible				
**Integrate mental health and polysubstance use treatment into MOUD care**					
Ex. Offer regular training between primary care/non MH providers with addiction psychiatry and other MH providers to manage co-morbidity with OUD	Ex. Unsure if providers would be interested in engaging				
**Reduce barriers to housing, transportation, childcare, and access to other community benefits for People with OUD**					
Expand transportation services/offerings to align with MOUD treatment center services (location, hours, etc.)	Ex. Local agency offers transportation services to clients				

Action Planning Tool

Purpose: To facilitate discussion and final strategy selection.

Description: The list of strategies discussed in the Decision Aid Tool was used to pre-populate an Action Planning Tool. The tool listed all strategies with their scores and new implementation feasibility ratings (High, Medium, Low) provided by faculty with expertise in implementation science and the continuum of care based on their knowledge of policy- and payor-level barriers and time required for scale up. The pre-populated Action Planning Tools were presented to coalitions for discussion and strategy selection through consensus.

Who completes the tool: Coalitions and/or coalition workgroups with facilitation by faculty and staff.

Action Planning Tool — Menu 3

For each strategy from the Decision Aid that had a priority score above 0, fill in the strategy and venue (i.e., from Column 1 of the Decision Aid), record the scores from the Decision Aid Tool in the Scores columns, and fill in the relevant sector. As coalitions consider their selection of strategies, they should ensure that strategies collectively will target those at highest risk (i.e., people with history of prior overdose, reduced opioid tolerance, who engage in polydrug use, with major medical or mental illness, and/or who inject drugs). The HCS Research Team will help you complete the Implementation Feasibility column by providing insight on whether each strategy has a high, medium, or low feasibility of implementation based on resources, partnerships, and other factors. After reviewing content of the other columns, coalitions should come to consensus on the Overall Priority Ranking with the highest priority strategy ranked 1, the second highest priority ranked 2, and so on. Of note, the Overall Priority Ranking may or may not correspond directly to the rank order from the other scoring columns. After completing this tool, the next step will involve creating an initial implementation plan for the top 1-3 overall priority ranked strategies; at least one of the strategies must be from the required sub-menu (i.e., Safe Prescribing/Dispensing).

Menu 3: Strategies to Improve Prescription Opioid Safety

Strategy and Venue	Scores				Sector?	Implementation Feasibility? (High/Medium/Low)	Overall Priority Ranking
	G	F	I	PS			
**a) Safer Opioid Prescribing/Dispensing Practices (Required)**							
Ex. Education materials around safe opioid disposal, targeting healthcare providers and patients	3	3	2.25	8.25	HC	High	1
Ex. Education for community pharmacists focusing on red flags during opioid dispensing (dose, indication, duration, concurrent therapies, comorbid conditions, age, etc.)	1.25	2.5	1.5	5.25	HC	High	2
Ex. Educational outreach and academic detailing related to pain management for patients with OUD	2.5	2	2	6.5	HC	Medium	
Ex. Patient educational materials regarding appropriate chronic pain management made available in AA meetings and OUD recovery groups	2.5	2.25	1.75	6.5	BH	Medium	3
**b) Safer Opioid Disposal Practices (Optional)**							
Ex. Installation of permanent disposal kiosks	1.8	2.7	2	6.5	HC	High	4
Ex. Expansion and promotion of take-back events	2.3	2.3	2.25	6.85	Other	Medium	

Priority Score: G: Gap; F: Feasibility; I: Impact; PS: Overall Priority Score

Sector: CJ = Criminal Justice, HC = Healthcare, BH = Behavioral Health (See ORCCA Overview for details on assignment of sectors).

Coalition Implementation Planning Tool

Purpose: To more thoroughly define strategies and generate potential lists of venues/agencies to be involved in the implementation

Description: The tool also captured coalition's feedback on what they could do to help support implementation, strategies to ensure reach to under-represented populations, and indicators of success or failure of the strategy. Faculty with expertise in implementation science and the continuum of care added details on what the HCS faculty and staff would do to implement the strategy. Once complete, the tools were reviewed and approved in their entirety by the coalitions.

Who completes the tool: Coalitions and/or coalition workgroups with faculty and staff.

Coalition Implementation Planning Tool — Menu 3

For at least one but no more than three of the strategies with the top Overall Ranking from the Action Plan table, create an initial implementation plan using the table below (i.e., one table per strategy for up to three strategies). At least one of these strategies must come from the required sub-menu in the action plan (i.e., must address safe prescribing/dispensing practices). Provide information about specific activities in as much detail as is feasible at this time; insert additional rows as needed to describe these activities for the coalition and the HCS team.

**Menu 3 Overall Ranking = 1**<br>**Strategy: Educational outreach and academic detailing related to opioid prescribing for routine pain management. (Includes multiple sub-strategies as outlined on the Community Action Planning Tool).**			
**What specifically will be done?** Ex. Educational outreach and academic detailing for all willing opioid prescribers (and possibly ambulatory pharmacists) in the County. Top priorities are: Primary Care, Hospital Emergency Department & local Urgent Care facilities, Dental Offices, Ortho Practices, Pain Management Clinics			
**What organization(s) should be involved? Provide points of contact if possible.**	Agency: Ex. Family Practice	Contact: Ex. Dr. Smith	
	Agency: Ex. Pharmacy	Contact: Ex. Dr. Jones	
	Agency:	Contact:	
**What will the coalition do?**			**Assigned to:**
1. Ex. Facilitate contact with Family Practice, Ambulatory Pharmacists			1. Ex. Coalition Chair
2. Ex. Help with endorsement letter (with signatures from local hospital MDs			2. Ex. Safe Prescribing Champion
**What will HCS do?**			**Assigned to:**
1. Coordinate with listed coalition members to connect with identified organizations.			1. Ex. Prevention Team
2. Draft a promotional letter/email to be reviewed and signed by community leaders who healthcare professionals respect and would positively respond to and recognize.			2. Ex. Prevention Team
3. Develop community-specific implementation plan, target list, and educational materials using audiences and messaging the coalition identifies.			3. Ex. Prevention Team and Community Coordinator
**How could we promote access/impact among diverse populations?**	Ex. Provide all patient-facing materials in Spanish, English, and any other languages deemed necessary; Prioritize County FQHC provider		
**How will we know it is working?**	Ex. Providers are willing to meet and listen/engage with continuing education materials, Providers agree to provide/request patient materials for distribution		
**What will make us change our strategy?**	Ex. Providers are not responsive or willing to engage, Educational materials are not reaching patients.		

Appendix 2. Massachusetts Action Planning Tools

Community Profile Summary Form

Purpose: Discuss and Prioritize EBP Strategies that Align with Community Goals

Description: Developing ORCCA-specific Community Goals was a multi-step iterative process driven by community teams (CEFs, CDMs, Community Faculty and CCs). Small group conversations and 1:1s were scheduled with coalition members, local stakeholders, and champions. Community Faculty set the stage with stakeholders by sharing data that demonstrated the impact a combination of initiation on medications for opioid use disorder (MOUD), improvement on MOUD retention, and increasing the distribution of naloxone could have on the HCS communities. These data facilitated conversations about the potential impact of different EBPs. Teams outlined the importance of increasing access to naloxone, MOUD initiation, and MOUD retention within the context of each community. Together, community teams and stakeholders used community profile information to contextualize these data for their community. Community profiles, which included an overview of opioid-related risk, available treatment, and fatal overdose prevention services in the community, were co-created by study staff and community members. The profile, along with local data and coalition insight was used to identify areas of opportunity where ORCCA related strategies could fill unmet needs and/or enhance extant programming. This information was compiled on the community profile summary form.

Who completes the tool: This was an iterative process that involved community teams and subcommittees soliciting feedback from coalition members, dialoging, vetting ideas and ranking solutions as they grappled with how to maximize impact among high-risk populations.

Massachusetts HCS Wave 1 Community Profile Summary

Community Profile Summary **Coalition: _______________**

ORCCA Interventions	Existing Interventions (currently taking place)	Who are the service providers?	High-risk Populations (currently receiving services)	Existing programmatic collaborations (referrals and partnerships)	Areas of opportunity to improve existing services (to identify gaps)	Sustainability Include: Current funding sources and duration	Community Narratives
**Objective 1: Increase Opioid Overdose Prevention Education and Naloxone Distribution (OEND)**							
Required OEND							
Optional OEND							
Naloxone administration (optional)							
**Objective 2: Outreach and Delivery of Medications for Opioid Use Disorder (MOUD) to High-Risk Populations***							
Expand MOUD treatment (required)							
Linkage to MOUD (required)							
Engagement and retention (required)							
**Objective 3: Improve Prescription Opioid Safety**							
Safer Prescribing/ Dispensing (required)							
Safer Disposal Practices (optional)							

*For Definitions of "high-risk populations" see the Section II of the ORCCA Overview

Action Plan Summary Form

Purpose: To comprehensively outline strategies and determine their fiscal feasibility.

Description: Once the community profile summary was complete, community teams and coalitions began the process of selecting specific evidence-based practices. This involved participating in workshops and trainings to learn more about evidenced based strategies supported by the study, conducting 1:1s to engage potential implementation partners through coalition contacts, and assessing the extent to which high-risk populations and venues were engaged with the coalition and conducting outreach. Subgroups engaged in focused conversations exploring potential evidence-based practices using knowledge from previous conversations about evidence-based practices, as well as their feasibility, impact, and sustainability.

Community partners created budgets and estimates that were included later in proposals for action plan items. The action plan summary information was then discussed in subgroup meetings. During subgroup meetings coalition members and partner organizations further flushed out details of their plans refining them for presentation to the larger coalition. At the coalition meeting action plan summaries were discussed and voted on by the coalition membership using decision-making procedures such as consensus or majority voting as outlined in their respective coalition charters.

Who completes the tool: This was an iterative process that involved community teams, subcommittees, and partner organizations.

Massachusetts HCS Wave 1 Community Profile Summary & Action Plan Template

Massachusetts Action Plan Summary **Coalition: XXX**

	Selected Strategies	ORCCA Goal (How it advances the overall HCS goal?	Targeted Populations	Primary High-Risk Sector			Specific Activities Needed to Implement Selected Strategy: (who, what, when, where)	TA Needed	HCS Funds Needed
				BH	CJ	HC			
**ORCCA Objective 2: Outreach and Delivery of MOUD to High-Risk Populations**									
MOUD Linkage (required)	H. Linkage program via embedding an "in-reach worker" at XXX County Correctional Facility	Through the addition of this staff member, inmates leaving the facility will be provided with naloxone and a MOUD treatment appointment, with transportation provided, on release date.<br><br>Worker will be responsible for: creating relationships with inmates, running harm reduction groups, distributing Narcan, making community connections, scheduling same day appointments, scheduling transportation as needed.	People with reduced tolerance post-incarceration		X		X, Y, and Z orgs will work collaboratively to create a 30-40 hour/week position to fill gap between leaving incarceration and returning home in order to support recovery and lower risk of overdose after incarceration. Facility will determine number of residents released to determine number of hours for staff to spend in the facility.<br><br>Representatives from all 3 organizations will create job description and conduct interview to ensure right fit to work with incarcerated individuals. Qualifications will include knowledge of harm reduction principals, MOUD, OEND, and local services and treatment programs for referrals and connections.<br><br>Worker will ensure Narcan kits are given to inmates upon release (8:30am everyday). Worker will assist in activating inmates MA Health and secure appointments with outside service providers to bridge MOUD treatment post-release. Worker will be deputized as TPP concierge (see below) to have access to schedule rides for inmates being released directly to MOUD appointments in the community.	Group facilitator training<br><br>BAMSI and/or HCS to provide harm reduction training.<br><br>Praxis Narcan Train the Trainer<br><br>Facilitation training	Fulltime Salaried Position for 18 months: Payroll/Fringe $74,344.31<br><br>Mileage $855.00<br><br>Phone/Computer/Supplies/PPE $1,800<br><br>Total Direct Cost: $76,999.31<br><br>Admin Fee: $7,699.31<br><br>**TOTAL COST: $84,699.24**

Action Plan Review Form

Purpose: To assess action plan adherence to the ORCCA menu, impact, inclusion of high-risk populations/venues, feasibility and resources.

Description: Community-specific plans approved by the coalition memberships were then reviewed by HCS MA Leadership and State partners from the department of public health using a standard review form assessing: ORCCA adherence, Impact, Inclusion of High-Risk Populations/Venues, Feasibility and Resources. Written feedback was provided to community teams who then discussed feedback with their coalitions. Leadership met with community teams and coalitions as needed to support with plan clarification or revision. Revised plans were resubmitted for approval by HCS MA Core leaders and state partners.

Who completes the tool: HCS MA Leadership and State partners from the department of public health.

Community:

Please comment below about the extent to which the plan meets the designated criteria, including strengths, challenges and modifications.

Approval Criteria	OEND Plan	MOUD Plan	Safer Prescribing Plan
**ORCCA Adherence**<br>☐ Within the scope of the ORCCA menu<br>☐ Aligned with the overall goal of HCS			
**Impact**<br>☐ Extent to which plan will:<br>o Increase # POUD on MOUD<br>o Retain POUD in care<br>o Increase OEND to POUD at highest risk<br>o Address gaps in the continuum of care<br>o Be sustainable			
**High-Risk Population/Venues**<br>☐ Extent to which plan will:<br>o Include efforts to engage with people who have been historically marginalized<br>o Prioritize populations at heightened risk for overdose - BH/CJ/HC (i.e. prior overdose, decreased tolerance, multi-substance use, concomitant medical or mental illness, homeless, non-English speakers, minority populations)<br>o Mitigate and not exacerbate underlying racial inequities<br>o Prevent unintended consequences			
**Feasibility**<br>☐ Extent to which plan is:<br>o Feasible to implement by January 1, 2021<br>o Clear on what will be done by who and by when to bring about change<br>☐ Extent to which structural or regulatory challenges act as a barrier for this plan			
**Resources**<br>☐ Plan is within budget			

Appendix 3. New York Action Planning Tools

Community Evidence Based Practice Goals

Purpose: To define community level goals for EBPs

Description: The starting point for action planning is reflection and discussion by the coalition on the Community Profile, Data Dashboard, and locally sourced data summarized in a Discussion Tool from Phase 3. This is followed by development of community goals for each ORCCA EBP. Coalitions were asked to reflect on the success of COVID-19 influenced fast tracked strategies from earlier phases and to consider expansion and sustainability of these strategies, as well as to use a health and racial equity lens in the development of community Specific, Measurable, Attainable, Realistic and Time-framed (SMART) goals for each EBP.

Who completes the tool: The community-based implementation team captures discussions and decisions from Coalition members starting in workgroups with their champions by ORCCA Menu and further facilitated in the larger coalition.

DIRECTIONS: Setting ORCCA Goals
The Implementation Team facilitates a discussion with the Coalition (Steering Committee) to identify ORCCA goals based on the recommendations of the ORCCA, Communication and Data Workgroups.

These will be community-level goals to provide OEND in high risk populations, provide outreach and delivery of MOUD to high populations, and provide for safer opioid prescribing and dispensing.

ORCCA Goals

Providing OEND in High Risk Populations	Ex. In 12 months expand OEND through four strategies: expanding peer navigation, expand virtual training, using a variety of distribution strategies, and expanding the number, integration and reach of community trainers by 50%.
Outreach and Delivery of MOUD to High-Risk Populations	Ex. In 9 months, expand access to MOUD by increasing the number of waivered MOUD prescribers and treatment slots; improving prescribing rates of current waivered prescribers; create and sustain a prescriber advisory board to support best practice; and enhancing care coordination, outreach and linkage to enhance engagement and retention in care. Use a data feedback system to improve coordination across providers and service systems and address SDH through a targeted response to increase MOUD retention.
Safer Opioid Prescribing and Dispensing	Ex. Improve safer opioid prescribing and dispensing practices by engaging prescribers and pharmacies to provide resources on safer prescribing, tapering practices, alternative pain management and promoting safer opioid disposal practices between 6-12 months from the time of implementation.

Action Planning Worksheet

Purpose: To identify EBP strategies, venues and priority populations in an iterative process using community profiles, and data identifying needs, gaps and opportunities

Description: Workgroups brainstormed possible ORCCA EBP strategies and communication messages following training and practice using the ORCCA and technical assistance guide (TAG) that could fill existing gaps with identified populations, sectors and venues discussed in Phase 3. ORCCA EBP strategies were considered that could strengthen ongoing community efforts, create opportunities for expansion and efficiency, and promote synergistic approaches to new and existing community strategies. These strategies were presented to the coalition for consideration.

Who completes the tool: Coalition members, workgroups led by champions from each menu and facilitated by community implementation teams

Table 1: Discussion Tool for STEP 1 (CTH PHASE 4)

Strategy	Description & Approach to Population Access (High Risk/Venue)<br>Briefly describe each specific strategy being considered.<br>For strategy, identify populations of focus and venues for reaching them.		STRATEGY<br>Label each strategy:<br>Scale up<br>New Population<br>New Feature<br>New Service<br>New Service & Population	Pros and Cons of each Strategy
	**High Risk Populations**<br>Prior Opioid Overdose<br>Reduced Opioid Tolerance<br>Other Substance Use<br>Co-occurring mental health<br>Co-occurring physical health<br>Injection Drugs	**Venues**<br>Harm Reduction/Syringe Service<br>Health Care<br>Criminal Justice<br>First Responder<br>Addiction Treatment & Recovery<br>Behavioral Health Treatment<br>Community Based -Social Service Hotline		
**OEND in High Risk Populations**				
Active OEND (required)	*EX: Distribute naloxone to released inmates at County Jail or other setting*		EX: scale up or new service	
Passive OEND (optional)	*EX: Distribute naloxone at pharmacy or dispensing program*		EX: New feature	
Naloxone administration strategies (optional)	*EX: First responder naloxone administration*		EX: scale up	
**Outreach and Delivery of MOUD to High-Risk Populations**				
Bridging MOUD (required)	*EX: bridge program for people returning to community from county jail*		EX: new service	
Linkage using Peer Navigator (required)	*EX: Linkage to peer navigation for patients who overdose in ED*		EX: new feature	
Engage and retain individuals in treatment (required)	*EX: telemedicine, virtual appointment*		EX: new feature	
**Safer Opioid Prescribing and Dispensing**				
Safer opioid prescribing/dispensing practices (required)	*EX: Emergency/urgent care*		EX: scaling up	
Safer opioid disposal practices (optional)	*EX: • Prescription drug drop-box/mail-back programs*		EX: scale up or new service	

An additional tool used to make decisions about strategies and their potential for synergy and impact, was agent-based and systems dynamic modeling. Feedback reports using this modeling were introduced to County Implementation Teams and workgroups alongside community profiles to highlight the importance of OEND and MOUD expansion specifically, in order to meet the intervention's opioid overdose prevention goals (see Figure 3), and to inform the prioritization, adaptation, maintenance and sustainability of effective interventions over time for action planning. The systems perspective is intended to be integrated into problem-solving and feedback during ongoing implementation to improve coordination and resource sharing within community networks.

Figure 3-NY. Systems Feedback Reports for Planning

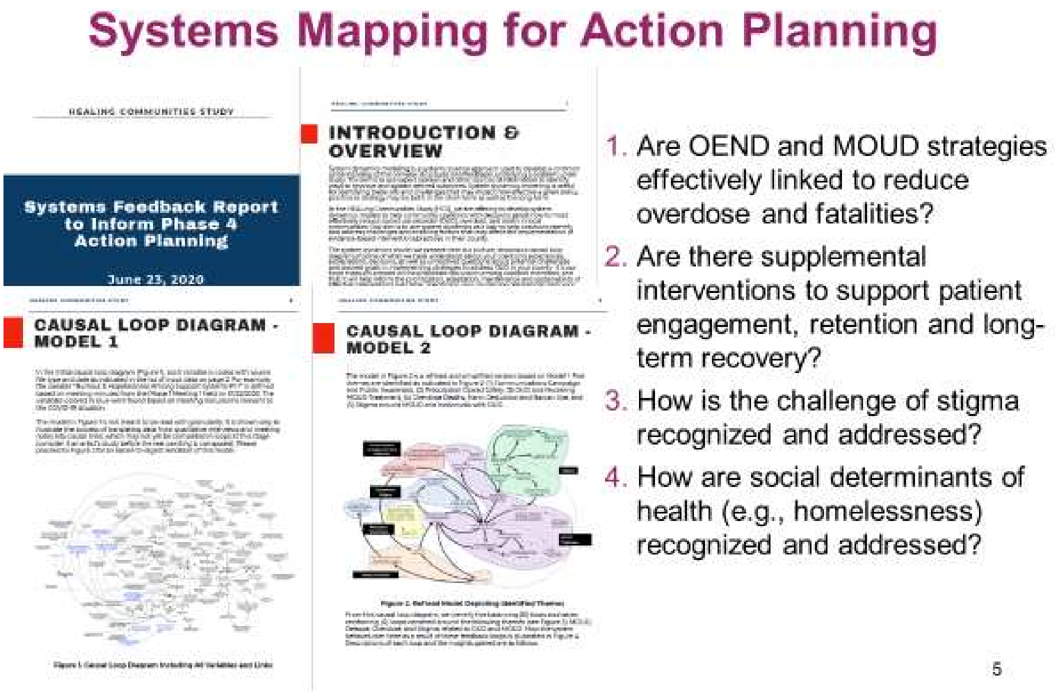

Consultation and Coaching Plan

Purpose: To provide coaching and feedback to assure adherence to HCS required EBP selections, and enhance capacity of local teams for community planning

Description: The research study team, including implementation coaches assigned to each community, offered iterative support as Community Implementation Teams and workgroups ranked strategy options based on earlier discussion of impact and feasibility determinations in workgroups, finalized strategy selections, and refined strategies into specific SMART goals. The ranking and strategy selection process was not uniform across coalitions. While no limits were placed on the number of strategies selected, attention was given to ensure the 5 required strategies defined in the ORCCA were present or a justification for why not were included. Opportunities were highlighted during coaching sessions to reinforce data driven justification for strategy selection and to feature the role of EBP workgroup champions in leading planning.

Who completes the tool: The Study Team reviews the Action Planning Worksheet with Summary Action Plans with the Implementation Team in coaching sessions using the criteria below:

ORCCA Goals	Prioritized EBPs	Draft CAP	Adopted CAP
Goals are aligned with HCS goal to reduce opioid overdose mortality by 40%	Priorities are aligned with the ORCCA-specific goals	CAP summarizes coalition goals	Summarizes coalition goals
Goals target identified gaps in the provision of OEND, MOUD, and safer opioid prescribing and dispensing	Priorities address the gaps documented in Community Profile	CAP summarizes selected strategies	Summarizes selected strategies, venues and populations
	Workgroup prioritized strategies as high impact	CAP identifies the team responsible for implementing the strategies	Identifies the team responsible for implementation planning and organizations for initial engagement
	Workgroup prioritized strategies as high feasibility	CAP identifies and the timeframe for implementation planning	Identifies the timeframe for implementation planning
		To what degree have considered health equity and racial disparities?	Have considered health equity and racial disparities
		Initial TA and resources needed, including plans to use Community Impact (CI) Funds	TA and resources needed, including plans to use CI Funds

SCHEDULING REVIEW AND CONSULTATION

Implementation Teams should upload and maintain documentation for each deliverable (ORCCA Goals, prioritized EBPs and the Community Action Plan) on the Public HCS County Folders drive. Project Managers should notify the Study Team by email to request review and coaching sessions. Please use the following timetable:

Deliverable	When to Request Review	Review Type	Target Deadline	Response Time
**ORCCA Goals**	Workgroup and Coalition consensus on ORCCA goals	Review and comment	**00/00/0000**	2 Business Days
**Prioritized EBPs Draft Community Action Plan**	Prioritized EPBs and draft Community Action Plan is ready for recommendation the Coalition	Coaching Meeting	At least 1 week prior to Coalition Meeting at which EBPs and AP will be considered	2 Business Days
**MOUD and Safer Rx EBP, AP**	Consulting addiction psychiatrists will also provide consultation and review of the prioritized EBPs and draft Community Action Plan for MOUD and Safer Prescribing Workgroups during Workgroup meetings.			
**Approved Community Action Plan**	Coalition has approved the Community Action Plan	Review and comment	Next business day after Coalition approval	2 Business Days

The Implementation Teams can invite the Study Team to join the Workgroup meeting in which EBP strategies are being prioritized to provide review and consultation, if that is the preference of the Workgroup, Champion and Implementation Team.

Summary Community Action Plan Form

Purpose: To provide a summary of EBPs and community goals, venues and priority populations with initial organizations, and first steps to be the focus for implementation planning in Phase 5.

Description: The final step in the Phase 4 Action Planning Worksheet is a summary community action plan for final coalition approval guided by the coalition's charter on decision-making. Most used a majority vote in a coalition meeting. The plan includes defined SMART goals with an outcome target for each ORCCA EBP-related strategy and identification of specific populations, sector for implementation (i.e., behavioral health, criminal justice, healthcare), reference to feasibility and impact rankings from workgroup discussions, and preliminary sustainability. Additionally, in anticipation of Phase 5 Implementation Plan Agreement development, the action plan includes recommended initial and anticipated steps for engaging community partners and estimates of technical assistance and resources.

Who completes the tool: Community implementation teams led by a program manager and CEF complete the summary and present to the coalition for adoption. In some cases a county executive or leadership team provides an additional level of approval.

TABLE 6: Action Plan Table (CTH PHASE 4)

Strategy Category	Selected Strategies	Targeted Populations	High-Risk Sector			Sustainability Feasibility/ Impact	Implementation Steps: who does what, by when and where	Technical Assistance & Other Resources
			BH	CJ	HC			
**ORCCA Objective 1: Increase Opioid Overdose Prevention Education and Naloxone Distribution**								
Active OEND (required)	A1:<br>A2:							
Passive OEND (optional)	B1:<br>B2:							
Naloxone administration (optional)	C1:<br>C2:							
**ORCCA Objective 2: Outreach and Delivery of MOUD to High-Risk Populations**								
Expand MOUD treatment (required)	D1:<br>D2:							
Linkage to MOUD (required)	E1:<br>E2:							
Engagement and retention (required)	F1:<br>F2:							
**ORCCA Objective 3: Improve Prescription Opioid Safety**								
Safer Prescribing/ Dispensing (required)	G1:<br>G2:							
Safer Disposal Practices (optional)	H1:<br>H2:							

Appendix 4. Ohio Action Planning Tools

Decision Aid 1 Matrix

Purpose: To identify existing venues related to ORCCA EBPs

Description: In Ohio, the HCS Intervention Facilitator, one field team (i.e., an HCS-hired Intervention Facilitator, Community Engagement Facilitator, Data Manager) worked with community coalitions, particularly coalition-identified EBP champions to utilize a multi-step Decision Aid Approach across Phase 4 study activities. The first step was to identify existing venues related to ORCCA EBPs. In Step 1: Cataloging Possible Approaches to Population Access, a matrix was used in Decision Aid 1 to map existing locations/methods of current *or potential* population access to priority populations. This allowed communities to inventory potential points of access and also highlighted options that were not available or are already highly represented.

Who completes the tool: Decision Aid 1 is completed by ORCCA EBP coalition champions in collaboration with HCS-hired staff.

**Community "A": Decision Aid 1**		**Points of Population Access**		
		**CJ**	**SSPs**	**EDs**
**Priority Populations**	**Prior Opioid Overdose**	ABC drug court<br>XYZ County Jail	Abc street exchange	None
	**Reduced Opioid Tolerance**	ABC drug court<br>XYZ County Jail		None
	**Use Other Substances**	ABC drug court<br>XYZ County Jail	Abc street exchange	None

Decision Aid 2

Purpose: To identify preliminary ORCCA-related goals for each of the three EBPs.

Description: In Step 2: Existing Service Inventory Mapped by Priority Population and Point of Population Access, a similar approach to Decision Aid 1 was employed, but limited to listing *currently available* services (i.e., interventions) specific to each of the three EBPs required by ORCCA. As a first step, a community would use their population access Decision Aid 1 from Step 1 to highlight any setting in which there was a current intervention related to the three ORCCA required objectives. Next, each community would catalog any existing services that address the three ORCCA required objectives using Decision Aids 2A (OEND), 2B (MOUD), and 2C (Safer Prescribing). This approach documents the gaps between potential methods for population access and the degree to which those were already being implemented.

Who completes the tool: Decision Aid 2 is completed by ORCCA EBP coalition champions in collaboration with HCS-hired staff.

**Community "A"- Decision Aid 2B: MOUD**		**Points of Population Access**		
		**CJ**	**SSPs**	**EDs**
**Priority Populations**	**Prior Opioid Overdose**	None	Abc Syringe Exchange-treatment referral	None
	**Reduced Opioid Tolerance**	None	None	None
	**Use Other Substances**	None	Abc Syringe Exchange-treatment referral	None

Decision Aid 3

Purpose: To identify, prioritize, and select EBP strategies.

Description: In Step 3 of the Decision Aid process, communities in collaboration with HCS *brainstormed potential intervention strategy and population access dyads.* This approach allowed for a shared understanding of "what was currently being done" and "where it was being done" in an efficient manner that mapped structurally to ORCCA priority populations and methods of population access. The Decision Aid was not menu-specific, but rather covered strategies for intervention across menus. This provided the foundation for brainstorming which ORCCA EBP strategies *might target existing gaps.* In this step, there is an emphasis on feasibility of implementation.

Discussion Aid 3 was employed in Step 3 and prompted communities to consider whether the identified options involved:

a. bringing an existing service to scale ("1")
b. disseminate an existing service to a new population or method of population access ("2")
c. add a new feature or improvement to an existing service (i.e., changing passive referral to formal linkage intervention); ("3")
d. add a new service; ("4")
e. add both a new method of population access and a new service (e.g., a syringe service program with naloxone distribution); ("5").

Who completes the tool: Decision Aid 3 is completed by ORCCA EBP coalition champions in collaboration with HCS-hired staff.

**Community A-Decision Aid 3: Possible Strategies for Intervention—*no priority order***		**Approach to Population Access**	**Category of Implementation (1-5 above)**
**1**	Distribute OEND	XYZ County Jail	1: bring to scale
**2**	Bridging MOUD	XYZ County Jail	4: new program
**3**	Linkage to Care-Mandated Treatment	ABC Drug Court	1: bring to scale
**4**	Linkage using Peer Navigator	XYZ County Jail	4: new program
**5**	Linkage using Peer Navigator	SSP	3/4: modifying referral program/new program

Decision Aid 4

Purpose: To prioritize EBP strategies by feasibility and expected impact.

Description: This step involved consideration of the intersection between the importance of a given plan and the degree of difficulty in implementing that plan with the notion that EBPs that were both *highly impactful* and *highly feasible* should be undertaken before other actions that are of lower impact and/or lower feasibility. In Decision Aid 4, the identified strategies were categorized by priority/importance (low, medium, high) and by feasibility/capacity (low, medium, high)

Who completes the tool: Decision Aid 4 is completed by ORCCA EBP coalition champions in collaboration with HCS-hired staff.

**Community A- Decision Aid 4: Possible Strategies for Intervention—*feasibility/priority***		**Feasibility/Capacity**		
		Low	Medium	High
**Priority/Importance**	Low	• Linkage to Care Mandated Treatment at ABC Drug Court (*i.e., already at scale with little opportunity to expand*)	• Linkage using peer-navigators at XYZ county Jail (i.e. may move to high priority if bridging MOUD is unsuccessful)	
	Medium		• Distribute OEND at XYZ county jail	
	High	• Bridging MOUD at XYZ county jail	•	• Linkage using peer-navigators at SSP

Decision Aid 5

Purpose: To prioritize and select EBP strategies.

Description: In Step 5: Prioritizing and Selecting EBPs, communities ranked the options outlined in Step 3 in priority order based on the feasibility and impact determinations of Step 4. Coalitions were encouraged to prioritize options that were high impact and high feasibility over interventions that were of low impact or feasibility. Of note, the coalition was not limited in the number of strategies they could target for implementation. This determination was encouraged to be paired with the decision on whether the community and HCS team would pursue a given option for implementation.

Utilizing Step 5 of the Decision Aid process, communities then create their initial action plan specific to their ORCCA goals and the required ORCCA EBPs. The action plan includes the specific strategies selected, the targeted sectors for the intervention, the identified practice settings or venues, preliminary action plans, potential technical assistance or other resource needs, the individual within the Ohio Intervention Design Team (IDT), comprised of academic and subject experts, who will be primarily overseeing and assisting with the implementation of the identified strategy, and additional notes regarding the strategy. For example, if an EBP were to be conducted in an Emergency Department setting, the Emergency Department IDT would oversee the implementation of that EBP and provision of technical assistance.

Who completes the tool: Decision Aid 5 and the Action Plan is completed by ORCCA EBP coalition champions and community coalitions in collaboration with HCS-hired staff.

**Community A-Decision Aid 5: Selected Strategies for Intervention—*priority order***		**Approach to Population Access**	**Target for Action**	**Notes/Preliminary Action Plan**
**1**	Linkage using Peer Navigator	SSP	Yes	Hire program coordinator; select peers
**2**	Bridging MOUD	XYZ County Jail	Yes	
**3**	Distribute OEND	XYZ County Jail	Yes	
**3**	Linkage to Care-Mandated Treatment	ABC Drug Court	No	
**4**	Linkage using Peer Navigator	XYZ County Jail	No	Revisit in 3 months pending exploration of bridging MOUD at XYZ County Jail

Action Plan Template and Sample Completed Action Plan

County: ________________________

Completed By: ___ ______
Initial Completion Date: _____/____/______
Revision #: ______________
Revision Completion Date: _____/________/________

	Specific Strategy	Approach to Population Access (Sector)	Practice Setting or Venue Name	Preliminary Action Plan	Potential TA/Resource Needs	State IDT Lead	Notes
**ORCCA Objective 1: Increase Opioid Overdose Prevention Education and Naloxone Distribution**							
Active OEND (Required)							
Passive OEND (optional)							
**ORCCA Objective 2: Outreach and Delivery of MOUD to High-Risk Populations**							
Expand MOUD treatment (required)							
Linkage to MOUD (required)							
Engagement and retention (required)							
**ORCCA Objective 3: Improve Prescription Opioid Safety**							
Safer Prescribing/ Dispensing (required)							
Safer Disposal Practices (optional)							

Strategy category	Specific Strategy	Approach to Population Access (Sector)	Practice Setting or Venue Name	Preliminary Action Plan	Potential TA/Resource Needs	State IDT Lead	Notes
**ORCCA Objective 1: Increase Opioid Overdose Prevention Education and Naloxone Distribution**							
**Active OEND (required)**	**Active OEND for at-risk individuals and their social networks**<br><br>*Expansion of Project DAWN (a program to provide naloxone to Ohio agencies) to a secondary site*	Behavioral Health	Church	Confirm partner agencies. (HCS)<br><br>Assemble Intervention Design Team/Operations Team to provide implementation assistance. (HCS)	Resources needed on technology/ data collection, billing for sustainability.	[NAME]	Date approved by coalition: DATE
**Active OEND (required)**	**Active OEND for at-risk individuals and their social networks**<br><br>*Expansion of Project DAWN to a secondary site*	Healthcare	[AGENCY] Mobile Unit	Confirm partner agencies. (HCS)<br><br>Assemble Intervention Design Team/Operations Team to provide implementation assistance. (HCS)	Resources needed on technology/ data collection, billing for sustainability.	[NAME]	Date approved by coalition: DATE
**Active OEND (required)**	**Active OEND at high-risk venues:**<br><br>*Naloxone Referral/ Distribution for Drug Court Participants*	Criminal Justice	Drug Court	Confirm partner agencies. (HCS)<br><br>Assemble Intervention Design Team/Operations Team to provide implementation assistance. (HCS)	Potential resources needed on increasing access and participant engagement.	[NAME]	Date approved by coalition: DATE
**Active OEND (required)**	**Active OEND at high-risk venues: “Leave behind”**	Healthcare	Fire/EMS	Confirm partner agencies. (HCS)<br><br>Assemble Intervention Design Team/Operations	Potential resources needed on policy/ implementation logistics, streamlined data collection for naloxone use.	[NAME]	Date approved by

Strategy category	Specific Strategy	Approach to Population Access (Sector)	Practice Setting or Venue Name	Preliminary Action Plan	Potential TA/Resource Needs	State IDT Lead	Notes
	**programs at sites of overdose**<br><br>*Fire/EMS Leave-Behind Program*			Team to provide implementation assistance. (HCS)			coalition: DATE
**OEND self-request (e.g. at pharmacy, community meeting or public health department)**	**Passive OEND**<br><br>Naloxone Distribution at Pharmacies in high risk areas	Healthcare	Pharmacy	Confirm partner agencies. (HCS)<br><br>Assemble Intervention Design Team/Operations Team to provide implementation assistance. (HCS)	Potential resources needed on logistics and possible policy development.	[NAME]	Date approved by coalition: DATE
**ORCCA Objective 2: Outreach and Delivery of MOUD to High-Risk Populations**							
**Expand MOUD treatment (required)**	**Adding/expanding MOUD treatment in Criminal Justice settings**<br><br>*MOUD in the Jail*	Criminal Justice	Jail	Confirm partner agencies. (HCS)<br><br>Assemble Intervention Design Team/Operations Team to provide implementation assistance. (HCS)	Potential resources needed on logistics, billing, etc.	[NAME]	Date approved by coalition: DATE
**Linkage to MOUD**<br><br>**(required)**	**Linkage Programs (all relevant settings)**<br><br>*Expand MOUD referral in ED*	Healthcare	Emergency Department	Confirm partner agencies. (HCS)<br><br>Identify opportunities for expansion of current services.<br><br>Assemble Intervention Design Team/Operations Team to provide implementation assistance. (HCS)	Potential resources needed on sustainability and patient engagement.	[NAME]	Date approved by coalition: DATE

Strategy category	Specific Strategy	Approach to Population Access (Sector)	Practice Setting or Venue Name	Preliminary Action Plan	Potential TA/Resource Needs	State IDT Lead	Notes
**Engagement and retention (required)**	**Reducing barriers to housing, transportation, childcare and accessing other community benefits for people with OUD**<br><br>*Reduce barriers to transportation to improve engagement/retention in treatment*	Behavioral Health	MOUD Treatment Facility<br><br>Potentially other MOUD treatment facilities with limited transportation options. (TBD)	Confirm partner agencies. (HCS)<br><br>Assemble Intervention Design Team/Operations Team to provide implementation assistance. (HCS)<br><br>Engage partners and IDT through an initial planning meeting. (HCS)	Potential resources needed on billing for sustainability.<br><br>Needs: Identify potential partnerships between XXX transportation and treatment agencies	[NAME]	Date approved by coalition: DATE
**ORCCA Objective 3: Improve Prescription Opioid Safety**							
**Safer Prescribing/ Dispensing (required)**	**Safer opioid prescribing for chronic pain**<br><br>Prescriber Accountability through OD Review	Healthcare	Pharmacy	Confirm partner agencies. (HCS)<br><br>Assemble Intervention Design Team/Operations Team to provide implementation assistance. (HCS)<br><br>Engage partners and IDT through an initial planning meeting. (HCS)	Potential resources needed on logistics and identifying allowable information to be shared between the XXX team and XXX Agency.	[NAME]	Date approved by coalition: DATE
**Safer Prescribing/ Dispensing (required)**	**Safer opioid dispensing**<br><br>Utilize the Take Charge Ohio Toolkit for pharmacist and patient education. (Co-dispensing naloxone, providing educational materials	Healthcare	Pharmacy	Engage Education and Awareness workgroup of Scioto County Collaborative Opioid Consortium.<br><br>Determine which parts of the Toolkit will be utilized.<br><br>Confirm partner agencies. (HCS)	Potential resources needed on logistics and possible policy development.	[NAME]	Date approved by coalition: DATE

Strategy category	Specific Strategy	Approach to Population Access (Sector)	Practice Setting or Venue Name	Preliminary Action Plan	Potential TA/Resource Needs	State IDT Lead	Notes
	on opioids and naloxone)			Assemble Intervention Design Team/Operations Team to provide implementation assistance. (HCS)			
