# Supplementary material for: Protocol for community-driven selection of strategies to implement evidence-based practices to reduce opioid overdoses in the HEALing Communities Study: a trial to evaluate a community-engaged intervention in Kentucky, Massachusetts, New York and Ohio
Source: BMJ Open. 2022 Sep 19;12(9):e059328. doi: 10.1136/bmjopen-2021-059328 (PMC9486330; doi:10.1136/bmjopen-2021-059328)
Supplement: Author's manuscript [file bmjopen-2021-059328.draft_revisions.pdf]

BMJ Open is committed to open peer review. As part of this commitment we make the peer review history of every article we publish publicly available.

When an article is published we post the peer reviewers' comments and the authors' responses online. We also post the versions of the paper that were used during peer review. These are the versions that the peer review comments apply to.

The versions of the paper that follow are the versions that were submitted during the peer review process. They are not the versions of record or the final published versions. They should not be cited or distributed as the published version of this manuscript.

BMJ Open is an open access journal and the full, final, typeset and author-corrected version of record of the manuscript is available on our site with no access controls, subscription charges or pay-per-view fees (http://bmjopen.bmj.com).

If you have any questions on BMJ Open's open peer review process please email info.bmjopen@bmj.com

BMJ Open

Addressing the opioid overdose epidemic: A protocol for community-driven selection of strategies to implement evidence-based practices in the HEALing Communities Study, a multi-site, parallel-group, cluster randomized wait-list controlled trial

Journal:	*BMJ Open*
Manuscript ID	bmjopen-2021-059328
Article Type:	Protocol
Date Submitted by the Author:	13-Dec-2021

Complete List of Authors:	Young, April; University of Kentucky, Epidemiology<br>Brown, Jennifer; University of Cincinnati<br>Hunt, Timothy; Columbia University<br>Sprague Martinez, Linda; Boston University<br>Chandler, Redonna; National Institutes of Health<br>Oga, Emmanuel; Research Triangle Institute<br>Winhusen, Theresa ; University of Cincinnati Medical Center, Psychiatry<br>Baker, Trevor; Boston Medical Center<br>Battaglia, Tracy; Boston Medical Center<br>Bowers-Sword, Rachel; Boston Medical Center<br>Button, Amy; Albert Einstein College of Medicine, Montefiore Hudson Valley Collaborative<br>Fallin-Bennett, Amanda ; University of Kentucky, College of Nursing<br>Fanucchi, Laura; University of Kentucky<br>Freeman, Patricia ; University of Kentucky College of Pharmacy<br>Glasgow, LaShawn; Research Triangle Institute<br>Gulley, Jennifer; Clark County Health Department<br>Kendell, Charles; Franklin County Agency for Substance Abuse Policy Board<br>Lofwall , Michelle ; University of Kentucky, Center on Drug and Alcohol Research<br>Lyons, Michael S.; Cincinnati Children's Hospital Medical Center<br>Quinn, Maria; Holyoke Medical Center<br>Rapkin, Bruce; Albert Einstein College of Medicine, Epiemiology and Population Health<br>Surratt, Hilary; University of Kentucky<br>Walsh, Sharon ; University of Kentucky

Keywords:	Substance misuse < PSYCHIATRY, PUBLIC HEALTH, STATISTICS & RESEARCH METHODS

SCHOLARONE™
Manuscripts

Addressing the opioid overdose epidemic: A protocol for community-driven selection of strategies to implement evidence-based practices in the HEALing Communities Study, a multi-site, parallel-group, cluster randomized wait-list controlled trial

Young AM, Brown JL, Hunt T, Sprague Martinez LS, Chandler R, Oga E, Winhusen TJ, Baker T, Battaglia TA, Bowers-Sword R, Button A, Fallin-Bennett A, Fanucchi L, Freeman PR, Glasgow LM, Gulley J, Kendell C, Lofwall MR, Lyons MS, Quinn M, Rapkin B, Surratt H, Walsh S.L.

Corresponding Lead Author: April M Young (april.young@uky.edu)

For peer review only

Abstract

Introduction: Opioid-involved overdose deaths continue to surge in many communities, despite numerous evidence-based practices (EBPs) that exist to prevent them. The HEALing Communities Study (HCS) was launched to develop and test an intervention (i.e., Communities That HEAL [CTH]) that supports communities in expanding uptake of EBPs to reduce opioid-involved overdose deaths. This paper describes a protocol for a process foundational to the CTH intervention through which community coalitions select strategies to implement EBPs locally.

Methods and analysis: The CTH is being implemented in 34 communities (randomized to receive the intervention) in four states in partnership with coalitions (one per community). Coalitions must select at least five strategies, including one to implement each of the following EBPs: (a) overdose education and naloxone distribution; expanded (b) access to medications for opioid use disorder [MOUD], (c) linkage to MOUD, (d) retention in MOUD; and (e) safer opioid prescribing/dispensing. Facilitated by decision aid tools, the community action planning process includes (1) data-driven goal setting, (2) discussion and prioritization of EBP strategies, (3) selection of EBP strategies, and (4) identification of next steps. Following review of epidemiologic data and information on existing local services, coalitions set goals, and discuss, score, and/or rank EBP strategies based on feasibility, appropriateness within the community context, and potential impact on reducing opioid-involved overdose deaths with a focus on three key sectors (health care, behavioral health and criminal justice) and high-risk/vulnerable populations. Coalitions then select EBP strategies through consensus or majority vote and, subsequently, suggest or choose agencies with which to partner for implementation.

Ethics and dissemination. The HCS protocol was approved by a central IRB (Advarra, Inc) and is registered with Clinical Trials.gov (NCT04111939). Results of the action planning process will be disseminated in academic conferences and peer-reviewed journals, online and print media, and in meetings with community stakeholders.

For peer review only

Strengths and limitations of this study

- A strength of the CTH action planning process is that it balances scientific rigor with community priorities by ensuring all communities follow a common core of structured activities and phased benchmarks, select from a common menu of strategies for implementing EBPs, and discuss similar considerations as they create their action plan, while also allowing variation across states and communities to best suit the local context.
- The CTH intervention elevates the voices of community members, whose insights about the local community context are critical to implementation success and whose involvement conveys to implementing agencies and local decision-makers that EBP implementation is a priority of the local community and constituents rather than of an outside (federal or academic) entity.
- Careful facilitation of the action planning process by coalition members and community staff is required to ensure that power dynamics do not result in some coalition members having more influence than others in the decision-making process and to manage conflicts of interest (i.e., selection of strategies that could benefit or be perceived to benefit individual coalition members or their agencies).
- Although the phased data-driven decision-making is critical for selection of strategies appropriate to a community's context, the phased and iterative action planning process is time-intensive requiring significant commitment from involved community members, faculty, and staff, and may slow implementation of EBPs.
- While necessitated by the constraints of the study timeline and novelty and complexity of the CTH intervention, the research teams' intensive involvement in facilitating action planning may have stifled participation by some coalition members who approached the process with deference and thereby limited the extent to which it was community-driven.

For peer review only

Introduction

Opioid-involved overdose deaths remain a public health crisis in many communities worldwide[1 2] although numerous evidence-based practices (EBPs) exist to prevent them. In the Unites States alone, opioid-involved overdose claimed over 490,000 lives between 1999 and 2019.[3] Despite national efforts to change the trajectory of overdose deaths, provisional data indicate over 94,000 total overdose deaths occurred in the 12 months ending in January 2021, making this the highest number of overdose deaths ever recorded in a 12-month period.[4]

Research has demonstrated that medication for opioid use disorder (MOUD), including methadone, buprenorphine, and extended-release naltrexone;[(e.g. 5)] prescription drug monitoring programs and safer prescribing guidelines for prescription opioids;[(e.g. 6)] naloxone, a medication that can reverse an opioid overdose;[(e.g.,7)] and behavioral/psychosocial interventions like recovery support services [(e.g. 5)] can prevent deaths and help people enter and stay in remission and recovery from opioid use disorder. Unfortunately, these EBPs remain under-utilized in many communities leaving significant unmet need for these critically important services.[8-15]

The HEALing Communities Study (HCS), supported by the National Institutes of Health and the Substance Abuse and Mental Health Services Administration, launched in 2019 to develop and test an approach to support communities in expanding uptake and availability of EBPs to address opioid-involved overdose deaths and opioid use disorder (OUD). HCS is a multi-site, parallel-group, cluster randomized wait-list controlled trial involving 67 communities (34 in active intervention and 33 in wait-list control) in Kentucky (KY), Massachusetts (MA), New York (NY), and Ohio (OH).[16] The study is designed to test the Communities That HEAL (CTH) intervention, which contains three components: a community-engaged process of data-driven decision making to select EBP strategies for local implementation and monitoring;[17] a

For peer review only

menu of EBPs and technical assistance guides (Opioid-Overdose Reduction Continuum of Care Approach [ORCCA]),[18] and a set of communication campaigns intended to address stigma and increase knowledge of and demand for EBPs.[19] The CTH intervention is being implemented in the active intervention communities from January 2020 to June 2022 and will be implemented in wait-list control communities from July 2022 to June 2023.

The CTH intervention is grounded in the recognition that communities affected by the opioid overdose crisis possess the best understanding of local strengths, gaps, barriers and facilitators related to local EBP implementation and uptake.[17] Thus, the CTH involves partnerships between academic researchers and community members through community coalitions. Coalitions are comprised of representatives from diverse agencies and institutions as well as individuals focused on tackling complex community issues through collective planning.[20] The CTH intervention employs a phased coalition planning process that was developed based on and expands the Communities that Care model,[17] which has supported communities in the selection of evidence-based prevention efforts in past research.[21-23] Coalition planning models are effective because they allow communities to leverage resources and expertise from across multiple sectors, which can facilitate creative problem solving and innovation, reduce duplication of efforts, and address service gaps.[24 25] The literature points to coalition planning as an effective strategy for implementing EBPs.[26-28]

The purpose of this paper is to describe the protocol for coalition action planning through which EBP strategies are selected to reduce opioid overdose deaths in their communities.[16] The protocol described in this manuscript is relevant beyond the field of substance use, as community engagement and community-driven decision making are recommended for improving responses to a variety of health challenges,[29-32] including, most recently, SARS-CoV-2 vaccination.[33]

For peer review only

Methods and Analysis

Participant involvement

As described elsewhere,[17] community engagement is central to the CTH intervention. The CTH intervention places communities, specifically community coalitions, at the center of the decision-making process in creating an action plan for implementing EBP strategies locally. The coalitions are comprised of key stakeholders in healthcare, behavioral health, criminal justice, public health, government, business, housing, and other sectors, and in many cases, include people with lived experience with or impacted by OUD. In some communities, the coalitions that are presently engaged in the CTH existed prior to the HCS, while others were formed specifically to guide the CTH intervention. In addition to driving the CTH intervention, these coalitions facilitate dissemination of information about the study and about EBPs to other local stakeholders. Coalitions, local agency partners, and partnerships formed between academic institutions and local stakeholders will also be key to sustainability of the CTH process and EBPs. Of note, coalitions were not involved in setting the research question, designing the intervention protocol or menu of EBPs, or establishing outcome measures. Of note, each of the four participating states has a Community Advisory Board, with multisectoral representation from across the communities that advises the research teams on study implementation.

Overview of CTH phases

The CTH intervention includes seven phases, described in detail elsewhere,[17] that guide coalitions through a systematic partnership process. Briefly, the process begins with

For peer review only

establishment or identification of a coalition and an environmental scan, or "landscape analysis," of local resources and gaps (Phase 0). During Phase 1, coalitions develop a shared charter and identify key community "champions" to support EBPs, data-driven decision making, and communication campaigns that have been described elsewhere.[19] In Phase 2, coalitions learn about data-driven decision making and the ORCCA menus containing EBP strategies related to overdose education and naloxone distribution (OEND, Menu 1), MOUD (Menu 2), and prescription opioid safety (Menu 3). The ORCCA menus have been described in detail elsewhere.[18] Following review of data describing the local epidemiology of OUD and overdose, simulation modeling data, and/or existing service resources and gaps identified by the landscape analysis (Phase 3), coalitions develop community action plans (one per coalition) by selecting strategies to implement EBPs that are in the ORCCA menu (Phase 4). After creation of the action plan, coalition selected EBP strategies are implemented and monitored (Phase 5) and sustainability plans are created (Phase 6). While these processes are ongoing, a health communication campaign is delivered at the community level to reduce stigma and increase engagement in OEND and MOUD.[19] This paper describes the protocol for coalition planning efforts in Phase 4 to select EBP strategies to reduce opioid overdose deaths locally.

Community Action Planning Guidelines

Coalitions are provided with guidelines to structure the community action planning process (Figure 1). Coalitions are required to select a minimum of five strategies, including at least one to implement each required EBP from the ORCCA: (a) expansion of proactive offering of OEND to at risk individuals and members of their social networks, (b) expansion of buprenorphine and/or methadone access, (c) improved linkage to MOUD, (d) increased retention

For peer review only - http://bmjopen.bmj.com/site/about/guidelines.xhtml

in MOUD, and (e) promotion of safer opioid prescribing/dispensing. The EBP strategies are required to include at least one to be implemented in each of the following three sectors: health care, behavioral health and criminal justice.[18] Coalitions are encouraged to consider EBP strategies that would maximize impact in key populations most vulnerable to experiencing an opioid-involved overdose, including those who had a prior opioid overdose;[8 34-37] have reduced opioid tolerance (e.g., completing medically supervised or socially managed withdrawal or release from institutional settings such as jail, residential treatment, hospital);[37-41] use other substances (e.g., alcohol, benzodiazepines, cocaine, and amphetamine like substances);[37 42-46] have concomitant major mental[47-50] or medical illness;[37 51-54] and/or inject drugs.[43 55] Coalitions are also asked to prioritize venues through which these key populations are most likely to be reached. Priority venues for each of the three EBPs (i.e., OEND, MOUD, and safer opioid prescribing and dispensing) are listed in Figure 1 and described in detail elsewhere.[18]

Inherently, the CTH allowed for communities to independently implement EBP strategies at their own pace, including expediting strategies in times of emergency. In June 2020, the HCS approved "fast tracking" certain HCS-supported activities related to OEND in response to the coalitions' concerns about the substantial uptick of opioid overdose observed in the initial months of the SARS-CoV-2 pandemic.[56-63] The protocol allowed HCS-supported naloxone distribution to begin in high-risk settings prior to the completion of action planning, but preserved the community-driven nature of the decision-making process. Coalitions were asked whether they wanted the HCS teams to provide technical assistance and/or resources to community venues with greatest reach to individuals at risk for overdose (i.e., jails, syringe service programs) to expand OEND. Upon coalition approval, HCS teams and/or community implementation teams began outreach to high-risk venues to expand OEND. Thus, the fast-

For peer review only

tracked OEND activities were ongoing during the Phase 4 action planning process. During the action planning process, coalitions were asked to reflect on the successes and challenges of the fast-tracked strategies and asked to decide whether they wanted to continue or expand OEND activities that had been fast-tracked as part of the action planning process.

Core components of the action planning process

The core components of the cross-state protocol for action planning are described in Figure 2. Action planning in each state involves (1) data-driven community goal setting, (2) discussion and prioritization of EBP strategies, (3) selection of EBP strategies to include in the action plan, and (4) identification of next steps for implementation of selected EBP strategies.

Coalitions' movement through the action planning process is facilitated by a combination of goal-setting tools and decision aids. Examples of tools developed by each state for the action planning process are provided in the Supplementary Appendix. The communities currently receiving the CTH intervention differ in geographic unit (i.e., counties, townships, cities), community-readiness for EBP strategy implementation, existing resources and gaps, coalition history (e.g., whether coalitions existed prior to the HCS or convened for primarily for the HCS), and other contextual factors thereby requiring some variation to the action planning process across and within states (Table 1).

Action planning in each state is driven by coalitions, coalition workgroups, and champions, and is facilitated by a team comprising clinical and research faculty, core staff, and community-based staff with expertise in community-engagement, data, OEND, MOUD, and safer opioid prescribing and dispensing. Coalition composition is described briefly in *Participant Involvement* and in detail elsewhere.[17] Of note, only in MA are *all* agencies to eventually be

For peer review only

involved in EBP strategy implementation represented on the coalition. In other states, the agencies to be involved in implementation are represented to varying degrees across coalitions. In NY, community government partners (i.e., health commissioners, county executives) are also involved in the action planning process.

Step 1. Develop Data-driven Community Goals

All coalitions begin the goal-setting process by reviewing community profiles and data dashboards, supplemented in one state (NY) by results of agent-based and systems dynamic modeling. The community profiles, developed through the CTH intervention, included lists of organizations in the community relevant to potential implementation of EBP strategies (e.g., substance use disorder treatment centers, harm reduction programs, emergency response agencies, hospitals). The data dashboards, also developed through the CTH intervention, contain data visualizations on community-specific trends in fatal and non-fatal opioid overdose, MOUD availability, naloxone distribution, and high-risk opioid prescribing.[9]

During and following data review, coalitions discuss strengths, services, gaps, and needs. The coalitions established goals varying in specificity across states and communities (e.g., “Increase OEND,” “Two emergency departments will initiate MOUD by January 2021”). Coalitions set goals at the ORCCA menu-level (i.e., for OEND, MOUD, and safer opioid prescribing/dispensing) or submenu-level (i.e., for active and passive OEND; for MOUD expansion, linkage, and retention; for safer opioid prescribing/dispensing and disposal). States vary in the other factors their coalitions are asked to consider when setting goals; these factors include sustainability of EBP strategies (NY, MA), EBP strategies’ reach to high-risk

For peer review only - http://bmjopen.bmj.com/site/about/guidelines.xhtml

populations (OH, MA), ability to ensure health equity across ethnic and racial groups (MA, NY), and priority venues for EBP strategy implementation (OH, MA).

Step 2. Discuss and Prioritize EBPs that Align with Community Goals

Following goal setting, coalitions consider specific strategies for implementing the EBPs included on the ORCCA menu and determine which are of highest priority for local implementation. In three states (OH, MA, NY), coalitions brainstorm and free-list EBP strategies that align with the study goals, priority sectors and/or venues, and high-risk populations. In KY, coalitions are provided with initial lists of EBP strategies to consider that are pre-populated from the ORCCA by the academic team. Coalitions in all states consider the EBP strategies in terms of the existing services, size of the gap between existing services and need, feasibility, and potential impact on opioid-involved overdose deaths. To frame the discussion of feasibility more concretely, coalitions in two states (OH, NY) are asked to consider whether the EBP strategy is a new service, scale-up of an existing service via a new method of service delivery, new target population, or new feature to an existing service. In two states (MA, NY), coalitions also consider sustainability. In all states, deliberations on the EBP strategies occur in full coalition meetings or in workgroups led by champions depending on the coalition's preference.

All coalitions begin by providing narrative feedback on each EBP strategy. One state's (KY) coalitions are asked to supplement the narrative feedback by scoring each EBP strategy in terms of size of the existing service gap, feasibility of implementation within the next 12 months, and potential impact on opioid-involved overdose deaths using Likert scales. The scale scores are used to produce a summative priority score to help guide EBP strategy selection. Two states (NY, OH) ask coalitions to rank EBP strategies in terms of impact and feasibility, with the most

For peer review only - http://bmjopen.bmj.com/site/about/guidelines.xhtml

impactful and feasible EBP strategies scoring highest. In one state (MA), coalitions prioritize EBP strategies through consensus building group dialogues as opposed to ranking.

In three states, coalitions are either required (MA) or encouraged to consider the cost of EBP strategy implementation (NY, OH). In MA, coalitions are provided with an overall budget cap to work within and as part of their planning are required to consider the cost of implementation as they make their EBP strategy selections. In NY, where communities receive funding for staffing and administrative costs and EBP strategy implementation based on population size, coalitions are encouraged, but not required, to consider the cost of EBP strategy implementation as they make their selections. In OH, all communities are provided with fixed and equivalent budgets with limits on expenditures for naloxone and technical assistance; coalitions consider these budget caps as they choose EBP strategies but are not asked to estimate the cost of individual EBP strategies. In KY, coalitions are encouraged *not* to consider cost, but rather to focus on potential need, impact, and feasibility of each EBP strategy.

<u>Step 3. Select EBP strategies to include in the community action plan</u>

In full coalition and coalition subgroup meetings, members review narrative notes, scores, and rankings from the process described above. In two states (KY, OH), coalitions are also provided with feedback from HCS faculty and staff on implementation feasibility with respect to structural or regulatory barriers. Coalitions weigh these considerations in the context of the guidelines described in Figure 1 and choose through consensus or majority vote the EBP strategies to be implemented with support of HCS. In making their initial selections, coalition members understand that they can later add and refine EBP strategies as capacity expands. Only one state (KY) set a maximum for the number of EBP strategies that could be selected initially,

For peer review only

not including OEND strategies already fast-tracked. Coalitions in KY are limited to twelve initial EBP strategies, requiring that they choose no more than three involving OEND, two for MOUD expansion, two for linkage to MOUD, two to improve MOUD retention, and three focused on safer opioid prescribing/dispensing.

Step 4. Identification of next steps for implementation of selected EBP strategies

Once EBP strategies are chosen, coalitions are encouraged to list partner agencies. In three states, coalition-listed agencies are suggested as potential partners for implementation (KY, NY, OH). In MA, the partner agencies for implementation are determined by coalition subgroups as part of the planning process and voted on by the coalitions, with the acceptation of agencies delivering office-based addictions treatment; these agencies were preselected by the study team. Coalition review of selected EBP strategies in one state (KY) also involves members describing what the coalition could do to support rollout (e.g., write op-eds in the local paper in support of the EBP strategy, provide warm handoffs to agencies, etc.) and ways to reach underserved populations with a primary focus on people of color and Spanish speaking individuals. Iteratively with coalition review, HCS faculty and community staff in each state conduct internal reviews. In MA, state government partners are also involved in the internal review. Internal reviews focus on ensuring the EBP strategies selected adhere to the ORCAA menu and include the required high-risk populations and sectors, potential impact and feasibility. Internal teams also determine next steps for team members, such as initial meetings with partner organizations, contracting considerations, and provision of technical assistance. In one state (MA), the review also includes planning for the mitigation of inequities associated with each EBP strategy and

For peer review only

examination of each EBP strategy's cost as estimated by coalitions with technical assistance from community staff and HCS faculty.

Upon completion of the steps described above, descriptions of selected EBP strategies are provided to faculty and staff teams with expertise in implementation science, OEND, MOUD, safer opioid prescribing/dispensing, and/or community engagement to start the process of working with agency partners and explore interest in and capacity for implementation of the coalitions selected EBP strategies. This process varies from site to site and involves the development of an agency specific implementation plan.

Ethics and Dissemination

The HEALing Communities Study protocol was approved by a central Institutional Review Board (IRB; Advarra Inc., Pro00038088) and is being conducted in accordance with The Code of Ethics of the World Medical Association (Declaration of Helsinki). As described elsewhere,[16] because no one person or group of people possess(es) the authority to give consent on behalf of all community members, investigators sought expert consultation and applied guidelines from the Ottawa Statement.[64] Because the CTH intervention poses no more than minimal risk to community members and the research could not be carried out otherwise (see 45.CFR.46.116), a waiver of informed consent was obtained for all community members who may be affected by the CTH intervention.

The action planning tools are disseminated to coalition members in hardcopy, via email, and/or through a password-protected, online portal. We plan to disseminate updated versions of these tools to communities currently in the waitlist-control group via the same mechanisms when intervention begins in their communities. Results of the action planning process (i.e., EBP

For peer review only

strategy selections) are shared with community members, community agencies, and community advisory boards through meetings, online and print media, and presentations to community stakeholder groups in the intervention communities. In the future, we also plan to disseminate our findings at relevant conferences, meetings and through peer-reviewed journals. Finally, we will disseminate our findings, manuals, toolkits, and other resources through federal partners including the National Institutes of Health, Substance Abuse and Mental Health Services Administration and the Centers for Disease Control and Prevention.

For peer review only

Table 1. Characteristics of action planning process that were unique to certain states

**Action Planning Steps**	**Characteristics**	**KY**	**MA**	**NY**	**OH**
Step 1. Develop Data-driven Community Goals	*Supplementary factors considered by coalitions:*				
	• Agent-based and systems dynamic modeling			X	
	• EBP strategy sustainability		X	X	
	• Reach to high-risk populations and priority venues		X		X
	• Equity across ethnic and racial groups		X	X	
Step 2. Discuss and Prioritize EBP strategies	Development of EBP strategy list considered by coalition	Pre-populated by the academic team	Generated by coalitions[1]	Generated by coalitions[1]	Generated by coalitions[1]
	Method of prioritizing strategies	Likert scale ratings of size of current service gap, feasibility, and potential impact.	Consensus building group dialogues	Rank by impact and feasibility	Rank by impact and feasibility
	Coalition consideration of cost	Encouraged *not* to consider cost, but to focus on need, impact, and feasibility of each EBP strategy.	Provided with budget cap and required to consider costs	Encouraged, but not required	Encouraged, but not required
	Supplementary factors considered by coalitions:				
	• EBP strategy sustainability		X	X	
	• Method of EBP expansion (i.e., new service, or scale-up of an existing service via a new method of delivery, target population, or feature)			X	X

For peer review only - http://bmjopen.bmj.com/site/about/guidelines.xhtml

**Action Planning Steps**	**Characteristics**	**KY**	**MA**	**NY**	**OH**
Step 3. Select EBP strategies to include in action plan	Limits on strategy selections	12 initial EBP strategies	No limit	No limit	No limit
Step 4. Identify next steps for implementation of selected EBP strategies	Coalition input on agencies to be involved in implementation	Coalitions suggest potential agencies to involve in implementation.	Coalition subgroups determine which agencies will be involved in implementation.	Coalitions suggest potential agencies to involve in implementation.	Coalitions suggest potential agencies to involve in implementation.
	Coalition input on next steps for beginning implementation	Coalition describes what they can do to support implementation and reach underserved populations who may experience disparities in access.	Coalitions estimate cost of each strategy with technical assistance from the academic team.	None beyond action plan established in Step 3	None beyond action plan established in Step 3

EBP: evidence-based practices

[1] "Coalitions" is inclusive of full coalitions, coalition workgroups, and coalition champions with expertise in specific EBPs

Authors' contributions

The following individuals contributed to the design of the action planning protocol described in this manuscript: AY, PRF, LSM, LF, MRL, JB, MSL, TH, AB, BR, RBS, and TAB. The following individuals made substantial contributions to the implementation of the action planning protocol: AY, PRF, AF, LF, JG, CK, MRL, HS, SLW, JB, MSL, TH, AB, and MQ. The following individuals made substantial contributions to the drafted work and/or substantively revised it: AY, JB, TH, LSM, RC, EO, TW, TAB, TB, RBS, AB, AF, LF, LMG, PRF, JG, CK, MSL, MRL, MQ, BR, HS, SLW. All authors read and approved the final manuscript and agree to be accountable for all aspects of the work.

Funding statement

This research was supported by the National Institutes of Health through the NIH HEAL (Helping to End Addiction Long-term^{SM}) Initiative under award numbers UM1DA049394, UM1DA049406, UM1DA049412, UM1DA049415, UM1DA049417 (ClinicalTrials.gov Identifier: NCT04111939). The Substance Abuse and Mental Health Services Administration (SAMHSA) is a non-funding sponsor of the study. This study protocol (Pro00038088) was approved by Advarra Inc., the HEALing Communities Study single Institutional Review Board. We wish to acknowledge the participation of the HEALing Communities Study communities, community coalitions, and Community Advisory Boards and state government officials who partnered with us on this study. The content is solely the responsibility of the authors and does not necessarily represent the official views of the National Institutes of Health, the Substance Abuse and Mental Health Services Administration or the NIH HEAL Initiative^{SM}.

Competing interest statement

Competing interest disclosures include the following: LSM is a consultant for the Boston Public Health Commission, Action for Boston Area development and the Young Foundation, SLW has served as a scientific advisor to Opiant Pharmaceuticals, Titan Pharmaceuticals, Astra Zeneca, Cerevel, Arbor Pharmaceuticals, Brainsway, Summit Biosciences, Trevi and Camurus. JB ad ML receive investigator-initiated support paid to the institution from Gilead Sciences, Inc. MRL has consulted for Titan Pharmaceuticals and Camurus. AFB is the Program Director of Voices of Hope, a University of Kentucky HCS partner organization. PRF, HS, AY, EO, LMG, RC, JG, CK and LCF have no competing interests.

Data Sharing Statement

The protocol described in this manuscript is for a community-engaged action planning process by which communities choose strategies to implement evidence-based practices in their communities. Data for this paper are considered to be the tools such as decision aids, action plans, and other documents used to facilitate the process and are included as a Supplementary Appendix. The protocol described in this manuscript is part of a larger trial for which data are of a different type; those data are described in the trial registry.

Acknowledgments

We wish to acknowledge the participation of the HEALing Communities Study communities, community coalitions, and Community Advisory Boards and state government officials who partnered with us on this study. We extend our sincere gratitude to the staff and community

For peer review only - http://bmjopen.bmj.com/site/about/guidelines.xhtml

members who have dedicated their time and passion to this project and without whom the HEALing Communities Study would not be possible.

For peer review only

References

1. Krausz RM, Westenberg JN, Ziafat K. The opioid overdose crisis as a global health challenge. *Current Opinion in Psychiatry* 2021;34(4):405-12.

2. World Health Organization. Opioid overdose 2021 [updated August 4 2021. Available from: https://www.who.int/news-room/fact-sheets/detail/opioid-overdose accessed August 15 2021.

3. National Institute on Drug Abuse. Overdose Death Rates Bethesda, MD2021 [updated January 29, 2021. Available from: https://www.drugabuse.gov/drug-topics/trends-statistics/overdose-death-rates accessed August 15 2021.

4. Ahmad FB RL, Sutton P. Provisional drug overdose death counts: National Center for Health Statistics. Designed by LM Rossen, A Lipphardt, FB Ahmad, JM Keralis, and Y Chong: National Center for Health Statistics., 2021.

5. Strang J, Volkow ND, Degenhardt L, et al. Opioid use disorder. *Nature Reviews Disease Primers* 2020;6(1):3. doi: 10.1038/s41572-019-0137-5

6. Bohnert AS, Guy Jr GP, Losby JL. Opioid prescribing in the United States before and after the Centers for Disease Control and Prevention's 2016 opioid guideline. *Annals of internal medicine* 2018;169(6):367-75.

7. Walley AY, Xuan Z, Hackman HH, et al. Opioid overdose rates and implementation of overdose education and nasal naloxone distribution in Massachusetts: interrupted time series analysis. *British Medical Journal* 2013;346

8. Larochelle MR, Bernson D, Land T, et al. Medication for opioid use disorder after nonfatal opioid overdose and association with mortality: a cohort study. *Annals of internal medicine* 2018;169(3):137-45.

9. Wu E, Villani J, Davis A, et al. Community dashboards to support data-informed decision-making in the HEALing communities study. *Drug and alcohol dependence* 2020;217:108331-31. doi: 10.1016/j.drugalcdep.2020.108331

10. Welty L, Harrison A, Abram K, et al. Substance Abuse and Mental Health Services Administration.(2017). Key substance use and mental health indicators in the United States: Results from the 2016 National Survey on Drug Use and Health (HHS Publication No. SMA 17-5044, NSDUH Series H-52). Rockville, MD: Center for Behavioral Health Statistics and Quality. *Substance Abuse and Mental Health Services Administration Retrieved College of Health Sciences* 2019;106(5):128.

11. Williams AR, Nunes EV, Bisaga A, et al. Developing an opioid use disorder treatment cascade: A review of quality measures. *Journal of substance abuse treatment* 2018;91:57-68.

For peer review only

12. Freeman PR, Hankosky ER, Lofwall MR, et al. The changing landscape of naloxone availability in the United States, 2011–2017. *Drug and alcohol dependence* 2018;191:361-64.

13. Guy Jr GP, Zhang K, Bohm MK, et al. Vital signs: changes in opioid prescribing in the United States, 2006–2015. *MMWR Morb Mortal Wkly Rep* 2017;66(26):697.

14. Guy Jr GP, Zhang K, Halpin J, et al. An examination of concurrent opioid and benzodiazepine prescribing in 9 states, 2015. *American journal of preventive medicine* 2019;57(5):629-36.

15. Guy GP, Zhang K, Schieber LZ, et al. County-level opioid prescribing in the United States, 2015 and 2017. *JAMA internal medicine* 2019;179(4):574-76.

16. Walsh SL, El-Bassel N, Jackson RD, et al. The HEALing (Helping to End Addiction Long-term SM) Communities Study: Protocol for a cluster randomized trial at the community level to reduce opioid overdose deaths through implementation of an integrated set of evidence-based practices. *Drug and Alcohol Dependence* 2020;217:108335.

17. Martinez LS, Rapkin BD, Young A, et al. Community engagement to implement evidence-based practices in the HEALing Communities Study. *Drug and Alcohol Dependence* 2020;217:108326.

18. Winhusen T, Walley A, Fanucchi LC, et al. The opioid-overdose reduction continuum of care approach (ORCCA): evidence-based practices in the HEALing communities study. *Drug and Alcohol Dependence* 2020;217:108325.

19. Lefebvre RC, Chandler RK, Helme DW, et al. Health communication campaigns to drive demand for evidence-based practices and reduce stigma in the HEALing Communities Study. *Drug and alcohol dependence* 2020;217:108338.

20. Rosenthal BB, Greenawalt J, Ivery JM, et al. Coalitions: Essential tools for organizing. In: Staples L, ed. Roots to Power: A Manual for Grassroots Organizing: A Manual for Grassroots Organizing. London: Praeger 2016:297.

21. Oesterle S, Kuklinski MR, Hawkins JD, et al. Long-Term Effects of the Communities That Care Trial on Substance Use, Antisocial Behavior, and Violence Through Age 21 Years. *Am J Public Health* 2018;108(5):659-65.

22. Shapiro VB, Hawkins JD, Oesterle S. Building local infrastructure for community adoption of science-based prevention: The role of coalition functioning. *Prevention Science* 2015;16(8):1136-46.

23. Arthur MW, Hawkins JD, Brown EC, et al. Implementation of the Communities That Care prevention system by coalitions in the Community Youth Development Study. *Journal of Community Psychology* 2010;38(2):245-58.

24. Butterfoss FD. Coalitions and partnerships in community health: John Wiley & Sons 2007.

For peer review only

25. Wandersman A, Goodman RM, Butterfoss FD. Understanding coalitions and how they operate. In: Minkler M, ed. Community organizing community building for health. 2 ed. New Brunswick, NJ Rutgers University Press 1997:261-77.

26. Powell BJ, McMillen JC, Proctor EK, et al. A compilation of strategies for implementing clinical innovations in health and mental health. *Medical care research review* 2012;69(2):123-57.

27. Rugs D, Hills HA, Moore KA, et al. A community planning process for the implementation of evidence-based practice. *Evaluation and Program Planning* 2011;34(1):29-36.

28. Pellecchia M, Mandell DS, Nuske HJ, et al. Community–academic partnerships in implementation research. *Journal of Community Psychology* 2018;46(7):941-52.

29. Centers for Disease Control and Prevention. Principles of Community Engagement. Second ed, 2011.

30. World Health Organization. WHO community engagement framework for quality, people-centred and resilient health services. : World Health Organization, 2017.

31. World Health Organization. Community engagement: a health promotion guide for universal health coverage in the hands of the people . Geneva: World Health Organization,,, 2020.

32. C. D. I. Study Group. Community-directed interventions for priority health problems in Africa: results of a multicountry study. *Bull World Health Organ* 2010;88(7):509-18. doi: 10.2471/BLT.09.069203 [published Online First: 2009/12/01]

33. World Health Organization & United Nations Children's Fund (UNICEF). Conducting community engagement for COVID-19 vaccines: interim guidance, 31 January 2021: World Health Organization, 2021.

34. Winhusen T, Theobald J, Lewis D, et al. Development and initial testing of a tailored telephone intervention delivered by peers to prevent recurring opioid-overdoses (TTIP-PRO). *Health education research* 2016;31(2):146-60. doi: 10.1093/her/cyw010

35. Caudarella A, Dong H, Milloy MJ, et al. Non-fatal overdose as a risk factor for subsequent fatal overdose among people who inject drugs. *Drug Alcohol Depend* 2016;162:51-5. doi: 10.1016/j.drugalcdep.2016.02.024

36. Darke S, Mills KL, Ross J, et al. Rates and correlates of mortality amongst heroin users: findings from the Australian Treatment Outcome Study (ATOS), 2001-2009. *Drug Alcohol Depend* 2011;115(3):190-5. doi: 10.1016/j.drugalcdep.2010.10.021 [published Online First: 2010/12/07]

37. Larochelle MR, Bernstein R, Bernson D, et al. Touchpoints - Opportunities to predict and prevent opioid overdose: A cohort study. *Drug and alcohol dependence* 2019;204:107537-37. doi: 10.1016/j.drugalcdep.2019.06.039 [published Online First: 2019/09/03]

For peer review only

38. Strang J, McCambridge J, Best D, et al. Loss of tolerance and overdose mortality after inpatient opiate detoxification: follow up study. *British Medical Journal* 2003;326(7396):959-60. doi: 10.1136/bmj.326.7396.959

39. Binswanger IA, Stern MF, Deyo RA, et al. Release from prison—a high risk of death for former inmates. *New England Journal of Medicine* 2007;356(2):157-65. doi: 10.1056/NEJMsa064115

40. Merrall ELC, Kariminia A, Binswanger IA, et al. Meta-analysis of drug-related deaths soon after release from prison. *Addiction* 2010;105(9):1545-54. doi: 10.1111/j.1360-0443.2010.02990.x

41. Walley AY, Lodi S, Li Y, et al. Association between mortality rates and medication and residential treatment after in-patient medically managed opioid withdrawal: a cohort analysis. *Addiction (Abingdon, England)* 2020;115(8):1496-508. doi: 10.1111/add.14964 [published Online First: 2020/02/25]

42. Sun EC, Dixit A, Humphreys K, et al. Association between concurrent use of prescription opioids and benzodiazepines and overdose: retrospective analysis. *British Medical Journal* 2017;356:j760-j60. doi: 10.1136/bmj.j760

43. Brugal MT, Barrio G, De LF, et al. Factors associated with non-fatal heroin overdose: assessing the effect of frequency and route of heroin administration. *Addiction* 2002;97(3):319-27. doi: 10.1046/j.1360-0443.2002.00058.x [published Online First: 2002/04/20]

44. Cho J, Spence MM, Niu F, et al. Risk of Overdose with Exposure to Prescription Opioids, Benzodiazepines, and Non-benzodiazepine Sedative-Hypnotics in Adults: a Retrospective Cohort Study. *J Gen Intern Med* 2020;35(3):696-703. doi: 10.1007/s11606-019-05545-y [published Online First: 2020/01/09]

45. Gladden RM, O'Donnell J, Mattson CL, et al. Changes in Opioid-Involved Overdose Deaths by Opioid Type and Presence of Benzodiazepines, Cocaine, and Methamphetamine - 25 States, July-December 2017 to January-June 2018. *MMWR Morb Mortal Wkly Rep* 2019;68(34):737-44. doi: 10.15585/mmwr.mm6834a2

46. Park TW, Larochelle MR, Saitz R, et al. Associations between prescribed benzodiazepines, overdose death and buprenorphine discontinuation among people receiving buprenorphine. *Addiction (Abingdon, England)* 2020;115(5):924-32. doi: 10.1111/add.14886 [published Online First: 2020/01/20]

47. O'Driscoll PT, McGough J, Hagan H, et al. Predictors of accidental fatal drug overdose among a cohort of injection drug users. *American journal of public health* 2001;91(6):984-87. doi: 10.2105/ajph.91.6.984

48. Pabayo R, Alcantara C, Kawachi I, et al. The role of depression and social support in non-fatal drug overdose among a cohort of injection drug users in a Canadian setting. *Drug*

For peer review only

and alcohol dependence 2013;132(3):603-09. doi: 10.1016/j.drugalcdep.2013.04.007 [published Online First: 2013/05/04]

49. Wines JD, Jr., Saitz R, Horton NJ, et al. Overdose after detoxification: a prospective study. *Drug Alcohol Depend* 2007;89(2-3):161-9. doi: 10.1016/j.drugalcdep.2006.12.019 [published Online First: 2007/02/07]

50. Tobin KE, Latkin CA. The relationship between depressive symptoms and nonfatal overdose among a sample of drug users in Baltimore, Maryland. *J Urban Health* 2003;80(2):220-29. doi: 10.1093/jurban/jtg025

51. Bosilkovska M, Walder B, Besson M, et al. Analgesics in patients with hepatic impairment: pharmacology and clinical implications. *Drugs* 2012;72(12):1645-69. doi: 10.2165/11635500-000000000-00000 [published Online First: 2012/08/08]

52. Green TC, McGowan SK, Yokell MA, et al. HIV infection and risk of overdose: a systematic review and meta-analysis. *AIDS* 2012;26(4):403-17. doi: 10.1097/QAD.0b013e32834f19b6

53. Jolley CJ, Bell J, Rafferty GF, et al. Understanding Heroin Overdose: A Study of the Acute Respiratory Depressant Effects of Injected Pharmaceutical Heroin. *PLoS One* 2015;10(10):e0140995-e95. doi: 10.1371/journal.pone.0140995

54. Vu Q, Beselman A, Monolakis J, et al. Risk factors for opioid overdose among hospitalized patients. *J Clin Pharm Ther* 2018;43(6):784-89. doi: 10.1111/jcpt.12701 [published Online First: 2018/05/26]

55. Bazazi AR, Zelenev A, Fu JJ, et al. High prevalence of non-fatal overdose among people who inject drugs in Malaysia: Correlates of overdose and implications for overdose prevention from a cross-sectional study. *International Journal on Drug Policy* 2015;26(7):675-81. doi: 10.1016/j.drugpo.2014.11.010

56. Slavova S, Quesinberry D, Hargrove S, et al. Trends in Drug Overdose Mortality Rates in Kentucky, 2019-2020. *JAMA network open* 2021;4(7):e2116391-e91. doi: 10.1001/jamanetworkopen.2021.16391

57. Slavova S, Rock P, Bush HM, et al. Signal of increased opioid overdose during COVID-19 from emergency medical services data. *Drug and alcohol dependence* 2020;214:108176-76. doi: 10.1016/j.drugalcdep.2020.108176

58. Stephenson J. Drug Overdose Deaths Head Toward Record Number in 2020, CDC Warns. *JAMA Health Forum* 2020;1(10):e201318-e18. doi: 10.1001/jamahealthforum.2020.1318

59. Wakeman SE, Green TC, Rich J. An overdose surge will compound the COVID-19 pandemic if urgent action is not taken. *Nature Medicine* 2020;26(6):819-20. doi: 10.1038/s41591-020-0898-0

For peer review only

60. Holland KM, Jones C, Vivolo-Kantor AM, et al. Trends in US emergency department visits for mental health, overdose, and violence outcomes before and during the COVID-19 pandemic. *JAMA psychiatry* 2021;78(4):372-79.

61. Friedman J, Beletsky L, Schriger DL. Overdose-related cardiac arrests observed by emergency medical services during the US COVID-19 epidemic. *JAMA psychiatry* 2021;78(5):562-64.

62. Centers for Disease Control and Prevention. Overdose Deaths Accelerating During COVID-19 2020 [Available from: https://www.cdc.gov/media/releases/2020/p1218-overdose-deaths-covid-19.html accessed May 19 2021.

63. Centers for Disease Control and Prevention. Increase in Fatal Drug Overdoses Across the United States Driven by Synthetic Opioids Before and During the COVID-19 Pandemic: CDC Health Alert Network, 2020.

64. Taljaard M, Weijer C, Grimshaw JM, et al. The Ottawa Statement on the ethical design and conduct of cluster randomised trials: precis for researchers and research ethics committees. *British Medical Journal* 2013;346:f2838. doi: 10.1136/bmj.f2838

For peer review only

Figure 1. Guidelines for HEALing Communities Study Community Action Planning Process to reduce opioid overdose deaths through overdose education and naloxone distribution, expansion of medications for opioid use disorder, and safer opioid prescribing and dispensing

For peer review only

Figure 2. Core components of the HEALing Communities Study Community Action Planning Process to reduce opioid overdose deaths through overdose education and naloxone distribution, medications for opioid use disorder, and safer opioid prescribing and dispensing

For peer review only

- Coalitions *must* select a minimum of five strategies for implementing evidence-based practices (EBPs) from the ORCCA menus including one involving each of the following
  - ORCCA Menu 1: Active OEND (i.e., proactively offer OEND to those at risk)
  - ORCCA Menu 2A: Expand MOUD access
  - ORCCA Menu 2B: Increase linkage to MOUD
  - ORCCA Menu 2C: Improve retention in MOUD
  - ORCCA Menu 3: Safer opioid prescribing/dispensing

- Priority sectors: Selected EBP strategies *must* include at least one to be implemented in each of the following:
  - Healthcare (i.e., outpatient healthcare centers, pre-hospital providers, emergency departments and urgent care, hospitals, primary care settings, public health departments, and pharmacies)
  - Behavioral health (i.e., substance use disorder and mental health treatment centers and social service agencies, including syringe service programs)
  - Criminal justice (i.e., pre-trial, jails, probation, parole, drug and problem-solving courts, police and "narcotics" task forces, halfway houses, community-based correctional facilities, and department of youth services)

- Key populations: Coalitions are encouraged to consider EBP strategies focused on those most vulnerable to opioid overdose:
  - People with a prior opioid overdose
  - People with reduced opioid tolerance
  - People who use other substances
  - People who have concomitant major mental illness
  - People who have a concomitant major medical illness
  - People who inject drugs

- Priority venues: Coalitions are encouraged to consider EBP strategies to be implemented in priority settings:
  - OEND: criminal justice settings, syringe service programs, emergency departments and acute care hospitals, sites of overdose for naloxone leave-behind, and mental health/addiction treatment programs
  - MOUD: criminal justice settings, primary care, general medical and behavioral/mental health settings, specialty addiction/substance use disorder treatment settings, and recovery programs
  - Safer opioid prescribing and dispensing: inpatient service, emergency/urgent care, outpatient clinics, ambulatory surgery, dental clinics, pharmacies

Figure 1. Guidelines for HEALing Communities Study Community Action Planning Process to reduce opioid overdose deaths through overdose education and naloxone distribution, expansion of medications for opioid use disorder, and safer opioid prescribing and dispensing

165x210mm (300 x 300 DPI)

1) Develop data-driven community goals

- Coalitions reviewed (1) lists of organizations in the community relevant to potential implementation of EBP strategies (e.g., substance use disorder treatment centers, harm reduction programs, emergency response agencies, hospitals), and (2) information about OEND, MOUD, and other related services available through administrative data and/or through phone surveys with community agencies. Coalitions used goal setting tools to describe strengths, services, gaps, and set goals with varying specificity.

2) Discuss and prioritize EBP strategies

- Coalitions reviewed decision aid tools (Supplementary Appendix) listing EBPs. Coalitions discussed relevant existing services, size of the gap between existing services and need, feasibility, and potential impact on opioid-involved overdose deaths. In some cases, coalitions scored the EBPs on Likert scales and/or ranked them according to impact and feasibility.

3) Select EBP strategies to include in the community action plan

- Coalitions reviewed narrative notes, scores, and rankings from the process described above and selected by consensus or majority vote at least five EBPs, including one each from the following categories: active OEND, MOUD expansion, linkage to MOUD, retention in MOUD, and safer opioid prescribing and dispensing.

4) Identify next steps for implementation of selected EBP strategies

- Coalitions suggested agencies that could partner to implement their selected strategies. Iteratively with coalition review, subject matter experts and staff reviewed strategies to determine next steps for team members (e.g., initial meetings with partner organizations, contracting considerations, and technical assistance).

OEND: overdose education and naloxone distribution, MOUD: medications for opioid use

Figure 2. Core components of the HEALing Communities Study Community Action Planning Process to reduce opioid overdose deaths through overdose education and naloxone distribution, medications for opioid use disorder, and safer opioid prescribing and dispensing

162x153mm (300 x 300 DPI)

Appendix 1. Kentucky Action Planning Tools

Community Goal Setting Tool

Purpose: To describe existing infrastructure, gaps, opportunities, needs, and goals related to opioid overdose prevention.

Description: The Kentucky Community Engagement and Continuum of Care teams partnered to facilitate communities' identification of their strengths and opportunities for expansion related to opioid overdose prevention followed by thoughtful consideration and selection of the ORCCA strategies. The process began with the Community Engagement team working with the coalition to describe existing infrastructure, gaps, opportunities, needs, and goals related to opioid overdose prevention in their communities using a Community Goal Setting Tool. The coalitions and/or menu-specific workgroups worked on completing the tool for each menu separately and presenting them to the full coalition.

Who completes the tool: Coalitions and/or coalition workgroups with facilitation by faculty and staff.

Community Goal Setting Tool — Menu 1

Menu 1 Goal: Increase Opioid Overdose Prevention Education and Naloxone Distribution (OEND)

Brainstorm! Think about your community: what you've experienced, what you learned from your community profile and data dashboard, and what you envision for the future. Answer the questions below and jot down your thoughts

What are we doing well? What OEND services already exist?

Ex. There is active Opioid Overdose Education and Naloxone Distribution (OEND) already occurring in the County Detention Center. The Detention Center is currently able to provide naloxone to all persons released from the facility (as part of partnership with the HEALing Communities Study) and is also partnering with other organizations to distribute naloxone to anyone who is interested and completes the requisite virtual training.

Who needs OEND in our community?

Ex. There is still a significant need for distribution among the peer community and their social networks, in homes with bystanders, and with medical and law enforcement professionals in contact with those at high-risk for overdose.

Where in our community should OEND services be provided?

Ex. There is significant opportunity for OEND expansion in Emergency Departments and the healthcare system generally, in pharmacies, with local Quick Response Teams (QRTs), in Substance Use Disorder (SUD) treatment facilities, and in other locations where the peer community can be found. The NKY Health Department also has capacity to expand OEND services.

Community OEND Goals:

Example:

1. Expand OEND in criminal justice settings, including Probation & Parole and Drug Court.
2. Expand OEND in healthcare settings, including Emergency Departments and Substance Use Disorder (SUD) treatment programs.
3. Expand OEND access among peer community and their support systems (via recovery support groups, social service agencies, and in "hot spots" frequented by those at high-risk, etc.).
4. Expand OEND with local QRTs.
5. Increase first responders carrying/administering naloxone.

Decision Aid Tool

Purpose: To facilitate discussion and prioritization of evidence-based practice strategies that align with community goals and the ORCCA

Description: HCS staff then populated menu-specific Decision Aid Tools with strategies listed in the ORCCA and TAG, integrating coalition's suggestions for specific strategies when available from Community Goal Setting Tool. In meetings facilitated by faculty and staff with expertise in community-engagement and the continuum of care, the coalitions and/or menu-specific workgroups reviewed each possible strategy one-by-one, offering a narrative description of factors that they believed would be relevant to deciding whether to implement the strategy (i.e., current relevant activities, barriers they anticipate encountering, agencies relevant to implementation). After offering a narrative description, the coalition scored on Likert scales the size of the current gap in the service/strategy (4-point scale; 0-3), feasibility of implementation within the next 12 months (4-point scale; 0-3), and its potential impact on overdose deaths (3-point scale; 1-3). Each dimension was scored through consensus or through in-meeting polling. Coalitions did not use specific criteria or thresholds in assigning the scores, rather they often scored them based on how they compared to others on the menu. Scores were summed to produce an overall "priority score" for each strategy.

Who completes the tool: Coalitions and/or coalition workgroups with facilitation by faculty and staff.

For peer review only

Menu 2: Strategies to Enhance Delivery of MOUD Maintenance Treatment, Including Agonist/Partial Agonist Medication					
**Strategy and Venue**	**Current Activity**	**Size of Gap** (Current Activity vs. Need)	**Feasibility in <12 Months**	**Impact on Overdose Deaths**	**Priority Score**
Working with the ORCCA menu, brainstorm possible strategies to meet the Community MOUD goals. Record all strategies in this column.	Use this column to briefly summarize current activity in your community	0. None<br>1. Small<br>2. Medium<br>3. Large	0. Extremely Low<br>1. Small<br>2. Medium<br>3. Large	1. Small<br>2. Medium<br>3. Large	Add previous 3 columns; if any column contains 0, priority score is 0
**a) Expand MOUD Treatment Availability (Capacity Building) – REQUIRED**					
**Add/expand MOUD in primary care, other general medical/BH settings, and specialty addiction/SUD & recovery programs**					
Ex. Expand Methadone in county OTPs 2.a.1.2	Ex. Have two OTP providers but one has long wait times; need to figure out what they need to be able to treat more patients				
**Add/expand MOUD treatment in Criminal Justice Settings (pre trial, jail, prison, probation, parole)**					
Ex. Expand prescription of XR naltrexone and buprenorphine within jail	Ex. Interest in XR naltrexone at the jail and jail might be open but have a lot of concerns/barriers				
**Expand access to MOUD through telemedicine, interim buprenorphine or methadone, or medication units**					
Ex. Expand use of Telemedicine/telehealth/tele-consults for buprenorphine treatment	Ex. Telehealth opportunities have grown due to COVID 19, but it is unknown whether this option will remain available.				
**b) Interventions to Link to MOUD – REQUIRED**					
**Linkage Programs (All Relevant settings)**					
Ex. Develop linkage program for incarcerated individuals about to be released (with peer support and/or other staff)	Ex. No current activity, the jail is somewhat interested but there are several barriers				
**Bridging MOUD medications as Linkage Adjunct (all relevant settings)**					
Ex. Expand bridge programs within county ER and inpatient settings	Ex. An existing bridge program is working well and can use as model for others. Difficulty with staffing & infrastructure is main barrier				
**c) MOUD Treatment Engagement and Retention – REQUIRED**					
**Enhancement of clinical delivery approaches that support engagement and retention**					
Ex. Expand recovery support services (peer support, telephone recovery support, recovery coaching, family support) in criminal justice settings (Voices of Hope)	Ex. Add peer supports within system; no current long term supports, or programs/mentorship programs engaged				
**Use virtual retention approaches (e.g. mobile, web, digital therapeutics)**					
Ex. Implement use of re-SET-0 app to improve retention, incentives, and education 2.c.2	Ex. Some clinics within county already using it; potential cost barriers and potential disinterest from buprenorphine providers				
**Utilize retention care coordinators**					
Ex. Utilize retention care navigators to increase/add care navigators in all MOUD treatment settings 2.c.3	Ex. Other care navigation efforts are already underway that could be leveraged, and there is a desire to increase support of and leverage local efforts where possible				
**Integrate mental health and polysubstance use treatment into MOUD care**					
Ex. Offer regular training between primary care/non MH providers with addiction psychiatry and other MH providers to manage co-morbidity with OUD	Ex. Unsure if providers would be interested in engaging				
**Reduce barriers to housing, transportation, childcare, and access to other community benefits for People with OUD**					
Expand transportation services/offerings to align with MOUD treatment center services (location, hours, etc.)	Ex. Local agency offers transportation services to clients				

Action Planning Tool

Purpose: To facilitate discussion and final strategy selection.

Description: The list of strategies discussed in the Decision Aid Tool was used to pre-populate an Action Planning Tool. The tool listed all strategies with their scores and new implementation feasibility ratings (High, Medium, Low) provided by faculty with expertise in implementation science and the continuum of care based on their knowledge of policy- and payor-level barriers and time required for scale up. The pre-populated Action Planning Tools were presented to coalitions for discussion and strategy selection through consensus.

Who completes the tool: Coalitions and/or coalition workgroups with facilitation by faculty and staff.

Action Planning Tool — Menu 3

For each strategy from the Decision Aid that had a priority score above 0, fill in the strategy and venue (i.e., from Column 1 of the Decision Aid), record the scores from the Decision Aid Tool in the Scores columns, and fill in the relevant sector. As coalitions consider their selection of strategies, they should ensure that strategies collectively will target those at highest risk (i.e., people with history of prior overdose, reduced opioid tolerance, who engage in polydrug use, with major medical or mental illness, and/or who inject drugs). The HCS Research Team will help you complete the Implementation Feasibility column by providing insight on whether each strategy has a high, medium, or low feasibility of implementation based on resources, partnerships, and other factors. After reviewing content of the other columns, coalitions should come to consensus on the Overall Priority Ranking with the highest priority strategy ranked 1, the second highest priority ranked 2, and so on. Of note, the Overall Priority Ranking may or may not correspond directly to the rank order from the other scoring columns. After completing this tool, the next step will involve creating an initial implementation plan for the top 1-3 overall priority ranked strategies; at least one of the strategies must be from the required sub-menu (i.e., Safe Prescribing/Dispensing).

Menu 3: Strategies to Improve Prescription Opioid Safety							
Strategy and Venue	Scores				Sector?	Implementation Feasibility? (High/Medium/Low)	Overall Priority Ranking
	G	F	I	PS			
**a) Safer Opioid Prescribing/Dispensing Practices (Required)**							
Ex. Education materials around safe opioid disposal, targeting healthcare providers and patients.	3	3	2.25	8.25	HC	High	1
Ex. Education for community pharmacists focusing on red flags during opioid dispensing (dose, indication, duration, concurrent therapies, comorbid conditions, age, etc.).	1.25	2.5	1.5	5.25	HC	High	2
Ex. Educational outreach and academic detailing related to pain management for patients with OUD	2.5	2	2	6.5	HC	Medium	
Ex. Patient educational materials regarding appropriate chronic pain management made available in AA meetings and OUD recovery groups	2.5	2.25	1.75	6.5	BH	Medium	3
**b) Safer Opioid Disposal Practices (Optional)**							
Ex. Installation of permanent disposal kiosks	1.8	2.7	2	6.5	HC	High	4
Ex. Expansion and promotion of take-back events	2.3	2.3	2.25	6.85	Other	Medium	

Priority Score: G: Gap; F: Feasibility; I: Impact; PS: Overall Priority Score

Sector: CJ = Criminal Justice, HC = Healthcare, BH = Behavioral Health (See ORCCA Overview for details on assignment of sectors).

Coalition Implementation Planning Tool

Purpose: To more thoroughly define strategies and generate potential lists of venues/agencies to be involved in the implementation

Description: The tool also captured coalition's feedback on what they could do to help support implementation, strategies to ensure reach to under-represented populations, and indicators of success or failure of the strategy. Faculty with expertise in implementation science and the continuum of care added details on what the HCS faculty and staff would do to implement the strategy. Once complete, the tools were reviewed and approved in their entirety by the coalitions.

Who completes the tool: Coalitions and/or coalition workgroups with faculty and staff.

Coalition Implementation Planning Tool — Menu 3

For at least one but no more than three of the strategies with the top Overall Ranking from the Action Plan table, create an initial implementation plan using the table below (i.e., one table per strategy for up to three strategies). At least one of these strategies must come from the required sub-menu in the action plan (i.e., must address safe prescribing/dispensing practices). Provide information about specific activities in as much detail as is feasible at this time; insert additional rows as needed to describe these activities for the coalition and the HCS team.

**Menu 3 Overall Ranking = 1**<br>**Strategy: Educational outreach and academic detailing related to opioid prescribing for routine pain management. (Includes multiple sub-strategies as outlined on the Community Action Planning Tool).**		
**What specifically will be done?** Ex: Educational outreach and academic detailing for all willing opioid prescribers (and possibly ambulatory pharmacists) in the County. Top priorities are: Primary Care, Hospital Emergency Department & local Urgent Care facilities, Dental Offices, Ortho Practices, Pain Management Clinics		
**What organization(s) should be involved? Provide points of contact if possible.**	Agency: Ex. Family Practice	Contact: Ex. Dr. Smith
	Agency: Ex. Pharmacy	Contact: Ex. Dr. Jones
	Agency:	Contact:
**What will the coalition do?**		**Assigned to:**
1. Ex. Facilitate contact with Family Practice, Ambulatory Pharmacists		1. Ex. Coalition Chair
2. Ex. Help with endorsement letter (with signatures from local hospital MDs		2. Ex. Safe Prescribing Champion
**What will HCS do?**		**Assigned to:**
1. Coordinate with listed coalition members to connect with identified organizations.		1. Ex. Prevention Team
2. Draft a promotional letter/email to be reviewed and signed by community leaders who healthcare professionals respect and would positively respond to and recognize.		2. Ex. Prevention Team
3. Develop community-specific implementation plan, target list, and educational materials using audiences and messaging the coalition identifies.		3. Ex. Prevention Team and Community Coordinator
**How could we promote access/impact among diverse populations?**	Ex. Provide all patient-facing materials in Spanish, English, and any other languages deemed necessary; Prioritize County FQHC provider	
**How will we know it is working?**	Ex. Providers are willing to meet and listen/engage with continuing education materials; Providers agree to provide/request patient materials for distribution	
**What will make us change our strategy?**	Ex. Providers are not responsive or willing to engage; Educational materials are not reaching patients.	

Appendix 2. Massachusetts Action Planning Tools

Community Profile Summary Form

Purpose: Discuss and Prioritize EBP Strategies that Align with Community Goals

Description: Developing ORCCA-specific Community Goals was a multi-step iterative process driven by community teams (CEFs, CDMs, Community Faculty and CCs). Small group conversations and 1:1s were scheduled with coalition members, local stakeholders, and champions. Community Faculty set the stage with stakeholders by sharing data that demonstrated the impact a combination of initiation on medications for opioid use disorder (MOUD), improvement on MOUD retention, and increasing the distribution of naloxone could have on the HCS communities. These data facilitated conversations about the potential impact of different EBPs. Teams outlined the importance of increasing access to naloxone, MOUD initiation, and MOUD retention within the context of each community. Together, community teams and stakeholders used community profile information to contextualize these data for their community. Community profiles, which included an overview of opioid-related risk, available treatment, and fatal overdose prevention services in the community, were co-created by study staff and community members. The profile, along with local data and coalition insight was used to identify areas of opportunity where ORCCA related strategies could fill unmet needs and/or enhance extant programming. This information was compiled on the community profile summary form.

Who completes the tool: This was an iterative process that involved community teams and subcommittees soliciting feedback from coalition members, dialoging, vetting ideas and ranking solutions as they grappled with how to maximize impact among high-risk populations.

Massachusetts HCS Wave 1 Community Profile Summary

Community Profile Summary **Coalition: _______________**

ORCCA Interventions	Existing Interventions (currently taking place)	Who are the service providers?	High-risk Populations (currently receiving services)	Existing programmatic collaborations (referrals and partnerships)	Areas of opportunity to improve existing services (to identify gaps)	Sustainability Include: Current funding sources and duration	Community Narratives
**Objective 1: Increase Opioid Overdose Prevention Education and Naloxone Distribution (OEND)**							
Required OEND							
Optional OEND							
Naloxone administration (optional)							
**Objective 2: Outreach and Delivery of Medications for Opioid Use Disorder (MOUD) to High-Risk Populations***							
Expand MOUD treatment (required)							
Linkage to MOUD (required)							
Engagement and retention (required)							
**Objective 3: Improve Prescription Opioid Safety**							
Safer Prescribing/ Dispensing (required)							
Safer Disposal Practices (optional)							

*For Definitions of "high-risk populations" see the Section II of the ORCCA Overview

Action Plan Summary Form

Purpose: To comprehensively outline strategies and determine their fiscal feasibility.

Description: Once the community profile summary was complete, community teams and coalitions began the process of selecting specific evidence-based practices. This involved participating in workshops and trainings to learn more about evidenced based strategies supported by the study, conducting 1:1s to engage potential implementation partners through coalition contacts, and assessing the extent to which high-risk populations and venues were engaged with the coalition and conducting outreach. Subgroups engaged in focused conversations exploring potential evidence-based practices using knowledge from previous conversations about evidence-based practices, as well as their feasibility, impact, and sustainability.

Community partners created budgets and estimates that were included later in proposals for action plan items. The action plan summary information was then discussed in subgroup meetings. During subgroup meetings coalition members and partner organizations further flushed out details of their plans refining them for presentation to the larger coalition. At the coalition meeting action plan summaries were discussed and voted on by the coalition membership using decision-making procedures such as consensus or majority voting as outlined in their respective coalition charters.

Who completes the tool: This was an iterative process that involved community teams, subcommittees, and partner organizations.

Massachusetts HCS Wave 1 Community Profile Summary & Action Plan Template

Massachusetts Action Plan Summary **Coalition: XXX**

	Selected Strategies	ORCCA Goal (How it advances the overall HCS goal?)	Targeted Populations	Primary High-Risk Sector			Specific Activities Needed to Implement Selected Strategy: (who, what, when, where)	TA Needed	HCS Funds Needed
				BH	CJ	HC			
**ORCCA Objective 2: Outreach and Delivery of MOUD to High-Risk Populations**									
MOUD Linkage (required)	H. Linkage program via embedding an "in-reach worker" at XXX County Correctional Facility	Through the addition of this staff member, inmates leaving the facility will be provided with naloxone and a MOUD treatment appointment, with transportation provided, on release date.<br><br>Worker will be responsible for: creating relationships with inmates, running harm reduction groups, distributing Narcan, making community connections, scheduling same day appointments, scheduling transportation as needed.	People with reduced tolerance post-incarceration		X		X, Y, and Z orgs will work collaboratively to create a 30-40 hour/week position to fill gap between leaving incarceration and returning home in order to support recovery and lower risk of overdose after incarceration. Facility will determine number of residents released to determine number of hours for staff to spend in the facility.<br><br>Representatives from all 3 organizations will create job description and conduct interview to ensure right fit to work with incarcerated individuals. Qualifications will include knowledge of harm reduction principals, MOUD, OEND, and local services and treatment programs for referrals and connections.<br><br>Worker will ensure Narcan kits are given to inmates upon release (8:30am everyday). Worker will assist in activating inmates MA Health and secure appointments with outside service providers to bridge MOUD treatment post-release. Worker will be deputized as TPP concierge (see below) to have access to schedule rides for inmates being released directly to MOUD appointments in the community.	Group facilitator training<br><br>BAMSI and/or HCS to provide harm reduction training.<br><br>Praxis Narcan Train the Trainer<br><br>Facilitation training	Fulltime Salaried Position for 18 months: Payroll/Fringe $74,344.31<br><br>Mileage $855.00<br><br>Phone/Computer/Supplies/PPE $1,800<br><br>Total Direct Cost: $76,999.31<br><br>Admin Fee: $7,699.31<br><br>**TOTAL COST: $84,699.24**

Action Plan Review Form

Purpose: To assess action plan adherence to the ORCCA menu, impact, inclusion of high-risk populations/venues, feasibility and resources.

Description: Community-specific plans approved by the coalition memberships were then reviewed by HCS MA Leadership and State partners from the department of public health using a standard review form assessing: ORCCA adherence, Impact, Inclusion of High-Risk Populations/Venues, Feasibility and Resources. Written feedback was provided to community teams who then discussed feedback with their coalitions. Leadership met with community teams and coalitions as needed to support with plan clarification or revision. Revised plans were resubmitted for approval by HCS MA Core leaders and state partners.

Who completes the tool: HCS MA Leadership and State partners from the department of public health.

For peer review only

Community:

Please comment below about the extent to which the plan meets the designated criteria, including strengths, challenges and modifications.

Approval Criteria	OEND Plan	MOUD Plan	Safer Prescribing Plan
**ORCCA Adherence**<br>☐ Within the scope of the ORCCA menu<br>☐ Aligned with the overall goal of HCS			
**Impact**<br>☐ Extent to which plan will:<br>o Increase # POUD on MOUD<br>o Retain POUD in care<br>o Increase OEND to POUD at highest risk<br>o Address gaps in the continuum of care<br>o Be sustainable			
**High-Risk Population/Venues**<br>☐ Extent to which plan will:<br>o Include efforts to engage with people who have been historically marginalized<br>o Prioritize populations at heightened risk for overdose - BH/CJ/HC (i.e. prior overdose, decreased tolerance, multi-substance use, concomitant medical or mental illness, homeless, non-English speakers, minority populations)<br>o Mitigate and not exacerbate underlying racial inequities<br>o Prevent unintended consequences			
**Feasibility**<br>☐ Extent to which plan is:<br>o Feasible to implement by January 1, 2021<br>o Clear on what will be done by who and by when to bring about change<br>☐ Extent to which structural or regulatory challenges act as a barrier for this plan			
**Resources**<br>☐ Plan is within budget			

Appendix 3. New York Action Planning Tools

Community Evidence Based Practice Goals

Purpose: To define community level goals for EBPs

Description: The starting point for action planning is reflection and discussion by the coalition on the Community Profile, Data Dashboard, and locally sourced data summarized in a Discussion Tool from Phase 3. This is followed by development of community goals for each ORCCA EBP. Coalitions were asked to reflect on the success of COVID-19 influenced fast tracked strategies from earlier phases and to consider expansion and sustainability of these strategies, as well as to use a health and racial equity lens in the development of community Specific, Measurable, Attainable, Realistic and Time-framed (SMART) goals for each EBP.

Who completes the tool: The community-based implementation team captures discussions and decisions from Coalition members starting in workgroups with their champions by ORCCA Menu and further facilitated in the larger coalition.

DIRECTIONS: Setting ORCCA Goals
The Implementation Team facilitates a discussion with the Coalition (Steering Committee) to identify ORCCA goals based on the recommendations of the ORCCA, Communication and Data Workgroups.

These will be community-level goals to provide OEND in high risk populations, provide outreach and delivery of MOUD to high populations, and provide for safer opioid prescribing and dispensing.

ORCCA Goals

Providing OEND in High Risk Populations	Ex. In 12 months expand OEND through four strategies: expanding peer navigation, expand virtual training, using a variety of distribution strategies, and expanding the number, integration and reach of community trainers by 50%.
Outreach and Delivery of MOUD to High-Risk Populations	Ex. In 9 months, expand access to MOUD by increasing the number of waivered MOUD prescribers and treatment slots; improving prescribing rates of current waivered prescribers; create and sustain a prescriber advisory board to support best practice; and enhancing care coordination, outreach and linkage to enhance engagement and retention in care. Use a data feedback system to improve coordination across providers and service systems and address SDH through a targeted response to increase MOUD retention.
Safer Opioid Prescribing and Dispensing	Ex. Improve safer opioid prescribing and dispensing practices by engaging prescribers and pharmacies to provide resources on safer prescribing, tapering practices, alternative pain management and promoting safer opioid disposal practices between 6-12 months from the time of implementation.

Action Planning Worksheet

Purpose: To identify EBP strategies, venues and priority populations in an iterative process using community profiles, and data identifying needs, gaps and opportunities

Description: Workgroups brainstormed possible ORCCA EBP strategies and communication messages following training and practice using the ORCCA and technical assistance guide (TAG) that could fill existing gaps with identified populations, sectors and venues discussed in Phase 3. ORCCA EBP strategies were considered that could strengthen ongoing community efforts, create opportunities for expansion and efficiency, and promote synergistic approaches to new and existing community strategies. These strategies were presented to the coalition for consideration.

Who completes the tool: Coalition members, workgroups led by champions from each menu and facilitated by community implementation teams

Table 1: Discussion Tool for STEP 1 (CTH PHASE 4)

Strategy	Description & Approach to Population Access (High Risk/Venue) Briefly describe each specific strategy being considered. For strategy, identify populations of focus and venues for reaching them.		STRATEGY	Pros and Cons of each Strategy
	**High Risk Populations** Prior Opioid Overdose; Reduced Opioid Tolerance; Other Substance Use; Co-occurring mental health; Co-occurring physical health; Injection Drugs	**Venues** Harm Reduction/Syringe Service; Health Care; Criminal Justice; First Responder; Addiction Treatment & Recovery; Behavioral Health Treatment; Community Based -Social Service Hotline	**Label each strategy:** Scale up; New Population; New Feature; New Service; New Service & Population	
**OEND in High Risk Populations**				
Active OEND (required)	*EX: Distribute naloxone to released inmates at County Jail or other setting*		EX: scale up or new service	
Passive OEND (optional)	*EX: Distribute naloxone at pharmacy or dispensing program*		EX: New feature	
Naloxone administration strategies (optional)	*EX: First responder naloxone administration*		EX: scale up	
**Outreach and Delivery of MOUD to High-Risk Populations**				
Bridging MOUD (required)	*EX: bridge program for people returning to community from county jail*		EX: new service	
Linkage using Peer Navigator (required)	*EX: Linkage to peer navigation for patients who overdose in ED*		EX: new feature	
Engage and retain individuals in treatment (required)	*EX: telemedicine, virtual appointment*		EX: new feature	
**Safer Opioid Prescribing and Dispensing**				
Safer opioid prescribing/dispensing practices (required)	*EX: Emergency/urgent care*		EX: scaling up	
Safer opioid disposal practices (optional)	*EX: • Prescription drug drop-box/mail-back programs*		EX: scale up or new service	

An additional tool used to make decisions about strategies and their potential for synergy and impact, was agent-based and systems dynamic modeling. Feedback reports using this modeling were introduced to County Implementation Teams and workgroups alongside community profiles to highlight the importance of OEND and MOUD expansion specifically, in order to meet the intervention's opioid overdose prevention goals (see Figure 3), and to inform the prioritization, adaptation, maintenance and sustainability of effective interventions over time for action planning. The systems perspective is intended to be integrated into problem-solving and feedback during ongoing implementation to improve coordination and resource sharing within community networks.

For peer review only - http://bmjopen.bmj.com/site/about/guidelines.xhtml

Figure 3-NY. Systems Feedback Reports for Planning

Systems Mapping for Action Planning

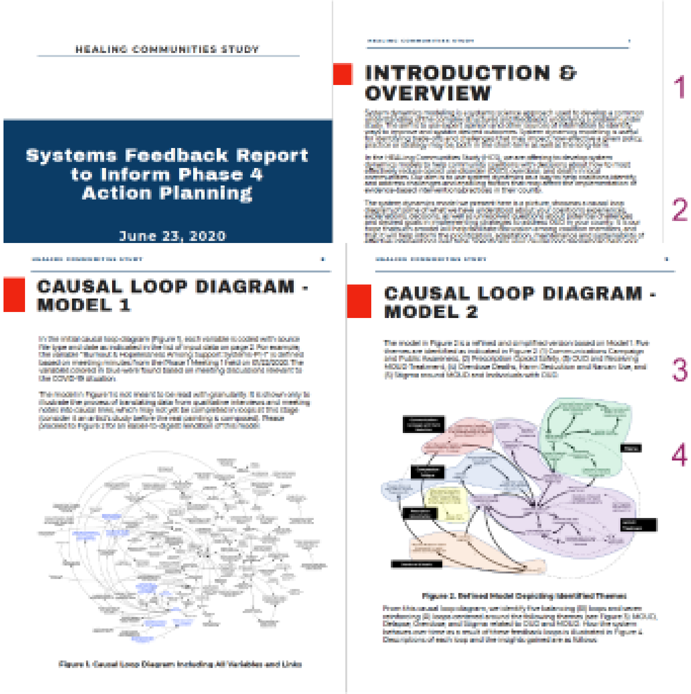

1. Are OEND and MOUD strategies effectively linked to reduce overdose and fatalities?
2. Are there supplemental interventions to support patient engagement, retention and long-term recovery?
3. How is the challenge of stigma recognized and addressed?
4. How are social determinants of health (e.g., homelessness) recognized and addressed?

5

For peer review only - http://bmjopen.bmj.com/site/about/guidelines.xhtml

Consultation and Coaching Plan

Purpose: To provide coaching and feedback to assure adherence to HCS required EBP selections, and enhance capacity of local teams for community planning

Description: The research study team, including implementation coaches assigned to each community, offered iterative support as Community Implementation Teams and workgroups ranked strategy options based on earlier discussion of impact and feasibility determinations in workgroups, finalized strategy selections, and refined strategies into specific SMART goals. The ranking and strategy selection process was not uniform across coalitions. While no limits were placed on the number of strategies selected, attention was given to ensure the 5 required strategies defined in the ORCCA were present or a justification for why not were included. Opportunities were highlighted during coaching sessions to reinforce data driven justification for strategy selection and to feature the role of EBP workgroup champions in leading planning.

Who completes the tool: The Study Team reviews the Action Planning Worksheet with Summary Action Plans with the Implementation Team in coaching sessions using the criteria below:

ORCCA Goals	Prioritized EBPs	Draft CAP	Adopted CAP
Goals are aligned with HCS goal to reduce opioid overdose mortality by 40%	Priorities are aligned with the ORCCA-specific goals	CAP summarizes coalition goals	Summarizes coalition goals
Goals target identified gaps in the provision of OEND, MOUD, and safer opioid prescribing and dispensing	Priorities address the gaps documented in Community Profile	CAP summarizes selected strategies	Summarizes selected strategies, venues and populations
	Workgroup prioritized strategies as high impact	CAP identifies the team responsible for implementing the strategies	Identifies the team responsible for implementation planning and organizations for initial engagement
	Workgroup prioritized strategies as high feasibility	CAP identifies and the timeframe for implementation planning	Identifies the timeframe for implementation planning
		To what degree have considered health equity and racial disparities?	Have considered health equity and racial disparities
		Initial TA and resources needed, including plans to use Community Impact (CI) Funds	TA and resources needed, including plans to use CI Funds

For peer review only

SCHEDULING REVIEW AND CONSULTATION

Implementation Teams should upload and maintain documentation for each deliverable (ORCCA Goals, prioritized EBPs and the Community Action Plan) on the Public HCS County Folders drive. Project Managers should notify the Study Team by email to request review and coaching sessions. Please use the following timetable:

Deliverable	When to Request Review	Review Type	Target Deadline	Response Time
**ORCCA Goals**	Workgroup and Coalition consensus on ORCCA goals	Review and comment	**00/00/0000**	2 Business Days
**Prioritized EBPs Draft Community Action Plan**	Prioritized EPBs and draft Community Action Plan is ready for recommendation the Coalition	Coaching Meeting	At least 1 week prior to Coalition Meeting at which EBPs and AP will be considered	2 Business Days
**MOUD and Safer Rx EBP, AP**	Consulting addiction psychiatrists will also provide consultation and review of the prioritized EBPs and draft Community Action Plan for MOUD and Safer Prescribing Workgroups during Workgroup meetings.			
**Approved Community Action Plan**	Coalition has approved the Community Action Plan	Review and comment	Next business day after Coalition approval	2 Business Days

The Implementation Teams can invite the Study Team to join the Workgroup meeting in which EBP strategies are being prioritized to provide review and consultation, if that is the preference of the Workgroup, Champion and Implementation Team.

For peer review only

Summary Community Action Plan Form

Purpose: To provide a summary of EBPs and community goals, venues and priority populations with initial organizations, and first steps to be the focus for implementation planning in Phase 5.

Description: The final step in the Phase 4 Action Planning Worksheet is a summary community action plan for final coalition approval guided by the coalition's charter on decision-making. Most used a majority vote in a coalition meeting. The plan includes defined SMART goals with an outcome target for each ORCCA EBP-related strategy and identification of specific populations, sector for implementation (i.e., behavioral health, criminal justice, healthcare), reference to feasibility and impact rankings from workgroup discussions, and preliminary sustainability. Additionally, in anticipation of Phase 5 Implementation Plan Agreement development, the action plan includes recommended initial and anticipated steps for engaging community partners and estimates of technical assistance and resources.

Who completes the tool: Community implementation teams led by a program manager and CEF complete the summary and present to the coalition for adoption. In some cases a county executive or leadership team provides an additional level of approval.

TABLE 6: Action Plan Table (CTH PHASE 4)

Strategy Category	Selected Strategies	Targeted Populations	High-Risk Sector			Sustainability Feasibility/ Impact	Implementation Steps: who does what, by when and where	Technical Assistance & Other Resources
			BH	CJ	HC			
**ORCCA Objective 1: Increase Opioid Overdose Prevention Education and Naloxone Distribution**								
Active OEND (required)	A1:<br>A2:							
Passive OEND (optional)	B1:<br>B2:							
Naloxone administration (optional)	C1:<br>C2:							
**ORCCA Objective 2: Outreach and Delivery of MOUD to High-Risk Populations**								
Expand MOUD treatment (required)	D1:<br>D2:							
Linkage to MOUD (required)	E1:<br>E2:							
Engagement and retention (required)	F1:<br>F2:							
**ORCCA Objective 3: Improve Prescription Opioid Safety**								
Safer Prescribing/ Dispensing (required)	G1:<br>G2:							
Safer Disposal Practices (optional)	H1:<br>H2:							

Appendix 4. Ohio Action Planning Tools

Decision Aid 1 Matrix

Purpose: To identify existing venues related to ORCCA EBPs

Description: In Ohio, the HCS Intervention Facilitator, one field team (i.e., an HCS-hired Intervention Facilitator, Community Engagement Facilitator, Data Manager) worked with community coalitions, particularly coalition-identified EBP champions to utilize a multi-step Decision Aid Approach across Phase 4 study activities. The first step was to identify existing venues related to ORCCA EBPs. In Step 1: Cataloging Possible Approaches to Population Access, a matrix was used in Decision Aid 1 to map existing locations/methods of current *or potential* population access to priority populations. This allowed communities to inventory potential points of access and also highlighted options that were not available or are already highly represented.

Who completes the tool: Decision Aid 1 is completed by ORCCA EBP coalition champions in collaboration with HCS-hired staff.

**Community "A": Decision Aid 1**		**Points of Population Access**		
		**CJ**	**SSPs**	**EDs**
**Priority Populations**	**Prior Opioid Overdose**	ABC drug court<br>XYZ County Jail	Abc street exchange	None
	**Reduced Opioid Tolerance**	ABC drug court<br>XYZ County Jail		None
	**Use Other Substances**	ABC drug court<br>XYZ County Jail	Abc street exchange	None

For peer review only

Decision Aid 2

Purpose: To identify preliminary ORCCA-related goals for each of the three EBPs.

Description: In Step 2: Existing Service Inventory Mapped by Priority Population and Point of Population Access, a similar approach to Decision Aid 1 was employed, but limited to listing *currently available* services (i.e., interventions) specific to each of the three EBPs required by ORCCA. As a first step, a community would use their population access Decision Aid 1 from Step 1 to highlight any setting in which there was a current intervention related to the three ORCCA required objectives. Next, each community would catalog any existing services that address the three ORCCA required objectives using Decision Aids 2A (OEND), 2B (MOUD), and 2C (Safer Prescribing). This approach documents the gaps between potential methods for population access and the degree to which those were already being implemented.

Who completes the tool: Decision Aid 2 is completed by ORCCA EBP coalition champions in collaboration with HCS-hired staff.

**Community "A"- Decision Aid 2B: MOUD**		**Points of Population Access**		
		**CJ**	**SSPs**	**EDs**
**Priority Populations**	**Prior Opioid Overdose**	None	Abc Syringe Exchange-treatment referral	None
	**Reduced Opioid Tolerance**	None	None	None
	**Use Other Substances**	None	Abc Syringe Exchange-treatment referral	None

For peer review only

Decision Aid 3

Purpose: To identify, prioritize, and select EBP strategies.

Description: In Step 3 of the Decision Aid process, communities in collaboration with HCS *brainstormed potential intervention strategy and population access dyads*. This approach allowed for a shared understanding of "what was currently being done" and "where it was being done" in an efficient manner that mapped structurally to ORCCA priority populations and methods of population access. The Decision Aid was not menu-specific, but rather covered strategies for intervention across menus. This provided the foundation for brainstorming which ORCCA EBP strategies *might target existing gaps.* In this step, there is an emphasis on feasibility of implementation.

Discussion Aid 3 was employed in Step 3 and prompted communities to consider whether the identified options involved:

a. bringing an existing service to scale ("1")
b. disseminate an existing service to a new population or method of population access ("2")
c. add a new feature or improvement to an existing service (i.e., changing passive referral to formal linkage intervention); ("3")
d. add a new service; ("4")
e. add both a new method of population access and a new service (e.g., a syringe service program with naloxone distribution); ("5").

Who completes the tool: Decision Aid 3 is completed by ORCCA EBP coalition champions in collaboration with HCS-hired staff.

**Community A-Decision Aid 3: Possible Strategies for Intervention—*no priority order***		**Approach to Population Access**	**Category of Implementation (1-5 above)**
**1**	Distribute OEND	XYZ County Jail	1: bring to scale
**2**	Bridging MOUD	XYZ County Jail	4: new program
**3**	Linkage to Care-Mandated Treatment	ABC Drug Court	1: bring to scale
**4**	Linkage using Peer Navigator	XYZ County Jail	4: new program
**5**	Linkage using Peer Navigator	SSP	3/4: modifying referral program/new program

For peer review only

Decision Aid 4

Purpose: To prioritize EBP strategies by feasibility and expected impact.

Description: This step involved consideration of the intersection between the importance of a given plan and the degree of difficulty in implementing that plan with the notion that EBPs that were both *highly impactful* and *highly feasible* should be undertaken before other actions that are of lower impact and/or lower feasibility. In Decision Aid 4, the identified strategies were categorized by priority/importance (low, medium, high) and by feasibility/capacity (low, medium, high)

Who completes the tool: Decision Aid 4 is completed by ORCCA EBP coalition champions in collaboration with HCS-hired staff.

**Community A- Decision Aid 4: Possible Strategies for Intervention—*feasibility/priority***		**Feasibility/Capacity**		
		Low	Medium	High
**Priority/Importance**	Low	• Linkage to Care Mandated Treatment at ABC Drug Court (*i.e., already at scale with little opportunity to expand*)	• Linkage using peer-navigators at XYZ county Jail (i.e. may move to high priority if bridging MOUD is unsuccessful)	
	Medium		• Distribute OEND at XYZ county jail	
	High	• Bridging MOUD at XYZ county jail	•	• Linkage using peer-navigators at SSP

For peer review only

Decision Aid 5

Purpose: To prioritize and select EBP strategies.

Description: In Step 5: Prioritizing and Selecting EBPs, communities ranked the options outlined in Step 3 in priority order based on the feasibility and impact determinations of Step 4. Coalitions were encouraged to prioritize options that were high impact and high feasibility over interventions that were of low impact or feasibility. Of note, the coalition was not limited in the number of strategies they could target for implementation. This determination was encouraged to be paired with the decision on whether the community and HCS team would pursue a given option for implementation.

Utilizing Step 5 of the Decision Aid process, communities then create their initial action plan specific to their ORCCA goals and the required ORCCA EBPs. The action plan includes the specific strategies selected, the targeted sectors for the intervention, the identified practice settings or venues, preliminary action plans, potential technical assistance or other resource needs, the individual within the Ohio Intervention Design Team (IDT), comprised of academic and subject experts, who will be primarily overseeing and assisting with the implementation of the identified strategy, and additional notes regarding the strategy. For example, if an EBP were to be conducted in an Emergency Department setting, the Emergency Department IDT would oversee the implementation of that EBP and provision of technical assistance.

Who completes the tool: Decision Aid 5 and the Action Plan is completed by ORCCA EBP coalition champions and community coalitions in collaboration with HCS-hired staff.

**Community A-Decision Aid 5: Selected Strategies for Intervention—*priority order***		**Approach to Population Access**	**Target for Action**	**Notes/Preliminary Action Plan**
**1**	Linkage using Peer Navigator	SSP	Yes	Hire program coordinator; select peers
**2**	Bridging MOUD	XYZ County Jail	Yes	
**3**	Distribute OEND	XYZ County Jail	Yes	
**3**	Linkage to Care-Mandated Treatment	ABC Drug Court	No	
**4**	Linkage using Peer Navigator	XYZ County Jail	No	Revisit in 3 months pending exploration of bridging MOUD at XYZ County Jail

For peer review only

Action Plan Template and Sample Completed Action Plan

County: ______________________

Completed By: ___ ______
Initial Completion Date: _____/____/______
Revision #: _____________
Revision Completion Date: _____/________/________

	Specific Strategy	Approach to Population Access (Sector)	Practice Setting or Venue Name	Preliminary Action Plan	Potential TA/Resource Needs	State IDT Lead	Notes
**ORCCA Objective 1: Increase Opioid Overdose Prevention Education and Naloxone Distribution**							
Active OEND (Required)							
Passive OEND (optional)							
**ORCCA Objective 2: Outreach and Delivery of MOUD to High-Risk Populations**							
Expand MOUD treatment (required)							
Linkage to MOUD (required)							
Engagement and retention (required)							
**ORCCA Objective 3: Improve Prescription Opioid Safety**							
Safer Prescribing/ Dispensing (required)							
Safer Disposal Practices (optional)							

Strategy category	Specific Strategy	Approach to Population Access (Sector)	Practice Setting or Venue Name	Preliminary Action Plan	Potential TA/Resource Needs	State IDT Lead	Notes
**ORCCA Objective 1: Increase Opioid Overdose Prevention Education and Naloxone Distribution**							
**Active OEND (required)**	**Active OEND for at-risk individuals and their social networks**<br><br>*Expansion of Project DAWN (a program to provide naloxone to Ohio agencies) to a secondary site*	Behavioral Health	Church	Confirm partner agencies. (HCS)<br><br>Assemble Intervention Design Team/Operations Team to provide implementation assistance. (HCS)	Resources needed on technology/ data collection, billing for sustainability.	[NAME]	Date approved by coalition: DATE
**Active OEND (required)**	**Active OEND for at-risk individuals and their social networks**<br><br>*Expansion of Project DAWN to a secondary site*	Healthcare	[AGENCY] Mobile Unit	Confirm partner agencies. (HCS)<br><br>Assemble Intervention Design Team/Operations Team to provide implementation assistance. (HCS)	Resources needed on technology/ data collection, billing for sustainability.	[NAME]	Date approved by coalition: DATE
**Active OEND (required)**	**Active OEND at high-risk venues:**<br><br>*Naloxone Referral/ Distribution for Drug Court Participants*	Criminal Justice	Drug Court	Confirm partner agencies. (HCS)<br><br>Assemble Intervention Design Team/Operations Team to provide implementation assistance. (HCS)	Potential resources needed on increasing access and participant engagement.	[NAME]	Date approved by coalition: DATE
**Active OEND (required)**	**Active OEND at high-risk venues: "Leave behind"**	Healthcare	Fire/EMS	Confirm partner agencies. (HCS)<br><br>Assemble Intervention Design Team/Operations	Potential resources needed on policy/ implementation logistics, streamlined data collection for naloxone use.	[NAME]	Date approved by

For peer review only

Strategy category	Specific Strategy	Approach to Population Access (Sector)	Practice Setting or Venue Name	Preliminary Action Plan	Potential TA/Resource Needs	State IDT Lead	Notes
	**programs at sites of overdose**<br><br>*Fire/EMS Leave-Behind Program*			Team to provide implementation assistance. (HCS)			coalition: DATE
**OEND self-request (e.g. at pharmacy, community meeting or public health department)**	**Passive OEND**<br><br>Naloxone Distribution at Pharmacies in high risk areas	Healthcare	Pharmacy	Confirm partner agencies. (HCS)<br><br>Assemble Intervention Design Team/Operations Team to provide implementation assistance. (HCS)	Potential resources needed on logistics and possible policy development.	[NAME]	Date approved by coalition: DATE
**ORCCA Objective 2: Outreach and Delivery of MOUD to High-Risk Populations**							
**Expand MOUD treatment (required)**	**Adding/expanding MOUD treatment in Criminal Justice settings**<br><br>*MOUD in the Jail*	Criminal Justice	Jail	Confirm partner agencies. (HCS)<br><br>Assemble Intervention Design Team/Operations Team to provide implementation assistance. (HCS)	Potential resources needed on logistics, billing, etc.	[NAME]	Date approved by coalition: DATE
**Linkage to MOUD**<br><br>**(required)**	**Linkage Programs (all relevant settings)**<br><br>*Expand MOUD referral in ED*	Healthcare	Emergency Department	Confirm partner agencies. (HCS)<br><br>Identify opportunities for expansion of current services.<br><br>Assemble Intervention Design Team/Operations Team to provide implementation assistance. (HCS)	Potential resources needed on sustainability and patient engagement.	[NAME]	Date approved by coalition: DATE

For peer review only

Strategy category	Specific Strategy	Approach to Population Access (Sector)	Practice Setting or Venue Name	Preliminary Action Plan	Potential TA/Resource Needs	State IDT Lead	Notes
**Engagement and retention (required)**	**Reducing barriers to housing, transportation, childcare and accessing other community benefits for people with OUD**<br><br>*Reduce barriers to transportation to improve engagement/retention in treatment*	Behavioral Health	MOUD Treatment Facility<br><br>Potentially other MOUD treatment facilities with limited transportation options. (TBD)	Confirm partner agencies. (HCS)<br><br>Assemble Intervention Design Team/Operations Team to provide implementation assistance. (HCS)<br><br>Engage partners and IDT through an initial planning meeting. (HCS)	Potential resources needed on billing for sustainability.<br><br>Needs: Identify potential partnerships between XXX transportation and treatment agencies	[NAME]	Date approved by coalition: DATE
**ORCCA Objective 3: Improve Prescription Opioid Safety**							
**Safer Prescribing/ Dispensing (required)**	**Safer opioid prescribing for chronic pain**<br><br>Prescriber Accountability through OD Review	Healthcare	Pharmacy	Confirm partner agencies. (HCS)<br><br>Assemble Intervention Design Team/Operations Team to provide implementation assistance. (HCS)<br><br>Engage partners and IDT through an initial planning meeting. (HCS)	Potential resources needed on logistics and identifying allowable information to be shared between the XXX team and XXX Agency.	[NAME]	Date approved by coalition: DATE
**Safer Prescribing/ Dispensing (required)**	**Safer opioid dispensing**<br><br>Utilize the Take Charge Ohio Toolkit for pharmacist and patient education. (Co-dispensing naloxone, providing educational materials	Healthcare	Pharmacy	Engage Education and Awareness workgroup of Scioto County Collaborative Opioid Consortium.<br><br>Determine which parts of the Toolkit will be utilized.<br><br>Confirm partner agencies. (HCS)	Potential resources needed on logistics and possible policy development.	[NAME]	Date approved by coalition: DATE

For peer review only

Strategy category	Specific Strategy	Approach to Population Access (Sector)	Practice Setting or Venue Name	Preliminary Action Plan	Potential TA/Resource Needs	State IDT Lead	Notes
	on opioids and naloxone)			Assemble Intervention Design Team/Operations Team to provide implementation assistance. (HCS)			

For peer review only

BMJ Open

Community-driven selection of strategies to implement evidence-based practices to reduce opioid overdoses in the HEALing Communities Study, a multi-site, parallel-group, cluster randomized wait-list controlled trial in Kentucky, Massachusetts, New York, and Ohio

Journal:	*BMJ Open*
Manuscript ID	bmjopen-2021-059328.R1
Article Type:	Protocol
Date Submitted by the Author:	16-Jun-2022

Complete List of Authors:	Young, April; University of Kentucky, Epidemiology<br>Brown, Jennifer; University of Cincinnati<br>Hunt, Timothy; Columbia University<br>Sprague Martinez, Linda; Boston University<br>Chandler, Redonna; National Institutes of Health<br>Oga, Emmanuel; Research Triangle Institute<br>Winhusen, Theresa ; University of Cincinnati Medical Center, Psychiatry<br>Baker, Trevor; Boston Medical Center<br>Battaglia, Tracy; Boston Medical Center<br>Bowers-Sword, Rachel; Boston Medical Center<br>Button, Amy; Albert Einstein College of Medicine, Montefiore Hudson Valley Collaborative<br>Fallin-Bennett, Amanda ; University of Kentucky, College of Nursing<br>Fanucchi, Laura; University of Kentucky<br>Freeman, Patricia ; University of Kentucky College of Pharmacy<br>Glasgow, LaShawn; Research Triangle Institute<br>Gulley, Jennifer; Clark County Health Department<br>Kendell, Charles; Franklin County Agency for Substance Abuse Policy Board<br>Lofwall , Michelle ; University of Kentucky, Center on Drug and Alcohol Research<br>Lyons, Michael S.; Cincinnati Children's Hospital Medical Center<br>Quinn, Maria; Holyoke Medical Center<br>Rapkin, Bruce; Albert Einstein College of Medicine, Epiemiology and Population Health<br>Surratt, Hilary; University of Kentucky<br>Walsh, Sharon ; University of Kentucky

**Primary Subject Heading**:	Addiction
Secondary Subject Heading:	Addiction, Evidence based practice, Public health, Research methods
Keywords:	Substance misuse < PSYCHIATRY, PUBLIC HEALTH, STATISTICS & RESEARCH METHODS

SCHOLARONE™
Manuscripts

Community-driven selection of strategies to implement evidence-based practices to reduce opioid overdoses in the HEALing Communities Study, a multi-site, parallel-group, cluster randomized wait-list controlled trial in Kentucky, Massachusetts, New York, and Ohio

Young AM, Brown JL, Hunt T, Sprague Martinez LS, Chandler R, Oga E, Winhusen TJ, Baker T, Battaglia TA, Bowers-Sword R, Button A, Fallin-Bennett A, Fanucchi L, Freeman PR, Glasgow LM, Gulley J, Kendell C, Lofwall MR, Lyons MS, Quinn M, Rapkin B, Surratt H, Walsh S.L.

Corresponding Lead Author: April M Young (april.young@uky.edu)

For peer review only

Abstract

Introduction: Opioid-involved overdose deaths continue to surge in many communities, despite numerous evidence-based practices (EBPs) that exist to prevent them. The HEALing Communities Study (HCS) was launched to develop and test an intervention (i.e., Communities That HEAL [CTH]) that supports communities in expanding uptake of EBPs to reduce opioid-involved overdose deaths. This paper describes a protocol for a process foundational to the CTH intervention through which community coalitions select strategies to implement EBPs locally.

Methods and analysis: The CTH is being implemented in 34 communities (randomized to receive the intervention) in four states in partnership with coalitions (one per community). Coalitions must select at least five strategies, including one to implement each of the following EBPs: (a) overdose education and naloxone distribution; expanded (b) access to medications for opioid use disorder [MOUD], (c) linkage to MOUD, (d) retention in MOUD; and (e) safer opioid prescribing/dispensing. Facilitated by decision aid tools, the community action planning process includes (1) data-driven goal setting, (2) discussion and prioritization of EBP strategies, (3) selection of EBP strategies, and (4) identification of next steps. Following review of epidemiologic data and information on existing local services, coalitions set goals, and discuss, score, and/or rank EBP strategies based on feasibility, appropriateness within the community context, and potential impact on reducing opioid-involved overdose deaths with a focus on three key sectors (health care, behavioral health and criminal justice) and high-risk/vulnerable populations. Coalitions then select EBP strategies through consensus or majority vote and, subsequently, suggest or choose agencies with which to partner for implementation.

Ethics and dissemination. The HCS protocol was approved by a central IRB (Advarra, Inc) and is registered with Clinical Trials.gov (NCT04111939). Results of the action planning process will be disseminated in academic conferences and peer-reviewed journals, online and print media, and in meetings with community stakeholders.

Strengths and limitations of this study

- The CTH action planning protocol balances scientific rigor with community priorities by ensuring all communities follow a common core of structured activities and phased benchmarks, select from a common menu of strategies for implementing EBPs, and discuss similar considerations as they create their action plan, while also allowing variation across states and communities to best suit the local context.
- The methods used in the CTH action planning process elevates the voices of community members, whose insights about the local community context are critical to implementation success and whose involvement conveys to implementing agencies and local decision-makers that EBP implementation is a priority of the local community and constituents rather than of an outside (federal or academic) entity.
- The action planning protocol requires careful facilitation by coalition members and community staff to ensure that power dynamics do not result in some coalition members having more influence than others in the decision-making process and to manage conflicts of interest (i.e., selection of strategies that could benefit or be perceived to benefit individual coalition members or their agencies).
- The phased and iterative action planning protocol is time-intensive requiring significant commitment from involved community members, faculty, and staff, and may slow implementation of EBPs.
- While necessitated by the constraints of the study timeline, the research teams' intensive involvement in facilitating movement through the action planning protocol may stifle participation by some coalition members who approached the process with deference and thereby limited the extent to which it was community-driven.

For peer review only

Introduction

Opioid-involved overdose deaths remain a public health crisis in many communities worldwide[1 2] although numerous evidence-based practices (EBPs) exist to prevent them. In the Unites States alone, opioid-involved overdose claimed over 490,000 lives between 1999 and 2019.[3] Despite national efforts to change the trajectory of overdose deaths, provisional data indicate over 94,000 total overdose deaths occurred in the 12 months ending in January 2021, making this the highest number of overdose deaths ever recorded in a 12-month period.[4]

Research has demonstrated that medication for opioid use disorder (MOUD), including methadone, buprenorphine, and extended-release naltrexone;[(e.g. 5)] prescription drug monitoring programs and safer prescribing guidelines for prescription opioids;[(e.g. 6)] naloxone, a medication that can reverse an opioid overdose;[(e.g.,7)] and behavioral/psychosocial interventions like recovery support services [(e.g. 5)] can prevent deaths and help people enter and stay in remission and recovery from opioid use disorder. Unfortunately, these EBPs remain under-utilized in many communities leaving significant unmet need for these critically important services.[8-15]

The HEALing Communities Study (HCS), supported by the National Institutes of Health and the Substance Abuse and Mental Health Services Administration, launched in 2019 to develop and test an approach to support communities in expanding uptake and availability of EBPs to address opioid-involved overdose deaths and opioid use disorder (OUD). HCS is a multi-site, parallel-group, cluster randomized wait-list controlled trial involving 67 communities (34 in active intervention and 33 in wait-list control) in Kentucky (KY), Massachusetts (MA), New York (NY), and Ohio (OH).[16] The study is designed to test the Communities That HEAL (CTH) intervention, which contains three components: a community-engaged process of data-driven decision making to select EBP strategies for local implementation and monitoring;[17] a

For peer review only

menu of EBPs and technical assistance guides (Opioid-Overdose Reduction Continuum of Care Approach [ORCCA]),[18] and a set of communication campaigns intended to address stigma and increase knowledge of and demand for EBPs.[19] The CTH intervention is being implemented in the active intervention communities from January 2020 to June 2022 and will be implemented in wait-list control communities from July 2022 to June 2023.

The CTH intervention is grounded in the recognition that communities affected by the opioid overdose crisis possess the best understanding of local strengths, gaps, barriers and facilitators related to local EBP implementation and uptake.[17] Thus, the CTH involves partnerships between academic researchers and community members through community coalitions. Coalitions are comprised of representatives from diverse agencies and institutions as well as individuals focused on tackling complex community issues through collective planning.[20] The CTH intervention employs a phased coalition planning process that was developed based on and expands the Communities that Care model,[17] which has supported communities in the selection of evidence-based prevention efforts in past research.[21-23] Coalition planning models are effective because they allow communities to leverage resources and expertise from across multiple sectors, which can facilitate creative problem solving and innovation, reduce duplication of efforts, and address service gaps.[24 25] The literature points to coalition planning as an effective strategy for implementing EBPs.[26-28]

The purpose of this paper is to describe the protocol for coalition action planning through which EBP strategies are selected to reduce opioid overdose deaths in their communities.[16] The protocol described in this manuscript is relevant beyond the field of substance use, as community engagement and community-driven decision making are recommended for improving responses to a variety of health challenges,[29-32] including, most recently, SARS-CoV-2 vaccination.[33]

For peer review only

Methods and Analysis

Participant involvement

As described elsewhere,[17] community engagement is central to the CTH intervention. The CTH intervention places communities, specifically community coalitions, at the center of the decision-making process in creating an action plan for implementing EBP strategies locally. The coalitions are comprised of key stakeholders in healthcare, behavioral health, criminal justice, public health, government, business, housing, and other sectors, and in many cases, include people with lived experience with or impacted by OUD. In some communities, the coalitions that are presently engaged in the CTH existed prior to the HCS, while others were formed specifically to guide the CTH intervention. In addition to driving the CTH intervention, these coalitions facilitate dissemination of information about the study and about EBPs to other local stakeholders. Coalitions, local agency partners, and partnerships formed between academic institutions and local stakeholders will also be key to sustainability of the CTH process and EBPs. Of note, coalitions were not involved in setting the research question, designing the intervention protocol or menu of EBPs, or establishing outcome measures. Of note, each of the four participating states has a Community Advisory Board, with multisectoral representation from across the communities that advises the research teams on study implementation.

Overview of CTH phases

The CTH intervention includes seven phases, described in detail elsewhere,[17] that guide coalitions through a systematic partnership process. The intervention began October 23, 2019

For peer review only

and the estimated study completion date is March 31, 2024. Briefly, the process begins with establishment or identification of a coalition and an environmental scan, or "landscape analysis," of local resources and gaps (Phase 0). During Phase 1, coalitions develop a shared charter and identify key community "champions" to support EBPs, data-driven decision making, and communication campaigns that have been described elsewhere.[19] In Phase 2, coalitions learn about data-driven decision making and the ORCCA menus containing EBP strategies related to overdose education and naloxone distribution (OEND, Menu 1), MOUD (Menu 2), and prescription opioid safety (Menu 3). The ORCCA menus have been described in detail elsewhere.[18] Following review of data describing the local epidemiology of OUD and overdose, simulation modeling data, and/or existing service resources and gaps identified by the landscape analysis (Phase 3), coalitions develop community action plans (one per coalition) by selecting strategies to implement EBPs that are in the ORCCA menu (Phase 4). After creation of the action plan, coalition selected EBP strategies are implemented and monitored (Phase 5) and sustainability plans are created (Phase 6). While these processes are ongoing, a health communication campaign is delivered at the community level to reduce stigma and increase engagement in OEND and MOUD.[19] This paper describes the protocol for coalition planning efforts in Phase 4 to select EBP strategies to achieve the study's primary outcome, reduction in number of opioid overdose deaths. The primary and secondary outcomes of the study and the analysis of the trial data are described in detail elsewhere.[34 35]

Community Action Planning Guidelines

Coalitions are provided with guidelines to structure the community action planning process (Figure 1). Coalitions are required to select a minimum of five strategies, including at

For peer review only

least one to implement each required EBP from the ORCCA: (a) expansion of proactive offering of OEND to at risk individuals and members of their social networks, (b) expansion of buprenorphine and/or methadone access, (c) improved linkage to MOUD, (d) increased retention in MOUD, and (e) promotion of safer opioid prescribing/dispensing. The EBP strategies are required to include at least one to be implemented in each of the following three sectors: health care, behavioral health and criminal justice.[18] Coalitions are encouraged to consider EBP strategies that would maximize impact in key populations most vulnerable to experiencing an opioid-involved overdose, including those who had a prior opioid overdose;[8 36-39] have reduced opioid tolerance (e.g., completing medically supervised or socially managed withdrawal or release from institutional settings such as jail, residential treatment, hospital);[39-43] use other substances (e.g., alcohol, benzodiazepines, cocaine, and amphetamine like substances);[39 44-48] have concomitant major mental[49-52] or medical illness;[39 53-56] and/or inject drugs.[45 57] Coalitions are also asked to prioritize venues through which these key populations are most likely to be reached. Priority venues for each of the three EBPs (i.e., OEND, MOUD, and safer opioid prescribing and dispensing) are listed in Figure 1 and described in detail elsewhere.[18]

Inherently, the CTH allowed for communities to independently implement EBP strategies at their own pace, including expediting strategies in times of emergency. In June 2020, the HCS approved "fast tracking" certain HCS-supported activities related to OEND in response to the coalitions' concerns about the substantial uptick of opioid overdose observed in the initial months of the SARS-CoV-2 pandemic.[58-65] The protocol allowed HCS-supported naloxone distribution to begin in high-risk settings prior to the completion of action planning, but preserved the community-driven nature of the decision-making process. Coalitions were asked whether they wanted the HCS teams to provide technical assistance and/or resources to

For peer review only

community venues with greatest reach to individuals at risk for overdose (i.e., jails, syringe service programs) to expand OEND. Upon coalition approval, HCS teams and/or community implementation teams began outreach to high-risk venues to expand OEND. Thus, the fast-tracked OEND activities were ongoing during the Phase 4 action planning process. During the action planning process, coalitions were asked to reflect on the successes and challenges of the fast-tracked strategies and asked to decide whether they wanted to continue or expand OEND activities that had been fast-tracked as part of the action planning process.

Core components of the action planning process

The core components of the cross-state protocol for action planning are described in Figure 2. Action planning in each state involves (1) data-driven community goal setting, (2) discussion and prioritization of EBP strategies, (3) selection of EBP strategies to include in the action plan, and (4) identification of next steps for implementation of selected EBP strategies.

Coalitions' movement through the action planning process is facilitated by a combination of goal-setting tools and decision aids. Examples of tools developed by each state for the action planning process are provided in the Supplementary Appendix. The communities currently receiving the CTH intervention differ in geographic unit (i.e., counties, townships, cities), community-readiness for EBP strategy implementation, existing resources and gaps, coalition history (e.g., whether coalitions existed prior to the HCS or convened for primarily for the HCS), and other contextual factors thereby requiring some variation to the action planning process across and within states (Table 1).

Action planning in each state is driven by coalitions, coalition workgroups, and champions, and is facilitated by a team comprising clinical and research faculty, core staff, and

For peer review only

community-based staff with expertise in community-engagement, data, OEND, MOUD, and safer opioid prescribing and dispensing. Coalition composition is described briefly in *Participant Involvement* and in detail elsewhere.[17] Of note, only in MA are *all* agencies to eventually be involved in EBP strategy implementation represented on the coalition. In other states, the agencies to be involved in implementation are represented to varying degrees across coalitions. In NY, community government partners (i.e., health commissioners, county executives) are also involved in the action planning process.

Step 1. Develop Data-driven Community Goals

All coalitions begin the goal-setting process by reviewing community profiles and data dashboards, supplemented in one state (NY) by results of agent-based and systems dynamic modeling. The community profiles, developed through the CTH intervention, included lists of organizations in the community relevant to potential implementation of EBP strategies (e.g., substance use disorder treatment centers, harm reduction programs, emergency response agencies, hospitals). The data dashboards, also developed through the CTH intervention, contain data visualizations on community-specific trends in fatal and non-fatal opioid overdose, MOUD availability, naloxone distribution, and high-risk opioid prescribing.[9]

During and following data review, coalitions discuss strengths, services, gaps, and needs. The coalitions established goals varying in specificity across states and communities (e.g., “Increase OEND,” “Two emergency departments will initiate MOUD by January 2021”). Coalitions set goals at the ORCCA menu-level (i.e., for OEND, MOUD, and safer opioid prescribing/dispensing) or submenu-level (i.e., for active and passive OEND; for MOUD expansion, linkage, and retention; for safer opioid prescribing/dispensing and disposal). States

For peer review only

vary in the other factors their coalitions are asked to consider when setting goals; these factors include sustainability of EBP strategies (NY, MA), EBP strategies' reach to high-risk populations (OH, MA), ability to ensure health equity across ethnic and racial groups (MA, NY), and priority venues for EBP strategy implementation (OH, MA).

Step 2. Discuss and Prioritize EBPs that Align with Community Goals

Following goal setting, coalitions consider specific strategies for implementing the EBPs included on the ORCCA menu and determine which are of highest priority for local implementation. In three states (OH, MA, NY), coalitions brainstorm and free-list EBP strategies that align with the study goals, priority sectors and/or venues, and high-risk populations. In KY, coalitions are provided with initial lists of EBP strategies to consider that are pre-populated from the ORCCA by the academic team. Coalitions in all states consider the EBP strategies in terms of the existing services, size of the gap between existing services and need, feasibility, and potential impact on opioid-involved overdose deaths. To frame the discussion of feasibility more concretely, coalitions in two states (OH, NY) are asked to consider whether the EBP strategy is a new service, scale-up of an existing service via a new method of service delivery, new target population, or new feature to an existing service. In two states (MA, NY), coalitions also consider sustainability. In all states, deliberations on the EBP strategies occur in full coalition meetings or in workgroups led by champions depending on the coalition's preference.

All coalitions begin by providing narrative feedback on each EBP strategy. One state's (KY) coalitions are asked to supplement the narrative feedback by scoring each EBP strategy in terms of size of the existing service gap, feasibility of implementation within the next 12 months, and potential impact on opioid-involved overdose deaths using Likert scales. The scale scores are

For peer review only

used to produce a summative priority score to help guide EBP strategy selection. Two states (NY, OH) ask coalitions to rank EBP strategies in terms of impact and feasibility, with the most impactful and feasible EBP strategies scoring highest. In one state (MA), coalitions prioritize EBP strategies through consensus building group dialogues as opposed to ranking.

In three states, coalitions are either required (MA) or encouraged to consider the cost of EBP strategy implementation (NY, OH). In MA, coalitions are provided with an overall budget cap to work within and as part of their planning are required to consider the cost of implementation as they make their EBP strategy selections. In NY, where communities receive funding for staffing and administrative costs and EBP strategy implementation based on population size, coalitions are encouraged, but not required, to consider the cost of EBP strategy implementation as they make their selections. In OH, all communities are provided with fixed and equivalent budgets with limits on expenditures for naloxone and technical assistance; coalitions consider these budget caps as they choose EBP strategies but are not asked to estimate the cost of individual EBP strategies. In KY, coalitions are encouraged *not* to consider cost, but rather to focus on potential need, impact, and feasibility of each EBP strategy.

For peer review only

Step 3. Select EBP strategies to include in the community action plan

In full coalition and coalition subgroup meetings, members review narrative notes, scores, and rankings from the process described above. In two states (KY, OH), coalitions are also provided with feedback from HCS faculty and staff on implementation feasibility with respect to structural or regulatory barriers. Coalitions weigh these considerations in the context of the guidelines described in Figure 1 and choose through consensus or majority vote the EBP strategies to be implemented with support of HCS. In making their initial selections, coalition

members understand that they can later add and refine EBP strategies as capacity expands. Only one state (KY) set a maximum for the number of EBP strategies that could be selected initially, not including OEND strategies already fast-tracked. Coalitions in KY are limited to twelve initial EBP strategies, requiring that they choose no more than three involving OEND, two for MOUD expansion, two for linkage to MOUD, two to improve MOUD retention, and three focused on safer opioid prescribing/dispensing.

Step 4. Identification of next steps for implementation of selected EBP strategies

Once EBP strategies are chosen, coalitions are encouraged to list partner agencies. In three states, coalition-listed agencies are suggested as potential partners for implementation (KY, NY, OH). In MA, the partner agencies for implementation are determined by coalition subgroups as part of the planning process and voted on by the coalitions, with the acceptation of agencies delivering office-based addictions treatment; these agencies were preselected by the study team. Coalition review of selected EBP strategies in one state (KY) also involves members describing what the coalition could do to support rollout (e.g., write op-eds in the local paper in support of the EBP strategy, provide warm handoffs to agencies, etc.) and ways to reach underserved populations with a primary focus on people of color and Spanish speaking individuals. Iteratively with coalition review, HCS faculty and community staff in each state conduct internal reviews. In MA, state government partners are also involved in the internal review. Internal reviews focus on ensuring the EBP strategies selected adhere to the ORCAA menu and include the required high-risk populations and sectors, potential impact and feasibility. Internal teams also determine next steps for team members, such as initial meetings with partner organizations, contracting considerations, and provision of technical assistance. In one state (MA), the review

For peer review only

also includes planning for the mitigation of inequities associated with each EBP strategy and examination of each EBP strategy's cost as estimated by coalitions with technical assistance from community staff and HCS faculty.

Upon completion of the steps described above, descriptions of selected EBP strategies are provided to faculty and staff teams with expertise in implementation science, OEND, MOUD, safer opioid prescribing/dispensing, and/or community engagement to start the process of working with agency partners and explore interest in and capacity for implementation of the coalitions selected EBP strategies. This process varies from site to site and involves the development of an agency specific implementation plan.

Assessment

Community research staff complete fidelity checklists monthly to document the extent to which all steps are followed and how long each step takes. EBP strategies selected by the coalitions are coded in the form of triads and entered into a harmonized data system shared across the four states. Specifically, the data are coded by venue type (e.g., addiction treatment facility, social services agency, etc), sector (i.e., criminal justice, behavioral health, or healthcare), and ORCCA menu (i.e., OEND, MOUD, or safer opioid prescribing and dispensing). For each entry in the dataset, additional data are recorded and updated throughout the study; these data include but are not limited to the date that the strategy was selected, when it was implemented, and whether it was successfully implemented. Analysis of the triad data is the focus of another paper and will involve enumeration of strategies selected across communities and by community characteristics. Data are also being collected on the strategies' reach (e.g., number of naloxone units distributed, number of clients involved in care navigation, etc). Cost of

For peer review only

the intervention, including the community engagement process, is also being assessed, as described in detail elsewhere. To collect data on coalition members' perspectives on the community engagement process, the HEALing Community Study includes repeated coalition member surveys and qualitative interviews which among many other domains, probes respondents on their experiences with the coalition and strengths and weaknesses of the partnership.[66 67]

Ethics and Dissemination

The HEALing Communities Study protocol was approved by a central Institutional Review Board (IRB; Advarra Inc., Pro00038088) and is being conducted in accordance with The Code of Ethics of the World Medical Association (Declaration of Helsinki). As described elsewhere,[16] because no one person or group of people possess(es) the authority to give consent on behalf of all community members, investigators sought expert consultation and applied guidelines from the Ottawa Statement.[68] Because the CTH intervention poses no more than minimal risk to community members and the research could not be carried out otherwise (see 45.CFR.46.116), a waiver of informed consent was obtained for all community members who may be affected by the CTH intervention.

The action planning tools are disseminated to coalition members in hardcopy, via email, and/or through a password-protected, online portal. We plan to disseminate updated versions of these tools to communities currently in the waitlist-control group via the same mechanisms when intervention begins in their communities. Results of the action planning process (i.e., EBP strategy selections) are shared with community members, community agencies, and community advisory boards through meetings, online and print media, and presentations to community

For peer review only

stakeholder groups in the intervention communities. In the future, we also plan to disseminate our findings at relevant conferences, meetings and through peer-reviewed journals. Finally, we will disseminate our findings, manuals, toolkits, and other resources through federal partners including the National Institutes of Health, Substance Abuse and Mental Health Services Administration and the Centers for Disease Control and Prevention.

For peer review only

Table 1. Characteristics of action planning process that were unique to certain states

**Action Planning Steps**	**Characteristics**	**KY**	**MA**	**NY**	**OH**
Step 1. Develop Data-driven Community Goals	*Supplementary factors considered by coalitions:*				
	• Agent-based and systems dynamic modeling			X	
	• EBP strategy sustainability		X	X	
	• Reach to high-risk populations and priority venues		X		X
	• Equity across ethnic and racial groups		X	X	
Step 2. Discuss and Prioritize EBP strategies	Development of EBP strategy list considered by coalition	Pre-populated by the academic team	Generated by coalitions[1]	Generated by coalitions[1]	Generated by coalitions[1]
	Method of prioritizing strategies	Likert scale ratings of size of current service gap, feasibility, and potential impact.	Consensus building group dialogues	Rank by impact and feasibility	Rank by impact and feasibility
	Coalition consideration of cost	Encouraged *not* to consider cost, but to focus on need, impact, and feasibility of each EBP strategy.	Provided with budget cap and required to consider costs	Encouraged, but not required	Encouraged, but not required
	Supplementary factors considered by coalitions:				
	• EBP strategy sustainability		X	X	
	• Method of EBP expansion (i.e., new service, or scale-up of an existing service via a new method of delivery, target population, or feature)			X	X

For peer review only - http://bmjopen.bmj.com/site/about/guidelines.xhtml

Action Planning Steps	Characteristics	KY	MA	NY	OH
Step 3. Select EBP strategies to include in action plan	Limits on strategy selections	12 initial EBP strategies	No limit	No limit	No limit
Step 4. Identify next steps for implementation of selected EBP strategies	Coalition input on agencies to be involved in implementation	Coalitions suggest potential agencies to involve in implementation.	Coalition subgroups determine which agencies will be involved in implementation.	Coalitions suggest potential agencies to involve in implementation.	Coalitions suggest potential agencies to involve in implementation.
	Coalition input on next steps for beginning implementation	Coalition describes what they can do to support implementation and reach underserved populations who may experience disparities in access.	Coalitions estimate cost of each strategy with technical assistance from the academic team.	None beyond action plan established in Step 3	None beyond action plan established in Step 3

EBP: evidence-based practices

[1] "Coalitions" is inclusive of full coalitions, coalition workgroups, and coalition champions with expertise in specific EBPs

Authors' contributions

The following individuals contributed to the design of the action planning protocol described in this manuscript: AY, PRF, LSM, LF, MRL, JB, MSL, TH, AB, BR, RBS, and TAB. The following individuals made substantial contributions to the implementation of the action planning protocol: AY, PRF, AF, LF, JG, CK, MRL, HS, SLW, JB, MSL, TH, AB, and MQ. The following individuals made substantial contributions to the drafted work and/or substantively revised it: AY, JB, TH, LSM, RC, EO, TW, TAB, TB, RBS, AB, AF, LF, LMG, PRF, JG, CK, MSL, MRL, MQ, BR, HS, SLW. All authors read and approved the final manuscript and agree to be accountable for all aspects of the work.

Funding statement

This research was supported by the National Institutes of Health through the NIH HEAL (Helping to End Addiction Long-term^{SM}) Initiative under award numbers UM1DA049394, UM1DA049406, UM1DA049412, UM1DA049415, UM1DA049417 (ClinicalTrials.gov Identifier: NCT04111939). The Substance Abuse and Mental Health Services Administration (SAMHSA) is a non-funding sponsor of the study. This study protocol (Pro00038088) was approved by Advarra Inc., the HEALing Communities Study single Institutional Review Board. We wish to acknowledge the participation of the HEALing Communities Study communities, community coalitions, and Community Advisory Boards and state government officials who partnered with us on this study. The content is solely the responsibility of the authors and does not necessarily represent the official views of the National Institutes of Health, the Substance Abuse and Mental Health Services Administration or the NIH HEAL Initiative^{SM}.

Competing interest statement

Competing interest disclosures include the following: LSM is a consultant for the Boston Public Health Commission, Action for Boston Area development and the Young Foundation, SLW has served as a scientific advisor to Opiant Pharmaceuticals, Titan Pharmaceuticals, Astra Zeneca, Cerevel, Arbor Pharmaceuticals, Brainsway, Summit Biosciences, Trevi and Camurus. JB ad ML receive investigator-initiated support paid to the institution from Gilead Sciences, Inc. MRL has consulted for Titan Pharmaceuticals and Camurus. AFB is the Program Director of Voices of Hope, a University of Kentucky HCS partner organization. PRF, HS, AY, EO, LMG, RC, JG, CK and LCF have no competing interests.

Data Sharing Statement

The protocol described in this manuscript is for a community-engaged action planning process by which communities choose strategies to implement evidence-based practices in their communities. Data for this paper are considered to be the tools such as decision aids, action plans, and other documents used to facilitate the process and are included as a Supplementary Appendix. The protocol described in this manuscript is part of a larger trial for which data are of a different type; those data are described in the trial registry.

Acknowledgments

We wish to acknowledge the participation of the HEALing Communities Study communities, community coalitions, and Community Advisory Boards and state government officials who partnered with us on this study. We extend our sincere gratitude to the staff and community

For peer review only

members who have dedicated their time and passion to this project and without whom the HEALing Communities Study would not be possible.

For peer review only

References

1. Krausz RM, Westenberg JN, Ziafat K. The opioid overdose crisis as a global health challenge. *Current Opinion in Psychiatry* 2021;34(4):405-12.

2. World Health Organization. Opioid overdose 2021 [updated August 4 2021. Available from: https://www.who.int/news-room/fact-sheets/detail/opioid-overdose accessed August 15 2021.

3. National Institute on Drug Abuse. Overdose Death Rates Bethesda, MD2021 [updated January 29, 2021. Available from: https://www.drugabuse.gov/drug-topics/trends-statistics/overdose-death-rates accessed August 15 2021.

4. Ahmad FB RL, Sutton P. Provisional drug overdose death counts: National Center for Health Statistics. Designed by LM Rossen, A Lipphardt, FB Ahmad, JM Keralis, and Y Chong: National Center for Health Statistics., 2021.

5. Strang J, Volkow ND, Degenhardt L, et al. Opioid use disorder. *Nature Reviews Disease Primers* 2020;6(1):3. doi: 10.1038/s41572-019-0137-5

6. Bohnert AS, Guy Jr GP, Losby JL. Opioid prescribing in the United States before and after the Centers for Disease Control and Prevention's 2016 opioid guideline. *Annals of internal medicine* 2018;169(6):367-75.

7. Walley AY, Xuan Z, Hackman HH, et al. Opioid overdose rates and implementation of overdose education and nasal naloxone distribution in Massachusetts: interrupted time series analysis. *British Medical Journal* 2013;346

8. Larochelle MR, Bernson D, Land T, et al. Medication for opioid use disorder after nonfatal opioid overdose and association with mortality: a cohort study. *Annals of internal medicine* 2018;169(3):137-45.

9. Wu E, Villani J, Davis A, et al. Community dashboards to support data-informed decision-making in the HEALing communities study. *Drug and alcohol dependence* 2020;217:108331-31. doi: 10.1016/j.drugalcdep.2020.108331

10. Welty L, Harrison A, Abram K, et al. Substance Abuse and Mental Health Services Administration.(2017). Key substance use and mental health indicators in the United States: Results from the 2016 National Survey on Drug Use and Health (HHS Publication No. SMA 17-5044, NSDUH Series H-52). Rockville, MD: Center for Behavioral Health Statistics and Quality. *Substance Abuse and Mental Health Services Administration Retrieved College of Health Sciences* 2019;106(5):128.

11. Williams AR, Nunes EV, Bisaga A, et al. Developing an opioid use disorder treatment cascade: A review of quality measures. *Journal of substance abuse treatment* 2018;91:57-68.

For peer review only

12. Freeman PR, Hankosky ER, Lofwall MR, et al. The changing landscape of naloxone availability in the United States, 2011–2017. *Drug and alcohol dependence* 2018;191:361-64.

13. Guy Jr GP, Zhang K, Bohm MK, et al. Vital signs: changes in opioid prescribing in the United States, 2006–2015. *MMWR Morb Mortal Wkly Rep* 2017;66(26):697.

14. Guy Jr GP, Zhang K, Halpin J, et al. An examination of concurrent opioid and benzodiazepine prescribing in 9 states, 2015. *American journal of preventive medicine* 2019;57(5):629-36.

15. Guy GP, Zhang K, Schieber LZ, et al. County-level opioid prescribing in the United States, 2015 and 2017. *JAMA internal medicine* 2019;179(4):574-76.

16. Walsh SL, El-Bassel N, Jackson RD, et al. The HEALing (Helping to End Addiction Long-term SM) Communities Study: Protocol for a cluster randomized trial at the community level to reduce opioid overdose deaths through implementation of an integrated set of evidence-based practices. *Drug and Alcohol Dependence* 2020;217:108335.

17. Martinez LS, Rapkin BD, Young A, et al. Community engagement to implement evidence-based practices in the HEALing Communities Study. *Drug and Alcohol Dependence* 2020;217:108326.

18. Winhusen T, Walley A, Fanucchi LC, et al. The opioid-overdose reduction continuum of care approach (ORCCA): evidence-based practices in the HEALing communities study. *Drug and Alcohol Dependence* 2020;217:108325.

19. Lefebvre RC, Chandler RK, Helme DW, et al. Health communication campaigns to drive demand for evidence-based practices and reduce stigma in the HEALing Communities Study. *Drug and alcohol dependence* 2020;217:108338.

20. Rosenthal BB, Greenawalt J, Ivery JM, et al. Coalitions: Essential tools for organizing. In: Staples L, ed. Roots to Power: A Manual for Grassroots Organizing: A Manual for Grassroots Organizing. London: Praeger 2016:297.

21. Oesterle S, Kuklinski MR, Hawkins JD, et al. Long-Term Effects of the Communities That Care Trial on Substance Use, Antisocial Behavior, and Violence Through Age 21 Years. *Am J Public Health* 2018;108(5):659-65.

22. Shapiro VB, Hawkins JD, Oesterle S. Building local infrastructure for community adoption of science-based prevention: The role of coalition functioning. *Prevention Science* 2015;16(8):1136-46.

23. Arthur MW, Hawkins JD, Brown EC, et al. Implementation of the Communities That Care prevention system by coalitions in the Community Youth Development Study. *Journal of Community Psychology* 2010;38(2):245-58.

24. Butterfoss FD. Coalitions and partnerships in community health: John Wiley & Sons 2007.

For peer review only

25. Wandersman A, Goodman RM, Butterfoss FD. Understanding coalitions and how they operate. In: Minkler M, ed. Community organizing community building for health. 2 ed. New Brunswick, NJ Rutgers University Press 1997:261-77.

26. Powell BJ, McMillen JC, Proctor EK, et al. A compilation of strategies for implementing clinical innovations in health and mental health. *Medical care research review* 2012;69(2):123-57.

27. Rugs D, Hills HA, Moore KA, et al. A community planning process for the implementation of evidence-based practice. *Evaluation and Program Planning* 2011;34(1):29-36.

28. Pellecchia M, Mandell DS, Nuske HJ, et al. Community–academic partnerships in implementation research. *Journal of Community Psychology* 2018;46(7):941-52.

29. Centers for Disease Control and Prevention. Principles of Community Engagement. Second ed, 2011.

30. World Health Organization. WHO community engagement framework for quality, people-centred and resilient health services. : World Health Organization, 2017.

31. World Health Organization. Community engagement: a health promotion guide for universal health coverage in the hands of the people . Geneva: World Health Organization,,, 2020.

32. C. D. I. Study Group. Community-directed interventions for priority health problems in Africa: results of a multicountry study. *Bull World Health Organ* 2010;88(7):509-18. doi: 10.2471/BLT.09.069203 [published Online First: 2009/12/01]

33. World Health Organization & United Nations Children's Fund (UNICEF). Conducting community engagement for COVID-19 vaccines: interim guidance, 31 January 2021: World Health Organization, 2021.

34. Slavova S, LaRochelle MR, Root ED, et al. Operationalizing and selecting outcome measures for the HEALing Communities Study. *Drug and alcohol dependence* 2020;217:108328.

35. Westgate PM, Cheng DM, Feaster DJ, et al. Marginal modeling in community randomized trials with rare events: Utilization of the negative binomial regression model. *Clinical Trials* 2022:17407745211063479.

36. Winhusen T, Theobald J, Lewis D, et al. Development and initial testing of a tailored telephone intervention delivered by peers to prevent recurring opioid-overdoses (TTIP-PRO). *Health education research* 2016;31(2):146-60. doi: 10.1093/her/cyw010

37. Caudarella A, Dong H, Milloy MJ, et al. Non-fatal overdose as a risk factor for subsequent fatal overdose among people who inject drugs. *Drug Alcohol Depend* 2016;162:51-5. doi: 10.1016/j.drugalcdep.2016.02.024

For peer review only

38. Darke S, Mills KL, Ross J, et al. Rates and correlates of mortality amongst heroin users: findings from the Australian Treatment Outcome Study (ATOS), 2001-2009. *Drug Alcohol Depend* 2011;115(3):190-5. doi: 10.1016/j.drugalcdep.2010.10.021 [published Online First: 2010/12/07]

39. Larochelle MR, Bernstein R, Bernson D, et al. Touchpoints - Opportunities to predict and prevent opioid overdose: A cohort study. *Drug and alcohol dependence* 2019;204:107537-37. doi: 10.1016/j.drugalcdep.2019.06.039 [published Online First: 2019/09/03]

40. Strang J, McCambridge J, Best D, et al. Loss of tolerance and overdose mortality after inpatient opiate detoxification: follow up study. *British Medical Journal* 2003;326(7396):959-60. doi: 10.1136/bmj.326.7396.959

41. Binswanger IA, Stern MF, Deyo RA, et al. Release from prison—a high risk of death for former inmates. *New England Journal of Medicine* 2007;356(2):157-65. doi: 10.1056/NEJMsa064115

42. Merrall ELC, Kariminia A, Binswanger IA, et al. Meta-analysis of drug-related deaths soon after release from prison. *Addiction* 2010;105(9):1545-54. doi: 10.1111/j.1360-0443.2010.02990.x

43. Walley AY, Lodi S, Li Y, et al. Association between mortality rates and medication and residential treatment after in-patient medically managed opioid withdrawal: a cohort analysis. *Addiction (Abingdon, England)* 2020;115(8):1496-508. doi: 10.1111/add.14964 [published Online First: 2020/02/25]

44. Sun EC, Dixit A, Humphreys K, et al. Association between concurrent use of prescription opioids and benzodiazepines and overdose: retrospective analysis. *British Medical Journal* 2017;356:j760-j60. doi: 10.1136/bmj.j760

45. Brugal MT, Barrio G, De LF, et al. Factors associated with non-fatal heroin overdose: assessing the effect of frequency and route of heroin administration. *Addiction* 2002;97(3):319-27. doi: 10.1046/j.1360-0443.2002.00058.x [published Online First: 2002/04/20]

46. Cho J, Spence MM, Niu F, et al. Risk of Overdose with Exposure to Prescription Opioids, Benzodiazepines, and Non-benzodiazepine Sedative-Hypnotics in Adults: a Retrospective Cohort Study. *J Gen Intern Med* 2020;35(3):696-703. doi: 10.1007/s11606-019-05545-y [published Online First: 2020/01/09]

47. Gladden RM, O'Donnell J, Mattson CL, et al. Changes in Opioid-Involved Overdose Deaths by Opioid Type and Presence of Benzodiazepines, Cocaine, and Methamphetamine - 25 States, July-December 2017 to January-June 2018. *MMWR Morb Mortal Wkly Rep* 2019;68(34):737-44. doi: 10.15585/mmwr.mm6834a2

48. Park TW, Larochelle MR, Saitz R, et al. Associations between prescribed benzodiazepines, overdose death and buprenorphine discontinuation among people receiving

For peer review only

buprenorphine. *Addiction (Abingdon, England)* 2020;115(5):924-32. doi: 10.1111/add.14886 [published Online First: 2020/01/20]

49. O'Driscoll PT, McGough J, Hagan H, et al. Predictors of accidental fatal drug overdose among a cohort of injection drug users. *American journal of public health* 2001;91(6):984-87. doi: 10.2105/ajph.91.6.984

50. Pabayo R, Alcantara C, Kawachi I, et al. The role of depression and social support in non-fatal drug overdose among a cohort of injection drug users in a Canadian setting. *Drug and alcohol dependence* 2013;132(3):603-09. doi: 10.1016/j.drugalcdep.2013.04.007 [published Online First: 2013/05/04]

51. Wines JD, Jr., Saitz R, Horton NJ, et al. Overdose after detoxification: a prospective study. *Drug Alcohol Depend* 2007;89(2-3):161-9. doi: 10.1016/j.drugalcdep.2006.12.019 [published Online First: 2007/02/07]

52. Tobin KE, Latkin CA. The relationship between depressive symptoms and nonfatal overdose among a sample of drug users in Baltimore, Maryland. *J Urban Health* 2003;80(2):220-29. doi: 10.1093/jurban/jtg025

53. Bosilkovska M, Walder B, Besson M, et al. Analgesics in patients with hepatic impairment: pharmacology and clinical implications. *Drugs* 2012;72(12):1645-69. doi: 10.2165/11635500-000000000-00000 [published Online First: 2012/08/08]

54. Green TC, McGowan SK, Yokell MA, et al. HIV infection and risk of overdose: a systematic review and meta-analysis. *AIDS* 2012;26(4):403-17. doi: 10.1097/QAD.0b013e32834f19b6

55. Jolley CJ, Bell J, Rafferty GF, et al. Understanding Heroin Overdose: A Study of the Acute Respiratory Depressant Effects of Injected Pharmaceutical Heroin. *PLoS One* 2015;10(10):e0140995-e95. doi: 10.1371/journal.pone.0140995

56. Vu Q, Beselman A, Monolakis J, et al. Risk factors for opioid overdose among hospitalized patients. *J Clin Pharm Ther* 2018;43(6):784-89. doi: 10.1111/jcpt.12701 [published Online First: 2018/05/26]

57. Bazazi AR, Zelenev A, Fu JJ, et al. High prevalence of non-fatal overdose among people who inject drugs in Malaysia: Correlates of overdose and implications for overdose prevention from a cross-sectional study. *International Journal on Drug Policy* 2015;26(7):675-81. doi: 10.1016/j.drugpo.2014.11.010

58. Slavova S, Quesinberry D, Hargrove S, et al. Trends in Drug Overdose Mortality Rates in Kentucky, 2019-2020. *JAMA network open* 2021;4(7):e2116391-e91. doi: 10.1001/jamanetworkopen.2021.16391

59. Slavova S, Rock P, Bush HM, et al. Signal of increased opioid overdose during COVID-19 from emergency medical services data. *Drug and alcohol dependence* 2020;214:108176-76. doi: 10.1016/j.drugalcdep.2020.108176

For peer review only

60. Stephenson J. Drug Overdose Deaths Head Toward Record Number in 2020, CDC Warns. *JAMA Health Forum* 2020;1(10):e201318-e18. doi: 10.1001/jamahealthforum.2020.1318

61. Wakeman SE, Green TC, Rich J. An overdose surge will compound the COVID-19 pandemic if urgent action is not taken. *Nature Medicine* 2020;26(6):819-20. doi: 10.1038/s41591-020-0898-0

62. Holland KM, Jones C, Vivolo-Kantor AM, et al. Trends in US emergency department visits for mental health, overdose, and violence outcomes before and during the COVID-19 pandemic. *JAMA psychiatry* 2021;78(4):372-79.

63. Friedman J, Beletsky L, Schriger DL. Overdose-related cardiac arrests observed by emergency medical services during the US COVID-19 epidemic. *JAMA psychiatry* 2021;78(5):562-64.

64. Centers for Disease Control and Prevention. Overdose Deaths Accelerating During COVID-19 2020 [Available from: https://www.cdc.gov/media/releases/2020/p1218-overdose-deaths-covid-19.html accessed May 19 2021.

65. Centers for Disease Control and Prevention. Increase in Fatal Drug Overdoses Across the United States Driven by Synthetic Opioids Before and During the COVID-19 Pandemic: CDC Health Alert Network, 2020.

66. Aldridge AP, Barbosa C, Barocas JA, et al. Health economic design for cost, cost-effectiveness and simulation analyses in the HEALing Communities Study. *Drug and alcohol dependence* 2020;217:108336.

67. Knudsen HK, Drainoni M-L, Gilbert L, et al. Model and approach for assessing implementation context and fidelity in the HEALing Communities Study. *Drug and alcohol dependence* 2020;217:108330.

68. Taljaard M, Weijer C, Grimshaw JM, et al. The Ottawa Statement on the ethical design and conduct of cluster randomised trials: precis for researchers and research ethics committees. *British Medical Journal* 2013;346:f2838. doi: 10.1136/bmj.f2838

For peer review only

Figure 1. Guidelines for HEALing Communities Study Community Action Planning Process to reduce opioid overdose deaths through overdose education and naloxone distribution, expansion of medications for opioid use disorder, and safer opioid prescribing and dispensing

For peer review only

Figure 2. Core components of the HEALing Communities Study Community Action Planning Process to reduce opioid overdose deaths through overdose education and naloxone distribution, medications for opioid use disorder, and safer opioid prescribing and dispensing

For peer review only

- Coalitions *must* select a minimum of five strategies for implementing evidence-based practices (EBPs) from the ORCCA menus including one involving each of the following
  - ORCCA Menu 1: Active OEND (i.e., proactively offer OEND to those at risk)
  - ORCCA Menu 2A: Expand MOUD access
  - ORCCA Menu 2B: Increase linkage to MOUD
  - ORCCA Menu 2C: Improve retention in MOUD
  - ORCCA Menu 3: Safer opioid prescribing/dispensing

- Priority sectors: Selected EBP strategies *must* include at least one to be implemented in each of the following:
  - Healthcare (i.e., outpatient healthcare centers, pre-hospital providers, emergency departments and urgent care, hospitals, primary care settings, public health departments, and pharmacies)
  - Behavioral health (i.e., substance use disorder and mental health treatment centers and social service agencies, including syringe service programs)
  - Criminal justice (i.e., pre-trial, jails, probation, parole, drug and problem-solving courts, police and "narcotics" task forces, halfway houses, community-based correctional facilities, and department of youth services)

- Key populations: Coalitions are encouraged to consider EBP strategies focused on those most vulnerable to opioid overdose:
  - People with a prior opioid overdose
  - People with reduced opioid tolerance
  - People who use other substances
  - People who have concomitant major mental illness
  - People who have a concomitant major medical illness
  - People who inject drugs

- Priority venues: Coalitions are encouraged to consider EBP strategies to be implemented in priority settings:
  - OEND: criminal justice settings, syringe service programs, emergency departments and acute care hospitals, sites of overdose for naloxone leave-behind, and mental health/addiction treatment programs
  - MOUD: criminal justice settings, primary care, general medical and behavioral/mental health settings, specialty addiction/substance use disorder treatment settings, and recovery programs
  - Safer opioid prescribing and dispensing: inpatient service, emergency/urgent care, outpatient clinics, ambulatory surgery, dental clinics, pharmacies

Figure 1. Guidelines for HEALing Communities Study Community Action Planning Process to reduce opioid overdose deaths through overdose education and naloxone distribution, expansion of medications for opioid use disorder, and safer opioid prescribing and dispensing

165x210mm (330 x 330 DPI)

For peer review only - http://bmjopen.bmj.com/site/about/guidelines.xhtml

1) Develop data-driven community goals

- Coalitions reviewed (1) lists of organizations in the community relevant to potential implementation of EBP strategies (e.g., substance use disorder treatment centers, harm reduction programs, emergency response agencies, hospitals), and (2) information about OEND, MOUD, and other related services available through administrative data and/or through phone surveys with community agencies. Coalitions used goal setting tools to describe strengths, services, gaps, and set goals with varying specificity.

2) Discuss and prioritize EBP strategies

- Coalitions reviewed decision aid tools (Supplementary Appendix) listing EBPs. Coalitions discussed relevant existing services, size of the gap between existing services and need, feasibility, and potential impact on opioid-involved overdose deaths. In some cases, coalitions scored the EBPs on Likert scales and/or ranked them according to impact and feasibility.

3) Select EBP strategies to include in the community action plan

- Coalitions reviewed narrative notes, scores, and rankings from the process described above and selected by consensus or majority vote at least five EBPs, including one each from the following categories: active OEND, MOUD expansion, linkage to MOUD, retention in MOUD, and safer opioid prescribing and dispensing.

4) Identify next steps for implementation of selected EBP strategies

- Coalitions suggested agencies that could partner to implement their selected strategies. Iteratively with coalition review, subject matter experts and staff reviewed strategies to determine next steps for team members (e.g., initial meetings with partner organizations, contracting considerations, and technical assistance).

OEND: overdose education and naloxone distribution, MOUD: medications for opioid use

Figure 2. Core components of the HEALing Communities Study Community Action Planning Process to reduce opioid overdose deaths through overdose education and naloxone distribution, medications for opioid use disorder, and safer opioid prescribing and dispensing

162x153mm (330 x 330 DPI)

Appendix 1. Kentucky Action Planning Tools

Community Goal Setting Tool

<u>Purpose</u>: To describe existing infrastructure, gaps, opportunities, needs, and goals related to opioid overdose prevention.

<u>Description</u>: The Kentucky Community Engagement and Continuum of Care teams partnered to facilitate communities' identification of their strengths and opportunities for expansion related to opioid overdose prevention followed by thoughtful consideration and selection of the ORCCA strategies. The process began with the Community Engagement team working with the coalition to describe existing infrastructure, gaps, opportunities, needs, and goals related to opioid overdose prevention in their communities using a Community Goal Setting Tool. The coalitions and/or menu-specific workgroups worked on completing the tool for each menu separately and presenting them to the full coalition.

<u>Who completes the tool</u>: Coalitions and/or coalition workgroups with facilitation by faculty and staff.

Community Goal Setting Tool — Menu 1

Menu 1 Goal: Increase Opioid Overdose Prevention Education and Naloxone Distribution (OEND)

Brainstorm! Think about your community: what you've experienced, what you learned from your community profile and data dashboard, and what you envision for the future. Answer the questions below and jot down your thoughts

What are we doing well? What OEND services already exist?

Ex. There is active Opioid Overdose Education and Naloxone Distribution (OEND) already occurring in the County Detention Center. The Detention Center is currently able to provide naloxone to all persons released from the facility (as part of partnership with the HEALing Communities Study) and is also partnering with other organizations to distribute naloxone to anyone who is interested and completes the requisite virtual training.

Who needs OEND in our community?

Ex. There is still a significant need for distribution among the peer community and their social networks, in homes with bystanders, and with medical and law enforcement professionals in contact with those at high-risk for overdose.

Where in our community should OEND services be provided?

Ex. There is significant opportunity for OEND expansion in Emergency Departments and the healthcare system generally, in pharmacies, with local Quick Response Teams (QRTs), in Substance Use Disorder (SUD) treatment facilities, and in other locations where the peer community can be found. The NKY Health Department also has capacity to expand OEND services.

Community OEND Goals:

Example:

1. Expand OEND in criminal justice settings, including Probation & Parole and Drug Court.
2. Expand OEND in healthcare settings, including Emergency Departments and Substance Use Disorder (SUD) treatment programs.
3. Expand OEND access among peer community and their support systems (via recovery support groups, social service agencies, and in "hot spots" frequented by those at high-risk, etc.).
4. Expand OEND with local QRTs.
5. Increase first responders carrying/administering naloxone.

Decision Aid Tool

Purpose: To facilitate discussion and prioritization of evidence-based practice strategies that align with community goals and the ORCCA

Description: HCS staff then populated menu-specific Decision Aid Tools with strategies listed in the ORCCA and TAG, integrating coalition's suggestions for specific strategies when available from Community Goal Setting Tool. In meetings facilitated by faculty and staff with expertise in community-engagement and the continuum of care, the coalitions and/or menu-specific workgroups reviewed each possible strategy one-by-one, offering a narrative description of factors that they believed would be relevant to deciding whether to implement the strategy (i.e., current relevant activities, barriers they anticipate encountering, agencies relevant to implementation). After offering a narrative description, the coalition scored on Likert scales the size of the current gap in the service/strategy (4-point scale; 0-3), feasibility of implementation within the next 12 months (4-point scale; 0-3), and its potential impact on overdose deaths (3-point scale; 1-3). Each dimension was scored through consensus or through in-meeting polling. Coalitions did not use specific criteria or thresholds in assigning the scores, rather they often scored them based on how they compared to others on the menu. Scores were summed to produce an overall "priority score" for each strategy.

Who completes the tool: Coalitions and/or coalition workgroups with facilitation by faculty and staff.

For peer review only

Menu 2: Strategies to Enhance Delivery of MOUD Maintenance Treatment, Including Agonist/Partial Agonist Medication					
**Strategy and Venue**	**Current Activity**	**Size of Gap** (Current Activity vs. Need)	**Feasibility in <12 Months**	**Impact on Overdose Deaths**	**Priority Score**
Working with the ORCCA menu, brainstorm possible strategies to meet the Community MOUD goals. Record all strategies in this column.	Use this column to briefly summarize current activity in your community	0. None<br>1. Small<br>2. Medium<br>3. Large	0. Extremely Low<br>1. Small<br>2. Medium<br>3. Large	 1. Small<br>2. Medium<br>3. Large	Add previous 3 columns; if any column contains 0, priority score is 0
**a) Expand MOUD Treatment Availability (Capacity Building) – REQUIRED**					
**Add/expand MOUD in primary care, other general medical/BH settings, and specialty addiction/SUD & recovery programs**					
Ex. Expand Methadone in county OTPs 2.a.1.2	Ex. Have two OTP providers but one has long wait times; need to figure out what they need to be able to treat more patients				
**Add/expand MOUD treatment in Criminal Justice Settings (pre trial, jail, prison, probation, parole)**					
Ex. Expand prescription of XR naltrexone and buprenorphine within jail	Ex. Interest in XR-naltrexone at the jail and jail might be open but have a lot of concerns/barriers				
**Expand access to MOUD through telemedicine, interim buprenorphine or methadone, or medication units**					
Ex. Expand use of Telemedicine/telehealth/tele-consults for buprenorphine treatment	Ex. Telehealth opportunities have grown due to COVID 19, but it is unknown whether this option will remain available.				
**b) Interventions to Link to MOUD – REQUIRED**					
**Linkage Programs (All Relevant settings)**					
Ex. Develop linkage program for incarcerated individuals about to be released (with peer support and/or other staff)	Ex. No current activity, the jail is somewhat interested but there are several barriers				
**Bridging MOUD medications as Linkage Adjunct (all relevant settings)**					
Ex. Expand bridge programs within county ER and inpatient settings	Ex. An existing bridge program is working well and can use as model for others. Difficulty with staffing & infrastructure is main barrier				
**c) MOUD Treatment Engagement and Retention – REQUIRED**					
**Enhancement of clinical delivery approaches that support engagement and retention**					
Ex. Expand recovery support services (peer support, telephone recovery support, recovery coaching, family support) in criminal justice settings (Voices of Hope)	Ex. Add peer supports within system; no current long term supports, or programs/mentorship programs engaged				
**Use virtual retention approaches (e.g. mobile, web, digital therapeutics)**					
Ex. Implement use of re-SET-0 app to improve retention, incentives, and education 2.c.2	Ex. Some clinics within county already using it; potential cost barriers and potential disinterest from buprenorphine providers				
**Utilize retention care coordinators**					
Ex. Utilize retention care navigators to increase/add care navigators in all MOUD treatment settings 2.c.3	Ex. Other care navigation efforts are already underway that could be leveraged, and there is a desire to increase support of and leverage local efforts where possible				
**Integrate mental health and polysubstance use treatment into MOUD care**					
Ex. Offer regular training between primary care/non MH providers with addiction psychiatry and other MH providers to manage co-morbidity with OUD	Ex. Unsure if providers would be interested in engaging				
**Reduce barriers to housing, transportation, childcare, and access to other community benefits for People with OUD**					
Expand transportation services/offerings to align with MOUD treatment center services (location, hours, etc.)	Ex. Local agency offers transportation services to clients				

Action Planning Tool

Purpose: To facilitate discussion and final strategy selection.

Description: The list of strategies discussed in the Decision Aid Tool was used to pre-populate an Action Planning Tool. The tool listed all strategies with their scores and new implementation feasibility ratings (High, Medium, Low) provided by faculty with expertise in implementation science and the continuum of care based on their knowledge of policy- and payor-level barriers and time required for scale up. The pre-populated Action Planning Tools were presented to coalitions for discussion and strategy selection through consensus.

Who completes the tool: Coalitions and/or coalition workgroups with facilitation by faculty and staff.

Action Planning Tool — Menu 3

For each strategy from the Decision Aid that had a priority score above 0, fill in the strategy and venue (i.e., from Column 1 of the Decision Aid), record the scores from the Decision Aid Tool in the Scores columns, and fill in the relevant sector. As coalitions consider their selection of strategies, they should ensure that strategies collectively will target those at highest risk (i.e., people with history of prior overdose, reduced opioid tolerance, who engage in polydrug use, with major medical or mental illness, and/or who inject drugs). The HCS Research Team will help you complete the Implementation Feasibility column by providing insight on whether each strategy has a high, medium, or low feasibility of implementation based on resources, partnerships, and other factors. After reviewing content of the other columns, coalitions should come to consensus on the Overall Priority Ranking with the highest priority strategy ranked 1, the second highest priority ranked 2, and so on. Of note, the Overall Priority Ranking may or may not correspond directly to the rank order from the other scoring columns. After completing this tool, the next step will involve creating an initial implementation plan for the top 1-3 overall priority ranked strategies; at least one of the strategies must be from the required sub-menu (i.e., Safe Prescribing/Dispensing).

Menu 3: Strategies to Improve Prescription Opioid Safety							
Strategy and Venue	Scores				Sector?	Implementation Feasibility? (High/Medium/Low)	Overall Priority Ranking
	G	F	I	PS			
**a) Safer Opioid Prescribing/Dispensing Practices (Required)**							
Ex. Education materials around safe opioid disposal, targeting healthcare providers and patients.	3	3	2.25	8.25	HC	High	1
Ex. Education for community pharmacists focusing on red flags during opioid dispensing (dose, indication, duration, concurrent therapies, comorbid conditions, age, etc.).	1.25	2.5	1.5	5.25	HC	High	2
Ex. Educational outreach and academic detailing related to pain management for patients with OUD	2.5	2	2	6.5	HC	Medium	
Ex. Patient educational materials regarding appropriate chronic pain management made available in AA meetings and OUD recovery groups	2.5	2.25	1.75	6.5	BH	Medium	3
**b) Safer Opioid Disposal Practices (Optional)**							
Ex. Installation of permanent disposal kiosks	1.8	2.7	2	6.5	HC	High	4
Ex. Expansion and promotion of take-back events	2.3	2.3	2.25	6.85	Other	Medium	

Priority Score: G: Gap; F: Feasibility; I: Impact; PS: Overall Priority Score

Sector: CJ = Criminal Justice, HC = Healthcare, BH = Behavioral Health (See ORCCA Overview for details on assignment of sectors).

Coalition Implementation Planning Tool

Purpose: To more thoroughly define strategies and generate potential lists of venues/agencies to be involved in the implementation

Description: The tool also captured coalition's feedback on what they could do to help support implementation, strategies to ensure reach to under-represented populations, and indicators of success or failure of the strategy. Faculty with expertise in implementation science and the continuum of care added details on what the HCS faculty and staff would do to implement the strategy. Once complete, the tools were reviewed and approved in their entirety by the coalitions.

Who completes the tool: Coalitions and/or coalition workgroups with faculty and staff.

Coalition Implementation Planning Tool — Menu 3

For at least one but no more than three of the strategies with the top Overall Ranking from the Action Plan table, create an initial implementation plan using the table below (i.e., one table per strategy for up to three strategies). At least one of these strategies must come from the required sub-menu in the action plan (i.e., must address safe prescribing/dispensing practices). Provide information about specific activities in as much detail as is feasible at this time; insert additional rows as needed to describe these activities for the coalition and the HCS team.

**Menu 3 Overall Ranking = 1**<br>**Strategy: Educational outreach and academic detailing related to opioid prescribing for routine pain management. (Includes multiple sub-strategies as outlined on the Community Action Planning Tool).**		
**What specifically will be done?** Ex: Educational outreach and academic detailing for all willing opioid prescribers (and possibly ambulatory pharmacists) in the County. Top priorities are: Primary Care, Hospital Emergency Department & local Urgent Care facilities, Dental Offices, Ortho Practices, Pain Management Clinics		
**What organization(s) should be involved? Provide points of contact if possible.**	Agency: Ex. Family Practice	Contact: Ex. Dr. Smith
	Agency: Ex. Pharmacy	Contact: Ex. Dr. Jones
	Agency:	Contact:
**What will the coalition do?**		**Assigned to:**
1. Ex. Facilitate contact with Family Practice, Ambulatory Pharmacists		1. Ex. Coalition Chair
2. Ex. Help with endorsement letter (with signatures from local hospital MDs		2. Ex. Safe Prescribing Champion
**What will HCS do?**		**Assigned to:**
1. Coordinate with listed coalition members to connect with identified organizations.		1. Ex. Prevention Team
2. Draft a promotional letter/email to be reviewed and signed by community leaders who healthcare professionals respect and would positively respond to and recognize.		2. Ex. Prevention Team
3. Develop community-specific implementation plan, target list, and educational materials using audiences and messaging the coalition identifies.		3. Ex. Prevention Team and Community Coordinator
**How could we promote access/impact among diverse populations?**	Ex. Provide all patient-facing materials in Spanish, English, and any other languages deemed necessary; Prioritize County FQHC provider	
**How will we know it is working?**	Ex. Providers are willing to meet and listen/engage with continuing education materials; Providers agree to provide/request patient materials for distribution	
**What will make us change our strategy?**	Ex. Providers are not responsive or willing to engage; Educational materials are not reaching patients.	

Appendix 2. Massachusetts Action Planning Tools

Community Profile Summary Form

Purpose: Discuss and Prioritize EBP Strategies that Align with Community Goals

Description: Developing ORCCA-specific Community Goals was a multi-step iterative process driven by community teams (CEFs, CDMs, Community Faculty and CCs). Small group conversations and 1:1s were scheduled with coalition members, local stakeholders, and champions. Community Faculty set the stage with stakeholders by sharing data that demonstrated the impact a combination of initiation on medications for opioid use disorder (MOUD), improvement on MOUD retention, and increasing the distribution of naloxone could have on the HCS communities. These data facilitated conversations about the potential impact of different EBPs. Teams outlined the importance of increasing access to naloxone, MOUD initiation, and MOUD retention within the context of each community. Together, community teams and stakeholders used community profile information to contextualize these data for their community. Community profiles, which included an overview of opioid-related risk, available treatment, and fatal overdose prevention services in the community, were co-created by study staff and community members. The profile, along with local data and coalition insight was used to identify areas of opportunity where ORCCA related strategies could fill unmet needs and/or enhance extant programming. This information was compiled on the community profile summary form.

Who completes the tool: This was an iterative process that involved community teams and subcommittees soliciting feedback from coalition members, dialoging, vetting ideas and ranking solutions as they grappled with how to maximize impact among high-risk populations.

Massachusetts HCS Wave 1 Community Profile Summary

Community Profile Summary **Coalition: ______________**

ORCCA Interventions	Existing Interventions (currently taking place)	Who are the service providers?	High-risk Populations (currently receiving services)	Existing programmatic collaborations (referrals and partnerships)	Areas of opportunity to improve existing services (to identify gaps)	Sustainability Include: Current funding sources and duration	Community Narratives
**Objective 1: Increase Opioid Overdose Prevention Education and Naloxone Distribution (OEND)**							
Required OEND							
Optional OEND							
Naloxone administration (optional)							
**Objective 2: Outreach and Delivery of Medications for Opioid Use Disorder (MOUD) to High-Risk Populations***							
Expand MOUD treatment (required)							
Linkage to MOUD (required)							
Engagement and retention (required)							
**Objective 3: Improve Prescription Opioid Safety**							
Safer Prescribing/ Dispensing (required)							
Safer Disposal Practices (optional)							

*For Definitions of "high-risk populations" see the Section II of the ORCCA Overview

Action Plan Summary Form

Purpose: To comprehensively outline strategies and determine their fiscal feasibility.

Description: Once the community profile summary was complete, community teams and coalitions began the process of selecting specific evidence-based practices. This involved participating in workshops and trainings to learn more about evidenced based strategies supported by the study, conducting 1:1s to engage potential implementation partners through coalition contacts, and assessing the extent to which high-risk populations and venues were engaged with the coalition and conducting outreach. Subgroups engaged in focused conversations exploring potential evidence-based practices using knowledge from previous conversations about evidence-based practices, as well as their feasibility, impact, and sustainability.

Community partners created budgets and estimates that were included later in proposals for action plan items. The action plan summary information was then discussed in subgroup meetings. During subgroup meetings coalition members and partner organizations further flushed out details of their plans refining them for presentation to the larger coalition. At the coalition meeting action plan summaries were discussed and voted on by the coalition membership using decision-making procedures such as consensus or majority voting as outlined in their respective coalition charters.

Who completes the tool: This was an iterative process that involved community teams, subcommittees, and partner organizations.

Massachusetts HCS Wave 1 Community Profile Summary & Action Plan Template

Massachusetts Action Plan Summary **Coalition: XXX**

	Selected Strategies	ORCCA Goal (How it advances the overall HCS goal?	Targeted Populations	Primary High-Risk Sector			Specific Activities Needed to Implement Selected Strategy: (who, what, when, where)	TA Needed	HCS Funds Needed
				BH	CJ	HC			
**ORCCA Objective 2: Outreach and Delivery of MOUD to High-Risk Populations**									
MOUD Linkage (required)	H. Linkage program via embedding an "In-reach worker" at XXX County Correctional Facility	Through the addition of this staff member, inmates leaving the facility will be provided with naloxone and a MOUD treatment appointment, with transportation provided, on release date.<br><br>Worker will be responsible for: creating relationships with inmates, running harm reduction groups, distributing Narcan, making community connections, scheduling same day appointments, scheduling transportation as needed.	People with reduced tolerance post-incarceration		X		X, Y, and Z orgs will work collaboratively to create a 30-40 hour/week position to fill gap between leaving incarceration and returning home in order to support recovery and lower risk of overdose after incarceration. Facility will determine number of residents released to determine number of hours for staff to spend in the facility.<br><br>Representatives from all 3 organizations will create job description and conduct interview to ensure right fit to work with incarcerated individuals. Qualifications will include knowledge of harm reduction principals, MOUD, OEND, and local services and treatment programs for referrals and connections.<br><br>Worker will ensure Narcan kits are given to inmates upon release (8:30am everyday). Worker will assist in activating inmates MA Health and secure appointments with outside service providers to bridge MOUD treatment post-release. Worker will be deputized as TPP concierge (see below) to have access to schedule rides for inmates being released directly to MOUD appointments in the community.	Group facilitator training<br><br>BAMSI and/or HCS to provide harm reduction training.<br><br>Praxis Narcan Train the Trainer<br><br>Facilitation training	Fulltime Salaried Position for 18 months: Payroll/Fringe $74,344.31<br><br>Mileage $855.00<br><br>Phone/Computer/Supplies/PPE $1,800<br><br>Total Direct Cost: $76,999.31<br><br>Admin Fee: $7,699.31<br><br>**TOTAL COST: $84,699.24**

Action Plan Review Form

Purpose: To assess action plan adherence to the ORCCA menu, impact, inclusion of high-risk populations/venues, feasibility and resources.

Description: Community-specific plans approved by the coalition memberships were then reviewed by HCS MA Leadership and State partners from the department of public health using a standard review form assessing: ORCCA adherence, Impact, Inclusion of High-Risk Populations/Venues, Feasibility and Resources. Written feedback was provided to community teams who then discussed feedback with their coalitions. Leadership met with community teams and coalitions as needed to support with plan clarification or revision. Revised plans were resubmitted for approval by HCS MA Core leaders and state partners.

Who completes the tool: HCS MA Leadership and State partners from the department of public health.

For peer review only

**Community:**			

Please comment below about the extent to which the plan meets the designated criteria, including strengths, challenges and modifications.

Approval Criteria	OEND Plan	MOUD Plan	Safer Prescribing Plan
**ORCCA Adherence**<br>☐ Within the scope of the ORCCA menu<br>☐ Aligned with the overall goal of HCS			
**Impact**<br>☐ Extent to which plan will:<br>o Increase # POUD on MOUD<br>o Retain POUD in care<br>o Increase OEND to POUD at highest risk<br>o Address gaps in the continuum of care<br>o Be sustainable			
**High-Risk Population/Venues**<br>☐ Extent to which plan will:<br>o Include efforts to engage with people who have been historically marginalized<br>o Prioritize populations at heightened risk for overdose - BH/CJ/HC (i.e. prior overdose, decreased tolerance, multi-substance use, concomitant medical or mental illness, homeless, non-English speakers, minority populations)<br>o Mitigate and not exacerbate underlying racial inequities<br>o Prevent unintended consequences			
**Feasibility**<br>☐ Extent to which plan is:<br>o Feasible to implement by January 1, 2021<br>o Clear on what will be done by who and by when to bring about change<br>☐ Extent to which structural or regulatory challenges act as a barrier for this plan			
**Resources**<br>☐ Plan is within budget			

For peer review only

Appendix 3. New York Action Planning Tools

Community Evidence Based Practice Goals

Purpose: To define community level goals for EBPs

Description: The starting point for action planning is reflection and discussion by the coalition on the Community Profile, Data Dashboard, and locally sourced data summarized in a Discussion Tool from Phase 3. This is followed by development of community goals for each ORCCA EBP. Coalitions were asked to reflect on the success of COVID-19 influenced fast tracked strategies from earlier phases and to consider expansion and sustainability of these strategies, as well as to use a health and racial equity lens in the development of community Specific, Measurable, Attainable, Realistic and Time-framed (SMART) goals for each EBP.

Who completes the tool: The community-based implementation team captures discussions and decisions from Coalition members starting in workgroups with their champions by ORCCA Menu and further facilitated in the larger coalition.

DIRECTIONS: Setting ORCCA Goals
The Implementation Team facilitates a discussion with the Coalition (Steering Committee) to identify ORCCA goals based on the recommendations of the ORCCA, Communication and Data Workgroups.

These will be community-level goals to provide OEND in high risk populations, provide outreach and delivery of MOUD to high populations, and provide for safer opioid prescribing and dispensing.

ORCCA Goals

Providing OEND in High Risk Populations	Ex. In 12 months expand OEND through four strategies: expanding peer navigation, expand virtual training, using a variety of distribution strategies, and expanding the number, integration and reach of community trainers by 50%.
Outreach and Delivery of MOUD to High-Risk Populations	Ex. In 9 months, expand access to MOUD by increasing the number of waivered MOUD prescribers and treatment slots; improving prescribing rates of current waivered prescribers; create and sustain a prescriber advisory board to support best practice; and enhancing care coordination, outreach and linkage to enhance engagement and retention in care. Use a data feedback system to improve coordination across providers and service systems and address SDH through a targeted response to increase MOUD retention.
Safer Opioid Prescribing and Dispensing	Ex. Improve safer opioid prescribing and dispensing practices by engaging prescribers and pharmacies to provide resources on safer prescribing, tapering practices, alternative pain management and promoting safer opioid disposal practices between 6-12 months from the time of implementation.

Action Planning Worksheet

Purpose: To identify EBP strategies, venues and priority populations in an iterative process using community profiles, and data identifying needs, gaps and opportunities

Description: Workgroups brainstormed possible ORCCA EBP strategies and communication messages following training and practice using the ORCCA and technical assistance guide (TAG) that could fill existing gaps with identified populations, sectors and venues discussed in Phase 3. ORCCA EBP strategies were considered that could strengthen ongoing community efforts, create opportunities for expansion and efficiency, and promote synergistic approaches to new and existing community strategies. These strategies were presented to the coalition for consideration.

Who completes the tool: Coalition members, workgroups led by champions from each menu and facilitated by community implementation teams

Table 1: Discussion Tool for STEP 1 (CTH PHASE 4)

Strategy	**Description & Approach to Population Access (High Risk/Venue)** Briefly describe each specific strategy being considered. For strategy, identify populations of focus and venues for reaching them.		STRATEGY	Pros and Cons of each Strategy
	**High Risk Populations**<br>Prior Opioid Overdose<br>Reduced Opioid Tolerance<br>Other Substance Use<br>Co-occurring mental health<br>Co-occurring physical health<br>Injection Drugs	**Venues**<br>Harm Reduction/Syringe Service<br>Health Care<br>Criminal Justice<br>First Responder<br>Addiction Treatment & Recovery<br>Behavioral Health Treatment<br>Community Based -Social Service Hotline	**Label each strategy:**<br>Scale up<br>New Population<br>New Feature<br>New Service<br>New Service &<br>Population	
**OEND in High Risk Populations**				
Active OEND (required)	*EX: Distribute naloxone to released inmates at County Jail or other setting*		EX: scale up or new service	
Passive OEND (optional)	*EX: Distribute naloxone at pharmacy or dispensing program*		EX: New feature	
Naloxone administration strategies (optional)	*EX: First responder naloxone administration*		EX: scale up	
**Outreach and Delivery of MOUD to High-Risk Populations**				
Bridging MOUD (required)	*EX: bridge program for people returning to community from county jail*		EX: new service	
Linkage using Peer Navigator (required)	*EX: Linkage to peer navigation for patients who overdose in ED*		EX: new feature	
Engage and retain individuals in treatment (required)	*EX: telemedicine, virtual appointment*		EX: new feature	
**Safer Opioid Prescribing and Dispensing**				
Safer opioid prescribing/dispensing practices (required)	*EX: Emergency/urgent care*		EX: scaling up	
Safer opioid disposal practices (optional)	*EX: • Prescription drug drop-box/mail-back programs*		EX: scale up or new service	

An additional tool used to make decisions about strategies and their potential for synergy and impact, was agent-based and systems dynamic modeling. Feedback reports using this modeling were introduced to County Implementation Teams and workgroups alongside community profiles to highlight the importance of OEND and MOUD expansion specifically, in order to meet the intervention's opioid overdose prevention goals (see Figure 3), and to inform the prioritization, adaptation, maintenance and sustainability of effective interventions over time for action planning. The systems perspective is intended to be integrated into problem-solving and feedback during ongoing implementation to improve coordination and resource sharing within community networks.

For peer review only - http://bmjopen.bmj.com/site/about/guidelines.xhtml

Figure 3-NY. Systems Feedback Reports for Planning

Systems Mapping for Action Planning

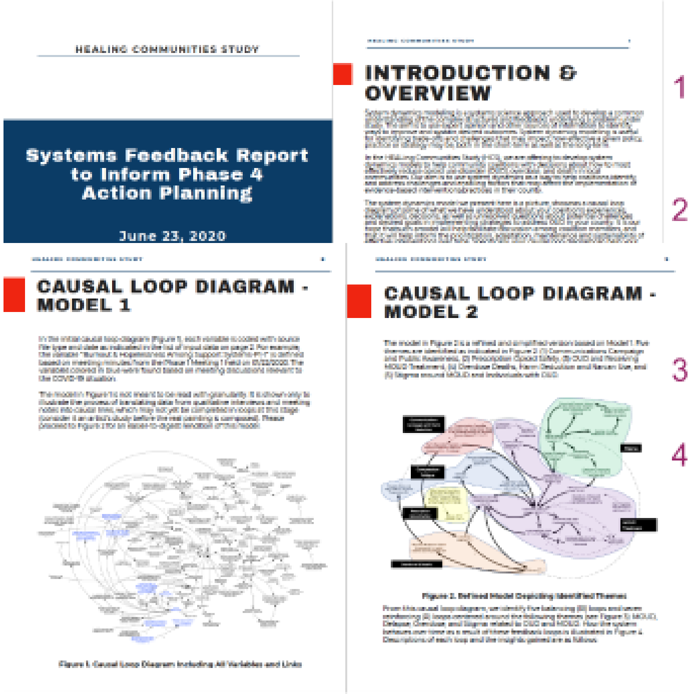

1. Are OEND and MOUD strategies effectively linked to reduce overdose and fatalities?
2. Are there supplemental interventions to support patient engagement, retention and long-term recovery?
3. How is the challenge of stigma recognized and addressed?
4. How are social determinants of health (e.g., homelessness) recognized and addressed?

5

For peer review only

Consultation and Coaching Plan

Purpose: To provide coaching and feedback to assure adherence to HCS required EBP selections, and enhance capacity of local teams for community planning

Description: The research study team, including implementation coaches assigned to each community, offered iterative support as Community Implementation Teams and workgroups ranked strategy options based on earlier discussion of impact and feasibility determinations in workgroups, finalized strategy selections, and refined strategies into specific SMART goals. The ranking and strategy selection process was not uniform across coalitions. While no limits were placed on the number of strategies selected, attention was given to ensure the 5 required strategies defined in the ORCCA were present or a justification for why not were included. Opportunities were highlighted during coaching sessions to reinforce data driven justification for strategy selection and to feature the role of EBP workgroup champions in leading planning.

Who completes the tool: The Study Team reviews the Action Planning Worksheet with Summary Action Plans with the Implementation Team in coaching sessions using the criteria below:

ORCCA Goals	Prioritized EBPs	Draft CAP	Adopted CAP
Goals are aligned with HCS goal to reduce opioid overdose mortality by 40%	Priorities are aligned with the ORCCA-specific goals	CAP summarizes coalition goals	Summarizes coalition goals
Goals target identified gaps in the provision of OEND, MOUD, and safer opioid prescribing and dispensing	Priorities address the gaps documented in Community Profile	CAP summarizes selected strategies	Summarizes selected strategies, venues and populations
	Workgroup prioritized strategies as high impact	CAP identifies the team responsible for implementing the strategies	Identifies the team responsible for implementation planning and organizations for initial engagement
	Workgroup prioritized strategies as high feasibility	CAP identifies and the timeframe for implementation planning	Identifies the timeframe for implementation planning
		To what degree have considered health equity and racial disparities?	Have considered health equity and racial disparities
		Initial TA and resources needed, including plans to use Community Impact (CI) Funds	TA and resources needed, including plans to use CI Funds

For peer review only

SCHEDULING REVIEW AND CONSULTATION

Implementation Teams should upload and maintain documentation for each deliverable (ORCCA Goals, prioritized EBPs and the Community Action Plan) on the Public HCS County Folders drive. Project Managers should notify the Study Team by email to request review and coaching sessions. Please use the following timetable:

Deliverable	When to Request Review	Review Type	Target Deadline	Response Time
**ORCCA Goals**	Workgroup and Coalition consensus on ORCCA goals	Review and comment	**00/00/0000**	2 Business Days
**Prioritized EBPs Draft Community Action Plan**	Prioritized EPBs and draft Community Action Plan is ready for recommendation the Coalition	Coaching Meeting	At least 1 week prior to Coalition Meeting at which EBPs and AP will be considered	2 Business Days
**MOUD and Safer Rx EBP, AP**	Consulting addiction psychiatrists will also provide consultation and review of the prioritized EBPs and draft Community Action Plan for MOUD and Safer Prescribing Workgroups during Workgroup meetings.			
**Approved Community Action Plan**	Coalition has approved the Community Action Plan	Review and comment	Next business day after Coalition approval	2 Business Days

The Implementation Teams can invite the Study Team to join the Workgroup meeting in which EBP strategies are being prioritized to provide review and consultation, if that is the preference of the Workgroup, Champion and Implementation Team.

Summary Community Action Plan Form

Purpose: To provide a summary of EBPs and community goals, venues and priority populations with initial organizations, and first steps to be the focus for implementation planning in Phase 5.

Description: The final step in the Phase 4 Action Planning Worksheet is a summary community action plan for final coalition approval guided by the coalition's charter on decision-making. Most used a majority vote in a coalition meeting. The plan includes defined SMART goals with an outcome target for each ORCCA EBP-related strategy and identification of specific populations, sector for implementation (i.e., behavioral health, criminal justice, healthcare), reference to feasibility and impact rankings from workgroup discussions, and preliminary sustainability. Additionally, in anticipation of Phase 5 Implementation Plan Agreement development, the action plan includes recommended initial and anticipated steps for engaging community partners and estimates of technical assistance and resources.

Who completes the tool: Community implementation teams led by a program manager and CEF complete the summary and present to the coalition for adoption. In some cases a county executive or leadership team provides an additional level of approval.

TABLE 6: Action Plan Table (CTH PHASE 4)

Strategy Category	Selected Strategies	Targeted Populations	High-Risk Sector			Sustainability Feasibility/ Impact	Implementation Steps: who does what, by when and where	Technical Assistance & Other Resources
			BH	CJ	HC			
**ORCCA Objective 1: Increase Opioid Overdose Prevention Education and Naloxone Distribution**								
Active OEND (required)	A1:<br>A2:							
Passive OEND (optional)	B1:<br>B2:							
Naloxone administration (optional)	C1:<br>C2:							
**ORCCA Objective 2: Outreach and Delivery of MOUD to High-Risk Populations**								
Expand MOUD treatment (required)	D1:<br>D2:							
Linkage to MOUD (required)	E1:<br>E2:							
Engagement and retention (required)	F1:<br>F2:							
**ORCCA Objective 3: Improve Prescription Opioid Safety**								
Safer Prescribing/ Dispensing (required)	G1:<br>G2:							
Safer Disposal Practices (optional)	H1:<br>H2:							

Appendix 4. Ohio Action Planning Tools

Decision Aid 1 Matrix

<u>Purpose</u>: To identify existing venues related to ORCCA EBPs

<u>Description</u>: In Ohio, the HCS Intervention Facilitator, one field team (i.e., an HCS-hired Intervention Facilitator, Community Engagement Facilitator, Data Manager) worked with community coalitions, particularly coalition-identified EBP champions to utilize a multi-step Decision Aid Approach across Phase 4 study activities. The first step was to identify existing venues related to ORCCA EBPs. In <u>Step 1: Cataloging Possible Approaches to Population Access,</u> a matrix was used in Decision Aid 1 to map existing locations/methods of current *or potential* population access to priority populations. This allowed communities to inventory potential points of access and also highlighted options that were not available or are already highly represented.

<u>Who completes the tool</u>: Decision Aid 1 is completed by ORCCA EBP coalition champions in collaboration with HCS-hired staff.

<table>
<tr><th colspan="2" rowspan="2">Community “A”: Decision Aid 1</th><th colspan="3">Points of Population Access</th></tr>
<tr><th>CJ</th><th>SSPs</th><th>EDs</th></tr>
<tr><th rowspan="3">Priority Populations</th><th>Prior Opioid Overdose</th><td>ABC drug court
XYZ County Jail</td><td>Abc street exchange</td><td>None</td></tr>
<tr><th>Reduced Opioid Tolerance</th><td>ABC drug court
XYZ County Jail</td><td></td><td>None</td></tr>
<tr><th>Use Other Substances</th><td>ABC drug court
XYZ County Jail</td><td>Abc street exchange</td><td>None</td></tr>
</table>

For peer review only

Decision Aid 2

Purpose: To identify preliminary ORCCA-related goals for each of the three EBPs.

Description: In Step 2: Existing Service Inventory Mapped by Priority Population and Point of Population Access, a similar approach to Decision Aid 1 was employed, but limited to listing *currently available* services (i.e., interventions) specific to each of the three EBPs required by ORCCA. As a first step, a community would use their population access Decision Aid 1 from Step 1 to highlight any setting in which there was a current intervention related to the three ORCCA required objectives. Next, each community would catalog any existing services that address the three ORCCA required objectives using Decision Aids 2A (OEND), 2B (MOUD), and 2C (Safer Prescribing). This approach documents the gaps between potential methods for population access and the degree to which those were already being implemented.

Who completes the tool: Decision Aid 2 is completed by ORCCA EBP coalition champions in collaboration with HCS-hired staff.

**Community "A"- Decision Aid 2B: MOUD**		**Points of Population Access**		
		**CJ**	**SSPs**	**EDs**
**Priority Populations**	**Prior Opioid Overdose**	None	Abc Syringe Exchange- treatment referral	None
	**Reduced Opioid Tolerance**	None	None	None
	**Use Other Substances**	None	Abc Syringe Exchange- treatment referral	None

For peer review only

Decision Aid 3

Purpose: To identify, prioritize, and select EBP strategies.

Description: In Step 3 of the Decision Aid process, communities in collaboration with HCS *brainstormed potential intervention strategy and population access dyads.* This approach allowed for a shared understanding of "what was currently being done" and "where it was being done" in an efficient manner that mapped structurally to ORCCA priority populations and methods of population access. The Decision Aid was not menu-specific, but rather covered strategies for intervention across menus. This provided the foundation for brainstorming which ORCCA EBP strategies *might target existing gaps.* In this step, there is an emphasis on feasibility of implementation.

Discussion Aid 3 was employed in Step 3 and prompted communities to consider whether the identified options involved:

a. bringing an existing service to scale ("1")
b. disseminate an existing service to a new population or method of population access ("2")
c. add a new feature or improvement to an existing service (i.e., changing passive referral to formal linkage intervention); ("3")
d. add a new service; ("4")
e. add both a new method of population access and a new service (e.g., a syringe service program with naloxone distribution); ("5").

Who completes the tool: Decision Aid 3 is completed by ORCCA EBP coalition champions in collaboration with HCS-hired staff.

**Community A-Decision Aid 3: Possible Strategies for Intervention—*no priority order***		**Approach to Population Access**	**Category of Implementation (1-5 above)**
**1**	Distribute OEND	XYZ County Jail	1: bring to scale
**2**	Bridging MOUD	XYZ County Jail	4: new program
**3**	Linkage to Care-Mandated Treatment	ABC Drug Court	1: bring to scale
**4**	Linkage using Peer Navigator	XYZ County Jail	4: new program
**5**	Linkage using Peer Navigator	SSP	3/4: modifying referral program/new program

Decision Aid 4

Purpose: To prioritize EBP strategies by feasibility and expected impact.

Description: This step involved consideration of the intersection between the importance of a given plan and the degree of difficulty in implementing that plan with the notion that EBPs that were both *highly impactful* and *highly feasible* should be undertaken before other actions that are of lower impact and/or lower feasibility. In Decision Aid 4, the identified strategies were categorized by priority/importance (low, medium, high) and by feasibility/capacity (low, medium, high)

Who completes the tool: Decision Aid 4 is completed by ORCCA EBP coalition champions in collaboration with HCS-hired staff.

**Community A- Decision Aid 4: Possible Strategies for Intervention—*feasibility/priority***		**Feasibility/Capacity**		
		Low	Medium	High
**Priority/Importance**	Low	• Linkage to Care Mandated Treatment at ABC Drug Court (*i.e., already at scale with little opportunity to expand*)	• Linkage using peer-navigators at XYZ county Jail (i.e. may move to high priority if bridging MOUD is unsuccessful)	
	Medium		• Distribute OEND at XYZ county jail	
	High	• Bridging MOUD at XYZ county jail	•	• Linkage using peer-navigators at SSP

For peer review only

Decision Aid 5

Purpose: To prioritize and select EBP strategies.

Description: In Step 5: Prioritizing and Selecting EBPs, communities ranked the options outlined in Step 3 in priority order based on the feasibility and impact determinations of Step 4. Coalitions were encouraged to prioritize options that were high impact and high feasibility over interventions that were of low impact or feasibility. Of note, the coalition was not limited in the number of strategies they could target for implementation. This determination was encouraged to be paired with the decision on whether the community and HCS team would pursue a given option for implementation.

Utilizing Step 5 of the Decision Aid process, communities then create their initial action plan specific to their ORCCA goals and the required ORCCA EBPs. The action plan includes the specific strategies selected, the targeted sectors for the intervention, the identified practice settings or venues, preliminary action plans, potential technical assistance or other resource needs, the individual within the Ohio Intervention Design Team (IDT), comprised of academic and subject experts, who will be primarily overseeing and assisting with the implementation of the identified strategy, and additional notes regarding the strategy. For example, if an EBP were to be conducted in an Emergency Department setting, the Emergency Department IDT would oversee the implementation of that EBP and provision of technical assistance.

Who completes the tool: Decision Aid 5 and the Action Plan is completed by ORCCA EBP coalition champions and community coalitions in collaboration with HCS-hired staff.

**Community A-Decision Aid 5: Selected Strategies for Intervention—*priority order***		**Approach to Population Access**	**Target for Action**	**Notes/Preliminary Action Plan**
**1**	Linkage using Peer Navigator	SSP	Yes	Hire program coordinator; select peers
**2**	Bridging MOUD	XYZ County Jail	Yes	
**3**	Distribute OEND	XYZ County Jail	Yes	
**3**	Linkage to Care-Mandated Treatment	ABC Drug Court	No	
**4**	Linkage using Peer Navigator	XYZ County Jail	No	Revisit in 3 months pending exploration of bridging MOUD at XYZ County Jail

For peer review only

Action Plan Template and Sample Completed Action Plan

County: ______________________

Completed By: ___ ______
Initial Completion Date: _____/____/______
Revision #: _____________
Revision Completion Date: _____/________/________

	Specific Strategy	Approach to Population Access (Sector)	Practice Setting or Venue Name	Preliminary Action Plan	Potential TA/Resource Needs	State IDT Lead	Notes
**ORCCA Objective 1: Increase Opioid Overdose Prevention Education and Naloxone Distribution**							
Active OEND (Required)							
Passive OEND (optional)							
**ORCCA Objective 2: Outreach and Delivery of MOUD to High-Risk Populations**							
Expand MOUD treatment (required)							
Linkage to MOUD (required)							
Engagement and retention (required)							
**ORCCA Objective 3: Improve Prescription Opioid Safety**							
Safer Prescribing/ Dispensing (required)							
Safer Disposal Practices (optional)							

review only

Strategy category	Specific Strategy	Approach to Population Access (Sector)	Practice Setting or Venue Name	Preliminary Action Plan	Potential TA/Resource Needs	State IDT Lead	Notes
**ORCCA Objective 1: Increase Opioid Overdose Prevention Education and Naloxone Distribution**							
**Active OEND (required)**	**Active OEND for at-risk individuals and their social networks**<br><br>*Expansion of Project DAWN (a program to provide naloxone to Ohio agencies) to a secondary site*	Behavioral Health	Church	Confirm partner agencies. (HCS)<br><br>Assemble Intervention Design Team/Operations Team to provide implementation assistance. (HCS)	Resources needed on technology/ data collection, billing for sustainability.	[NAME]	Date approved by coalition: DATE
**Active OEND (required)**	**Active OEND for at-risk individuals and their social networks**<br><br>*Expansion of Project DAWN to a secondary site*	Healthcare	[AGENCY] Mobile Unit	Confirm partner agencies. (HCS)<br><br>Assemble Intervention Design Team/Operations Team to provide implementation assistance. (HCS)	Resources needed on technology/ data collection, billing for sustainability.	[NAME]	Date approved by coalition: DATE
**Active OEND (required)**	**Active OEND at high-risk venues:**<br><br>*Naloxone Referral/ Distribution for Drug Court Participants*	Criminal Justice	Drug Court	Confirm partner agencies. (HCS)<br><br>Assemble Intervention Design Team/Operations Team to provide implementation assistance. (HCS)	Potential resources needed on increasing access and participant engagement.	[NAME]	Date approved by coalition: DATE
**Active OEND (required)**	**Active OEND at high-risk venues: "Leave behind"**	Healthcare	Fire/EMS	Confirm partner agencies. (HCS)<br><br>Assemble Intervention Design Team/Operations	Potential resources needed on policy/ implementation logistics, streamlined data collection for naloxone use.	[NAME]	Date approved by

For peer review only

Strategy category	Specific Strategy	Approach to Population Access (Sector)	Practice Setting or Venue Name	Preliminary Action Plan	Potential TA/Resource Needs	State IDT Lead	Notes
	**programs at sites of overdose**<br><br>*Fire/EMS Leave-Behind Program*			Team to provide implementation assistance. (HCS)			coalition: DATE
**OEND self-request (e.g. at pharmacy, community meeting or public health department)**	**Passive OEND**<br><br>Naloxone Distribution at Pharmacies in high risk areas	Healthcare	Pharmacy	Confirm partner agencies. (HCS)<br><br>Assemble Intervention Design Team/Operations Team to provide implementation assistance. (HCS)	Potential resources needed on logistics and possible policy development.	[NAME]	Date approved by coalition: DATE
**ORCCA Objective 2: Outreach and Delivery of MOUD to High-Risk Populations**							
**Expand MOUD treatment (required)**	**Adding/expanding MOUD treatment in Criminal Justice settings**<br><br>*MOUD in the Jail*	Criminal Justice	Jail	Confirm partner agencies. (HCS)<br><br>Assemble Intervention Design Team/Operations Team to provide implementation assistance. (HCS)	Potential resources needed on logistics, billing, etc.	[NAME]	Date approved by coalition: DATE
**Linkage to MOUD**<br><br>**(required)**	**Linkage Programs (all relevant settings)**<br><br>*Expand MOUD referral in ED*	Healthcare	Emergency Department	Confirm partner agencies. (HCS)<br><br>Identify opportunities for expansion of current services.<br><br>Assemble Intervention Design Team/Operations Team to provide implementation assistance. (HCS)	Potential resources needed on sustainability and patient engagement.	[NAME]	Date approved by coalition: DATE

For peer review only

Strategy category	Specific Strategy	Approach to Population Access (Sector)	Practice Setting or Venue Name	Preliminary Action Plan	Potential TA/Resource Needs	State IDT Lead	Notes
**Engagement and retention (required)**	**Reducing barriers to housing, transportation, childcare and accessing other community benefits for people with OUD**<br><br>*Reduce barriers to transportation to improve engagement/retention in treatment*	Behavioral Health	MOUD Treatment Facility<br><br>Potentially other MOUD treatment facilities with limited transportation options. (TBD)	Confirm partner agencies. (HCS)<br><br>Assemble Intervention Design Team/Operations Team to provide implementation assistance. (HCS)<br><br>Engage partners and IDT through an initial planning meeting. (HCS)	Potential resources needed on billing for sustainability.<br><br>Needs: Identify potential partnerships between XXX transportation and treatment agencies	[NAME]	Date approved by coalition: DATE
**ORCCA Objective 3: Improve Prescription Opioid Safety**							
**Safer Prescribing/ Dispensing (required)**	**Safer opioid prescribing for chronic pain**<br><br>Prescriber Accountability through OD Review	Healthcare	Pharmacy	Confirm partner agencies. (HCS)<br><br>Assemble Intervention Design Team/Operations Team to provide implementation assistance. (HCS)<br><br>Engage partners and IDT through an initial planning meeting. (HCS)	Potential resources needed on logistics and identifying allowable information to be shared between the XXX team and XXX Agency.	[NAME]	Date approved by coalition: DATE
**Safer Prescribing/ Dispensing (required)**	**Safer opioid dispensing**<br><br>Utilize the Take Charge Ohio Toolkit for pharmacist and patient education. (Co-dispensing naloxone, providing educational materials	Healthcare	Pharmacy	Engage Education and Awareness workgroup of Scioto County Collaborative Opioid Consortium.<br><br>Determine which parts of the Toolkit will be utilized.<br><br>Confirm partner agencies. (HCS)	Potential resources needed on logistics and possible policy development.	[NAME]	Date approved by coalition: DATE

For peer review only

Strategy category	Specific Strategy	Approach to Population Access (Sector)	Practice Setting or Venue Name	Preliminary Action Plan	Potential TA/Resource Needs	State IDT Lead	Notes
	on opioids and naloxone)			Assemble Intervention Design Team/Operations Team to provide implementation assistance. (HCS)			

For peer review only

BMJ Open

Community-driven selection of strategies to implement evidence-based practices to reduce opioid overdoses in the HEALing Communities Study, a trial to evaluate a community-engaged intervention in Kentucky, Massachusetts, New York, and Ohio

Journal:	*BMJ Open*
Manuscript ID	bmjopen-2021-059328.R2
Article Type:	Protocol
Date Submitted by the Author:	08-Aug-2022

Complete List of Authors:	Young, April; University of Kentucky, Epidemiology<br>Brown, Jennifer; University of Cincinnati<br>Hunt, Timothy; Columbia University<br>Sprague Martinez, Linda; Boston University<br>Chandler, Redonna; National Institutes of Health<br>Oga, Emmanuel; Research Triangle Institute<br>Winhusen, Theresa ; University of Cincinnati Medical Center, Psychiatry<br>Baker, Trevor; Boston Medical Center<br>Battaglia, Tracy; Boston Medical Center<br>Bowers-Sword, Rachel; Boston Medical Center<br>Button, Amy; Albert Einstein College of Medicine, Montefiore Hudson Valley Collaborative<br>Fallin-Bennett, Amanda ; University of Kentucky, College of Nursing<br>Fanucchi, Laura; University of Kentucky<br>Freeman, Patricia ; University of Kentucky College of Pharmacy<br>Glasgow, LaShawn; Research Triangle Institute<br>Gulley, Jennifer; Clark County Health Department<br>Kendell, Charles; Franklin County Agency for Substance Abuse Policy Board<br>Lofwall , Michelle ; University of Kentucky, Center on Drug and Alcohol Research<br>Lyons, Michael S.; Cincinnati Children's Hospital Medical Center<br>Quinn, Maria; Holyoke Medical Center<br>Rapkin, Bruce; Albert Einstein College of Medicine, Epiemiology and Population Health<br>Surratt, Hilary; University of Kentucky<br>Walsh, Sharon ; University of Kentucky

**Primary Subject Heading**:	Addiction
Secondary Subject Heading:	Addiction, Evidence based practice, Public health, Research methods
Keywords:	Substance misuse < PSYCHIATRY, PUBLIC HEALTH, STATISTICS & RESEARCH METHODS

Community-driven selection of strategies to implement evidence-based practices to reduce opioid overdoses in the HEALing Communities Study, a trial to evaluate a community-engaged intervention in Kentucky, Massachusetts, New York, and Ohio

Young AM, Brown JL, Hunt T, Sprague Martinez LS, Chandler R, Oga E, Winhusen TJ, Baker T, Battaglia TA, Bowers-Sword R, Button A, Fallin-Bennett A, Fanucchi L, Freeman PR, Glasgow LM, Gulley J, Kendell C, Lofwall MR, Lyons MS, Quinn M, Rapkin B, Surratt H, Walsh S.L.

Corresponding Lead Author: April M Young (april.young@uky.edu)

For peer review only

Abstract

Introduction: Opioid-involved overdose deaths continue to surge in many communities, despite numerous evidence-based practices (EBPs) that exist to prevent them. The HEALing Communities Study (HCS) was launched to develop and test an intervention (i.e., Communities That HEAL [CTH]) that supports communities in expanding uptake of EBPs to reduce opioid-involved overdose deaths. This paper describes a protocol for a process foundational to the CTH intervention through which community coalitions select strategies to implement EBPs locally.

Methods and analysis: The CTH is being implemented in 67 communities (randomized to receive the intervention) in four states in partnership with coalitions (one per community). Coalitions must select at least five strategies, including one to implement each of the following EBPs: (a) overdose education and naloxone distribution; expanded (b) access to medications for opioid use disorder [MOUD], (c) linkage to MOUD, (d) retention in MOUD; and (e) safer opioid prescribing/dispensing. Facilitated by decision aid tools, the community action planning process includes (1) data-driven goal setting, (2) discussion and prioritization of EBP strategies, (3) selection of EBP strategies, and (4) identification of next steps. Following review of epidemiologic data and information on existing local services, coalitions set goals, and discuss, score, and/or rank EBP strategies based on feasibility, appropriateness within the community context, and potential impact on reducing opioid-involved overdose deaths with a focus on three key sectors (health care, behavioral health and criminal justice) and high-risk/vulnerable populations. Coalitions then select EBP strategies through consensus or majority vote and, subsequently, suggest or choose agencies with which to partner for implementation.

Ethics and dissemination. The HCS protocol was approved by a central IRB (Advarra, Inc) and is registered with Clinical Trials.gov (NCT04111939). Results of the action planning process will be disseminated in academic conferences and peer-reviewed journals, online and print media, and in meetings with community stakeholders.

For peer review only

Strengths and limitations of this study

- The CTH action planning protocol balances scientific rigor with community priorities by ensuring all communities follow a common core of structured activities and phased benchmarks, select from a common menu of strategies for implementing EBPs, and discuss similar considerations as they create their action plan, while also allowing variation across states and communities to best suit the local context.
- The methods used in the CTH action planning process elevates the voices of community members, whose insights about the local community context are critical to implementation success and whose involvement conveys to implementing agencies and local decision-makers that EBP implementation is a priority of the local community and constituents rather than of an outside (federal or academic) entity.
- The action planning protocol requires careful facilitation by coalition members and community staff to ensure that power dynamics do not result in some coalition members having more influence than others in the decision-making process and to manage conflicts of interest (i.e., selection of strategies that could benefit or be perceived to benefit individual coalition members or their agencies).
- The phased and iterative action planning protocol is time-intensive requiring significant commitment from involved community members, faculty, and staff, and may slow implementation of EBPs.
- While necessitated by the constraints of the study timeline, the research teams' intensive involvement in facilitating movement through the action planning protocol may stifle participation by some coalition members who approached the process with deference and thereby limited the extent to which it was community-driven.

For peer review only

Introduction

Opioid-involved overdose deaths remain a public health crisis in many communities worldwide[1 2] although numerous evidence-based practices (EBPs) exist to prevent them. In the Unites States alone, opioid-involved overdose claimed over 490,000 lives between 1999 and 2019.[3] Despite national efforts to change the trajectory of overdose deaths, provisional data indicate over 94,000 total overdose deaths occurred in the 12 months ending in January 2021, making this the highest number of overdose deaths ever recorded in a 12-month period.[4]

Research has demonstrated that medication for opioid use disorder (MOUD), including methadone, buprenorphine, and extended-release naltrexone;[(e.g. 5)] prescription drug monitoring programs and safer prescribing guidelines for prescription opioids;[(e.g. 6)] naloxone, a medication that can reverse an opioid overdose;[(e.g.,7)] and behavioral/psychosocial interventions like recovery support services [(e.g. 5)] can prevent deaths and help people enter and stay in remission and recovery from opioid use disorder. Unfortunately, these EBPs remain under-utilized in many communities leaving significant unmet need for these critically important services.[8-15]

The HEALing Communities Study (HCS), supported by the National Institutes of Health and the Substance Abuse and Mental Health Services Administration, launched in 2019 to develop and test an approach to support communities in expanding uptake and availability of EBPs to address opioid-involved overdose deaths and opioid use disorder (OUD). HCS is a multi-site, parallel-group, cluster randomized wait-list controlled trial involving 67 communities (34 in active intervention and 33 in wait-list control) in Kentucky (KY), Massachusetts (MA), New York (NY), and Ohio (OH).[16] The study is designed to test the Communities That HEAL (CTH) intervention, which contains three components: a community-engaged process of data-driven decision making to select EBP strategies for local implementation and monitoring;[17] a

For peer review only

menu of EBPs and technical assistance guides (Opioid-Overdose Reduction Continuum of Care Approach [ORCCA]),[18] and a set of communication campaigns intended to address stigma and increase knowledge of and demand for EBPs.[19] The CTH intervention is being implemented in the active intervention communities from January 2020 to June 2022 and will be implemented in wait-list control communities from July 2022 to December 2023.

The CTH intervention is grounded in the recognition that communities affected by the opioid overdose crisis possess the best understanding of local strengths, gaps, barriers and facilitators related to local EBP implementation and uptake.[17] Thus, the CTH involves partnerships between academic researchers and community members through community coalitions. Coalitions are comprised of representatives from diverse agencies and institutions as well as individuals focused on tackling complex community issues through collective planning.[20] The CTH intervention employs a phased coalition planning process that was developed based on and expands the Communities that Care model,[17] which has supported communities in the selection of evidence-based prevention efforts in past research.[21-23] Coalition planning models are effective because they allow communities to leverage resources and expertise from across multiple sectors, which can facilitate creative problem solving and innovation, reduce duplication of efforts, and address service gaps.[24 25] The literature points to coalition planning as an effective strategy for implementing EBPs.[26-28]

The purpose of this paper is to describe the protocol for coalition action planning through which EBP strategies are selected to reduce opioid overdose deaths in their communities.[16] The protocol described in this manuscript is relevant beyond the field of substance use, as community engagement and community-driven decision making are recommended for improving responses

For peer review only

to a variety of health challenges,[29-32] including, most recently, SARS-CoV-2 vaccination[33] and Monkeypox.[34]

Methods and Analysis

Participant involvement

As described elsewhere,[17] community engagement is central to the CTH intervention. The CTH intervention places communities, specifically community coalitions, at the center of the decision-making process in creating an action plan for implementing EBP strategies locally. The coalitions are comprised of key stakeholders in healthcare, behavioral health, criminal justice, public health, government, business, housing, and other sectors, and in many cases, include people with lived experience with or impacted by OUD. In some communities, the coalitions that are presently engaged in the CTH existed prior to the HCS, while others were formed specifically to guide the CTH intervention. In addition to driving the CTH intervention, these coalitions facilitate dissemination of information about the study and about EBPs to other local stakeholders. Coalitions, local agency partners, and partnerships formed between academic institutions and local stakeholders will also be key to sustainability of the CTH process and EBPs. Of note, coalitions were not involved in setting the research question, designing the intervention protocol or menu of EBPs, or establishing outcome measures. Of note, each of the four participating states has a Community Advisory Board, with multisectoral representation from across the communities that advises the research teams on study implementation.

Overview of CTH phases

For peer review only

The CTH intervention includes seven phases, described in detail elsewhere,[17] that guide coalitions through a systematic partnership process. The intervention began October 23, 2019. The anticipated trial completion date is December 31, 2023. Briefly, the process begins with establishment or identification of a coalition and an environmental scan, or “landscape analysis,” of local resources and gaps (Phase 0). During Phase 1, coalitions develop a shared charter and identify key community “champions” to support EBPs, data-driven decision making, and communication campaigns that have been described elsewhere.[19] In Phase 2, coalitions learn about data-driven decision making and the ORCCA menus containing EBP strategies related to overdose education and naloxone distribution (OEND, Menu 1), MOUD (Menu 2), and prescription opioid safety (Menu 3). The ORCCA menus have been described in detail elsewhere.[18] Following review of data describing the local epidemiology of OUD and overdose, simulation modeling data, and/or existing service resources and gaps identified by the landscape analysis (Phase 3), coalitions develop community action plans (one per coalition) by selecting strategies to implement EBPs that are in the ORCCA menu (Phase 4). After creation of the action plan, coalition selected EBP strategies are implemented and monitored (Phase 5) and sustainability plans are created (Phase 6). While these processes are ongoing, a health communication campaign is delivered at the community level to reduce stigma and increase engagement in OEND and MOUD.[19] This paper describes the protocol for coalition planning efforts in Phase 4 to select EBP strategies to achieve the study’s primary outcome, reduction in number of opioid overdose deaths. The primary and secondary outcomes of the study and the analysis of the trial data are described in detail elsewhere.[35 36]

Strategies Included in the Community Action Planning Process

For peer review only

Coalitions are provided with guidelines to structure strategy selection in the community action planning process (Figure 1). Coalitions are required to select a minimum of five strategies, including at least one to implement each required EBP from the ORCCA: (a) expansion of proactive offering of OEND to at risk individuals and members of their social networks, (b) expansion of buprenorphine and/or methadone access, (c) improved linkage to MOUD, (d) increased retention in MOUD, and (e) promotion of safer opioid prescribing/dispensing. The EBP strategies are required to include at least one to be implemented in each of the following three sectors: health care, behavioral health and criminal justice.[18] Coalitions are encouraged to consider EBP strategies that would maximize impact in key populations most vulnerable to experiencing an opioid-involved overdose, including those who had a prior opioid overdose;[8 37-40] have reduced opioid tolerance (e.g., completing medically supervised or socially managed withdrawal or release from institutional settings such as jail, residential treatment, hospital);[40-44] use other substances (e.g., alcohol, benzodiazepines, cocaine, and amphetamine like substances);[40 45-49] have concomitant major mental[50-53] or medical illness;[40 54-57] and/or inject drugs.[46 58] Coalitions are also asked to prioritize venues through which these key populations are most likely to be reached. Priority venues for each of the three EBPs (i.e., OEND, MOUD, and safer opioid prescribing and dispensing) are listed in Figure 1 and described in detail elsewhere.[18]

Inherently, the CTH allowed for communities to independently implement EBP strategies at their own pace, including expediting strategies in times of emergency. In June 2020, the HCS approved "fast tracking" certain HCS-supported activities related to OEND in response to the coalitions' concerns about the substantial uptick of opioid overdose observed in the initial months of the SARS-CoV-2 pandemic.[59-66] The protocol allowed HCS-supported naloxone

For peer review only

distribution to begin in high-risk settings prior to the completion of action planning, but preserved the community-driven nature of the decision-making process. Coalitions were asked whether they wanted the HCS teams to provide technical assistance and/or resources to community venues with greatest reach to individuals at risk for overdose (i.e., jails, syringe service programs) to expand OEND. Upon coalition approval, HCS teams and/or community implementation teams began outreach to high-risk venues to expand OEND. Thus, the fast-tracked OEND activities were ongoing during the Phase 4 action planning process. During the action planning process, coalitions were asked to reflect on the successes and challenges of the fast-tracked strategies and asked to decide whether they wanted to continue or expand OEND activities that had been fast-tracked as part of the action planning process.

Strategy selection and action planning process

The core components of the cross-state protocol for action planning and strategy selection are described in Figure 2. Action planning in each state involves (1) data-driven community goal setting, (2) discussion and prioritization of EBP strategies, (3) selection of EBP strategies to include in the action plan, and (4) identification of next steps for implementation of selected EBP strategies.

Coalitions' movement through the action planning process is facilitated by a combination of goal-setting tools, data visualizations and decision aids. Examples of tools developed by each state for the action planning process are provided in the Supplementary Appendix. The communities currently receiving the CTH intervention differ in geographic unit (i.e., counties, townships, cities), community-readiness for EBP strategy implementation, existing resources and gaps, coalition history (e.g., whether coalitions existed prior to the HCS or convened for

For peer review only - http://bmjopen.bmj.com/site/about/guidelines.xhtml

primarily for the HCS), and other contextual factors thereby requiring some variation to the action planning process across and within states (Table 1).

Action planning in each state is driven by coalitions, coalition workgroups, and champions, and is facilitated by a team comprising clinical and research faculty, core staff, and community-based staff with expertise in community-engagement, data, OEND, MOUD, and safer opioid prescribing and dispensing. Coalition composition is described briefly in *Participant Involvement* and in detail elsewhere.[17] Of note, only in MA are *all* agencies to eventually be involved in EBP strategy implementation represented on the coalition. In other states, the agencies to be involved in implementation are represented to varying degrees across coalitions. In NY, community government partners (i.e., health commissioners, county executives) are also involved in the action planning process.

Step 1. Develop Data-driven Community Goals

All coalitions begin the goal-setting process by reviewing community profiles and data dashboards, supplemented in one state (NY) by results of agent-based and systems dynamic modeling. The community profiles, developed through the CTH intervention, included lists of organizations in the community relevant to potential implementation of EBP strategies (e.g., substance use disorder treatment centers, harm reduction programs, emergency response agencies, hospitals). The data dashboards, also developed through the CTH intervention, contain data visualizations on community-specific trends in fatal and non-fatal opioid overdose, MOUD availability, naloxone distribution, and high-risk opioid prescribing.[9]

During and following data review, coalitions discuss strengths, services, gaps, and needs. The coalitions established goals varying in specificity across states and communities (e.g.,

For peer review only

"Increase OEND," "Two emergency departments will initiate MOUD by January 2021"). Coalitions set goals at the ORCCA menu-level (i.e., for OEND, MOUD, and safer opioid prescribing/dispensing) or submenu-level (i.e., for active and passive OEND; for MOUD expansion, linkage, and retention; for safer opioid prescribing/dispensing and disposal). States vary in the other factors their coalitions are asked to consider when setting goals; these factors include sustainability of EBP strategies (NY, MA), EBP strategies' reach to high-risk populations (OH, MA), ability to ensure health equity across ethnic and racial groups (MA, NY), and priority venues for EBP strategy implementation (OH, MA).

Step 2. Discuss and Prioritize EBPs that Align with Community Goals

Following goal setting, coalitions consider specific strategies for implementing the EBPs included on the ORCCA menu and determine which are of highest priority for local implementation. In three states (OH, MA, NY), coalitions brainstorm and free-list EBP strategies that align with the study goals, priority sectors and/or venues, and high-risk populations. In KY, coalitions are provided with initial lists of EBP strategies to consider that are pre-populated from the ORCCA by the academic team. Coalitions in all states consider the EBP strategies in terms of the existing services, size of the gap between existing services and need, feasibility, and potential impact on opioid-involved overdose deaths. To frame the discussion of feasibility more concretely, coalitions in two states (OH, NY) are asked to consider whether the EBP strategy is a new service, scale-up of an existing service via a new method of service delivery, new target population, or new feature to an existing service. In two states (MA, NY), coalitions also consider sustainability. In all states, deliberations on the EBP strategies occur in full coalition meetings or in workgroups led by champions depending on the coalition's preference.

For peer review only

All coalitions begin by providing narrative feedback on each EBP strategy. One state's (KY) coalitions are asked to supplement the narrative feedback by scoring each EBP strategy in terms of size of the existing service gap, feasibility of implementation within the next 12 months, and potential impact on opioid-involved overdose deaths using Likert scales. The scale scores are used to produce a summative priority score to help guide EBP strategy selection. Two states (NY, OH) ask coalitions to rank EBP strategies in terms of impact and feasibility, with the most impactful and feasible EBP strategies scoring highest. In one state (MA), coalitions prioritize EBP strategies through consensus building group dialogues as opposed to ranking.

In three states, coalitions are either required (MA) or encouraged to consider the cost of EBP strategy implementation (NY, OH). In MA, coalitions are provided with an overall budget cap to work within and as part of their planning are required to consider the cost of implementation as they make their EBP strategy selections. In NY, where communities receive funding for staffing and administrative costs and EBP strategy implementation based on population size, coalitions are encouraged, but not required, to consider the cost of EBP strategy implementation as they make their selections. In OH, all communities are provided with fixed and equivalent budgets with limits on expenditures for naloxone and technical assistance; coalitions consider these budget caps as they choose EBP strategies but are not asked to estimate the cost of individual EBP strategies. In KY, coalitions are encouraged *not* to consider cost, but rather to focus on potential need, impact, and feasibility of each EBP strategy.

Step 3. Select EBP strategies to include in the community action plan

In full coalition and coalition subgroup meetings, members review narrative notes, scores, and rankings from the process described above. In two states (KY, OH), coalitions are

For peer review only

also provided with feedback from HCS faculty and staff on implementation feasibility with respect to structural or regulatory barriers. Coalitions weigh these considerations in the context of the guidelines described in Figure 1 and choose through consensus or majority vote the EBP strategies to be implemented with support of HCS. In making their initial selections, coalition members understand that they can later add and refine EBP strategies as capacity expands. Only one state (KY) set a maximum for the number of EBP strategies that could be selected initially, not including OEND strategies already fast-tracked. Coalitions in KY are limited to twelve initial EBP strategies, requiring that they choose no more than three involving OEND, two for MOUD expansion, two for linkage to MOUD, two to improve MOUD retention, and three focused on safer opioid prescribing/dispensing.

Step 4. Identification of next steps for implementation of selected EBP strategies

Once EBP strategies are chosen, coalitions are encouraged to list partner agencies. In three states, coalition-listed agencies are suggested as potential partners for implementation (KY, NY, OH). In MA, the partner agencies for implementation are determined by coalition subgroups as part of the planning process and voted on by the coalitions, with the acceptation of agencies delivering office-based addictions treatment; these agencies were preselected by the study team. Coalition review of selected EBP strategies in one state (KY) also involves members describing what the coalition could do to support rollout (e.g., write op-eds in the local paper in support of the EBP strategy, provide warm handoffs to agencies, etc.) and ways to reach underserved populations with a primary focus on people of color and Spanish speaking individuals. Iteratively with coalition review, HCS faculty and community staff in each state conduct internal reviews. In MA, state government partners are also involved in the internal review. Internal

For peer review only

reviews focus on ensuring the EBP strategies selected adhere to the ORCAA menu and include the required high-risk populations and sectors, potential impact and feasibility. Internal teams also determine next steps for team members, such as initial meetings with partner organizations, contracting considerations, and provision of technical assistance. In one state (MA), the review also includes planning for the mitigation of inequities associated with each EBP strategy and examination of each EBP strategy's cost as estimated by coalitions with technical assistance from community staff and HCS faculty.

Upon completion of the steps described above, descriptions of selected EBP strategies are provided to faculty and staff teams with expertise in implementation science, OEND, MOUD, safer opioid prescribing/dispensing, and/or community engagement to start the process of working with agency partners and explore interest in and capacity for implementation of the coalitions selected EBP strategies. This process varies from site to site and involves the development of an agency specific implementation plan.

Process and Outcome Assessment

Community research staff complete fidelity checklists monthly to document the extent to which all steps are followed and how long each step takes. EBP strategies selected by the coalitions are coded in the form of triads and entered into a harmonized data system shared across the four states. Specifically, the data are coded by venue type (e.g., addiction treatment facility, social services agency, etc), sector (i.e., criminal justice, behavioral health, or healthcare), and ORCCA menu (i.e., OEND, MOUD, or safer opioid prescribing and dispensing). For each entry in the dataset, additional data are recorded and updated throughout the study; these data include but are not limited to the date that the strategy was selected, when it

For peer review only - http://bmjopen.bmj.com/site/about/guidelines.xhtml

was implemented, and whether it was successfully implemented. Analysis of the triad data is the focus of another paper and will involve enumeration of strategies selected across communities and by community characteristics. Data are also being collected on the strategies' reach (e.g., number of naloxone units distributed, number of clients initiated on buprenorphine or involved in care navigation, etc). Cost of the intervention, including the community engagement process, is also being assessed, as described in detail elsewhere. To collect data on coalition members' perspectives on the community engagement process, the HEALing Community Study includes repeated coalition member surveys and qualitative interviews which among many other domains, probes respondents on their experiences with the coalition and strengths and weaknesses of the partnership.[67 68]

Ethics and Dissemination

The HEALing Communities Study protocol was approved by a central Institutional Review Board (IRB; Advarra Inc., Pro00038088) and is being conducted in accordance with The Code of Ethics of the World Medical Association (Declaration of Helsinki). As described elsewhere,[16] because no one person or group of people possess(es) the authority to give consent on behalf of all community members, investigators sought expert consultation and applied guidelines from the Ottawa Statement.[69] Because the CTH intervention poses no more than minimal risk to community members and the research could not be carried out otherwise (see 45.CFR.46.116), a waiver of informed consent was obtained for all community members who may be affected by the CTH intervention.

The action planning tools are disseminated to coalition members in hardcopy, via email, and/or through a password-protected, online portal. We plan to disseminate updated versions of

For peer review only

these tools to communities currently in the waitlist-control group via the same mechanisms when intervention begins in their communities. Results of the action planning process (i.e., EBP strategy selections) are shared with community members, community agencies, and community advisory boards through meetings, online and print media, and presentations to community stakeholder groups in the intervention communities. In the future, we also plan to disseminate our findings at relevant conferences, meetings and through peer-reviewed journals. Finally, we will disseminate our findings, manuals, toolkits, and other resources through federal partners including the National Institutes of Health, Substance Abuse and Mental Health Services Administration and the Centers for Disease Control and Prevention.

For peer review only

Table 1. Characteristics of action planning process that were unique to certain states

Action Planning Steps	Characteristics	KY	MA	NY	OH
Step 1. Develop Data-driven Community Goals	*Supplementary factors considered by coalitions:*				
	• Agent-based and systems dynamic modeling			X	
	• EBP strategy sustainability		X	X	
	• Reach to high-risk populations and priority venues		X		X
	• Equity across ethnic and racial groups		X	X	
Step 2. Discuss and Prioritize EBP strategies	Development of EBP strategy list considered by coalition	Pre-populated by the academic team	Generated by coalitions[1]	Generated by coalitions[1]	Generated by coalitions[1]
	Method of prioritizing strategies	Likert scale ratings of size of current service gap, feasibility, and potential impact.	Consensus building group dialogues	Rank by impact and feasibility	Rank by impact and feasibility
	Coalition consideration of cost	Encouraged *not* to consider cost, but to focus on need, impact, and feasibility of each EBP strategy.	Provided with budget cap and required to consider costs	Encouraged, but not required	Encouraged, but not required
	Supplementary factors considered by coalitions:				
	• EBP strategy sustainability		X	X	
	• Method of EBP expansion (i.e., new service, or scale-up of an existing service via a new method of delivery, target population, or feature)			X	X

For peer review only - http://bmjopen.bmj.com/site/about/guidelines.xhtml

Action Planning Steps	Characteristics	KY	MA	NY	OH
Step 3. Select EBP strategies to include in action plan	Limits on strategy selections	12 initial EBP strategies	No limit	No limit	No limit
Step 4. Identify next steps for implementation of selected EBP strategies	Coalition input on agencies to be involved in implementation	Coalitions suggest potential agencies to involve in implementation.	Coalition subgroups determine which agencies will be involved in implementation.	Coalitions suggest potential agencies to involve in implementation.	Coalitions suggest potential agencies to involve in implementation.
	Coalition input on next steps for beginning implementation	Coalition describes what they can do to support implementation and reach underserved populations who may experience disparities in access.	Coalitions estimate cost of each strategy with technical assistance from the academic team.	None beyond action plan established in Step 3	None beyond action plan established in Step 3

EBP: evidence-based practices

[1] "Coalitions" is inclusive of full coalitions, coalition workgroups, and coalition champions with expertise in specific EBPs

Authors' contributions

The following individuals contributed to the design of the action planning protocol described in this manuscript: AY, PRF, LSM, LF, MRL, JB, MSL, TH, AB, BR, RBS, and TAB. The following individuals made substantial contributions to the implementation of the action planning protocol: AY, PRF, AF, LF, JG, CK, MRL, HS, SLW, JB, MSL, TH, AB, and MQ. The following individuals made substantial contributions to the drafted work and/or substantively revised it: AY, JB, TH, LSM, RC, EO, TW, TAB, TB, RBS, AB, AF, LF, LMG, PRF, JG, CK, MSL, MRL, MQ, BR, HS, SLW. All authors read and approved the final manuscript and agree to be accountable for all aspects of the work.

Funding statement

This research was supported by the National Institutes of Health through the NIH HEAL (Helping to End Addiction Long-term^{SM}) Initiative under award numbers UM1DA049394, UM1DA049406, UM1DA049412, UM1DA049415, UM1DA049417 (ClinicalTrials.gov Identifier: NCT04111939). The Substance Abuse and Mental Health Services Administration (SAMHSA) is a non-funding sponsor of the study. This study protocol (Pro00038088) was approved by Advarra Inc., the HEALing Communities Study single Institutional Review Board. We wish to acknowledge the participation of the HEALing Communities Study communities, community coalitions, and Community Advisory Boards and state government officials who partnered with us on this study. The content is solely the responsibility of the authors and does not necessarily represent the official views of the National Institutes of Health, the Substance Abuse and Mental Health Services Administration or the NIH HEAL Initiative^{SM}.

Competing interest statement

Competing interest disclosures include the following: LSM is a consultant for the Boston Public Health Commission, Action for Boston Area development and the Young Foundation, SLW has served as a scientific advisor to Opiant Pharmaceuticals, Titan Pharmaceuticals, Astra Zeneca, Cerevel, Arbor Pharmaceuticals, Brainsway, Summit Biosciences, Trevi and Camurus. JB ad ML receive investigator-initiated support paid to the institution from Gilead Sciences, Inc. MRL has consulted for Titan Pharmaceuticals and Camurus. AFB is the Program Director of Voices of Hope, a University of Kentucky HCS partner organization. PRF, HS, AY, EO, LMG, RC, JG, CK and LCF have no competing interests.

Data Sharing Statement

The protocol described in this manuscript is for a community-engaged action planning process by which communities choose strategies to implement evidence-based practices in their communities. Data for this paper are considered to be the tools such as decision aids, action plans, and other documents used to facilitate the process and are included as a Supplementary Appendix. The protocol described in this manuscript is part of a larger trial for which data are of a different type; those data are described in the trial registry.

Acknowledgments

We wish to acknowledge the participation of the HEALing Communities Study communities, community coalitions, and Community Advisory Boards and state government officials who partnered with us on this study. We extend our sincere gratitude to the staff and community

members who have dedicated their time and passion to this project and without whom the HEALing Communities Study would not be possible.

For peer review only

References

1. Krausz RM, Westenberg JN, Ziafat K. The opioid overdose crisis as a global health challenge. *Current Opinion in Psychiatry* 2021;34(4):405-12.

2. World Health Organization. Opioid overdose 2021 [updated August 4 2021. Available from: https://www.who.int/news-room/fact-sheets/detail/opioid-overdose accessed August 15 2021.

3. National Institute on Drug Abuse. Overdose Death Rates Bethesda, MD2021 [updated January 29, 2021. Available from: https://www.drugabuse.gov/drug-topics/trends-statistics/overdose-death-rates accessed August 15 2021.

4. Ahmad FB RL, Sutton P. Provisional drug overdose death counts: National Center for Health Statistics. Designed by LM Rossen, A Lipphardt, FB Ahmad, JM Keralis, and Y Chong: National Center for Health Statistics., 2021.

5. Strang J, Volkow ND, Degenhardt L, et al. Opioid use disorder. *Nature Reviews Disease Primers* 2020;6(1):3. doi: 10.1038/s41572-019-0137-5

6. Bohnert AS, Guy Jr GP, Losby JL. Opioid prescribing in the United States before and after the Centers for Disease Control and Prevention's 2016 opioid guideline. *Annals of internal medicine* 2018;169(6):367-75.

7. Walley AY, Xuan Z, Hackman HH, et al. Opioid overdose rates and implementation of overdose education and nasal naloxone distribution in Massachusetts: interrupted time series analysis. *British Medical Journal* 2013;346

8. Larochelle MR, Bernson D, Land T, et al. Medication for opioid use disorder after nonfatal opioid overdose and association with mortality: a cohort study. *Annals of internal medicine* 2018;169(3):137-45.

9. Wu E, Villani J, Davis A, et al. Community dashboards to support data-informed decision-making in the HEALing communities study. *Drug and alcohol dependence* 2020;217:108331-31. doi: 10.1016/j.drugalcdep.2020.108331

10. Welty L, Harrison A, Abram K, et al. Substance Abuse and Mental Health Services Administration.(2017). Key substance use and mental health indicators in the United States: Results from the 2016 National Survey on Drug Use and Health (HHS Publication No. SMA 17-5044, NSDUH Series H-52). Rockville, MD: Center for Behavioral Health Statistics and Quality. *Substance Abuse and Mental Health Services Administration Retrieved College of Health Sciences* 2019;106(5):128.

11. Williams AR, Nunes EV, Bisaga A, et al. Developing an opioid use disorder treatment cascade: A review of quality measures. *Journal of substance abuse treatment* 2018;91:57-68.

For peer review only

12. Freeman PR, Hankosky ER, Lofwall MR, et al. The changing landscape of naloxone availability in the United States, 2011–2017. *Drug and alcohol dependence* 2018;191:361-64.

13. Guy Jr GP, Zhang K, Bohm MK, et al. Vital signs: changes in opioid prescribing in the United States, 2006–2015. *MMWR Morb Mortal Wkly Rep* 2017;66(26):697.

14. Guy Jr GP, Zhang K, Halpin J, et al. An examination of concurrent opioid and benzodiazepine prescribing in 9 states, 2015. *American journal of preventive medicine* 2019;57(5):629-36.

15. Guy GP, Zhang K, Schieber LZ, et al. County-level opioid prescribing in the United States, 2015 and 2017. *JAMA internal medicine* 2019;179(4):574-76.

16. Walsh SL, El-Bassel N, Jackson RD, et al. The HEALing (Helping to End Addiction Long-term SM) Communities Study: Protocol for a cluster randomized trial at the community level to reduce opioid overdose deaths through implementation of an integrated set of evidence-based practices. *Drug and Alcohol Dependence* 2020;217:108335.

17. Martinez LS, Rapkin BD, Young A, et al. Community engagement to implement evidence-based practices in the HEALing Communities Study. *Drug and Alcohol Dependence* 2020;217:108326.

18. Winhusen T, Walley A, Fanucchi LC, et al. The opioid-overdose reduction continuum of care approach (ORCCA): evidence-based practices in the HEALing communities study. *Drug and Alcohol Dependence* 2020;217:108325.

19. Lefebvre RC, Chandler RK, Helme DW, et al. Health communication campaigns to drive demand for evidence-based practices and reduce stigma in the HEALing Communities Study. *Drug and alcohol dependence* 2020;217:108338.

20. Rosenthal BB, Greenawalt J, Ivery JM, et al. Coalitions: Essential tools for organizing. In: Staples L, ed. Roots to Power: A Manual for Grassroots Organizing: A Manual for Grassroots Organizing. London: Praeger 2016:297.

21. Oesterle S, Kuklinski MR, Hawkins JD, et al. Long-Term Effects of the Communities That Care Trial on Substance Use, Antisocial Behavior, and Violence Through Age 21 Years. *Am J Public Health* 2018;108(5):659-65.

22. Shapiro VB, Hawkins JD, Oesterle S. Building local infrastructure for community adoption of science-based prevention: The role of coalition functioning. *Prevention Science* 2015;16(8):1136-46.

23. Arthur MW, Hawkins JD, Brown EC, et al. Implementation of the Communities That Care prevention system by coalitions in the Community Youth Development Study. *Journal of Community Psychology* 2010;38(2):245-58.

24. Butterfoss FD. Coalitions and partnerships in community health: John Wiley & Sons 2007.

For peer review only

25. Wandersman A, Goodman RM, Butterfoss FD. Understanding coalitions and how they operate. In: Minkler M, ed. Community organizing community building for health. 2 ed. New Brunswick, NJ Rutgers University Press 1997:261-77.

26. Powell BJ, McMillen JC, Proctor EK, et al. A compilation of strategies for implementing clinical innovations in health and mental health. *Medical care research review* 2012;69(2):123-57.

27. Rugs D, Hills HA, Moore KA, et al. A community planning process for the implementation of evidence-based practice. *Evaluation and Program Planning* 2011;34(1):29-36.

28. Pellecchia M, Mandell DS, Nuske HJ, et al. Community–academic partnerships in implementation research. *Journal of Community Psychology* 2018;46(7):941-52.

29. Centers for Disease Control and Prevention. Principles of Community Engagement. Second ed, 2011.

30. World Health Organization. WHO community engagement framework for quality, people-centred and resilient health services. : World Health Organization, 2017.

31. World Health Organization. Community engagement: a health promotion guide for universal health coverage in the hands of the people . Geneva: World Health Organization,,, 2020.

32. C. D. I. Study Group. Community-directed interventions for priority health problems in Africa: results of a multicountry study. *Bull World Health Organ* 2010;88(7):509-18. doi: 10.2471/BLT.09.069203 [published Online First: 2009/12/01]

33. World Health Organization & United Nations Children's Fund (UNICEF). Conducting community engagement for COVID-19 vaccines: interim guidance, 31 January 2021: World Health Organization, 2021.

34. US Department of Health and Human Services. Biden-Harris Administration Bolsters Monkeypox Response; HHS Secretary Becerra Declares Public Health Emergency. Washington, D.C., 2022.

35. Slavova S, LaRochelle MR, Root ED, et al. Operationalizing and selecting outcome measures for the HEALing Communities Study. *Drug and alcohol dependence* 2020;217:108328.

36. Westgate PM, Cheng DM, Feaster DJ, et al. Marginal modeling in community randomized trials with rare events: Utilization of the negative binomial regression model. *Clinical Trials* 2022:17407745211063479.

37. Winhusen T, Theobald J, Lewis D, et al. Development and initial testing of a tailored telephone intervention delivered by peers to prevent recurring opioid-overdoses (TTIP-PRO). *Health education research* 2016;31(2):146-60. doi: 10.1093/her/cyw010

For peer review only

38. Caudarella A, Dong H, Milloy MJ, et al. Non-fatal overdose as a risk factor for subsequent fatal overdose among people who inject drugs. *Drug Alcohol Depend* 2016;162:51-5. doi: 10.1016/j.drugalcdep.2016.02.024

39. Darke S, Mills KL, Ross J, et al. Rates and correlates of mortality amongst heroin users: findings from the Australian Treatment Outcome Study (ATOS), 2001-2009. *Drug Alcohol Depend* 2011;115(3):190-5. doi: 10.1016/j.drugalcdep.2010.10.021 [published Online First: 2010/12/07]

40. Larochelle MR, Bernstein R, Bernson D, et al. Touchpoints - Opportunities to predict and prevent opioid overdose: A cohort study. *Drug and alcohol dependence* 2019;204:107537-37. doi: 10.1016/j.drugalcdep.2019.06.039 [published Online First: 2019/09/03]

41. Strang J, McCambridge J, Best D, et al. Loss of tolerance and overdose mortality after inpatient opiate detoxification: follow up study. *British Medical Journal* 2003;326(7396):959-60. doi: 10.1136/bmj.326.7396.959

42. Binswanger IA, Stern MF, Deyo RA, et al. Release from prison—a high risk of death for former inmates. *New England Journal of Medicine* 2007;356(2):157-65. doi: 10.1056/NEJMsa064115

43. Merrall ELC, Kariminia A, Binswanger IA, et al. Meta-analysis of drug-related deaths soon after release from prison. *Addiction* 2010;105(9):1545-54. doi: 10.1111/j.1360-0443.2010.02990.x

44. Walley AY, Lodi S, Li Y, et al. Association between mortality rates and medication and residential treatment after in-patient medically managed opioid withdrawal: a cohort analysis. *Addiction (Abingdon, England)* 2020;115(8):1496-508. doi: 10.1111/add.14964 [published Online First: 2020/02/25]

45. Sun EC, Dixit A, Humphreys K, et al. Association between concurrent use of prescription opioids and benzodiazepines and overdose: retrospective analysis. *British Medical Journal* 2017;356:j760-j60. doi: 10.1136/bmj.j760

46. Brugal MT, Barrio G, De LF, et al. Factors associated with non-fatal heroin overdose: assessing the effect of frequency and route of heroin administration. *Addiction* 2002;97(3):319-27. doi: 10.1046/j.1360-0443.2002.00058.x [published Online First: 2002/04/20]

47. Cho J, Spence MM, Niu F, et al. Risk of Overdose with Exposure to Prescription Opioids, Benzodiazepines, and Non-benzodiazepine Sedative-Hypnotics in Adults: a Retrospective Cohort Study. *J Gen Intern Med* 2020;35(3):696-703. doi: 10.1007/s11606-019-05545-y [published Online First: 2020/01/09]

48. Gladden RM, O'Donnell J, Mattson CL, et al. Changes in Opioid-Involved Overdose Deaths by Opioid Type and Presence of Benzodiazepines, Cocaine, and Methamphetamine - 25

For peer review only

States, July-December 2017 to January-June 2018. *MMWR Morb Mortal Wkly Rep* 2019;68(34):737-44. doi: 10.15585/mmwr.mm6834a2

49. Park TW, Larochelle MR, Saitz R, et al. Associations between prescribed benzodiazepines, overdose death and buprenorphine discontinuation among people receiving buprenorphine. *Addiction (Abingdon, England)* 2020;115(5):924-32. doi: 10.1111/add.14886 [published Online First: 2020/01/20]

50. O'Driscoll PT, McGough J, Hagan H, et al. Predictors of accidental fatal drug overdose among a cohort of injection drug users. *American journal of public health* 2001;91(6):984-87. doi: 10.2105/ajph.91.6.984

51. Pabayo R, Alcantara C, Kawachi I, et al. The role of depression and social support in non-fatal drug overdose among a cohort of injection drug users in a Canadian setting. *Drug and alcohol dependence* 2013;132(3):603-09. doi: 10.1016/j.drugalcdep.2013.04.007 [published Online First: 2013/05/04]

52. Wines JD, Jr., Saitz R, Horton NJ, et al. Overdose after detoxification: a prospective study. *Drug Alcohol Depend* 2007;89(2-3):161-9. doi: 10.1016/j.drugalcdep.2006.12.019 [published Online First: 2007/02/07]

53. Tobin KE, Latkin CA. The relationship between depressive symptoms and nonfatal overdose among a sample of drug users in Baltimore, Maryland. *J Urban Health* 2003;80(2):220-29. doi: 10.1093/jurban/jtg025

54. Bosilkovska M, Walder B, Besson M, et al. Analgesics in patients with hepatic impairment: pharmacology and clinical implications. *Drugs* 2012;72(12):1645-69. doi: 10.2165/11635500-000000000-00000 [published Online First: 2012/08/08]

55. Green TC, McGowan SK, Yokell MA, et al. HIV infection and risk of overdose: a systematic review and meta-analysis. *AIDS* 2012;26(4):403-17. doi: 10.1097/QAD.0b013e32834f19b6

56. Jolley CJ, Bell J, Rafferty GF, et al. Understanding Heroin Overdose: A Study of the Acute Respiratory Depressant Effects of Injected Pharmaceutical Heroin. *PLoS One* 2015;10(10):e0140995-e95. doi: 10.1371/journal.pone.0140995

57. Vu Q, Beselman A, Monolakis J, et al. Risk factors for opioid overdose among hospitalized patients. *J Clin Pharm Ther* 2018;43(6):784-89. doi: 10.1111/jcpt.12701 [published Online First: 2018/05/26]

58. Bazazi AR, Zelenev A, Fu JJ, et al. High prevalence of non-fatal overdose among people who inject drugs in Malaysia: Correlates of overdose and implications for overdose prevention from a cross-sectional study. *International Journal on Drug Policy* 2015;26(7):675-81. doi: 10.1016/j.drugpo.2014.11.010

For peer review only

59. Slavova S, Quesinberry D, Hargrove S, et al. Trends in Drug Overdose Mortality Rates in Kentucky, 2019-2020. *JAMA network open* 2021;4(7):e2116391-e91. doi: 10.1001/jamanetworkopen.2021.16391

60. Slavova S, Rock P, Bush HM, et al. Signal of increased opioid overdose during COVID-19 from emergency medical services data. *Drug and alcohol dependence* 2020;214:108176-76. doi: 10.1016/j.drugalcdep.2020.108176

61. Stephenson J. Drug Overdose Deaths Head Toward Record Number in 2020, CDC Warns. *JAMA Health Forum* 2020;1(10):e201318-e18. doi: 10.1001/jamahealthforum.2020.1318

62. Wakeman SE, Green TC, Rich J. An overdose surge will compound the COVID-19 pandemic if urgent action is not taken. *Nature Medicine* 2020;26(6):819-20. doi: 10.1038/s41591-020-0898-0

63. Holland KM, Jones C, Vivolo-Kantor AM, et al. Trends in US emergency department visits for mental health, overdose, and violence outcomes before and during the COVID-19 pandemic. *JAMA psychiatry* 2021;78(4):372-79.

64. Friedman J, Beletsky L, Schriger DL. Overdose-related cardiac arrests observed by emergency medical services during the US COVID-19 epidemic. *JAMA psychiatry* 2021;78(5):562-64.

65. Centers for Disease Control and Prevention. Overdose Deaths Accelerating During COVID-19 2020 [Available from: https://www.cdc.gov/media/releases/2020/p1218-overdose-deaths-covid-19.html accessed May 19 2021.

66. Centers for Disease Control and Prevention. Increase in Fatal Drug Overdoses Across the United States Driven by Synthetic Opioids Before and During the COVID-19 Pandemic: CDC Health Alert Network, 2020.

67. Aldridge AP, Barbosa C, Barocas JA, et al. Health economic design for cost, cost-effectiveness and simulation analyses in the HEALing Communities Study. *Drug and alcohol dependence* 2020;217:108336.

68. Knudsen HK, Drainoni M-L, Gilbert L, et al. Model and approach for assessing implementation context and fidelity in the HEALing Communities Study. *Drug and alcohol dependence* 2020;217:108330.

69. Taljaard M, Weijer C, Grimshaw JM, et al. The Ottawa Statement on the ethical design and conduct of cluster randomised trials: precis for researchers and research ethics committees. *British Medical Journal* 2013;346:f2838. doi: 10.1136/bmj.f2838

For peer review only

Figure 1. Guidelines for HEALing Communities Study Community Action Planning Process to reduce opioid overdose deaths through overdose education and naloxone distribution, expansion of medications for opioid use disorder, and safer opioid prescribing and dispensing

For peer review only

Figure 2. Core components of the HEALing Communities Study Community Action Planning Process to reduce opioid overdose deaths through overdose education and naloxone distribution, medications for opioid use disorder, and safer opioid prescribing and dispensing

For peer review only

- Coalitions *must* select a minimum of five strategies for implementing evidence-based practices (EBPs) from the ORCCA menus including one involving each of the following
  - ORCCA Menu 1: Active OEND (i.e., proactively offer OEND to those at risk)
  - ORCCA Menu 2A: Expand MOUD access
  - ORCCA Menu 2B: Increase linkage to MOUD
  - ORCCA Menu 2C: Improve retention in MOUD
  - ORCCA Menu 3: Safer opioid prescribing/dispensing

- Priority sectors: Selected EBP strategies *must* include at least one to be implemented in each of the following:
  - Healthcare (i.e., outpatient healthcare centers, pre-hospital providers, emergency departments and urgent care, hospitals, primary care settings, public health departments, and pharmacies)
  - Behavioral health (i.e., substance use disorder and mental health treatment centers and social service agencies, including syringe service programs)
  - Criminal justice (i.e., pre-trial, jails, probation, parole, drug and problem-solving courts, police and "narcotics" task forces, halfway houses, community-based correctional facilities, and department of youth services)

- Key populations: Coalitions are encouraged to consider EBP strategies focused on those most vulnerable to opioid overdose:
  - People with a prior opioid overdose
  - People with reduced opioid tolerance
  - People who use other substances
  - People who have concomitant major mental illness
  - People who have a concomitant major medical illness
  - People who inject drugs

- Priority venues: Coalitions are encouraged to consider EBP strategies to be implemented in priority settings:
  - OEND: criminal justice settings, syringe service programs, emergency departments and acute care hospitals, sites of overdose for naloxone leave-behind, and mental health/addiction treatment programs
  - MOUD: criminal justice settings, primary care, general medical and behavioral/mental health settings, specialty addiction/substance use disorder treatment settings, and recovery programs
  - Safer opioid prescribing and dispensing: inpatient service, emergency/urgent care, outpatient clinics, ambulatory surgery, dental clinics, pharmacies

Figure 1. Guidelines for HEALing Communities Study Community Action Planning Process to reduce opioid overdose deaths through overdose education and naloxone distribution, expansion of medications for opioid use disorder, and safer opioid prescribing and dispensing

165x210mm (330 x 330 DPI)

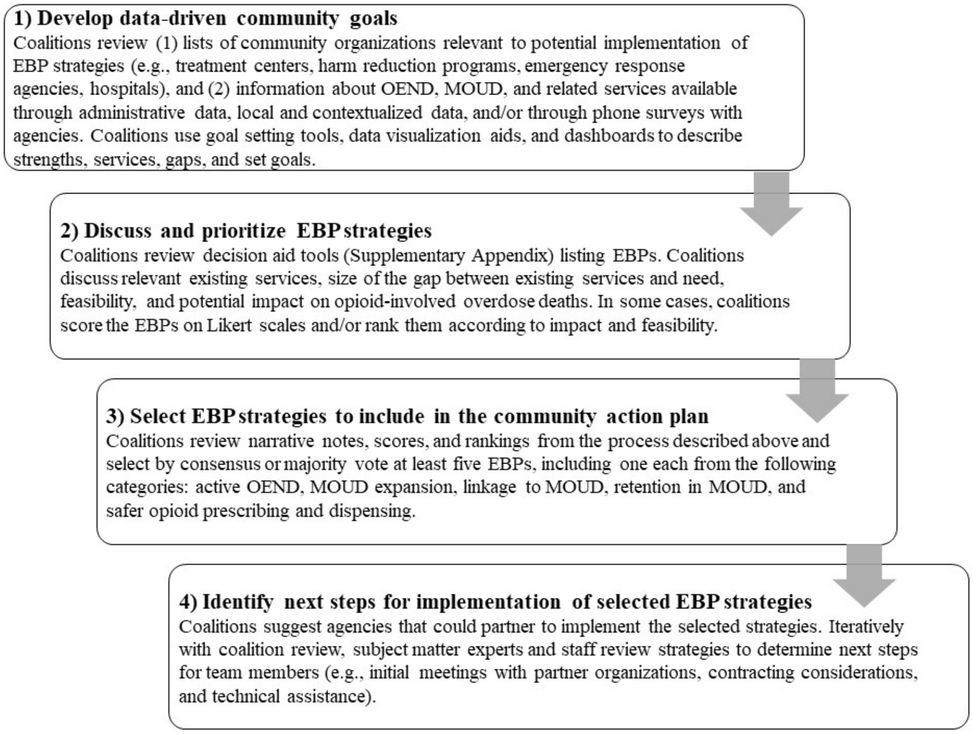

Figure 2. Core components of the HEALing Communities Study Community Action Planning Process to reduce opioid overdose deaths through overdose education and naloxone distribution, medications for opioid use disorder, and safer opioid prescribing and dispensing

254x190mm (96 x 96 DPI)

Appendix 1. Kentucky Action Planning Tools

Community Goal Setting Tool

Purpose: To describe existing infrastructure, gaps, opportunities, needs, and goals related to opioid overdose prevention.

Description: The Kentucky Community Engagement and Continuum of Care teams partnered to facilitate communities' identification of their strengths and opportunities for expansion related to opioid overdose prevention followed by thoughtful consideration and selection of the ORCCA strategies. The process began with the Community Engagement team working with the coalition to describe existing infrastructure, gaps, opportunities, needs, and goals related to opioid overdose prevention in their communities using a Community Goal Setting Tool. The coalitions and/or menu-specific workgroups worked on completing the tool for each menu separately and presenting them to the full coalition.

Who completes the tool: Coalitions and/or coalition workgroups with facilitation by faculty and staff.

Community Goal Setting Tool — Menu 1

Menu 1 Goal: Increase Opioid Overdose Prevention Education and Naloxone Distribution (OEND)

Brainstorm! Think about your community: what you've experienced, what you learned from your community profile and data dashboard, and what you envision for the future. Answer the questions below and jot down your thoughts

What are we doing well? What OEND services already exist?

Ex. There is active Opioid Overdose Education and Naloxone Distribution (OEND) already occurring in the County Detention Center. The Detention Center is currently able to provide naloxone to all persons released from the facility (as part of partnership with the HEALing Communities Study) and is also partnering with other organizations to distribute naloxone to anyone who is interested and completes the requisite virtual training.

Who needs OEND in our community?

Ex. There is still a significant need for distribution among the peer community and their social networks, in homes with bystanders, and with medical and law enforcement professionals in contact with those at high-risk for overdose.

Where in our community should OEND services be provided?

Ex. There is significant opportunity for OEND expansion in Emergency Departments and the healthcare system generally, in pharmacies, with local Quick Response Teams (QRTs), in Substance Use Disorder (SUD) treatment facilities, and in other locations where the peer community can be found. The NKY Health Department also has capacity to expand OEND services.

Community OEND Goals:

Example:

1. Expand OEND in criminal justice settings, including Probation & Parole and Drug Court.
2. Expand OEND in healthcare settings, including Emergency Departments and Substance Use Disorder (SUD) treatment programs.
3. Expand OEND access among peer community and their support systems (via recovery support groups, social service agencies, and in "hot spots" frequented by those at high-risk, etc.).
4. Expand OEND with local QRTs.
5. Increase first responders carrying/administering naloxone.

Decision Aid Tool

Purpose: To facilitate discussion and prioritization of evidence-based practice strategies that align with community goals and the ORCCA

Description: HCS staff then populated menu-specific Decision Aid Tools with strategies listed in the ORCCA and TAG, integrating coalition's suggestions for specific strategies when available from Community Goal Setting Tool. In meetings facilitated by faculty and staff with expertise in community-engagement and the continuum of care, the coalitions and/or menu-specific workgroups reviewed each possible strategy one-by-one, offering a narrative description of factors that they believed would be relevant to deciding whether to implement the strategy (i.e., current relevant activities, barriers they anticipate encountering, agencies relevant to implementation). After offering a narrative description, the coalition scored on Likert scales the size of the current gap in the service/strategy (4-point scale; 0-3), feasibility of implementation within the next 12 months (4-point scale; 0-3), and its potential impact on overdose deaths (3-point scale; 1-3). Each dimension was scored through consensus or through in-meeting polling. Coalitions did not use specific criteria or thresholds in assigning the scores, rather they often scored them based on how they compared to others on the menu. Scores were summed to produce an overall "priority score" for each strategy.

Who completes the tool: Coalitions and/or coalition workgroups with facilitation by faculty and staff.

For peer review only

Menu 2: Strategies to Enhance Delivery of MOUD Maintenance Treatment, Including Agonist/Partial Agonist Medication					
**Strategy and Venue**	**Current Activity**	**Size of Gap** (Current Activity vs. Need)	**Feasibility in <12 Months**	**Impact on Overdose Deaths**	**Priority Score**
Working with the ORCCA menu, brainstorm possible strategies to meet the Community MOUD goals. Record all strategies in this column.	Use this column to briefly summarize current activity in your community	0. None<br>1. Small<br>2. Medium<br>3. Large	0. Extremely Low<br>1. Small<br>2. Medium<br>3. Large	1. Small<br>2. Medium<br>3. Large	Add previous 3 columns; if any column contains 0, priority score is 0
**a) Expand MOUD Treatment Availability (Capacity Building) – REQUIRED**					
**Add/expand MOUD in primary care, other general medical/BH settings, and specialty addiction/SUD & recovery programs**					
Ex. Expand Methadone in county OTPs 2.a.1.2	Ex. Have two OTP providers but one has long wait times; need to figure out what they need to be able to treat more patients				
**Add/expand MOUD treatment in Criminal Justice Settings (pre-trial, jail, prison, probation, parole)**					
Ex. Expand prescription of XR naltrexone and buprenorphine within jail	Ex. Interest in XR naltrexone at the jail and jail might be open but have a lot of concerns/barriers				
**Expand access to MOUD through telemedicine, interim buprenorphine or methadone, or medication units**					
Ex. Expand use of Telemedicine/telehealth/tele-consults for buprenorphine treatment	Ex. Telehealth opportunities have grown due to COVID 19, but it is unknown whether this option will remain available.				
**b) Interventions to Link to MOUD – REQUIRED**					
**Linkage Programs (All Relevant settings)**					
Ex. Develop linkage program for incarcerated individuals about to be released (with peer support and/or other staff)	Ex. No current activity, the jail is somewhat interested but there are several barriers				
**Bridging MOUD medications as Linkage Adjunct (all relevant settings)**					
Ex. Expand bridge programs within county ER and inpatient settings	Ex. An existing bridge program is working well and can use as model for others. Dificulty with staffing & infrastructure is main barrier				
**c) MOUD Treatment Engagement and Retention – REQUIRED**					
**Enhancement of clinical delivery approaches that support engagement and retention**					
Ex. Expand recovery support services (peer support, telephone recovery support, recovery coaching, family support) in criminal justice settings (Voices of Hope)	Ex. Add peer supports within system; no current long term supports, or programs/mentorship programs engaged				
**Use virtual retention approaches (e.g. mobile, web, digital therapeutics)**					
Ex. Implement use of re-SET-0 app to improve retention, incentives, and education 2.c.2	Ex. Some clinics within county already using it; potential cost barriers and potential disinterest from buprenorphine providers				
**Utilize retention care coordinators**					
Ex. Utilize retention care navigators to increase/add care navigators in all MOUD treatment settings 2.c.3	Ex. Other care navigation efforts are already underway that could be leveraged, and there is a desire to increase support of and leverage local efforts where possible				
**Integrate mental health and polysubstance use treatment into MOUD care**					
Ex. Offer regular training between primary care/non MH providers with addiction psychiatry and other MH providers to manage co-morbidity with OUD	Ex. Unsure if providers would be interested in engaging				
**Reduce barriers to housing, transportation, childcare, and access to other community benefits for People with OUD**					
Expand transportation services/offerings to align with MOUD treatment center services (location, hours, etc.)	Ex. Local agency offers transportation services to clients				

Action Planning Tool

<u>Purpose</u>: To facilitate discussion and final strategy selection.

<u>Description</u>: The list of strategies discussed in the Decision Aid Tool was used to pre-populate an Action Planning Tool. The tool listed all strategies with their scores and new implementation feasibility ratings (High, Medium, Low) provided by faculty with expertise in implementation science and the continuum of care based on their knowledge of policy- and payor-level barriers and time required for scale up. The pre-populated Action Planning Tools were presented to coalitions for discussion and strategy selection through consensus.

<u>Who completes the tool</u>: Coalitions and/or coalition workgroups with facilitation by faculty and staff.

Action Planning Tool — Menu 3

For each strategy from the Decision Aid that had a priority score above 0, fill in the strategy and venue (i.e., from Column 1 of the Decision Aid), record the scores from the Decision Aid Tool in the Scores columns, and fill in the relevant sector. As coalitions consider their selection of strategies, they should ensure that strategies collectively will target those at highest risk (i.e., people with history of prior overdose, reduced opioid tolerance, who engage in polydrug use, with major medical or mental illness, and/or who inject drugs). The HCS Research Team will help you complete the Implementation Feasibility column by providing insight on whether each strategy has a high, medium, or low feasibility of implementation based on resources, partnerships, and other factors. After reviewing content of the other columns, coalitions should come to consensus on the Overall Priority Ranking with the highest priority strategy ranked 1, the second highest priority ranked 2, and so on. Of note, the Overall Priority Ranking may or may not correspond directly to the rank order from the other scoring columns. After completing this tool, the next step will involve creating an initial implementation plan for the top 1-3 overall priority ranked strategies; at least one of the strategies must be from the required sub-menu (i.e., Safe Prescribing/Dispensing).

Menu 3: Strategies to Improve Prescription Opioid Safety							
	Scores					Implementation Feasibility? (High/Medium/Low)	Overall Priority Ranking
Strategy and Venue	G	F	I	PS	Sector?		
**a) Safer Opioid Prescribing/Dispensing Practices (Required)**							
Ex. Education materials around safe opioid disposal, targeting healthcare providers and patients.	3	3	2.25	8.25	HC	High	1
Ex. Education for community pharmacists focusing on red flags during opioid dispensing (dose, indication, duration, concurrent therapies, comorbid conditions, age, etc.).	1.25	2.5	1.5	5.25	HC	High	2
Ex. Educational outreach and academic detailing related to pain management for patients with OUD	2.5	2	2	6.5	HC	Medium	
Ex. Patient educational materials regarding appropriate chronic pain management made available in AA meetings and OUD recovery groups	2.5	2.25	1.75	6.5	BH	Medium	3
**b) Safer Opioid Disposal Practices (Optional)**							
Ex. Installation of permanent disposal kiosks	1.8	2.7	2	6.5	HC	High	4
Ex. Expansion and promotion of take-back events	2.3	2.3	2.25	6.85	Other	Medium	

Priority Score: G: Gap; F: Feasibility; I: Impact; PS: Overall Priority Score

Sector: CJ = Criminal Justice, HC = Healthcare, BH = Behavioral Health (See ORCCA Overview for details on assignment of sectors).

Coalition Implementation Planning Tool

Purpose: To more thoroughly define strategies and generate potential lists of venues/agencies to be involved in the implementation

Description: The tool also captured coalition's feedback on what they could do to help support implementation, strategies to ensure reach to under-represented populations, and indicators of success or failure of the strategy. Faculty with expertise in implementation science and the continuum of care added details on what the HCS faculty and staff would do to implement the strategy. Once complete, the tools were reviewed and approved in their entirety by the coalitions.

Who completes the tool: Coalitions and/or coalition workgroups with faculty and staff.

Coalition Implementation Planning Tool — Menu 3

For at least one but no more than three of the strategies with the top Overall Ranking from the Action Plan table, create an initial implementation plan using the table below (i.e., one table per strategy for up to three strategies). At least one of these strategies must come from the required sub-menu in the action plan (i.e., must address safe prescribing/dispensing practices). Provide information about specific activities in as much detail as is feasible at this time; insert additional rows as needed to describe these activities for the coalition and the HCS team.

<table>
<tr><td colspan="3">Menu 3 Overall Ranking = 1
Strategy: Educational outreach and academic detailing related to opioid prescribing for routine pain management. (Includes multiple sub-strategies as outlined on the Community Action Planning Tool).</td></tr>
<tr><td colspan="3">What specifically will be done? Ex: Educational outreach and academic detailing for all willing opioid prescribers (and possibly ambulatory pharmacists) in the County. Top priorities are: Primary Care, Hospital Emergency Department & local Urgent Care facilities, Dental Offices, Ortho Practices, Pain Management Clinics</td></tr>
<tr><td rowspan="3">What organization(s) should be involved? Provide points of contact if possible.</td><td>Agency: Ex. Family Practice</td><td>Contact: Ex. Dr. Smith</td></tr>
<tr><td>Agency: Ex. Pharmacy</td><td>Contact: Ex. Dr. Jones</td></tr>
<tr><td>Agency:</td><td>Contact:</td></tr>
<tr><td colspan="2">What will the coalition do?</td><td>Assigned to:</td></tr>
<tr><td colspan="2">1. Ex. Facilitate contact with Family Practice, Ambulatory Pharmacists</td><td>1. Ex. Coalition Chair</td></tr>
<tr><td colspan="2">2. Ex. Help with endorsement letter (with signatures from local hospital MDs</td><td>2. Ex. Safe Prescribing Champion</td></tr>
<tr><td colspan="2">What will HCS do?</td><td>Assigned to:</td></tr>
<tr><td colspan="2">1. Coordinate with listed coalition members to connect with identified organizations.</td><td>1. Ex. Prevention Team</td></tr>
<tr><td colspan="2">2. Draft a promotional letter/email to be reviewed and signed by community leaders who healthcare professionals respect and would positively respond to and recognize.</td><td>2. Ex. Prevention Team</td></tr>
<tr><td colspan="2">3. Develop community-specific implementation plan, target list, and educational materials using audiences and messaging the coalition identifies.</td><td>3. Ex. Prevention Team and Community Coordinator</td></tr>
<tr><td>How could we promote access/impact among diverse populations?</td><td colspan="2">Ex. Provide all patient-facing materials in Spanish, English, and any other languages deemed necessary; Prioritize County FQHC provider</td></tr>
<tr><td>How will we know it is working?</td><td colspan="2">Ex. Providers are willing to meet and listen/engage with continuing education materials; Providers agree to provide/request patient materials for distribution</td></tr>
<tr><td>What will make us change our strategy?</td><td colspan="2">Ex. Providers are not responsive or willing to engage; Educational materials are not reaching patients.</td></tr>
</table>

Appendix 2. Massachusetts Action Planning Tools

Community Profile Summary Form

Purpose: Discuss and Prioritize EBP Strategies that Align with Community Goals

Description: Developing ORCCA-specific Community Goals was a multi-step iterative process driven by community teams (CEFs, CDMs, Community Faculty and CCs). Small group conversations and 1:1s were scheduled with coalition members, local stakeholders, and champions. Community Faculty set the stage with stakeholders by sharing data that demonstrated the impact a combination of initiation on medications for opioid use disorder (MOUD), improvement on MOUD retention, and increasing the distribution of naloxone could have on the HCS communities. These data facilitated conversations about the potential impact of different EBPs. Teams outlined the importance of increasing access to naloxone, MOUD initiation, and MOUD retention within the context of each community. Together, community teams and stakeholders used community profile information to contextualize these data for their community. Community profiles, which included an overview of opioid-related risk, available treatment, and fatal overdose prevention services in the community, were co-created by study staff and community members. The profile, along with local data and coalition insight was used to identify areas of opportunity where ORCCA related strategies could fill unmet needs and/or enhance extant programming. This information was compiled on the community profile summary form.

Who completes the tool: This was an iterative process that involved community teams and subcommittees soliciting feedback from coalition members, dialoging, vetting ideas and ranking solutions as they grappled with how to maximize impact among high-risk populations.

Massachusetts HCS Wave 1 Community Profile Summary

Community Profile Summary **Coalition: _______________**

ORCCA Interventions	Existing Interventions (currently taking place)	Who are the service providers?	High-risk Populations (currently receiving services)	Existing programmatic collaborations (referrals and partnerships)	Areas of opportunity to improve existing services (to identify gaps)	Sustainability Include: Current funding sources and duration	Community Narratives
**Objective 1: Increase Opioid Overdose Prevention Education and Naloxone Distribution (OEND)**							
Required OEND							
Optional OEND							
Naloxone administration (optional)							
**Objective 2: Outreach and Delivery of Medications for Opioid Use Disorder (MOUD) to High-Risk Populations***							
Expand MOUD treatment (required)							
Linkage to MOUD (required)							
Engagement and retention (required)							
**Objective 3: Improve Prescription Opioid Safety**							
Safer Prescribing/ Dispensing (required)							
Safer Disposal Practices (optional)							

*For Definitions of "high-risk populations" see the Section II of the ORCCA Overview

Action Plan Summary Form

Purpose: To comprehensively outline strategies and determine their fiscal feasibility.

Description: Once the community profile summary was complete, community teams and coalitions began the process of selecting specific evidence-based practices. This involved participating in workshops and trainings to learn more about evidenced based strategies supported by the study, conducting 1:1s to engage potential implementation partners through coalition contacts, and assessing the extent to which high-risk populations and venues were engaged with the coalition and conducting outreach. Subgroups engaged in focused conversations exploring potential evidence-based practices using knowledge from previous conversations about evidence-based practices, as well as their feasibility, impact, and sustainability.

Community partners created budgets and estimates that were included later in proposals for action plan items. The action plan summary information was then discussed in subgroup meetings. During subgroup meetings coalition members and partner organizations further flushed out details of their plans refining them for presentation to the larger coalition. At the coalition meeting action plan summaries were discussed and voted on by the coalition membership using decision-making procedures such as consensus or majority voting as outlined in their respective coalition charters.

Who completes the tool: This was an iterative process that involved community teams, subcommittees, and partner organizations.

Massachusetts HCS Wave 1 Community Profile Summary & Action Plan Template

Massachusetts Action Plan Summary

Coalition: XXX

	Selected Strategies	ORCCA Goal (How it advances the overall HCS goal?	Targeted Populations	Primary High-Risk Sector BH	CJ	HC	Specific Activities Needed to Implement Selected Strategy: (who, what, when, where)	TA Needed	HCS Funds Needed
**ORCCA Objective 2: Outreach and Delivery of MOUD to High-Risk Populations**									
MOUD Linkage (required)	H. Linkage program via embedding an "In-reach worker" at XXX County Correctional Facility	Through the addition of this staff member, inmates leaving the facility will be provided with naloxone and a MOUD treatment appointment, with transportation provided, on release date.<br><br>Worker will be responsible for: creating relationships with inmates, running harm reduction groups, distributing Narcan, making community connections, scheduling same day appointments, scheduling transportation as needed.	People with reduced tolerance post-incarceration		X		X, Y, and Z orgs will work collaboratively to create a 30-40 hour/week position to fill gap between leaving incarceration and returning home in order to support recovery and lower risk of overdose after incarceration. Facility will determine number of residents released to determine number of hours for staff to spend in the facility.<br><br>Representatives from all 3 organizations will create job description and conduct interview to ensure right fit to work with incarcerated individuals. Qualifications will include knowledge of harm reduction principals, MOUD, OEND, and local services and treatment programs for referrals and connections.<br><br>Worker will ensure Narcan kits are given to inmates upon release (8:30am everyday). Worker will assist in activating inmates MA Health and secure appointments with outside service providers to bridge MOUD treatment post-release. Worker will be deputized as TPP concierge (see below) to have access to schedule rides for inmates being released directly to MOUD appointments in the community.	Group facilitator training<br><br>BAMSI and/or HCS to provide harm reduction training.<br><br>Praxis Narcan Train the Trainer<br><br>Facilitation training	Fulltime Salaried Position for 18 months: Payroll/Fringe $74,344.31<br><br>Mileage $855.00<br><br>Phone/Computer/Supplies/PPE $1,800<br><br>Total Direct Cost: $76,999.31<br><br>Admin Fee: $7,699.31<br><br>**TOTAL COST: $84,699.24**

Action Plan Review Form

Purpose: To assess action plan adherence to the ORCCA menu, impact, inclusion of high-risk populations/venues, feasibility and resources.

Description: Community-specific plans approved by the coalition memberships were then reviewed by HCS MA Leadership and State partners from the department of public health using a standard review form assessing: ORCCA adherence, Impact, Inclusion of High-Risk Populations/Venues, Feasibility and Resources. Written feedback was provided to community teams who then discussed feedback with their coalitions. Leadership met with community teams and coalitions as needed to support with plan clarification or revision. Revised plans were resubmitted for approval by HCS MA Core leaders and state partners.

Who completes the tool: HCS MA Leadership and State partners from the department of public health.

For peer review only

Community:

Please comment below about the extent to which the plan meets the designated criteria, including strengths, challenges and modifications.

Approval Criteria	OEND Plan	MOUD Plan	Safer Prescribing Plan
**ORCCA Adherence**<br>☐ Within the scope of the ORCCA menu<br>☐ Aligned with the overall goal of HCS			
**Impact**<br>☐ Extent to which plan will:<br>o Increase # POUD on MOUD<br>o Retain POUD in care<br>o Increase OEND to POUD at highest risk<br>o Address gaps in the continuum of care<br>o Be sustainable			
**High-Risk Population/Venues**<br>☐ Extent to which plan will:<br>o Include efforts to engage with people who have been historically marginalized<br>o Prioritize populations at heightened risk for overdose - BH/CJ/HC (i.e. prior overdose, decreased tolerance, multi-substance use, concomitant medical or mental illness, homeless, non-English speakers, minority populations)<br>o Mitigate and not exacerbate underlying racial inequities<br>o Prevent unintended consequences			
**Feasibility**<br>☐ Extent to which plan is:<br>o Feasible to implement by January 1, 2021<br>o Clear on what will be done by who and by when to bring about change<br>☐ Extent to which structural or regulatory challenges act as a barrier for this plan			
**Resources**<br>☐ Plan is within budget			

For peer review only

Appendix 3. New York Action Planning Tools

Community Evidence Based Practice Goals

Purpose: To define community level goals for EBPs

Description: The starting point for action planning is reflection and discussion by the coalition on the Community Profile, Data Dashboard, and locally sourced data summarized in a Discussion Tool from Phase 3. This is followed by development of community goals for each ORCCA EBP. Coalitions were asked to reflect on the success of COVID-19 influenced fast tracked strategies from earlier phases and to consider expansion and sustainability of these strategies, as well as to use a health and racial equity lens in the development of community Specific, Measurable, Attainable, Realistic and Time-framed (SMART) goals for each EBP.

Who completes the tool: The community-based implementation team captures discussions and decisions from Coalition members starting in workgroups with their champions by ORCCA Menu and further facilitated in the larger coalition.

DIRECTIONS: Setting ORCCA Goals
The Implementation Team facilitates a discussion with the Coalition (Steering Committee) to identify ORCCA goals based on the recommendations of the ORCCA, Communication and Data Workgroups.

These will be community-level goals to provide OEND in high risk populations, provide outreach and delivery of MOUD to high populations, and provide for safer opioid prescribing and dispensing.

ORCCA Goals

Providing OEND in High Risk Populations	Ex. In 12 months expand OEND through four strategies: expanding peer navigation, expand virtual training, using a variety of distribution strategies, and expanding the number, integration and reach of community trainers by 50%.
Outreach and Delivery of MOUD to High-Risk Populations	Ex. In 9 months, expand access to MOUD by increasing the number of waivered MOUD prescribers and treatment slots; improving prescribing rates of current waivered prescribers; create and sustain a prescriber advisory board to support best practice; and enhancing care coordination, outreach and linkage to enhance engagement and retention in care. Use a data feedback system to improve coordination across providers and service systems and address SDH through a targeted response to increase MOUD retention.
Safer Opioid Prescribing and Dispensing	Ex. Improve safer opioid prescribing and dispensing practices by engaging prescribers and pharmacies to provide resources on safer prescribing, tapering practices, alternative pain management and promoting safer opioid disposal practices between 6-12 months from the time of implementation.

Action Planning Worksheet

Purpose: To identify EBP strategies, venues and priority populations in an iterative process using community profiles, and data identifying needs, gaps and opportunities

Description: Workgroups brainstormed possible ORCCA EBP strategies and communication messages following training and practice using the ORCCA and technical assistance guide (TAG) that could fill existing gaps with identified populations, sectors and venues discussed in Phase 3. ORCCA EBP strategies were considered that could strengthen ongoing community efforts, create opportunities for expansion and efficiency, and promote synergistic approaches to new and existing community strategies. These strategies were presented to the coalition for consideration.

Who completes the tool: Coalition members, workgroups led by champions from each menu and facilitated by community implementation teams

Table 1: Discussion Tool for STEP 1 (CTH PHASE 4)

Strategy	Description & Approach to Population Access (High Risk/Venue)<br>Briefly describe each specific strategy being considered.<br>For strategy, identify populations of focus and venues for reaching them.		STRATEGY<br>Label each strategy:<br>Scale up<br>New Population<br>New Feature<br>New Service<br>New Service & Population	Pros and Cons of each Strategy
	**High Risk Populations**<br>Prior Opioid Overdose<br>Reduced Opioid Tolerance<br>Other Substance Use<br>Co-occurring mental health<br>Co-occurring physical health<br>Injection Drugs	**Venues**<br>Harm Reduction/Syringe Service<br>Health Care<br>Criminal Justice<br>First Responder<br>Addiction Treatment & Recovery<br>Behavioral Health Treatment<br>Community Based -Social Service Hotline		
**OEND in High Risk Populations**				
Active OEND (required)	*EX: Distribute naloxone to released inmates at County Jail or other setting*		EX: scale up or new service	
Passive OEND (optional)	*EX: Distribute naloxone at pharmacy or dispensing program*		EX: New feature	
Naloxone administration strategies (optional)	*EX: First responder naloxone administration*		EX: scale up	
**Outreach and Delivery of MOUD to High-Risk Populations**				
Bridging MOUD (required)	*EX: bridge program for people returning to community from county jail*		EX: new service	
Linkage using Peer Navigator (required)	*EX: Linkage to peer navigation for patients who overdose in ED*		EX: new feature	
Engage and retain individuals in treatment (required)	*EX: telemedicine, virtual appointment*		EX: new feature	
**Safer Opioid Prescribing and Dispensing**				
Safer opioid prescribing/dispensing practices (required)	*EX: Emergency/urgent care*		EX: scaling up	
Safer opioid disposal practices (optional)	*EX: • Prescription drug drop-box/mail-back programs*		EX: scale up or new service	

An additional tool used to make decisions about strategies and their potential for synergy and impact, was agent-based and systems dynamic modeling. Feedback reports using this modeling were introduced to County Implementation Teams and workgroups alongside community profiles to highlight the importance of OEND and MOUD expansion specifically, in order to meet the intervention's opioid overdose prevention goals (see Figure 3), and to inform the prioritization, adaptation, maintenance and sustainability of effective interventions over time for action planning. The systems perspective is intended to be integrated into problem-solving and feedback during ongoing implementation to improve coordination and resource sharing within community networks.

For peer review only - http://bmjopen.bmj.com/site/about/guidelines.xhtml

Figure 3-NY. Systems Feedback Reports for Planning

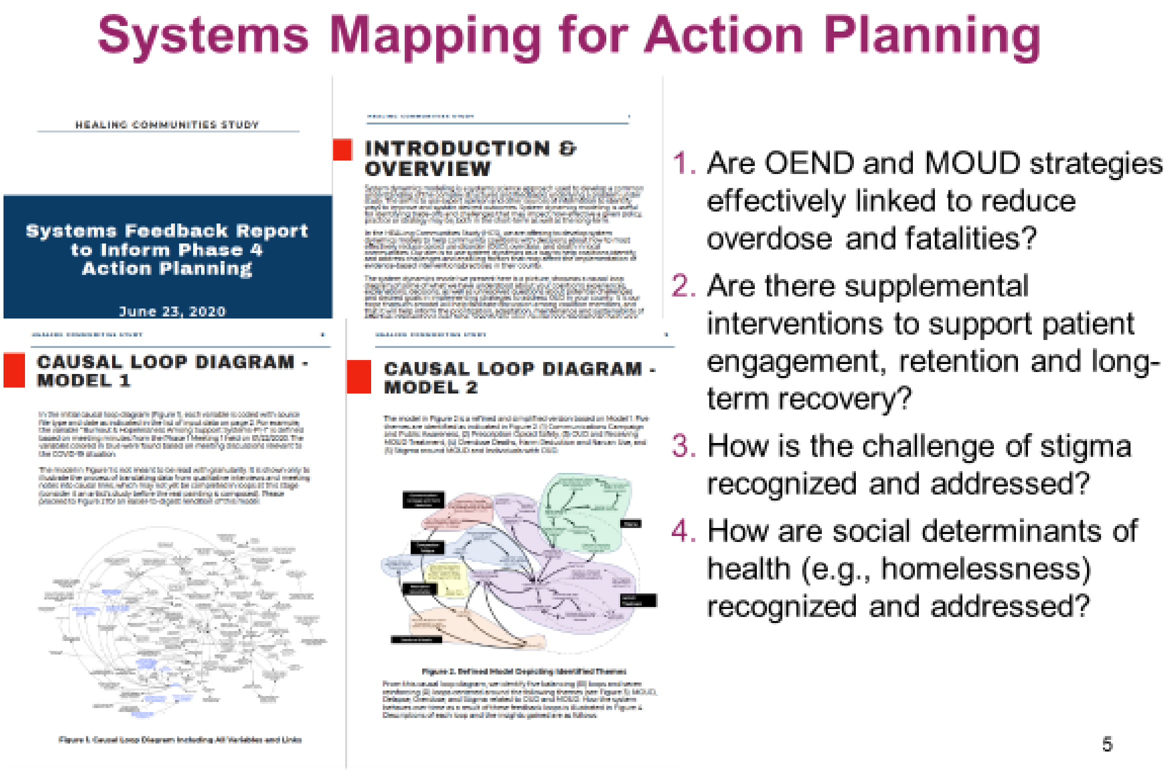

ew only

Consultation and Coaching Plan

Purpose: To provide coaching and feedback to assure adherence to HCS required EBP selections, and enhance capacity of local teams for community planning

Description: The research study team, including implementation coaches assigned to each community, offered iterative support as Community Implementation Teams and workgroups ranked strategy options based on earlier discussion of impact and feasibility determinations in workgroups, finalized strategy selections, and refined strategies into specific SMART goals. The ranking and strategy selection process was not uniform across coalitions. While no limits were placed on the number of strategies selected, attention was given to ensure the 5 required strategies defined in the ORCCA were present or a justification for why not were included. Opportunities were highlighted during coaching sessions to reinforce data driven justification for strategy selection and to feature the role of EBP workgroup champions in leading planning.

Who completes the tool: The Study Team reviews the Action Planning Worksheet with Summary Action Plans with the Implementation Team in coaching sessions using the criteria below:

ORCCA Goals	Prioritized EBPs	Draft CAP	Adopted CAP
Goals are aligned with HCS goal to reduce opioid overdose mortality by 40%	Priorities are aligned with the ORCCA-specific goals	CAP summarizes coalition goals	Summarizes coalition goals
Goals target identified gaps in the provision of OEND, MOUD, and safer opioid prescribing and dispensing	Priorities address the gaps documented in Community Profile	CAP summarizes selected strategies	Summarizes selected strategies, venues and populations
	Workgroup prioritized strategies as high impact	CAP identifies the team responsible for implementing the strategies	Identifies the team responsible for implementation planning and organizations for initial engagement
	Workgroup prioritized strategies as high feasibility	CAP identifies and the timeframe for implementation planning	Identifies the timeframe for implementation planning
		To what degree have considered health equity and racial disparities?	Have considered health equity and racial disparities
		Initial TA and resources needed, including plans to use Community Impact (CI) Funds	TA and resources needed, including plans to use CI Funds

For peer review only

SCHEDULING REVIEW AND CONSULTATION

Implementation Teams should upload and maintain documentation for each deliverable (ORCCA Goals, prioritized EBPs and the Community Action Plan) on the Public HCS County Folders drive. Project Managers should notify the Study Team by email to request review and coaching sessions. Please use the following timetable:

Deliverable	When to Request Review	Review Type	Target Deadline	Response Time
**ORCCA Goals**	Workgroup and Coalition consensus on ORCCA goals	Review and comment	**00/00/0000**	2 Business Days
**Prioritized EBPs Draft Community Action Plan**	Prioritized EPBs and draft Community Action Plan is ready for recommendation the Coalition	Coaching Meeting	At least 1 week prior to Coalition Meeting at which EBPs and AP will be considered	2 Business Days
**MOUD and Safer Rx EBP, AP**	Consulting addiction psychiatrists will also provide consultation and review of the prioritized EBPs and draft Community Action Plan for MOUD and Safer Prescribing Workgroups during Workgroup meetings.			
**Approved Community Action Plan**	Coalition has approved the Community Action Plan	Review and comment	Next business day after Coalition approval	2 Business Days

The Implementation Teams can invite the Study Team to join the Workgroup meeting in which EBP strategies are being prioritized to provide review and consultation, if that is the preference of the Workgroup, Champion and Implementation Team.

For peer review only

Summary Community Action Plan Form

Purpose: To provide a summary of EBPs and community goals, venues and priority populations with initial organizations, and first steps to be the focus for implementation planning in Phase 5.

Description: The final step in the Phase 4 Action Planning Worksheet is a summary community action plan for final coalition approval guided by the coalition's charter on decision-making. Most used a majority vote in a coalition meeting. The plan includes defined SMART goals with an outcome target for each ORCCA EBP-related strategy and identification of specific populations, sector for implementation (i.e., behavioral health, criminal justice, healthcare), reference to feasibility and impact rankings from workgroup discussions, and preliminary sustainability. Additionally, in anticipation of Phase 5 Implementation Plan Agreement development, the action plan includes recommended initial and anticipated steps for engaging community partners and estimates of technical assistance and resources.

Who completes the tool: Community implementation teams led by a program manager and CEF complete the summary and present to the coalition for adoption. In some cases a county executive or leadership team provides an additional level of approval.

TABLE 6: Action Plan Table (CTH PHASE 4)

Strategy Category	Selected Strategies	Targeted Populations	High-Risk Sector			Sustainability Feasibility/ Impact	Implementation Steps: who does what, by when and where	Technical Assistance & Other Resources
			BH	CJ	HC			
**ORCCA Objective 1: Increase Opioid Overdose Prevention Education and Naloxone Distribution**								
Active OEND (required)	A1:<br>A2:							
Passive OEND (optional)	B1:<br>B2:							
Naloxone administration (optional)	C1:<br>C2:							
**ORCCA Objective 2: Outreach and Delivery of MOUD to High-Risk Populations**								
Expand MOUD treatment (required)	D1:<br>D2:							
Linkage to MOUD (required)	E1:<br>E2:							
Engagement and retention (required)	F1:<br>F2:							
**ORCCA Objective 3: Improve Prescription Opioid Safety**								
Safer Prescribing/ Dispensing (required)	G1:<br>G2:							
Safer Disposal Practices (optional)	H1:<br>H2:							

For peer review only - http://bmjopen.bmj.com/site/about/guidelines.xhtml

Appendix 4. Ohio Action Planning Tools

Decision Aid 1 Matrix

Purpose: To identify existing venues related to ORCCA EBPs

Description: In Ohio, the HCS Intervention Facilitator, one field team (i.e., an HCS-hired Intervention Facilitator, Community Engagement Facilitator, Data Manager) worked with community coalitions, particularly coalition-identified EBP champions to utilize a multi-step Decision Aid Approach across Phase 4 study activities. The first step was to identify existing venues related to ORCCA EBPs. In Step 1: Cataloging Possible Approaches to Population Access, a matrix was used in Decision Aid 1 to map existing locations/methods of current *or potential* population access to priority populations. This allowed communities to inventory potential points of access and also highlighted options that were not available or are already highly represented.

Who completes the tool: Decision Aid 1 is completed by ORCCA EBP coalition champions in collaboration with HCS-hired staff.

**Community "A": Decision Aid 1**		**Points of Population Access**		
		**CJ**	**SSPs**	**EDs**
**Priority Populations**	**Prior Opioid Overdose**	ABC drug court<br>XYZ County Jail	Abc street exchange	None
	**Reduced Opioid Tolerance**	ABC drug court<br>XYZ County Jail		None
	**Use Other Substances**	ABC drug court<br>XYZ County Jail	Abc street exchange	None

For peer review only

Decision Aid 2

Purpose: To identify preliminary ORCCA-related goals for each of the three EBPs.

Description: In Step 2: Existing Service Inventory Mapped by Priority Population and Point of Population Access, a similar approach to Decision Aid 1 was employed, but limited to listing *currently available* services (i.e., interventions) specific to each of the three EBPs required by ORCCA. As a first step, a community would use their population access Decision Aid 1 from Step 1 to highlight any setting in which there was a current intervention related to the three ORCCA required objectives. Next, each community would catalog any existing services that address the three ORCCA required objectives using Decision Aids 2A (OEND), 2B (MOUD), and 2C (Safer Prescribing). This approach documents the gaps between potential methods for population access and the degree to which those were already being implemented.

Who completes the tool: Decision Aid 2 is completed by ORCCA EBP coalition champions in collaboration with HCS-hired staff.

Community "A"- Decision Aid 2B: MOUD		Points of Population Access		
		**CJ**	**SSPs**	**EDs**
**Priority Populations**	**Prior Opioid Overdose**	None	Abc Syringe Exchange-treatment referral	None
	**Reduced Opioid Tolerance**	None	None	None
	**Use Other Substances**	None	Abc Syringe Exchange-treatment referral	None

For peer review only

Decision Aid 3

Purpose: To identify, prioritize, and select EBP strategies.

Description: In Step 3 of the Decision Aid process, communities in collaboration with HCS *brainstormed potential intervention strategy and population access dyads.* This approach allowed for a shared understanding of "what was currently being done" and "where it was being done" in an efficient manner that mapped structurally to ORCCA priority populations and methods of population access. The Decision Aid was not menu-specific, but rather covered strategies for intervention across menus. This provided the foundation for brainstorming which ORCCA EBP strategies *might target existing gaps.* In this step, there is an emphasis on feasibility of implementation.

Discussion Aid 3 was employed in Step 3 and prompted communities to consider whether the identified options involved:

a. bringing an existing service to scale ("1")
b. disseminate an existing service to a new population or method of population access ("2")
c. add a new feature or improvement to an existing service (i.e., changing passive referral to formal linkage intervention); ("3")
d. add a new service; ("4")
e. add both a new method of population access and a new service (e.g., a syringe service program with naloxone distribution); ("5").

Who completes the tool: Decision Aid 3 is completed by ORCCA EBP coalition champions in collaboration with HCS-hired staff.

**Community A-Decision Aid 3: Possible Strategies for Intervention—*no priority order***		**Approach to Population Access**	**Category of Implementation (1-5 above)**
**1**	Distribute OEND	XYZ County Jail	1: bring to scale
**2**	Bridging MOUD	XYZ County Jail	4: new program
**3**	Linkage to Care-Mandated Treatment	ABC Drug Court	1: bring to scale
**4**	Linkage using Peer Navigator	XYZ County Jail	4: new program
**5**	Linkage using Peer Navigator	SSP	3/4: modifying referral program/new program

For peer review only

Decision Aid 4

Purpose: To prioritize EBP strategies by feasibility and expected impact.

Description: This step involved consideration of the intersection between the importance of a given plan and the degree of difficulty in implementing that plan with the notion that EBPs that were both *highly impactful* and *highly feasible* should be undertaken before other actions that are of lower impact and/or lower feasibility. In Decision Aid 4, the identified strategies were categorized by priority/importance (low, medium, high) and by feasibility/capacity (low, medium, high)

Who completes the tool: Decision Aid 4 is completed by ORCCA EBP coalition champions in collaboration with HCS-hired staff.

**Community A-Decision Aid 4: Possible Strategies for Intervention—*feasibility/priority***		**Feasibility/Capacity**		
		Low	Medium	High
**Priority/Importance**	Low	• Linkage to Care Mandated Treatment at ABC Drug Court (*i.e., already at scale with little opportunity to expand*)	• Linkage using peer-navigators at XYZ county Jail (i.e. may move to high priority if bridging MOUD is unsuccessful)	
	Medium		• Distribute OEND at XYZ county jail	
	High	• Bridging MOUD at XYZ county jail	•	• Linkage using peer-navigators at SSP

For peer review only

Decision Aid 5

Purpose: To prioritize and select EBP strategies.

Description: In Step 5: Prioritizing and Selecting EBPs, communities ranked the options outlined in Step 3 in priority order based on the feasibility and impact determinations of Step 4. Coalitions were encouraged to prioritize options that were high impact and high feasibility over interventions that were of low impact or feasibility. Of note, the coalition was not limited in the number of strategies they could target for implementation. This determination was encouraged to be paired with the decision on whether the community and HCS team would pursue a given option for implementation.

Utilizing Step 5 of the Decision Aid process, communities then create their initial action plan specific to their ORCCA goals and the required ORCCA EBPs. The action plan includes the specific strategies selected, the targeted sectors for the intervention, the identified practice settings or venues, preliminary action plans, potential technical assistance or other resource needs, the individual within the Ohio Intervention Design Team (IDT), comprised of academic and subject experts, who will be primarily overseeing and assisting with the implementation of the identified strategy, and additional notes regarding the strategy. For example, if an EBP were to be conducted in an Emergency Department setting, the Emergency Department IDT would oversee the implementation of that EBP and provision of technical assistance.

Who completes the tool: Decision Aid 5 and the Action Plan is completed by ORCCA EBP coalition champions and community coalitions in collaboration with HCS-hired staff.

**Community A-Decision Aid 5: Selected Strategies for Intervention—*priority order***		**Approach to Population Access**	**Target for Action**	**Notes/Preliminary Action Plan**
**1**	Linkage using Peer Navigator	SSP	Yes	Hire program coordinator; select peers
**2**	Bridging MOUD	XYZ County Jail	Yes	
**3**	Distribute OEND	XYZ County Jail	Yes	
**3**	Linkage to Care-Mandated Treatment	ABC Drug Court	No	
**4**	Linkage using Peer Navigator	XYZ County Jail	No	Revisit in 3 months pending exploration of bridging MOUD at XYZ County Jail

For peer review only

Action Plan Template and Sample Completed Action Plan

County: _______________________

Completed By: ___ ______
Initial Completion Date: _____/____/______
Revision #: _____________
Revision Completion Date: _____/________/________

	Specific Strategy	Approach to Population Access (Sector)	Practice Setting or Venue Name	Preliminary Action Plan	Potential TA/Resource Needs	State IDT Lead	Notes
**ORCCA Objective 1: Increase Opioid Overdose Prevention Education and Naloxone Distribution**							
Active OEND (Required)							
Passive OEND (optional)							
**ORCCA Objective 2: Outreach and Delivery of MOUD to High-Risk Populations**							
Expand MOUD treatment (required)							
Linkage to MOUD (required)							
Engagement and retention (required)							
**ORCCA Objective 3: Improve Prescription Opioid Safety**							
Safer Prescribing/ Dispensing (required)							
Safer Disposal Practices (optional)							

review only

Strategy category	Specific Strategy	Approach to Population Access (Sector)	Practice Setting or Venue Name	Preliminary Action Plan	Potential TA/Resource Needs	State IDT Lead	Notes
**ORCCA Objective 1: Increase Opioid Overdose Prevention Education and Naloxone Distribution**							
**Active OEND (required)**	**Active OEND for at-risk individuals and their social networks**<br><br>*Expansion of Project DAWN (a program to provide naloxone to Ohio agencies) to a secondary site*	Behavioral Health	Church	Confirm partner agencies. (HCS)<br><br>Assemble Intervention Design Team/Operations Team to provide implementation assistance. (HCS)	Resources needed on technology/ data collection, billing for sustainability.	[NAME]	Date approved by coalition: DATE
**Active OEND (required)**	**Active OEND for at-risk individuals and their social networks**<br><br>*Expansion of Project DAWN to a secondary site*	Healthcare	[AGENCY] Mobile Unit	Confirm partner agencies. (HCS)<br><br>Assemble Intervention Design Team/Operations Team to provide implementation assistance. (HCS)	Resources needed on technology/ data collection, billing for sustainability.	[NAME]	Date approved by coalition: DATE
**Active OEND (required)**	**Active OEND at high-risk venues:**<br><br>*Naloxone Referral/ Distribution for Drug Court Participants*	Criminal Justice	Drug Court	Confirm partner agencies. (HCS)<br><br>Assemble Intervention Design Team/Operations Team to provide implementation assistance. (HCS)	Potential resources needed on increasing access and participant engagement.	[NAME]	Date approved by coalition: DATE
**Active OEND (required)**	**Active OEND at high-risk venues: "Leave behind"**	Healthcare	Fire/EMS	Confirm partner agencies. (HCS)<br><br>Assemble Intervention Design Team/Operations	Potential resources needed on policy/ implementation logistics, streamlined data collection for naloxone use.	[NAME]	Date approved by

For peer review only

Strategy category	Specific Strategy	Approach to Population Access (Sector)	Practice Setting or Venue Name	Preliminary Action Plan	Potential TA/Resource Needs	State IDT Lead	Notes
	**programs at sites of overdose**<br><br>*Fire/EMS Leave-Behind Program*			Team to provide implementation assistance. (HCS)			coalition: DATE
**OEND self-request (e.g. at pharmacy, community meeting or public health department)**	**Passive OEND**<br><br>Naloxone Distribution at Pharmacies in high risk areas	Healthcare	Pharmacy	Confirm partner agencies. (HCS)<br><br>Assemble Intervention Design Team/Operations Team to provide implementation assistance. (HCS)	Potential resources needed on logistics and possible policy development.	[NAME]	Date approved by coalition: DATE
**ORCCA Objective 2: Outreach and Delivery of MOUD to High-Risk Populations**							
**Expand MOUD treatment (required)**	**Adding/expanding MOUD treatment in Criminal Justice settings**<br><br>*MOUD in the Jail*	Criminal Justice	Jail	Confirm partner agencies. (HCS)<br><br>Assemble Intervention Design Team/Operations Team to provide implementation assistance. (HCS)	Potential resources needed on logistics, billing, etc.	[NAME]	Date approved by coalition: DATE
**Linkage to MOUD**<br><br>**(required)**	**Linkage Programs (all relevant settings)**<br><br>*Expand MOUD referral in ED*	Healthcare	Emergency Department	Confirm partner agencies. (HCS)<br><br>Identify opportunities for expansion of current services.<br><br>Assemble Intervention Design Team/Operations Team to provide implementation assistance. (HCS)	Potential resources needed on sustainability and patient engagement.	[NAME]	Date approved by coalition: DATE

For peer review only

Strategy category	Specific Strategy	Approach to Population Access (Sector)	Practice Setting or Venue Name	Preliminary Action Plan	Potential TA/Resource Needs	State IDT Lead	Notes
**Engagement and retention (required)**	**Reducing barriers to housing, transportation, childcare and accessing other community benefits for people with OUD**<br><br>*Reduce barriers to transportation to improve engagement/retention in treatment*	Behavioral Health	MOUD Treatment Facility<br><br>Potentially other MOUD treatment facilities with limited transportation options. (TBD)	Confirm partner agencies. (HCS)<br><br>Assemble Intervention Design Team/Operations Team to provide implementation assistance. (HCS)<br><br>Engage partners and IDT through an initial planning meeting. (HCS)	Potential resources needed on billing for sustainability.<br><br>Needs: Identify potential partnerships between XXX transportation and treatment agencies	[NAME]	Date approved by coalition: DATE
**ORCCA Objective 3: Improve Prescription Opioid Safety**							
**Safer Prescribing/ Dispensing (required)**	**Safer opioid prescribing for chronic pain**<br><br>Prescriber Accountability through OD Review	Healthcare	Pharmacy	Confirm partner agencies. (HCS)<br><br>Assemble Intervention Design Team/Operations Team to provide implementation assistance. (HCS)<br><br>Engage partners and IDT through an initial planning meeting. (HCS)	Potential resources needed on logistics and identifying allowable information to be shared between the XXX team and XXX Agency.	[NAME]	Date approved by coalition: DATE
**Safer Prescribing/ Dispensing (required)**	**Safer opioid dispensing**<br><br>Utilize the Take Charge Ohio Toolkit for pharmacist and patient education. (Co-dispensing naloxone, providing educational materials	Healthcare	Pharmacy	Engage Education and Awareness workgroup of Scioto County Collaborative Opioid Consortium.<br><br>Determine which parts of the Toolkit will be utilized.<br><br>Confirm partner agencies. (HCS)	Potential resources needed on logistics and possible policy development.	[NAME]	Date approved by coalition: DATE

For peer review only

Strategy category	Specific Strategy	Approach to Population Access (Sector)	Practice Setting or Venue Name	Preliminary Action Plan	Potential TA/Resource Needs	State IDT Lead	Notes
	on opioids and naloxone)			Assemble Intervention Design Team/Operations Team to provide implementation assistance. (HCS)			

For peer review only

BMJ Open

Protocol for community-driven selection of strategies to implement evidence-based practices to reduce opioid overdoses in the HEALing Communities Study, a trial to evaluate a community-engaged intervention in Kentucky, Massachusetts, New York, and Ohio

Journal:	*BMJ Open*
Manuscript ID	bmjopen-2021-059328.R3
Article Type:	Protocol
Date Submitted by the Author:	22-Aug-2022
Complete List of Authors:	Young, April; University of Kentucky, Epidemiology<br>Brown, Jennifer; University of Cincinnati<br>Hunt, Timothy; Columbia University<br>Sprague Martinez, Linda; Boston University<br>Chandler, Redonna; National Institutes of Health<br>Oga, Emmanuel; Research Triangle Institute<br>Winhusen, Theresa ; University of Cincinnati Medical Center, Psychiatry<br>Baker, Trevor; Boston Medical Center<br>Battaglia, Tracy; Boston Medical Center<br>Bowers-Sword, Rachel; Boston Medical Center<br>Button, Amy; Albert Einstein College of Medicine, Montefiore Hudson Valley Collaborative<br>Fallin-Bennett, Amanda ; University of Kentucky, College of Nursing<br>Fanucchi, Laura; University of Kentucky<br>Freeman, Patricia ; University of Kentucky College of Pharmacy<br>Glasgow, LaShawn; Research Triangle Institute<br>Gulley, Jennifer; Clark County Health Department<br>Kendell, Charles; Franklin County Agency for Substance Abuse Policy Board<br>Lofwall , Michelle ; University of Kentucky, Center on Drug and Alcohol Research<br>Lyons, Michael S.; Cincinnati Children's Hospital Medical Center<br>Quinn, Maria; Holyoke Medical Center<br>Rapkin, Bruce; Albert Einstein College of Medicine, Epiemiology and Population Health<br>Surratt, Hilary; University of Kentucky<br>Walsh, Sharon ; University of Kentucky
**Primary Subject Heading**:	Addiction
Secondary Subject Heading:	Addiction, Evidence based practice, Public health, Research methods
Keywords:	Substance misuse < PSYCHIATRY, PUBLIC HEALTH, STATISTICS & RESEARCH METHODS

For peer review only - http://bmjopen.bmj.com/site/about/guidelines.xhtml

SCHOLARONE™
Manuscripts

Protocol for community-driven selection of strategies to implement evidence-based practices to reduce opioid overdoses in the HEALing Communities Study, a trial to evaluate a community-engaged intervention in Kentucky, Massachusetts, New York, and Ohio

Young AM, Brown JL, Hunt T, Sprague Martinez LS, Chandler R, Oga E, Winhusen TJ, Baker T, Battaglia TA, Bowers-Sword R, Button A, Fallin-Bennett A, Fanucchi L, Freeman PR, Glasgow LM, Gulley J, Kendell C, Lofwall MR, Lyons MS, Quinn M, Rapkin B, Surratt H, Walsh S.L.

Corresponding Lead Author: April M Young (april.young@uky.edu)

For peer review only

Abstract

Introduction: Opioid-involved overdose deaths continue to surge in many communities, despite numerous evidence-based practices (EBPs) that exist to prevent them. The HEALing Communities Study (HCS) was launched to develop and test an intervention (i.e., Communities That HEAL [CTH]) that supports communities in expanding uptake of EBPs to reduce opioid-involved overdose deaths. This paper describes a protocol for a process foundational to the CTH intervention through which community coalitions select strategies to implement EBPs locally.

Methods and analysis: The CTH is being implemented in 67 communities (randomized to receive the intervention) in four states in partnership with coalitions (one per community). Coalitions must select at least five strategies, including one to implement each of the following EBPs: (a) overdose education and naloxone distribution; expanded (b) access to medications for opioid use disorder [MOUD], (c) linkage to MOUD, (d) retention in MOUD; and (e) safer opioid prescribing/dispensing. Facilitated by decision aid tools, the community action planning process includes (1) data-driven goal setting, (2) discussion and prioritization of EBP strategies, (3) selection of EBP strategies, and (4) identification of next steps. Following review of epidemiologic data and information on existing local services, coalitions set goals, and discuss, score, and/or rank EBP strategies based on feasibility, appropriateness within the community context, and potential impact on reducing opioid-involved overdose deaths with a focus on three key sectors (health care, behavioral health and criminal justice) and high-risk/vulnerable populations. Coalitions then select EBP strategies through consensus or majority vote and, subsequently, suggest or choose agencies with which to partner for implementation.

Ethics and dissemination. The HCS protocol was approved by a central IRB (Advarra, Inc) and is registered with Clinical Trials.gov (NCT04111939). Results of the action planning process will be disseminated in academic conferences and peer-reviewed journals, online and print media, and in meetings with community stakeholders.

For peer review only

Strengths and limitations of this study

- A strength of the CTH action planning protocol is that it balances scientific rigor with community priorities by ensuring all communities follow a common core of structured activities while also allowing variation across states and communities to best suit the local context.
- The methods used in the CTH action planning process elevate the voices of community members, whose insights are critical to implementation success and whose involvement conveys to local stakeholders that EBP implementation is a priority of the local community.
- The action planning protocol requires careful facilitation by coalition members and community staff to ensure that conflicts of interest are managed and power dynamics do not result in some coalition members having more influence than others in the decision-making process.
- The phased and iterative action planning protocol is time-intensive requiring significant commitment from involved community members, faculty, and staff, and may slow implementation of EBPs.
- While necessitated by the constraints of the study timeline, the research teams' intensive involvement in facilitating the action planning protocol could be a limitation as it may stifle participation by coalition members who approach the process with deference and thereby limit the extent to which it is community-driven.

For peer review only

Introduction

Opioid-involved overdose deaths remain a public health crisis in many communities worldwide[1 2] although numerous evidence-based practices (EBPs) exist to prevent them. In the Unites States alone, opioid-involved overdose claimed over 490,000 lives between 1999 and 2019.[3] Despite national efforts to change the trajectory of overdose deaths, provisional data indicate over 94,000 total overdose deaths occurred in the 12 months ending in January 2021, making this the highest number of overdose deaths ever recorded in a 12-month period.[4]

Research has demonstrated that medication for opioid use disorder (MOUD), including methadone, buprenorphine, and extended-release naltrexone;[(e.g. 5)] prescription drug monitoring programs and safer prescribing guidelines for prescription opioids;[(e.g. 6)] naloxone, a medication that can reverse an opioid overdose;[(e.g.,7)] and behavioral/psychosocial interventions like recovery support services [(e.g. 5)] can prevent deaths and help people enter and stay in remission and recovery from opioid use disorder. Unfortunately, these EBPs remain under-utilized in many communities leaving significant unmet need for these critically important services.[8-15]

The HEALing Communities Study (HCS), supported by the National Institutes of Health and the Substance Abuse and Mental Health Services Administration, launched in 2019 to develop and test an approach to support communities in expanding uptake and availability of EBPs to address opioid-involved overdose deaths and opioid use disorder (OUD). HCS is a multi-site, parallel-group, cluster randomized wait-list controlled trial involving 67 communities (34 in active intervention and 33 in wait-list control) in Kentucky (KY), Massachusetts (MA), New York (NY), and Ohio (OH).[16] The study is designed to test the Communities That HEAL (CTH) intervention, which contains three components: a community-engaged process of data-driven decision making to select EBP strategies for local implementation and monitoring;[17] a

For peer review only

menu of EBPs and technical assistance guides (Opioid-Overdose Reduction Continuum of Care Approach [ORCCA]),[18] and a set of communication campaigns intended to address stigma and increase knowledge of and demand for EBPs.[19] The CTH intervention is being implemented in the active intervention communities from January 2020 to June 2022 and will be implemented in wait-list control communities from July 2022 to December 2023.

The CTH intervention is grounded in the recognition that communities affected by the opioid overdose crisis possess the best understanding of local strengths, gaps, barriers and facilitators related to local EBP implementation and uptake.[17] Thus, the CTH involves partnerships between academic researchers and community members through community coalitions. Coalitions are comprised of representatives from diverse agencies and institutions as well as individuals focused on tackling complex community issues through collective planning.[20] The CTH intervention employs a phased coalition planning process that was developed based on and expands the Communities that Care model,[17] which has supported communities in the selection of evidence-based prevention efforts in past research.[21-23] Coalition planning models are effective because they allow communities to leverage resources and expertise from across multiple sectors, which can facilitate creative problem solving and innovation, reduce duplication of efforts, and address service gaps.[24 25] The literature points to coalition planning as an effective strategy for implementing EBPs.[26-28]

The purpose of this paper is to describe the protocol for coalition action planning through which EBP strategies are selected to reduce opioid overdose deaths in their communities.[16] The protocol described in this manuscript is relevant beyond the field of substance use, as community engagement and community-driven decision making are recommended for improving responses

For peer review only

to a variety of health challenges,[29-32] including, most recently, SARS-CoV-2 vaccination[33] and Monkeypox.[34]

Methods and Analysis

Participant involvement

As described elsewhere,[17] community engagement is central to the CTH intervention. The CTH intervention places communities, specifically community coalitions, at the center of the decision-making process in creating an action plan for implementing EBP strategies locally. The coalitions are comprised of key stakeholders in healthcare, behavioral health, criminal justice, public health, government, business, housing, and other sectors, and in many cases, include people with lived experience with or impacted by OUD. In some communities, the coalitions that are presently engaged in the CTH existed prior to the HCS, while others were formed specifically to guide the CTH intervention. In addition to driving the CTH intervention, these coalitions facilitate dissemination of information about the study and about EBPs to other local stakeholders. Coalitions, local agency partners, and partnerships formed between academic institutions and local stakeholders will also be key to sustainability of the CTH process and EBPs. Of note, coalitions were not involved in setting the research question, designing the intervention protocol or menu of EBPs, or establishing outcome measures. Of note, each of the four participating states has a Community Advisory Board, with multisectoral representation from across the communities that advises the research teams on study implementation.

Overview of CTH phases

For peer review only

The CTH intervention includes seven phases, described in detail elsewhere,[17] that guide coalitions through a systematic partnership process. The intervention began October 23, 2019. The anticipated trial completion date is December 31, 2023. Briefly, the process begins with establishment or identification of a coalition and an environmental scan, or "landscape analysis," of local resources and gaps (Phase 0). During Phase 1, coalitions develop a shared charter and identify key community "champions" to support EBPs, data-driven decision making, and communication campaigns that have been described elsewhere.[19] In Phase 2, coalitions learn about data-driven decision making and the ORCCA menus containing EBP strategies related to overdose education and naloxone distribution (OEND, Menu 1), MOUD (Menu 2), and prescription opioid safety (Menu 3). The ORCCA menus have been described in detail elsewhere.[18] Following review of data describing the local epidemiology of OUD and overdose, simulation modeling data, and/or existing service resources and gaps identified by the landscape analysis (Phase 3), coalitions develop community action plans (one per coalition) by selecting strategies to implement EBPs that are in the ORCCA menu (Phase 4). After creation of the action plan, coalition selected EBP strategies are implemented and monitored (Phase 5) and sustainability plans are created (Phase 6). While these processes are ongoing, a health communication campaign is delivered at the community level to reduce stigma and increase engagement in OEND and MOUD.[19] This paper describes the protocol for coalition planning efforts in Phase 4 to select EBP strategies to achieve the study's primary outcome, reduction in number of opioid overdose deaths. The primary and secondary outcomes of the study and the analysis of the trial data are described in detail elsewhere.[35 36]

Strategies Included in the Community Action Planning Process

For peer review only

Coalitions are provided with guidelines to structure strategy selection in the community action planning process (Figure 1). Coalitions are required to select a minimum of five strategies, including at least one to implement each required EBP from the ORCCA: (a) expansion of proactive offering of OEND to at risk individuals and members of their social networks, (b) expansion of buprenorphine and/or methadone access, (c) improved linkage to MOUD, (d) increased retention in MOUD, and (e) promotion of safer opioid prescribing/dispensing. The EBP strategies are required to include at least one to be implemented in each of the following three sectors: health care, behavioral health and criminal justice.[18] Coalitions are encouraged to consider EBP strategies that would maximize impact in key populations most vulnerable to experiencing an opioid-involved overdose, including those who had a prior opioid overdose;[8 37-40] have reduced opioid tolerance (e.g., completing medically supervised or socially managed withdrawal or release from institutional settings such as jail, residential treatment, hospital);[40-44] use other substances (e.g., alcohol, benzodiazepines, cocaine, and amphetamine like substances);[40 45-49] have concomitant major mental[50-53] or medical illness;[40 54-57] and/or inject drugs.[46 58] Coalitions are also asked to prioritize venues through which these key populations are most likely to be reached. Priority venues for each of the three EBPs (i.e., OEND, MOUD, and safer opioid prescribing and dispensing) are listed in Figure 1 and described in detail elsewhere.[18]

Inherently, the CTH allowed for communities to independently implement EBP strategies at their own pace, including expediting strategies in times of emergency. In June 2020, the HCS approved "fast tracking" certain HCS-supported activities related to OEND in response to the coalitions' concerns about the substantial uptick of opioid overdose observed in the initial months of the SARS-CoV-2 pandemic.[59-66] The protocol allowed HCS-supported naloxone

For peer review only - http://bmjopen.bmj.com/site/about/guidelines.xhtml

distribution to begin in high-risk settings prior to the completion of action planning, but preserved the community-driven nature of the decision-making process. Coalitions were asked whether they wanted the HCS teams to provide technical assistance and/or resources to community venues with greatest reach to individuals at risk for overdose (i.e., jails, syringe service programs) to expand OEND. Upon coalition approval, HCS teams and/or community implementation teams began outreach to high-risk venues to expand OEND. Thus, the fast-tracked OEND activities were ongoing during the Phase 4 action planning process. During the action planning process, coalitions were asked to reflect on the successes and challenges of the fast-tracked strategies and asked to decide whether they wanted to continue or expand OEND activities that had been fast-tracked as part of the action planning process.

Strategy selection and action planning process

The core components of the cross-state protocol for action planning and strategy selection are described in Figure 2. Action planning in each state involves (1) data-driven community goal setting, (2) discussion and prioritization of EBP strategies, (3) selection of EBP strategies to include in the action plan, and (4) identification of next steps for implementation of selected EBP strategies.

Coalitions' movement through the action planning process is facilitated by a combination of goal-setting tools, data visualizations and decision aids. Examples of tools developed by each state for the action planning process are provided in the Supplementary Appendix. The communities currently receiving the CTH intervention differ in geographic unit (i.e., counties, townships, cities), community-readiness for EBP strategy implementation, existing resources and gaps, coalition history (e.g., whether coalitions existed prior to the HCS or convened for

For peer review only

primarily for the HCS), and other contextual factors thereby requiring some variation to the action planning process across and within states (Table 1).

Action planning in each state is driven by coalitions, coalition workgroups, and champions, and is facilitated by a team comprising clinical and research faculty, core staff, and community-based staff with expertise in community-engagement, data, OEND, MOUD, and safer opioid prescribing and dispensing. Coalition composition is described briefly in *Participant Involvement* and in detail elsewhere.[17] Of note, only in MA are *all* agencies to eventually be involved in EBP strategy implementation represented on the coalition. In other states, the agencies to be involved in implementation are represented to varying degrees across coalitions. In NY, community government partners (i.e., health commissioners, county executives) are also involved in the action planning process.

<u>Step 1. Develop Data-driven Community Goals</u>

All coalitions begin the goal-setting process by reviewing community profiles and data dashboards, supplemented in one state (NY) by results of agent-based and systems dynamic modeling. The community profiles, developed through the CTH intervention, included lists of organizations in the community relevant to potential implementation of EBP strategies (e.g., substance use disorder treatment centers, harm reduction programs, emergency response agencies, hospitals). The data dashboards, also developed through the CTH intervention, contain data visualizations on community-specific trends in fatal and non-fatal opioid overdose, MOUD availability, naloxone distribution, and high-risk opioid prescribing.[9]

During and following data review, coalitions discuss strengths, services, gaps, and needs. The coalitions established goals varying in specificity across states and communities (e.g.,

For peer review only

"Increase OEND," "Two emergency departments will initiate MOUD by January 2021"). Coalitions set goals at the ORCCA menu-level (i.e., for OEND, MOUD, and safer opioid prescribing/dispensing) or submenu-level (i.e., for active and passive OEND; for MOUD expansion, linkage, and retention; for safer opioid prescribing/dispensing and disposal). States vary in the other factors their coalitions are asked to consider when setting goals; these factors include sustainability of EBP strategies (NY, MA), EBP strategies' reach to high-risk populations (OH, MA), ability to ensure health equity across ethnic and racial groups (MA, NY), and priority venues for EBP strategy implementation (OH, MA).

Step 2. Discuss and Prioritize EBPs that Align with Community Goals

Following goal setting, coalitions consider specific strategies for implementing the EBPs included on the ORCCA menu and determine which are of highest priority for local implementation. In three states (OH, MA, NY), coalitions brainstorm and free-list EBP strategies that align with the study goals, priority sectors and/or venues, and high-risk populations. In KY, coalitions are provided with initial lists of EBP strategies to consider that are pre-populated from the ORCCA by the academic team. Coalitions in all states consider the EBP strategies in terms of the existing services, size of the gap between existing services and need, feasibility, and potential impact on opioid-involved overdose deaths. To frame the discussion of feasibility more concretely, coalitions in two states (OH, NY) are asked to consider whether the EBP strategy is a new service, scale-up of an existing service via a new method of service delivery, new target population, or new feature to an existing service. In two states (MA, NY), coalitions also consider sustainability. In all states, deliberations on the EBP strategies occur in full coalition meetings or in workgroups led by champions depending on the coalition's preference.

For peer review only

All coalitions begin by providing narrative feedback on each EBP strategy. One state's (KY) coalitions are asked to supplement the narrative feedback by scoring each EBP strategy in terms of size of the existing service gap, feasibility of implementation within the next 12 months, and potential impact on opioid-involved overdose deaths using Likert scales. The scale scores are used to produce a summative priority score to help guide EBP strategy selection. Two states (NY, OH) ask coalitions to rank EBP strategies in terms of impact and feasibility, with the most impactful and feasible EBP strategies scoring highest. In one state (MA), coalitions prioritize EBP strategies through consensus building group dialogues as opposed to ranking.

In three states, coalitions are either required (MA) or encouraged to consider the cost of EBP strategy implementation (NY, OH). In MA, coalitions are provided with an overall budget cap to work within and as part of their planning are required to consider the cost of implementation as they make their EBP strategy selections. In NY, where communities receive funding for staffing and administrative costs and EBP strategy implementation based on population size, coalitions are encouraged, but not required, to consider the cost of EBP strategy implementation as they make their selections. In OH, all communities are provided with fixed and equivalent budgets with limits on expenditures for naloxone and technical assistance; coalitions consider these budget caps as they choose EBP strategies but are not asked to estimate the cost of individual EBP strategies. In KY, coalitions are encouraged *not* to consider cost, but rather to focus on potential need, impact, and feasibility of each EBP strategy.

Step 3. Select EBP strategies to include in the community action plan

In full coalition and coalition subgroup meetings, members review narrative notes, scores, and rankings from the process described above. In two states (KY, OH), coalitions are

also provided with feedback from HCS faculty and staff on implementation feasibility with respect to structural or regulatory barriers. Coalitions weigh these considerations in the context of the guidelines described in Figure 1 and choose through consensus or majority vote the EBP strategies to be implemented with support of HCS. In making their initial selections, coalition members understand that they can later add and refine EBP strategies as capacity expands. Only one state (KY) set a maximum for the number of EBP strategies that could be selected initially, not including OEND strategies already fast-tracked. Coalitions in KY are limited to twelve initial EBP strategies, requiring that they choose no more than three involving OEND, two for MOUD expansion, two for linkage to MOUD, two to improve MOUD retention, and three focused on safer opioid prescribing/dispensing.

Step 4. Identification of next steps for implementation of selected EBP strategies

Once EBP strategies are chosen, coalitions are encouraged to list partner agencies. In three states, coalition-listed agencies are suggested as potential partners for implementation (KY, NY, OH). In MA, the partner agencies for implementation are determined by coalition subgroups as part of the planning process and voted on by the coalitions, with the acceptation of agencies delivering office-based addictions treatment; these agencies were preselected by the study team. Coalition review of selected EBP strategies in one state (KY) also involves members describing what the coalition could do to support rollout (e.g., write op-eds in the local paper in support of the EBP strategy, provide warm handoffs to agencies, etc.) and ways to reach underserved populations with a primary focus on people of color and Spanish speaking individuals. Iteratively with coalition review, HCS faculty and community staff in each state conduct internal reviews. In MA, state government partners are also involved in the internal review. Internal

For peer review only

reviews focus on ensuring the EBP strategies selected adhere to the ORCAA menu and include the required high-risk populations and sectors, potential impact and feasibility. Internal teams also determine next steps for team members, such as initial meetings with partner organizations, contracting considerations, and provision of technical assistance. In one state (MA), the review also includes planning for the mitigation of inequities associated with each EBP strategy and examination of each EBP strategy's cost as estimated by coalitions with technical assistance from community staff and HCS faculty.

Upon completion of the steps described above, descriptions of selected EBP strategies are provided to faculty and staff teams with expertise in implementation science, OEND, MOUD, safer opioid prescribing/dispensing, and/or community engagement to start the process of working with agency partners and explore interest in and capacity for implementation of the coalitions selected EBP strategies. This process varies from site to site and involves the development of an agency specific implementation plan.

Process and Outcome Assessment

Community research staff complete fidelity checklists monthly to document the extent to which all steps are followed and how long each step takes. EBP strategies selected by the coalitions are coded in the form of triads and entered into a harmonized data system shared across the four states. Specifically, the data are coded by venue type (e.g., addiction treatment facility, social services agency, etc), sector (i.e., criminal justice, behavioral health, or healthcare), and ORCCA menu (i.e., OEND, MOUD, or safer opioid prescribing and dispensing). For each entry in the dataset, additional data are recorded and updated throughout the study; these data include but are not limited to the date that the strategy was selected, when it

For peer review only

was implemented, and whether it was successfully implemented. Analysis of the triad data is the focus of another paper and will involve enumeration of strategies selected across communities and by community characteristics. Data are also being collected on the strategies' reach (e.g., number of naloxone units distributed, number of clients initiated on buprenorphine or involved in care navigation, etc). Cost of the intervention, including the community engagement process, is also being assessed, as described in detail elsewhere. To collect data on coalition members' perspectives on the community engagement process, the HEALing Community Study includes repeated coalition member surveys and qualitative interviews which among many other domains, probes respondents on their experiences with the coalition and strengths and weaknesses of the partnership.[67 68]

Ethics and Dissemination

The HEALing Communities Study protocol was approved by a central Institutional Review Board (IRB; Advarra Inc., Pro00038088) and is being conducted in accordance with The Code of Ethics of the World Medical Association (Declaration of Helsinki). As described elsewhere,[16] because no one person or group of people possess(es) the authority to give consent on behalf of all community members, investigators sought expert consultation and applied guidelines from the Ottawa Statement.[69] Because the CTH intervention poses no more than minimal risk to community members and the research could not be carried out otherwise (see 45.CFR.46.116), a waiver of informed consent was obtained for all community members who may be affected by the CTH intervention.

The action planning tools are disseminated to coalition members in hardcopy, via email, and/or through a password-protected, online portal. We plan to disseminate updated versions of

For peer review only

these tools to communities currently in the waitlist-control group via the same mechanisms when intervention begins in their communities. Results of the action planning process (i.e., EBP strategy selections) are shared with community members, community agencies, and community advisory boards through meetings, online and print media, and presentations to community stakeholder groups in the intervention communities. In the future, we also plan to disseminate our findings at relevant conferences, meetings and through peer-reviewed journals. Finally, we will disseminate our findings, manuals, toolkits, and other resources through federal partners including the National Institutes of Health, Substance Abuse and Mental Health Services Administration and the Centers for Disease Control and Prevention.

For peer review only

Table 1. Characteristics of action planning process that were unique to certain states

**Action Planning Steps**	**Characteristics**	**KY**	**MA**	**NY**	**OH**
Step 1. Develop Data-driven Community Goals	*Supplementary factors considered by coalitions:*				
	• Agent-based and systems dynamic modeling			X	
	• EBP strategy sustainability		X	X	
	• Reach to high-risk populations and priority venues		X		X
	• Equity across ethnic and racial groups		X	X	
Step 2. Discuss and Prioritize EBP strategies	Development of EBP strategy list considered by coalition	Pre-populated by the academic team	Generated by coalitions[1]	Generated by coalitions[1]	Generated by coalitions[1]
	Method of prioritizing strategies	Likert scale ratings of size of current service gap, feasibility, and potential impact.	Consensus building group dialogues	Rank by impact and feasibility	Rank by impact and feasibility
	Coalition consideration of cost	Encouraged *not* to consider cost, but to focus on need, impact, and feasibility of each EBP strategy.	Provided with budget cap and required to consider costs	Encouraged, but not required	Encouraged, but not required
	Supplementary factors considered by coalitions:				
	• EBP strategy sustainability		X	X	
	• Method of EBP expansion (i.e., new service, or scale-up of an existing service via a new method of delivery, target population, or feature)			X	X

For peer review only - http://bmjopen.bmj.com/site/about/guidelines.xhtml

**Action Planning Steps**	**Characteristics**	**KY**	**MA**	**NY**	**OH**
Step 3. Select EBP strategies to include in action plan	Limits on strategy selections	12 initial EBP strategies	No limit	No limit	No limit
Step 4. Identify next steps for implementation of selected EBP strategies	Coalition input on agencies to be involved in implementation	Coalitions suggest potential agencies to involve in implementation.	Coalition subgroups determine which agencies will be involved in implementation.	Coalitions suggest potential agencies to involve in implementation.	Coalitions suggest potential agencies to involve in implementation.
	Coalition input on next steps for beginning implementation	Coalition describes what they can do to support implementation and reach underserved populations who may experience disparities in access.	Coalitions estimate cost of each strategy with technical assistance from the academic team.	None beyond action plan established in Step 3	None beyond action plan established in Step 3

EBP: evidence-based practices

[1] "Coalitions" is inclusive of full coalitions, coalition workgroups, and coalition champions with expertise in specific EBPs

Authors' contributions

The following individuals contributed to the design of the action planning protocol described in this manuscript: AY, PRF, LSM, LF, MRL, JB, MSL, TH, AB, BR, RBS, and TAB. The following individuals made substantial contributions to the implementation of the action planning protocol: AY, PRF, AF, LF, JG, CK, MRL, HS, SLW, JB, MSL, TH, AB, and MQ. The following individuals made substantial contributions to the drafted work and/or substantively revised it: AY, JB, TH, LSM, RC, EO, TW, TAB, TB, RBS, AB, AF, LF, LMG, PRF, JG, CK, MSL, MRL, MQ, BR, HS, SLW. All authors read and approved the final manuscript and agree to be accountable for all aspects of the work.

Funding statement

This research was supported by the National Institutes of Health through the NIH HEAL (Helping to End Addiction Long-term^{SM}) Initiative under award numbers UM1DA049394, UM1DA049406, UM1DA049412, UM1DA049415, UM1DA049417 (ClinicalTrials.gov Identifier: NCT04111939). The Substance Abuse and Mental Health Services Administration (SAMHSA) is a non-funding sponsor of the study. This study protocol (Pro00038088) was approved by Advarra Inc., the HEALing Communities Study single Institutional Review Board. We wish to acknowledge the participation of the HEALing Communities Study communities, community coalitions, and Community Advisory Boards and state government officials who partnered with us on this study. The content is solely the responsibility of the authors and does not necessarily represent the official views of the National Institutes of Health, the Substance Abuse and Mental Health Services Administration or the NIH HEAL Initiative^{SM}. R. Chandler was substantially involved in this paper, consistent with her role as Scientific Officer.

Competing interest statement

Competing interest disclosures include the following: LSM is a consultant for the Boston Public Health Commission, Action for Boston Area development and the Young Foundation, SLW has served as a scientific advisor to Opiant Pharmaceuticals, Titan Pharmaceuticals, Astra Zeneca, Cerevel, Arbor Pharmaceuticals, Brainsway, Summit Biosciences, Trevi and Camurus. JB ad ML receive investigator-initiated support paid to the institution from Gilead Sciences, Inc. MRL has consulted for Titan Pharmaceuticals and Camurus. AFB is the Program Director of Voices of Hope, a University of Kentucky HCS partner organization. PRF, HS, AY, EO, LMG, RC, JG, CK and LCF have no competing interests.

Data Sharing Statement

The protocol described in this manuscript is for a community-engaged action planning process by which communities choose strategies to implement evidence-based practices in their communities. Data for this paper are considered to be the tools such as decision aids, action plans, and other documents used to facilitate the process and are included as a Supplementary Appendix. The protocol described in this manuscript is part of a larger trial for which data are of a different type; those data are described in the trial registry.

Acknowledgments

We wish to acknowledge the participation of the HEALing Communities Study communities, community coalitions, and Community Advisory Boards and state government officials who partnered with us on this study. We extend our sincere gratitude to the staff and community

For peer review only

members who have dedicated their time and passion to this project and without whom the HEALing Communities Study would not be possible.

For peer review only

References

1. Krausz RM, Westenberg JN, Ziafat K. The opioid overdose crisis as a global health challenge. *Current Opinion in Psychiatry* 2021;34(4):405-12.

2. World Health Organization. Opioid overdose 2021 [updated August 4 2021. Available from: https://www.who.int/news-room/fact-sheets/detail/opioid-overdose accessed August 15 2021.

3. National Institute on Drug Abuse. Overdose Death Rates Bethesda, MD2021 [updated January 29, 2021. Available from: https://www.drugabuse.gov/drug-topics/trends-statistics/overdose-death-rates accessed August 15 2021.

4. Ahmad FB RL, Sutton P. Provisional drug overdose death counts: National Center for Health Statistics. Designed by LM Rossen, A Lipphardt, FB Ahmad, JM Keralis, and Y Chong: National Center for Health Statistics., 2021.

5. Strang J, Volkow ND, Degenhardt L, et al. Opioid use disorder. *Nature Reviews Disease Primers* 2020;6(1):3. doi: 10.1038/s41572-019-0137-5

6. Bohnert AS, Guy Jr GP, Losby JL. Opioid prescribing in the United States before and after the Centers for Disease Control and Prevention's 2016 opioid guideline. *Annals of internal medicine* 2018;169(6):367-75.

7. Walley AY, Xuan Z, Hackman HH, et al. Opioid overdose rates and implementation of overdose education and nasal naloxone distribution in Massachusetts: interrupted time series analysis. *British Medical Journal* 2013;346

8. Larochelle MR, Bernson D, Land T, et al. Medication for opioid use disorder after nonfatal opioid overdose and association with mortality: a cohort study. *Annals of internal medicine* 2018;169(3):137-45.

9. Wu E, Villani J, Davis A, et al. Community dashboards to support data-informed decision-making in the HEALing communities study. *Drug and alcohol dependence* 2020;217:108331-31. doi: 10.1016/j.drugalcdep.2020.108331

10. Welty L, Harrison A, Abram K, et al. Substance Abuse and Mental Health Services Administration.(2017). Key substance use and mental health indicators in the United States: Results from the 2016 National Survey on Drug Use and Health (HHS Publication No. SMA 17-5044, NSDUH Series H-52). Rockville, MD: Center for Behavioral Health Statistics and Quality. *Substance Abuse and Mental Health Services Administration Retrieved College of Health Sciences* 2019;106(5):128.

11. Williams AR, Nunes EV, Bisaga A, et al. Developing an opioid use disorder treatment cascade: A review of quality measures. *Journal of substance abuse treatment* 2018;91:57-68.

For peer review only - http://bmjopen.bmj.com/site/about/guidelines.xhtml

12. Freeman PR, Hankosky ER, Lofwall MR, et al. The changing landscape of naloxone availability in the United States, 2011–2017. *Drug and alcohol dependence* 2018;191:361-64.

13. Guy Jr GP, Zhang K, Bohm MK, et al. Vital signs: changes in opioid prescribing in the United States, 2006–2015. *MMWR Morb Mortal Wkly Rep* 2017;66(26):697.

14. Guy Jr GP, Zhang K, Halpin J, et al. An examination of concurrent opioid and benzodiazepine prescribing in 9 states, 2015. *American journal of preventive medicine* 2019;57(5):629-36.

15. Guy GP, Zhang K, Schieber LZ, et al. County-level opioid prescribing in the United States, 2015 and 2017. *JAMA internal medicine* 2019;179(4):574-76.

16. Walsh SL, El-Bassel N, Jackson RD, et al. The HEALing (Helping to End Addiction Long-term SM) Communities Study: Protocol for a cluster randomized trial at the community level to reduce opioid overdose deaths through implementation of an integrated set of evidence-based practices. *Drug and Alcohol Dependence* 2020;217:108335.

17. Martinez LS, Rapkin BD, Young A, et al. Community engagement to implement evidence-based practices in the HEALing Communities Study. *Drug and Alcohol Dependence* 2020;217:108326.

18. Winhusen T, Walley A, Fanucchi LC, et al. The opioid-overdose reduction continuum of care approach (ORCCA): evidence-based practices in the HEALing communities study. *Drug and Alcohol Dependence* 2020;217:108325.

19. Lefebvre RC, Chandler RK, Helme DW, et al. Health communication campaigns to drive demand for evidence-based practices and reduce stigma in the HEALing Communities Study. *Drug and alcohol dependence* 2020;217:108338.

20. Rosenthal BB, Greenawalt J, Ivery JM, et al. Coalitions: Essential tools for organizing. In: Staples L, ed. Roots to Power: A Manual for Grassroots Organizing: A Manual for Grassroots Organizing. London: Praeger 2016:297.

21. Oesterle S, Kuklinski MR, Hawkins JD, et al. Long-Term Effects of the Communities That Care Trial on Substance Use, Antisocial Behavior, and Violence Through Age 21 Years. *Am J Public Health* 2018;108(5):659-65.

22. Shapiro VB, Hawkins JD, Oesterle S. Building local infrastructure for community adoption of science-based prevention: The role of coalition functioning. *Prevention Science* 2015;16(8):1136-46.

23. Arthur MW, Hawkins JD, Brown EC, et al. Implementation of the Communities That Care prevention system by coalitions in the Community Youth Development Study. *Journal of Community Psychology* 2010;38(2):245-58.

24. Butterfoss FD. Coalitions and partnerships in community health: John Wiley & Sons 2007.

For peer review only

25. Wandersman A, Goodman RM, Butterfoss FD. Understanding coalitions and how they operate. In: Minkler M, ed. Community organizing community building for health. 2 ed. New Brunswick, NJ Rutgers University Press 1997:261-77.

26. Powell BJ, McMillen JC, Proctor EK, et al. A compilation of strategies for implementing clinical innovations in health and mental health. *Medical care research review* 2012;69(2):123-57.

27. Rugs D, Hills HA, Moore KA, et al. A community planning process for the implementation of evidence-based practice. *Evaluation and Program Planning* 2011;34(1):29-36.

28. Pellecchia M, Mandell DS, Nuske HJ, et al. Community–academic partnerships in implementation research. *Journal of Community Psychology* 2018;46(7):941-52.

29. Centers for Disease Control and Prevention. Principles of Community Engagement. Second ed, 2011.

30. World Health Organization. WHO community engagement framework for quality, people-centred and resilient health services. : World Health Organization, 2017.

31. World Health Organization. Community engagement: a health promotion guide for universal health coverage in the hands of the people . Geneva: World Health Organization,,, 2020.

32. C. D. I. Study Group. Community-directed interventions for priority health problems in Africa: results of a multicountry study. *Bull World Health Organ* 2010;88(7):509-18. doi: 10.2471/BLT.09.069203 [published Online First: 2009/12/01]

33. World Health Organization & United Nations Children's Fund (UNICEF). Conducting community engagement for COVID-19 vaccines: interim guidance, 31 January 2021: World Health Organization, 2021.

34. US Department of Health and Human Services. Biden-Harris Administration Bolsters Monkeypox Response; HHS Secretary Becerra Declares Public Health Emergency. Washington, D.C., 2022.

35. Slavova S, LaRochelle MR, Root ED, et al. Operationalizing and selecting outcome measures for the HEALing Communities Study. *Drug and alcohol dependence* 2020;217:108328.

36. Westgate PM, Cheng DM, Feaster DJ, et al. Marginal modeling in community randomized trials with rare events: Utilization of the negative binomial regression model. *Clinical Trials* 2022:17407745211063479.

37. Winhusen T, Theobald J, Lewis D, et al. Development and initial testing of a tailored telephone intervention delivered by peers to prevent recurring opioid-overdoses (TTIP-PRO). *Health education research* 2016;31(2):146-60. doi: 10.1093/her/cyw010

For peer review only

38. Caudarella A, Dong H, Milloy MJ, et al. Non-fatal overdose as a risk factor for subsequent fatal overdose among people who inject drugs. *Drug Alcohol Depend* 2016;162:51-5. doi: 10.1016/j.drugalcdep.2016.02.024

39. Darke S, Mills KL, Ross J, et al. Rates and correlates of mortality amongst heroin users: findings from the Australian Treatment Outcome Study (ATOS), 2001-2009. *Drug Alcohol Depend* 2011;115(3):190-5. doi: 10.1016/j.drugalcdep.2010.10.021 [published Online First: 2010/12/07]

40. Larochelle MR, Bernstein R, Bernson D, et al. Touchpoints - Opportunities to predict and prevent opioid overdose: A cohort study. *Drug and alcohol dependence* 2019;204:107537-37. doi: 10.1016/j.drugalcdep.2019.06.039 [published Online First: 2019/09/03]

41. Strang J, McCambridge J, Best D, et al. Loss of tolerance and overdose mortality after inpatient opiate detoxification: follow up study. *British Medical Journal* 2003;326(7396):959-60. doi: 10.1136/bmj.326.7396.959

42. Binswanger IA, Stern MF, Deyo RA, et al. Release from prison—a high risk of death for former inmates. *New England Journal of Medicine* 2007;356(2):157-65. doi: 10.1056/NEJMsa064115

43. Merrall ELC, Kariminia A, Binswanger IA, et al. Meta-analysis of drug-related deaths soon after release from prison. *Addiction* 2010;105(9):1545-54. doi: 10.1111/j.1360-0443.2010.02990.x

44. Walley AY, Lodi S, Li Y, et al. Association between mortality rates and medication and residential treatment after in-patient medically managed opioid withdrawal: a cohort analysis. *Addiction (Abingdon, England)* 2020;115(8):1496-508. doi: 10.1111/add.14964 [published Online First: 2020/02/25]

45. Sun EC, Dixit A, Humphreys K, et al. Association between concurrent use of prescription opioids and benzodiazepines and overdose: retrospective analysis. *British Medical Journal* 2017;356:j760-j60. doi: 10.1136/bmj.j760

46. Brugal MT, Barrio G, De LF, et al. Factors associated with non-fatal heroin overdose: assessing the effect of frequency and route of heroin administration. *Addiction* 2002;97(3):319-27. doi: 10.1046/j.1360-0443.2002.00058.x [published Online First: 2002/04/20]

47. Cho J, Spence MM, Niu F, et al. Risk of Overdose with Exposure to Prescription Opioids, Benzodiazepines, and Non-benzodiazepine Sedative-Hypnotics in Adults: a Retrospective Cohort Study. *J Gen Intern Med* 2020;35(3):696-703. doi: 10.1007/s11606-019-05545-y [published Online First: 2020/01/09]

48. Gladden RM, O'Donnell J, Mattson CL, et al. Changes in Opioid-Involved Overdose Deaths by Opioid Type and Presence of Benzodiazepines, Cocaine, and Methamphetamine - 25

For peer review only

States, July-December 2017 to January-June 2018. *MMWR Morb Mortal Wkly Rep* 2019;68(34):737-44. doi: 10.15585/mmwr.mm6834a2

49. Park TW, Larochelle MR, Saitz R, et al. Associations between prescribed benzodiazepines, overdose death and buprenorphine discontinuation among people receiving buprenorphine. *Addiction (Abingdon, England)* 2020;115(5):924-32. doi: 10.1111/add.14886 [published Online First: 2020/01/20]

50. O'Driscoll PT, McGough J, Hagan H, et al. Predictors of accidental fatal drug overdose among a cohort of injection drug users. *American journal of public health* 2001;91(6):984-87. doi: 10.2105/ajph.91.6.984

51. Pabayo R, Alcantara C, Kawachi I, et al. The role of depression and social support in non-fatal drug overdose among a cohort of injection drug users in a Canadian setting. *Drug and alcohol dependence* 2013;132(3):603-09. doi: 10.1016/j.drugalcdep.2013.04.007 [published Online First: 2013/05/04]

52. Wines JD, Jr., Saitz R, Horton NJ, et al. Overdose after detoxification: a prospective study. *Drug Alcohol Depend* 2007;89(2-3):161-9. doi: 10.1016/j.drugalcdep.2006.12.019 [published Online First: 2007/02/07]

53. Tobin KE, Latkin CA. The relationship between depressive symptoms and nonfatal overdose among a sample of drug users in Baltimore, Maryland. *J Urban Health* 2003;80(2):220-29. doi: 10.1093/jurban/jtg025

54. Bosilkovska M, Walder B, Besson M, et al. Analgesics in patients with hepatic impairment: pharmacology and clinical implications. *Drugs* 2012;72(12):1645-69. doi: 10.2165/11635500-000000000-00000 [published Online First: 2012/08/08]

55. Green TC, McGowan SK, Yokell MA, et al. HIV infection and risk of overdose: a systematic review and meta-analysis. *AIDS* 2012;26(4):403-17. doi: 10.1097/QAD.0b013e32834f19b6

56. Jolley CJ, Bell J, Rafferty GF, et al. Understanding Heroin Overdose: A Study of the Acute Respiratory Depressant Effects of Injected Pharmaceutical Heroin. *PLoS One* 2015;10(10):e0140995-e95. doi: 10.1371/journal.pone.0140995

57. Vu Q, Beselman A, Monolakis J, et al. Risk factors for opioid overdose among hospitalized patients. *J Clin Pharm Ther* 2018;43(6):784-89. doi: 10.1111/jcpt.12701 [published Online First: 2018/05/26]

58. Bazazi AR, Zelenev A, Fu JJ, et al. High prevalence of non-fatal overdose among people who inject drugs in Malaysia: Correlates of overdose and implications for overdose prevention from a cross-sectional study. *International Journal on Drug Policy* 2015;26(7):675-81. doi: 10.1016/j.drugpo.2014.11.010

For peer review only

59. Slavova S, Quesinberry D, Hargrove S, et al. Trends in Drug Overdose Mortality Rates in Kentucky, 2019-2020. *JAMA network open* 2021;4(7):e2116391-e91. doi: 10.1001/jamanetworkopen.2021.16391

60. Slavova S, Rock P, Bush HM, et al. Signal of increased opioid overdose during COVID-19 from emergency medical services data. *Drug and alcohol dependence* 2020;214:108176-76. doi: 10.1016/j.drugalcdep.2020.108176

61. Stephenson J. Drug Overdose Deaths Head Toward Record Number in 2020, CDC Warns. *JAMA Health Forum* 2020;1(10):e201318-e18. doi: 10.1001/jamahealthforum.2020.1318

62. Wakeman SE, Green TC, Rich J. An overdose surge will compound the COVID-19 pandemic if urgent action is not taken. *Nature Medicine* 2020;26(6):819-20. doi: 10.1038/s41591-020-0898-0

63. Holland KM, Jones C, Vivolo-Kantor AM, et al. Trends in US emergency department visits for mental health, overdose, and violence outcomes before and during the COVID-19 pandemic. *JAMA psychiatry* 2021;78(4):372-79.

64. Friedman J, Beletsky L, Schriger DL. Overdose-related cardiac arrests observed by emergency medical services during the US COVID-19 epidemic. *JAMA psychiatry* 2021;78(5):562-64.

65. Centers for Disease Control and Prevention. Overdose Deaths Accelerating During COVID-19 2020 [Available from: https://www.cdc.gov/media/releases/2020/p1218-overdose-deaths-covid-19.html accessed May 19 2021.

66. Centers for Disease Control and Prevention. Increase in Fatal Drug Overdoses Across the United States Driven by Synthetic Opioids Before and During the COVID-19 Pandemic: CDC Health Alert Network, 2020.

67. Aldridge AP, Barbosa C, Barocas JA, et al. Health economic design for cost, cost-effectiveness and simulation analyses in the HEALing Communities Study. *Drug and alcohol dependence* 2020;217:108336.

68. Knudsen HK, Drainoni M-L, Gilbert L, et al. Model and approach for assessing implementation context and fidelity in the HEALing Communities Study. *Drug and alcohol dependence* 2020;217:108330.

69. Taljaard M, Weijer C, Grimshaw JM, et al. The Ottawa Statement on the ethical design and conduct of cluster randomised trials: precis for researchers and research ethics committees. *British Medical Journal* 2013;346:f2838. doi: 10.1136/bmj.f2838

For peer review only

Figure 1. Guidelines for HEALing Communities Study Community Action Planning Process to reduce opioid overdose deaths through overdose education and naloxone distribution, expansion of medications for opioid use disorder, and safer opioid prescribing and dispensing

For peer review only

Figure 2. Core components of the HEALing Communities Study Community Action Planning Process to reduce opioid overdose deaths through overdose education and naloxone distribution, medications for opioid use disorder, and safer opioid prescribing and dispensing

For peer review only

- Coalitions *must* select a minimum of five strategies for implementing evidence-based practices (EBPs) from the ORCCA menus including one involving each of the following
  - ORCCA Menu 1: Active OEND (i.e., proactively offer OEND to those at risk)
  - ORCCA Menu 2A: Expand MOUD access
  - ORCCA Menu 2B: Increase linkage to MOUD
  - ORCCA Menu 2C: Improve retention in MOUD
  - ORCCA Menu 3: Safer opioid prescribing/dispensing

- Priority sectors: Selected EBP strategies *must* include at least one to be implemented in each of the following:
  - Healthcare (i.e., outpatient healthcare centers, pre-hospital providers, emergency departments and urgent care, hospitals, primary care settings, public health departments, and pharmacies)
  - Behavioral health (i.e., substance use disorder and mental health treatment centers and social service agencies, including syringe service programs)
  - Criminal justice (i.e., pre-trial, jails, probation, parole, drug and problem-solving courts, police and "narcotics" task forces, halfway houses, community-based correctional facilities, and department of youth services)

- Key populations: Coalitions are encouraged to consider EBP strategies focused on those most vulnerable to opioid overdose:
  - People with a prior opioid overdose
  - People with reduced opioid tolerance
  - People who use other substances
  - People who have concomitant major mental illness
  - People who have a concomitant major medical illness
  - People who inject drugs

- Priority venues: Coalitions are encouraged to consider EBP strategies to be implemented in priority settings:
  - OEND: criminal justice settings, syringe service programs, emergency departments and acute care hospitals, sites of overdose for naloxone leave-behind, and mental health/addiction treatment programs
  - MOUD: criminal justice settings, primary care, general medical and behavioral/mental health settings, specialty addiction/substance use disorder treatment settings, and recovery programs
  - Safer opioid prescribing and dispensing: inpatient service, emergency/urgent care, outpatient clinics, ambulatory surgery, dental clinics, pharmacies

Figure 1. Guidelines for HEALing Communities Study Community Action Planning Process to reduce opioid overdose deaths through overdose education and naloxone distribution, expansion of medications for opioid use disorder, and safer opioid prescribing and dispensing

165x210mm (330 x 330 DPI)

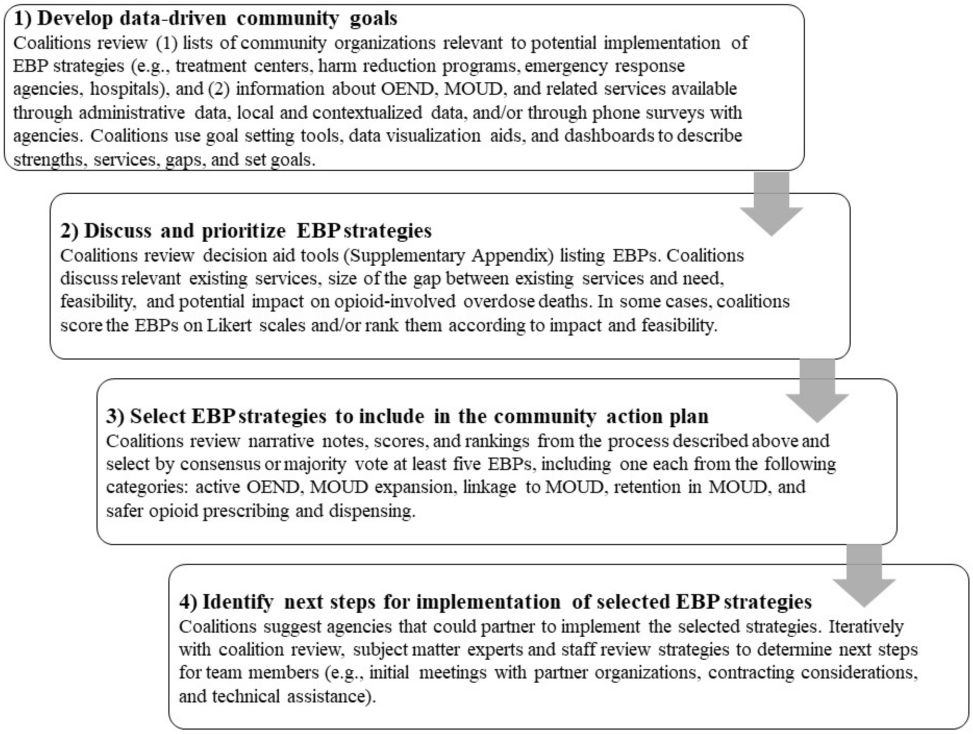

Figure 2. Core components of the HEALing Communities Study Community Action Planning Process to reduce opioid overdose deaths through overdose education and naloxone distribution, medications for opioid use disorder, and safer opioid prescribing and dispensing

254x190mm (96 x 96 DPI)

Appendix 1. Kentucky Action Planning Tools

Community Goal Setting Tool

Purpose: To describe existing infrastructure, gaps, opportunities, needs, and goals related to opioid overdose prevention.

Description: The Kentucky Community Engagement and Continuum of Care teams partnered to facilitate communities' identification of their strengths and opportunities for expansion related to opioid overdose prevention followed by thoughtful consideration and selection of the ORCCA strategies. The process began with the Community Engagement team working with the coalition to describe existing infrastructure, gaps, opportunities, needs, and goals related to opioid overdose prevention in their communities using a Community Goal Setting Tool. The coalitions and/or menu-specific workgroups worked on completing the tool for each menu separately and presenting them to the full coalition.

Who completes the tool: Coalitions and/or coalition workgroups with facilitation by faculty and staff.

Community Goal Setting Tool — Menu 1

Menu 1 Goal: Increase Opioid Overdose Prevention Education and Naloxone Distribution (OEND)

Brainstorm! Think about your community: what you've experienced, what you learned from your community profile and data dashboard, and what you envision for the future. Answer the questions below and jot down your thoughts

What are we doing well? What OEND services already exist?

Ex. There is active Opioid Overdose Education and Naloxone Distribution (OEND) already occurring in the County Detention Center. The Detention Center is currently able to provide naloxone to all persons released from the facility (as part of partnership with the HEALing Communities Study) and is also partnering with other organizations to distribute naloxone to anyone who is interested and completes the requisite virtual training.

Who needs OEND in our community?

Ex. There is still a significant need for distribution among the peer community and their social networks, in homes with bystanders, and with medical and law enforcement professionals in contact with those at high-risk for overdose.

Where in our community should OEND services be provided?

Ex. There is significant opportunity for OEND expansion in Emergency Departments and the healthcare system generally, in pharmacies, with local Quick Response Teams (QRTs), in Substance Use Disorder (SUD) treatment facilities, and in other locations where the peer community can be found. The NKY Health Department also has capacity to expand OEND services.

Community OEND Goals:

Example:

1. Expand OEND in criminal justice settings, including Probation & Parole and Drug Court.
2. Expand OEND in healthcare settings, including Emergency Departments and Substance Use Disorder (SUD) treatment programs.
3. Expand OEND access among peer community and their support systems (via recovery support groups, social service agencies, and in "hot spots" frequented by those at high-risk, etc.).
4. Expand OEND with local QRTs.
5. Increase first responders carrying/administering naloxone.

Decision Aid Tool

Purpose: To facilitate discussion and prioritization of evidence-based practice strategies that align with community goals and the ORCCA

Description: HCS staff then populated menu-specific Decision Aid Tools with strategies listed in the ORCCA and TAG, integrating coalition's suggestions for specific strategies when available from Community Goal Setting Tool. In meetings facilitated by faculty and staff with expertise in community-engagement and the continuum of care, the coalitions and/or menu-specific workgroups reviewed each possible strategy one-by-one, offering a narrative description of factors that they believed would be relevant to deciding whether to implement the strategy (i.e., current relevant activities, barriers they anticipate encountering, agencies relevant to implementation). After offering a narrative description, the coalition scored on Likert scales the size of the current gap in the service/strategy (4-point scale; 0-3), feasibility of implementation within the next 12 months (4-point scale; 0-3), and its potential impact on overdose deaths (3-point scale; 1-3). Each dimension was scored through consensus or through in-meeting polling. Coalitions did not use specific criteria or thresholds in assigning the scores, rather they often scored them based on how they compared to others on the menu. Scores were summed to produce an overall "priority score" for each strategy.

Who completes the tool: Coalitions and/or coalition workgroups with facilitation by faculty and staff.

For peer review only

Menu 2: Strategies to Enhance Delivery of MOUD Maintenance Treatment, Including Agonist/Partial Agonist Medication					
**Strategy and Venue**	**Current Activity**	**Size of Gap** (Current Activity vs. Need)	**Feasibility in <12 Months**	**Impact on Overdose Deaths**	**Priority Score**
Working with the ORCCA menu, brainstorm possible strategies to meet the Community MOUD goals. Record all strategies in this column.	Use this column to briefly summarize current activity in your community	0. None<br>1. Small<br>2. Medium<br>3. Large	0. Extremely Low<br>1. Small<br>2. Medium<br>3. Large	1. Small<br>2. Medium<br>3. Large	Add previous 3 columns; if any column contains 0, priority score is 0
**a) Expand MOUD Treatment Availability (Capacity Building) – REQUIRED**					
**Add/expand MOUD in primary care, other general medical/BH settings, and specialty addiction/SUD & recovery programs**					
Ex. Expand Methadone in county OTPs 2.a.1.2	Ex. Have two OTP providers but one has long wait times; need to figure out what they need to be able to treat more patients				
**Add/expand MOUD treatment in Criminal Justice Settings (pre trial, jail, prison, probation, parole)**					
Ex. Expand prescription of XR naltrexone and buprenorphine within jail	Ex. Interest in XR-naltrexone at the jail and jail might be open but have a lot of concerns/barriers				
**Expand access to MOUD through telemedicine, interim buprenorphine or methadone, or medication units**					
Ex. Expand use of Telemedicine/telehealth/tele-consults for buprenorphine treatment	Ex. Telehealth opportunities have grown due to COVID 19, but it is unknown whether this option will remain available.				
**b) Interventions to Link to MOUD – REQUIRED**					
**Linkage Programs (All Relevant settings)**					
Ex. Develop linkage program for incarcerated individuals about to be released (with peer support and/or other staff)	Ex. No current activity, the jail is somewhat interested but there are several barriers				
**Bridging MOUD medications as Linkage Adjunct (all relevant settings)**					
Ex. Expand bridge programs within county ER and inpatient settings	Ex. An existing bridge program is working well and can use as model for others. Difficulty with staffing & infrastructure is main barrier				
**c) MOUD Treatment Engagement and Retention – REQUIRED**					
**Enhancement of clinical delivery approaches that support engagement and retention**					
Ex. Expand recovery support services (peer support, telephone recovery support, recovery coaching, family support) in criminal justice settings (Voices of Hope)	Ex. Add peer supports within system; no current long term supports, or programs/mentorship programs engaged				
**Use virtual retention approaches (e.g. mobile, web, digital therapeutics)**					
Ex. Implement use of re-SET-0 app to improve retention, incentives, and education 2.c.2	Ex. Some clinics within county already using it; potential cost barriers and potential disinterest from buprenorphine providers				
**Utilize retention care coordinators**					
Ex. Utilize retention care navigators to increase/add care navigators in all MOUD treatment settings 2.c.3	Ex. Other care navigation efforts are already underway that could be leveraged, and there is a desire to increase support of and leverage local efforts where possible				
**Integrate mental health and polysubstance use treatment into MOUD care**					
Ex. Offer regular training between primary care/non MH providers with addiction psychiatry and other MH providers to manage co-morbidity with OUD	Ex. Unsure if providers would be interested in engaging				
**Reduce barriers to housing, transportation, childcare, and access to other community benefits for People with OUD**					
Expand transportation services/offerings to align with MOUD treatment center services (location, hours, etc.)	Ex. Local agency offers transportation services to clients				

Action Planning Tool

<u>Purpose</u>: To facilitate discussion and final strategy selection.

<u>Description</u>: The list of strategies discussed in the Decision Aid Tool was used to pre-populate an Action Planning Tool. The tool listed all strategies with their scores and new implementation feasibility ratings (High, Medium, Low) provided by faculty with expertise in implementation science and the continuum of care based on their knowledge of policy- and payor-level barriers and time required for scale up. The pre-populated Action Planning Tools were presented to coalitions for discussion and strategy selection through consensus.

<u>Who completes the tool</u>: Coalitions and/or coalition workgroups with facilitation by faculty and staff.

Action Planning Tool — Menu 3

For each strategy from the Decision Aid that had a priority score above 0, fill in the strategy and venue (i.e., from Column 1 of the Decision Aid), record the scores from the Decision Aid Tool in the Scores columns, and fill in the relevant sector. As coalitions consider their selection of strategies, they should ensure that strategies collectively will target those at highest risk (i.e., people with history of prior overdose, reduced opioid tolerance, who engage in polydrug use, with major medical or mental illness, and/or who inject drugs). The HCS Research Team will help you complete the Implementation Feasibility column by providing insight on whether each strategy has a high, medium, or low feasibility of implementation based on resources, partnerships, and other factors. After reviewing content of the other columns, coalitions should come to consensus on the Overall Priority Ranking with the highest priority strategy ranked 1, the second highest priority ranked 2, and so on. Of note, the Overall Priority Ranking may or may not correspond directly to the rank order from the other scoring columns. After completing this tool, the next step will involve creating an initial implementation plan for the top 1-3 overall priority ranked strategies; at least one of the strategies must be from the required sub-menu (i.e., Safe Prescribing/Dispensing).

Menu 3: Strategies to Improve Prescription Opioid Safety							
	Scores						
Strategy and Venue	G	F	I	PS	Sector?	Implementation Feasibility? (High/Medium/Low)	Overall Priority Ranking
**a) Safer Opioid Prescribing/Dispensing Practices (Required)**							
Ex. Education materials around safe opioid disposal, targeting healthcare providers and patients.	3	3	2.25	8.25	HC	High	1
Ex. Education for community pharmacists focusing on red flags during opioid dispensing (dose, indication, duration, concurrent therapies, comorbid conditions, age, etc.).	1.25	2.5	1.5	5.25	HC	High	2
Ex. Educational outreach and academic detailing related to pain management for patients with OUD	2.5	2	2	6.5	HC	Medium	
Ex. Patient educational materials regarding appropriate chronic pain management made available in AA meetings and OUD recovery groups	2.5	2.25	1.75	6.5	BH	Medium	3
**b) Safer Opioid Disposal Practices (Optional)**							
Ex. Installation of permanent disposal kiosks	1.8	2.7	2	6.5	HC	High	4
Ex. Expansion and promotion of take-back events	2.3	2.3	2.25	6.85	Other	Medium	

Priority Score: G: Gap; F: Feasibility; I: Impact; PS: Overall Priority Score

Sector: CJ = Criminal Justice, HC = Healthcare, BH = Behavioral Health (See ORCCA Overview for details on assignment of sectors).

Coalition Implementation Planning Tool

Purpose: To more thoroughly define strategies and generate potential lists of venues/agencies to be involved in the implementation

Description: The tool also captured coalition's feedback on what they could do to help support implementation, strategies to ensure reach to under-represented populations, and indicators of success or failure of the strategy. Faculty with expertise in implementation science and the continuum of care added details on what the HCS faculty and staff would do to implement the strategy. Once complete, the tools were reviewed and approved in their entirety by the coalitions.

Who completes the tool: Coalitions and/or coalition workgroups with faculty and staff.

Coalition Implementation Planning Tool — Menu 3

For at least one but no more than three of the strategies with the top Overall Ranking from the Action Plan table, create an initial implementation plan using the table below (i.e., one table per strategy for up to three strategies). At least one of these strategies must come from the required sub-menu in the action plan (i.e., must address safe prescribing/dispensing practices). Provide information about specific activities in as much detail as is feasible at this time; insert additional rows as needed to describe these activities for the coalition and the HCS team.

**Menu 3 Overall Ranking = 1**<br>**Strategy: Educational outreach and academic detailing related to opioid prescribing for routine pain management. (Includes multiple sub-strategies as outlined on the Community Action Planning Tool).**		
**What specifically will be done?** Ex: Educational outreach and academic detailing for all willing opioid prescribers (and possibly ambulatory pharmacists) in the County. Top priorities are: Primary Care, Hospital Emergency Department & local Urgent Care facilities, Dental Offices, Ortho Practices, Pain Management Clinics		
**What organization(s) should be involved? Provide points of contact if possible.**	Agency: Ex. Family Practice	Contact: Ex. Dr. Smith
	Agency: Ex. Pharmacy	Contact: Ex. Dr. Jones
	Agency:	Contact:
**What will the coalition do?**		**Assigned to:**
1. Ex. Facilitate contact with Family Practice, Ambulatory Pharmacists		1. Ex. Coalition Chair
2. Ex. Help with endorsement letter (with signatures from local hospital MDs		2. Ex. Safe Prescribing Champion
**What will HCS do?**		**Assigned to:**
1. Coordinate with listed coalition members to connect with identified organizations.		1. Ex. Prevention Team
2. Draft a promotional letter/email to be reviewed and signed by community leaders who healthcare professionals respect and would positively respond to and recognize.		2. Ex. Prevention Team
3. Develop community-specific implementation plan, target list, and educational materials using audiences and messaging the coalition identifies.		3. Ex. Prevention Team and Community Coordinator
**How could we promote access/impact among diverse populations?**	Ex. Provide all patient-facing materials in Spanish, English, and any other languages deemed necessary; Prioritize County FQHC provider	
**How will we know it is working?**	Ex. Providers are willing to meet and listen/engage with continuing education materials; Providers agree to provide/request patient materials for distribution	
**What will make us change our strategy?**	Ex. Providers are not responsive or willing to engage; Educational materials are not reaching patients.	

Appendix 2. Massachusetts Action Planning Tools

Community Profile Summary Form

Purpose: Discuss and Prioritize EBP Strategies that Align with Community Goals

Description: Developing ORCCA-specific Community Goals was a multi-step iterative process driven by community teams (CEFs, CDMs, Community Faculty and CCs). Small group conversations and 1:1s were scheduled with coalition members, local stakeholders, and champions. Community Faculty set the stage with stakeholders by sharing data that demonstrated the impact a combination of initiation on medications for opioid use disorder (MOUD), improvement on MOUD retention, and increasing the distribution of naloxone could have on the HCS communities. These data facilitated conversations about the potential impact of different EBPs. Teams outlined the importance of increasing access to naloxone, MOUD initiation, and MOUD retention within the context of each community. Together, community teams and stakeholders used community profile information to contextualize these data for their community. Community profiles, which included an overview of opioid-related risk, available treatment, and fatal overdose prevention services in the community, were co-created by study staff and community members. The profile, along with local data and coalition insight was used to identify areas of opportunity where ORCCA related strategies could fill unmet needs and/or enhance extant programming. This information was compiled on the community profile summary form.

Who completes the tool: This was an iterative process that involved community teams and subcommittees soliciting feedback from coalition members, dialoging, vetting ideas and ranking solutions as they grappled with how to maximize impact among high-risk populations.

Massachusetts HCS Wave 1 Community Profile Summary

Community Profile Summary **Coalition: _______________**

ORCCA Interventions	Existing Interventions (currently taking place)	Who are the service providers?	High-risk Populations (currently receiving services)	Existing programmatic collaborations (referrals and partnerships)	Areas of opportunity to improve existing services (to identify gaps)	Sustainability Include: Current funding sources and duration	Community Narratives
**Objective 1: Increase Opioid Overdose Prevention Education and Naloxone Distribution (OEND)**							
Required OEND							
Optional OEND							
Naloxone administration (optional)							
**Objective 2: Outreach and Delivery of Medications for Opioid Use Disorder (MOUD) to High-Risk Populations***							
Expand MOUD treatment (required)							
Linkage to MOUD (required)							
Engagement and retention (required)							
**Objective 3: Improve Prescription Opioid Safety**							
Safer Prescribing/ Dispensing (required)							
Safer Disposal Practices (optional)							

*For Definitions of "high-risk populations" see the Section II of the ORCCA Overview

Action Plan Summary Form

Purpose: To comprehensively outline strategies and determine their fiscal feasibility.

Description: Once the community profile summary was complete, community teams and coalitions began the process of selecting specific evidence-based practices. This involved participating in workshops and trainings to learn more about evidenced based strategies supported by the study, conducting 1:1s to engage potential implementation partners through coalition contacts, and assessing the extent to which high-risk populations and venues were engaged with the coalition and conducting outreach. Subgroups engaged in focused conversations exploring potential evidence-based practices using knowledge from previous conversations about evidence-based practices, as well as their feasibility, impact, and sustainability.

Community partners created budgets and estimates that were included later in proposals for action plan items. The action plan summary information was then discussed in subgroup meetings. During subgroup meetings coalition members and partner organizations further flushed out details of their plans refining them for presentation to the larger coalition. At the coalition meeting action plan summaries were discussed and voted on by the coalition membership using decision-making procedures such as consensus or majority voting as outlined in their respective coalition charters.

Who completes the tool: This was an iterative process that involved community teams, subcommittees, and partner organizations.

Massachusetts HCS Wave 1 Community Profile Summary & Action Plan Template

Massachusetts Action Plan Summary **Coalition: XXX**

	Selected Strategies	ORCCA Goal (How it advances the overall HCS goal?	Targeted Populations	Primary High-Risk Sector			Specific Activities Needed to Implement Selected Strategy: (who, what, when, where)	TA Needed	HCS Funds Needed
				BH	CJ	HC			
**ORCCA Objective 2: Outreach and Delivery of MOUD to High-Risk Populations**									
MOUD Linkage (required)	H. Linkage program via embedding an "In-reach worker" at XXX County Correctional Facility	Through the addition of this staff member, inmates leaving the facility will be provided with naloxone and a MOUD treatment appointment, with transportation provided, on release date.<br><br>Worker will be responsible for: creating relationships with inmates, running harm reduction groups, distributing Narcan, making community connections, scheduling same day appointments, scheduling transportation as needed.	People with reduced tolerance post-incarceration		X		X, Y, and Z orgs will work collaboratively to create a 30-40 hour/week position to fill gap between leaving incarceration and returning home in order to support recovery and lower risk of overdose after incarceration. Facility will determine number of residents released to determine number of hours for staff to spend in the facility.<br><br>Representatives from all 3 organizations will create job description and conduct interview to ensure right fit to work with incarcerated individuals. Qualifications will include knowledge of harm reduction principals, MOUD, OEND, and local services and treatment programs for referrals and connections.<br><br>Worker will ensure Narcan kits are given to inmates upon release (8:30am everyday). Worker will assist in activating inmates MA Health and secure appointments with outside service providers to bridge MOUD treatment post-release. Worker will be deputized as TPP concierge (see below) to have access to schedule rides for inmates being released directly to MOUD appointments in the community.	Group facilitator training<br><br>BAMSI and/or HCS to provide harm reduction training.<br><br>Praxis Narcan Train the Trainer<br><br>Facilitation training	Fulltime Salaried Position for 18 months: Payroll/Fringe $74,344.31<br><br>Mileage $855.00<br><br>Phone/Computer/Supplies/PPE $1,800<br><br>Total Direct Cost: $76,999.31<br><br>Admin Fee: $7,699.31<br><br>TOTAL COST: $84,699.24

Action Plan Review Form

Purpose: To assess action plan adherence to the ORCCA menu, impact, inclusion of high-risk populations/venues, feasibility and resources.

Description: Community-specific plans approved by the coalition memberships were then reviewed by HCS MA Leadership and State partners from the department of public health using a standard review form assessing: ORCCA adherence, Impact, Inclusion of High-Risk Populations/Venues, Feasibility and Resources. Written feedback was provided to community teams who then discussed feedback with their coalitions. Leadership met with community teams and coalitions as needed to support with plan clarification or revision. Revised plans were resubmitted for approval by HCS MA Core leaders and state partners.

Who completes the tool: HCS MA Leadership and State partners from the department of public health.

For peer review only

Community:

Please comment below about the extent to which the plan meets the designated criteria, including strengths, challenges and modifications.

Approval Criteria	OEND Plan	MOUD Plan	Safer Prescribing Plan
**ORCCA Adherence**<br>☐ Within the scope of the ORCCA menu<br>☐ Aligned with the overall goal of HCS			
**Impact**<br>☐ Extent to which plan will:<br>o Increase # POUD on MOUD<br>o Retain POUD in care<br>o Increase OEND to POUD at highest risk<br>o Address gaps in the continuum of care<br>o Be sustainable			
**High-Risk Population/Venues**<br>☐ Extent to which plan will:<br>o Include efforts to engage with people who have been historically marginalized<br>o Prioritize populations at heightened risk for overdose - BH/CJ/HC (i.e. prior overdose, decreased tolerance, multi-substance use, concomitant medical or mental illness, homeless, non-English speakers, minority populations)<br>o Mitigate and not exacerbate underlying racial inequities<br>o Prevent unintended consequences			
**Feasibility**<br>☐ Extent to which plan is:<br>o Feasible to implement by January 1, 2021<br>o Clear on what will be done by who and by when to bring about change<br>☐ Extent to which structural or regulatory challenges act as a barrier for this plan			
**Resources**<br>☐ Plan is within budget			

For peer review only

Appendix 3. New York Action Planning Tools

Community Evidence Based Practice Goals

Purpose: To define community level goals for EBPs

Description: The starting point for action planning is reflection and discussion by the coalition on the Community Profile, Data Dashboard, and locally sourced data summarized in a Discussion Tool from Phase 3. This is followed by development of community goals for each ORCCA EBP. Coalitions were asked to reflect on the success of COVID-19 influenced fast tracked strategies from earlier phases and to consider expansion and sustainability of these strategies, as well as to use a health and racial equity lens in the development of community Specific, Measurable, Attainable, Realistic and Time-framed (SMART) goals for each EBP.

Who completes the tool: The community-based implementation team captures discussions and decisions from Coalition members starting in workgroups with their champions by ORCCA Menu and further facilitated in the larger coalition.

DIRECTIONS: Setting ORCCA Goals
The Implementation Team facilitates a discussion with the Coalition (Steering Committee) to identify ORCCA goals based on the recommendations of the ORCCA, Communication and Data Workgroups.

These will be community-level goals to provide OEND in high risk populations, provide outreach and delivery of MOUD to high populations, and provide for safer opioid prescribing and dispensing.

ORCCA Goals

Providing OEND in High Risk Populations	Ex. In 12 months expand OEND through four strategies: expanding peer navigation, expand virtual training, using a variety of distribution strategies, and expanding the number, integration and reach of community trainers by 50%.
Outreach and Delivery of MOUD to High-Risk Populations	Ex. In 9 months, expand access to MOUD by increasing the number of waivered MOUD prescribers and treatment slots; improving prescribing rates of current waivered prescribers; create and sustain a prescriber advisory board to support best practice; and enhancing care coordination, outreach and linkage to enhance engagement and retention in care. Use a data feedback system to improve coordination across providers and service systems and address SDH through a targeted response to increase MOUD retention.
Safer Opioid Prescribing and Dispensing	Ex. Improve safer opioid prescribing and dispensing practices by engaging prescribers and pharmacies to provide resources on safer prescribing, tapering practices, alternative pain management and promoting safer opioid disposal practices between 6-12 months from the time of implementation.

Action Planning Worksheet

<u>Purpose</u>: To identify EBP strategies, venues and priority populations in an iterative process using community profiles, and data identifying needs, gaps and opportunities

<u>Description</u>: Workgroups brainstormed possible ORCCA EBP strategies and communication messages following training and practice using the ORCCA and technical assistance guide (TAG) that could fill existing gaps with identified populations, sectors and venues discussed in Phase 3. ORCCA EBP strategies were considered that could strengthen ongoing community efforts, create opportunities for expansion and efficiency, and promote synergistic approaches to new and existing community strategies. These strategies were presented to the coalition for consideration.

<u>Who completes the tool</u>: Coalition members, workgroups led by champions from each menu and facilitated by community implementation teams

Table 1: Discussion Tool for STEP 1 (CTH PHASE 4)

Strategy	Description & Approach to Population Access (High Risk/Venue)<br>Briefly describe each specific strategy being considered.<br>For strategy, identify populations of focus and venues for reaching them.		STRATEGY<br>Label each strategy:<br>Scale up<br>New Population<br>New Feature<br>New Service<br>New Service & Population	Pros and Cons of each Strategy
	**High Risk Populations**<br>Prior Opioid Overdose<br>Reduced Opioid Tolerance<br>Other Substance Use<br>Co-occurring mental health<br>Co-occurring physical health<br>Injection Drugs	**Venues**<br>Harm Reduction/Syringe Service<br>Health Care<br>Criminal Justice<br>First Responder<br>Addiction Treatment & Recovery<br>Behavioral Health Treatment<br>Community Based -Social Service Hotline		
**OEND in High Risk Populations**				
Active OEND (required)	*EX: Distribute naloxone to released inmates at County Jail or other setting*		EX: scale up or new service	
Passive OEND (optional)	*EX: Distribute naloxone at pharmacy or dispensing program*		EX: New feature	
Naloxone administration strategies (optional)	*EX: First responder naloxone administration*		EX: scale up	
**Outreach and Delivery of MOUD to High-Risk Populations**				
Bridging MOUD (required)	*EX: bridge program for people returning to community from county jail*		EX: new service	
Linkage using Peer Navigator (required)	*EX: Linkage to peer navigation for patients who overdose in ED*		EX: new feature	
Engage and retain individuals in treatment (required)	*EX: telemedicine, virtual appointment*		EX: new feature	
**Safer Opioid Prescribing and Dispensing**				
Safer opioid prescribing/dispensing practices (required)	*EX: Emergency/urgent care*		EX: scaling up	
Safer opioid disposal practices (optional)	*EX: • Prescription drug drop-box/mail-back programs*		EX: scale up or new service	

An additional tool used to make decisions about strategies and their potential for synergy and impact, was agent-based and systems dynamic modeling. Feedback reports using this modeling were introduced to County Implementation Teams and workgroups alongside community profiles to highlight the importance of OEND and MOUD expansion specifically, in order to meet the intervention's opioid overdose prevention goals (see Figure 3), and to inform the prioritization, adaptation, maintenance and sustainability of effective interventions over time for action planning. The systems perspective is intended to be integrated into problem-solving and feedback during ongoing implementation to improve coordination and resource sharing within community networks.

For peer review only - http://bmjopen.bmj.com/site/about/guidelines.xhtml

Figure 3-NY. Systems Feedback Reports for Planning

Systems Mapping for Action Planning

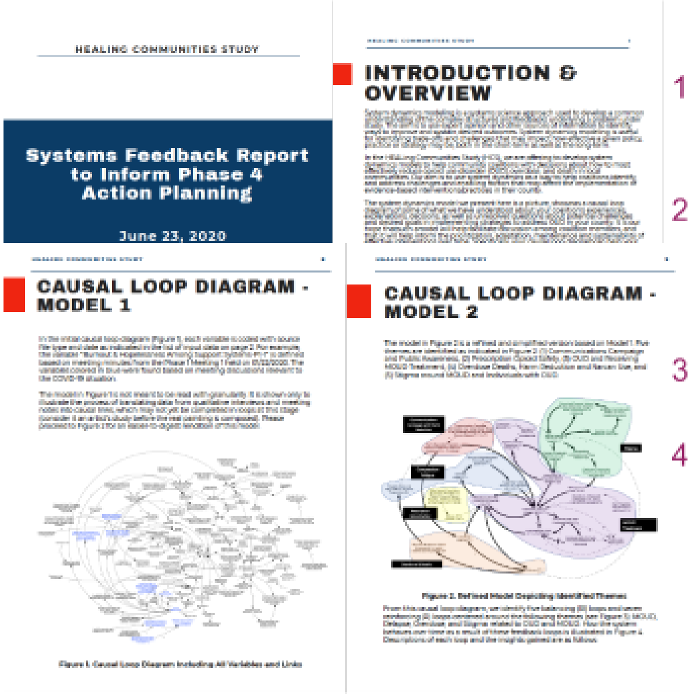

1. Are OEND and MOUD strategies effectively linked to reduce overdose and fatalities?
2. Are there supplemental interventions to support patient engagement, retention and long-term recovery?
3. How is the challenge of stigma recognized and addressed?
4. How are social determinants of health (e.g., homelessness) recognized and addressed?

5

ew only

Consultation and Coaching Plan

Purpose: To provide coaching and feedback to assure adherence to HCS required EBP selections, and enhance capacity of local teams for community planning

Description: The research study team, including implementation coaches assigned to each community, offered iterative support as Community Implementation Teams and workgroups ranked strategy options based on earlier discussion of impact and feasibility determinations in workgroups, finalized strategy selections, and refined strategies into specific SMART goals. The ranking and strategy selection process was not uniform across coalitions. While no limits were placed on the number of strategies selected, attention was given to ensure the 5 required strategies defined in the ORCCA were present or a justification for why not were included. Opportunities were highlighted during coaching sessions to reinforce data driven justification for strategy selection and to feature the role of EBP workgroup champions in leading planning.

Who completes the tool: The Study Team reviews the Action Planning Worksheet with Summary Action Plans with the Implementation Team in coaching sessions using the criteria below:

ORCCA Goals	Prioritized EBPs	Draft CAP	Adopted CAP
Goals are aligned with HCS goal to reduce opioid overdose mortality by 40%	Priorities are aligned with the ORCCA-specific goals	CAP summarizes coalition goals	Summarizes coalition goals
Goals target identified gaps in the provision of OEND, MOUD, and safer opioid prescribing and dispensing	Priorities address the gaps documented in Community Profile	CAP summarizes selected strategies	Summarizes selected strategies, venues and populations
	Workgroup prioritized strategies as high impact	CAP identifies the team responsible for implementing the strategies	Identifies the team responsible for implementation planning and organizations for initial engagement
	Workgroup prioritized strategies as high feasibility	CAP identifies and the timeframe for implementation planning	Identifies the timeframe for implementation planning
		To what degree have considered health equity and racial disparities?	Have considered health equity and racial disparities
		Initial TA and resources needed, including plans to use Community Impact (CI) Funds	TA and resources needed, including plans to use CI Funds

For peer review only

SCHEDULING REVIEW AND CONSULTATION

Implementation Teams should upload and maintain documentation for each deliverable (ORCCA Goals, prioritized EBPs and the Community Action Plan) on the Public HCS County Folders drive. Project Managers should notify the Study Team by email to request review and coaching sessions. Please use the following timetable:

Deliverable	When to Request Review	Review Type	Target Deadline	Response Time
**ORCCA Goals**	Workgroup and Coalition consensus on ORCCA goals	Review and comment	**00/00/0000**	2 Business Days
**Prioritized EBPs Draft Community Action Plan**	Prioritized EPBs and draft Community Action Plan is ready for recommendation the Coalition	Coaching Meeting	At least 1 week prior to Coalition Meeting at which EBPs and AP will be considered	2 Business Days
**MOUD and Safer Rx EBP, AP**	Consulting addiction psychiatrists will also provide consultation and review of the prioritized EBPs and draft Community Action Plan for MOUD and Safer Prescribing Workgroups during Workgroup meetings.			
**Approved Community Action Plan**	Coalition has approved the Community Action Plan	Review and comment	Next business day after Coalition approval	2 Business Days

The Implementation Teams can invite the Study Team to join the Workgroup meeting in which EBP strategies are being prioritized to provide review and consultation, if that is the preference of the Workgroup, Champion and Implementation Team.

For peer review only

Summary Community Action Plan Form

Purpose: To provide a summary of EBPs and community goals, venues and priority populations with initial organizations, and first steps to be the focus for implementation planning in Phase 5.

Description: The final step in the Phase 4 Action Planning Worksheet is a summary community action plan for final coalition approval guided by the coalition's charter on decision-making. Most used a majority vote in a coalition meeting. The plan includes defined SMART goals with an outcome target for each ORCCA EBP-related strategy and identification of specific populations, sector for implementation (i.e., behavioral health, criminal justice, healthcare), reference to feasibility and impact rankings from workgroup discussions, and preliminary sustainability. Additionally, in anticipation of Phase 5 Implementation Plan Agreement development, the action plan includes recommended initial and anticipated steps for engaging community partners and estimates of technical assistance and resources.

Who completes the tool: Community implementation teams led by a program manager and CEF complete the summary and present to the coalition for adoption. In some cases a county executive or leadership team provides an additional level of approval.

TABLE 6: Action Plan Table (CTH PHASE 4)

Strategy Category	Selected Strategies	Targeted Populations	High-Risk Sector			Sustainability Feasibility/ Impact	Implementation Steps: who does what, by when and where	Technical Assistance & Other Resources
			BH	CJ	HC			
**ORCCA Objective 1: Increase Opioid Overdose Prevention Education and Naloxone Distribution**								
Active OEND (required)	A1:<br>A2:							
Passive OEND (optional)	B1:<br>B2:							
Naloxone administration (optional)	C1:<br>C2:							
**ORCCA Objective 2: Outreach and Delivery of MOUD to High-Risk Populations**								
Expand MOUD treatment (required)	D1:<br>D2:							
Linkage to MOUD (required)	E1:<br>E2:							
Engagement and retention (required)	F1:<br>F2:							
**ORCCA Objective 3: Improve Prescription Opioid Safety**								
Safer Prescribing/ Dispensing (required)	G1:<br>G2:							
Safer Disposal Practices (optional)	H1:<br>H2:							

For peer review only - http://bmjopen.bmj.com/site/about/guidelines.xhtml

Appendix 4. Ohio Action Planning Tools

Decision Aid 1 Matrix

Purpose: To identify existing venues related to ORCCA EBPs

Description: In Ohio, the HCS Intervention Facilitator, one field team (i.e., an HCS-hired Intervention Facilitator, Community Engagement Facilitator, Data Manager) worked with community coalitions, particularly coalition-identified EBP champions to utilize a multi-step Decision Aid Approach across Phase 4 study activities. The first step was to identify existing venues related to ORCCA EBPs. In Step 1: Cataloging Possible Approaches to Population Access, a matrix was used in Decision Aid 1 to map existing locations/methods of current *or potential* population access to priority populations. This allowed communities to inventory potential points of access and also highlighted options that were not available or are already highly represented.

Who completes the tool: Decision Aid 1 is completed by ORCCA EBP coalition champions in collaboration with HCS-hired staff.

**Community "A": Decision Aid 1**		**Points of Population Access**		
		**CJ**	**SSPs**	**EDs**
**Priority Populations**	**Prior Opioid Overdose**	ABC drug court<br>XYZ County Jail	Abc street exchange	None
	**Reduced Opioid Tolerance**	ABC drug court<br>XYZ County Jail		None
	**Use Other Substances**	ABC drug court<br>XYZ County Jail	Abc street exchange	None

For peer review only

Decision Aid 2

Purpose: To identify preliminary ORCCA-related goals for each of the three EBPs.

Description: In Step 2: Existing Service Inventory Mapped by Priority Population and Point of Population Access, a similar approach to Decision Aid 1 was employed, but limited to listing *currently available* services (i.e., interventions) specific to each of the three EBPs required by ORCCA. As a first step, a community would use their population access Decision Aid 1 from Step 1 to highlight any setting in which there was a current intervention related to the three ORCCA required objectives. Next, each community would catalog any existing services that address the three ORCCA required objectives using Decision Aids 2A (OEND), 2B (MOUD), and 2C (Safer Prescribing). This approach documents the gaps between potential methods for population access and the degree to which those were already being implemented.

Who completes the tool: Decision Aid 2 is completed by ORCCA EBP coalition champions in collaboration with HCS-hired staff.

**Community "A"- Decision Aid 2B: MOUD**		**Points of Population Access**		
		**CJ**	**SSPs**	**EDs**
**Priority Populations**	**Prior Opioid Overdose**	None	Abc Syringe Exchange-treatment referral	None
	**Reduced Opioid Tolerance**	None	None	None
	**Use Other Substances**	None	Abc Syringe Exchange-treatment referral	None

For peer review only

Decision Aid 3

Purpose: To identify, prioritize, and select EBP strategies.

Description: In Step 3 of the Decision Aid process, communities in collaboration with HCS *brainstormed potential intervention strategy and population access dyads.* This approach allowed for a shared understanding of "what was currently being done" and "where it was being done" in an efficient manner that mapped structurally to ORCCA priority populations and methods of population access. The Decision Aid was not menu-specific, but rather covered strategies for intervention across menus. This provided the foundation for brainstorming which ORCCA EBP strategies *might target existing gaps.* In this step, there is an emphasis on feasibility of implementation.

Discussion Aid 3 was employed in Step 3 and prompted communities to consider whether the identified options involved:

a. bringing an existing service to scale ("1")
b. disseminate an existing service to a new population or method of population access ("2")
c. add a new feature or improvement to an existing service (i.e., changing passive referral to formal linkage intervention); ("3")
d. add a new service; ("4")
e. add both a new method of population access and a new service (e.g., a syringe service program with naloxone distribution); ("5").

Who completes the tool: Decision Aid 3 is completed by ORCCA EBP coalition champions in collaboration with HCS-hired staff.

**Community A-Decision Aid 3: Possible Strategies for Intervention—*no priority order***		**Approach to Population Access**	**Category of Implementation (1-5 above)**
**1**	Distribute OEND	XYZ County Jail	1: bring to scale
**2**	Bridging MOUD	XYZ County Jail	4: new program
**3**	Linkage to Care-Mandated Treatment	ABC Drug Court	1: bring to scale
**4**	Linkage using Peer Navigator	XYZ County Jail	4: new program
**5**	Linkage using Peer Navigator	SSP	3/4: modifying referral program/new program

For peer review only

Decision Aid 4

<u>Purpose</u>: To prioritize EBP strategies by feasibility and expected impact.

<u>Description</u>: This step involved consideration of the intersection between the importance of a given plan and the degree of difficulty in implementing that plan with the notion that EBPs that were both *highly impactful* and *highly feasible* should be undertaken before other actions that are of lower impact and/or lower feasibility. In Decision Aid 4, the identified strategies were categorized by priority/importance (low, medium, high) and by feasibility/capacity (low, medium, high)

<u>Who completes the tool</u>: Decision Aid 4 is completed by ORCCA EBP coalition champions in collaboration with HCS-hired staff.

**Community A-Decision Aid 4: Possible Strategies for Intervention—*feasibility/priority***		**Feasibility/Capacity**		
		Low	Medium	High
**Priority/Importance**	Low	• Linkage to Care Mandated Treatment at ABC Drug Court (*i.e., already at scale with little opportunity to expand*)	• Linkage using peer-navigators at XYZ county Jail (i.e. may move to high priority if bridging MOUD is unsuccessful)	
	Medium		• Distribute OEND at XYZ county jail	
	High	• Bridging MOUD at XYZ county jail	•	• Linkage using peer-navigators at SSP

For peer review only

Decision Aid 5

Purpose: To prioritize and select EBP strategies.

Description: In Step 5: Prioritizing and Selecting EBPs, communities ranked the options outlined in Step 3 in priority order based on the feasibility and impact determinations of Step 4. Coalitions were encouraged to prioritize options that were high impact and high feasibility over interventions that were of low impact or feasibility. Of note, the coalition was not limited in the number of strategies they could target for implementation. This determination was encouraged to be paired with the decision on whether the community and HCS team would pursue a given option for implementation.

Utilizing Step 5 of the Decision Aid process, communities then create their initial action plan specific to their ORCCA goals and the required ORCCA EBPs. The action plan includes the specific strategies selected, the targeted sectors for the intervention, the identified practice settings or venues, preliminary action plans, potential technical assistance or other resource needs, the individual within the Ohio Intervention Design Team (IDT), comprised of academic and subject experts, who will be primarily overseeing and assisting with the implementation of the identified strategy, and additional notes regarding the strategy. For example, if an EBP were to be conducted in an Emergency Department setting, the Emergency Department IDT would oversee the implementation of that EBP and provision of technical assistance.

Who completes the tool: Decision Aid 5 and the Action Plan is completed by ORCCA EBP coalition champions and community coalitions in collaboration with HCS-hired staff.

**Community A-Decision Aid 5: Selected Strategies for Intervention—*priority order***		**Approach to Population Access**	**Target for Action**	**Notes/Preliminary Action Plan**
**1**	Linkage using Peer Navigator	SSP	Yes	Hire program coordinator; select peers
**2**	Bridging MOUD	XYZ County Jail	Yes	
**3**	Distribute OEND	XYZ County Jail	Yes	
**3**	Linkage to Care-Mandated Treatment	ABC Drug Court	No	
**4**	Linkage using Peer Navigator	XYZ County Jail	No	Revisit in 3 months pending exploration of bridging MOUD at XYZ County Jail

For peer review only

Action Plan Template and Sample Completed Action Plan

County: ______________________

Completed By: ___ ______
Initial Completion Date: _____/____/______
Revision #: _____________
Revision Completion Date: _____/________/________

	Specific Strategy	Approach to Population Access (Sector)	Practice Setting or Venue Name	Preliminary Action Plan	Potential TA/Resource Needs	State IDT Lead	Notes
**ORCCA Objective 1: Increase Opioid Overdose Prevention Education and Naloxone Distribution**							
Active OEND (Required)							
Passive OEND (optional)							
**ORCCA Objective 2: Outreach and Delivery of MOUD to High-Risk Populations**							
Expand MOUD treatment (required)							
Linkage to MOUD (required)							
Engagement and retention (required)							
**ORCCA Objective 3: Improve Prescription Opioid Safety**							
Safer Prescribing/ Dispensing (required)							
Safer Disposal Practices (optional)							

For peer review only

Strategy category	Specific Strategy	Approach to Population Access (Sector)	Practice Setting or Venue Name	Preliminary Action Plan	Potential TA/Resource Needs	State IDT Lead	Notes
**ORCCA Objective 1: Increase Opioid Overdose Prevention Education and Naloxone Distribution**							
**Active OEND (required)**	**Active OEND for at-risk individuals and their social networks**<br><br>*Expansion of Project DAWN (a program to provide naloxone to Ohio agencies) to a secondary site*	Behavioral Health	Church	Confirm partner agencies. (HCS)<br><br>Assemble Intervention Design Team/Operations Team to provide implementation assistance. (HCS)	Resources needed on technology/ data collection, billing for sustainability.	[NAME]	Date approved by coalition: DATE
**Active OEND (required)**	**Active OEND for at-risk individuals and their social networks**<br><br>*Expansion of Project DAWN to a secondary site*	Healthcare	[AGENCY] Mobile Unit	Confirm partner agencies. (HCS)<br><br>Assemble Intervention Design Team/Operations Team to provide implementation assistance. (HCS)	Resources needed on technology/ data collection, billing for sustainability.	[NAME]	Date approved by coalition: DATE
**Active OEND (required)**	**Active OEND at high-risk venues:**<br><br>*Naloxone Referral/ Distribution for Drug Court Participants*	Criminal Justice	Drug Court	Confirm partner agencies. (HCS)<br><br>Assemble Intervention Design Team/Operations Team to provide implementation assistance. (HCS)	Potential resources needed on increasing access and participant engagement.	[NAME]	Date approved by coalition: DATE
**Active OEND (required)**	**Active OEND at high-risk venues: “Leave behind”**	Healthcare	Fire/EMS	Confirm partner agencies. (HCS)<br><br>Assemble Intervention Design Team/Operations	Potential resources needed on policy/ implementation logistics, streamlined data collection for naloxone use.	[NAME]	Date approved by

For peer review only

Strategy category	Specific Strategy	Approach to Population Access (Sector)	Practice Setting or Venue Name	Preliminary Action Plan	Potential TA/Resource Needs	State IDT Lead	Notes
	**programs at sites of overdose**<br><br>*Fire/EMS Leave-Behind Program*			Team to provide implementation assistance. (HCS)			coalition: DATE
**OEND self-request (e.g. at pharmacy, community meeting or public health department)**	**Passive OEND**<br><br>Naloxone Distribution at Pharmacies in high risk areas	Healthcare	Pharmacy	Confirm partner agencies. (HCS)<br><br>Assemble Intervention Design Team/Operations Team to provide implementation assistance. (HCS)	Potential resources needed on logistics and possible policy development.	[NAME]	Date approved by coalition: DATE
**ORCCA Objective 2: Outreach and Delivery of MOUD to High-Risk Populations**							
**Expand MOUD treatment (required)**	**Adding/expanding MOUD treatment in Criminal Justice settings**<br><br>*MOUD in the Jail*	Criminal Justice	Jail	Confirm partner agencies. (HCS)<br><br>Assemble Intervention Design Team/Operations Team to provide implementation assistance. (HCS)	Potential resources needed on logistics, billing, etc.	[NAME]	Date approved by coalition: DATE
**Linkage to MOUD**<br><br>**(required)**	**Linkage Programs (all relevant settings)**<br><br>*Expand MOUD referral in ED*	Healthcare	Emergency Department	Confirm partner agencies. (HCS)<br><br>Identify opportunities for expansion of current services.<br><br>Assemble Intervention Design Team/Operations Team to provide implementation assistance. (HCS)	Potential resources needed on sustainability and patient engagement.	[NAME]	Date approved by coalition: DATE

For peer review only

Strategy category	Specific Strategy	Approach to Population Access (Sector)	Practice Setting or Venue Name	Preliminary Action Plan	Potential TA/Resource Needs	State IDT Lead	Notes
**Engagement and retention (required)**	**Reducing barriers to housing, transportation, childcare and accessing other community benefits for people with OUD**<br><br>*Reduce barriers to transportation to improve engagement/retention in treatment*	Behavioral Health	MOUD Treatment Facility<br><br>Potentially other MOUD treatment facilities with limited transportation options. (TBD)	Confirm partner agencies. (HCS)<br><br>Assemble Intervention Design Team/Operations Team to provide implementation assistance. (HCS)<br><br>Engage partners and IDT through an initial planning meeting. (HCS)	Potential resources needed on billing for sustainability.<br><br>Needs: Identify potential partnerships between XXX transportation and treatment agencies	[NAME]	Date approved by coalition: DATE
**ORCCA Objective 3: Improve Prescription Opioid Safety**							
**Safer Prescribing/ Dispensing (required)**	**Safer opioid prescribing for chronic pain**<br><br>Prescriber Accountability through OD Review	Healthcare	Pharmacy	Confirm partner agencies. (HCS)<br><br>Assemble Intervention Design Team/Operations Team to provide implementation assistance. (HCS)<br><br>Engage partners and IDT through an initial planning meeting. (HCS)	Potential resources needed on logistics and identifying allowable information to be shared between the XXX team and XXX Agency.	[NAME]	Date approved by coalition: DATE
**Safer Prescribing/ Dispensing (required)**	**Safer opioid dispensing**<br><br>Utilize the Take Charge Ohio Toolkit for pharmacist and patient education. (Co-dispensing naloxone, providing educational materials	Healthcare	Pharmacy	Engage Education and Awareness workgroup of Scioto County Collaborative Opioid Consortium.<br><br>Determine which parts of the Toolkit will be utilized.<br><br>Confirm partner agencies. (HCS)	Potential resources needed on logistics and possible policy development.	[NAME]	Date approved by coalition: DATE

For peer review only

Strategy category	Specific Strategy	Approach to Population Access (Sector)	Practice Setting or Venue Name	Preliminary Action Plan	Potential TA/Resource Needs	State IDT Lead	Notes
	on opioids and naloxone)			Assemble Intervention Design Team/Operations Team to provide implementation assistance. (HCS)			

For peer review only
